# Rhodium(I)-Catalyzed Defluorinative Bisarylation of Monofluorodienes with Boronic Acids

**DOI:** 10.1021/acs.orglett.4c00383

**Published:** 2024-03-11

**Authors:** Min Li, Gavin Chit Tsui

**Affiliations:** † Department of Chemistry, The Chinese University of Hong Kong, Shatin, New Territories, Hong Kong SAR 999077, China

## Abstract

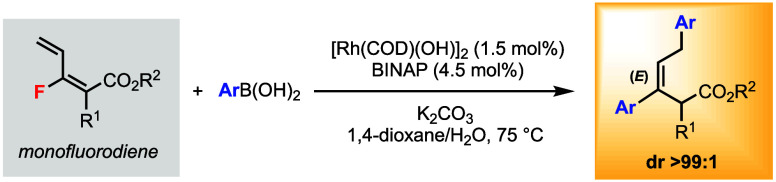

We herein describe a Rh(I)-catalyzed bisarylation reaction of monofluorodienes using arylboronic acids. Two aryl groups are installed in the trisubstituted (*E*)-alkene products in one step with excellent diastereoselectivities. An intriguing reaction sequence of Rh(I)-catalyzed 1,6-addition followed by defluorinative coupling is proposed for product formation.

Rhodium(I)-catalyzed addition reaction of organometallic reagents to alkenes is a powerful strategy for the construction of carbon–carbon bonds.^1^ The use of organoboron reagents, especially boronic acids,^2^ is highly attractive in such transformations due to their commercial availability, stability, and low toxicity. It has been well-established that *α,β*-unsaturated carbonyl compounds and styrene derivatives can undergo Rh(I)-catalyzed conjugate addition with organoboron reagents.^3^ On the other hand, much less is known about the reactions of *fluoroalkenes* under Rh(I) conditions.^4^

We have recently reported stereoselective Rh(I)-catalyzed arylation reactions of β-fluoroacrylate derivatives with arylboronic acids.^5^ For instance, Rh(I)-catalyzed defluorinative coupling of (*E*)-monofluoroalkenes could generate trisubstituted (*Z*)-alkene products with inversion of double bond geometry (Scheme 1a).^5a^ Also, Rh(I)-catalyzed C–F bond arylation of *gem*-difluoroalkenes could give access to tetrasubstituted (*E*)-monofluoroalkene products (Scheme 1b).^5b^ In this work, we describe an unprecedented Rh(I)-catalyzed defluorinative *bisarylation* reaction of (*E*)-*monofluorodienes***1**, which led to the synthesis of a new class of trisubstituted (*E*)-alkene products **2** with the cleavage of a C–F bond and formation of two C–C bonds (Scheme 1c).

**Scheme 1:**
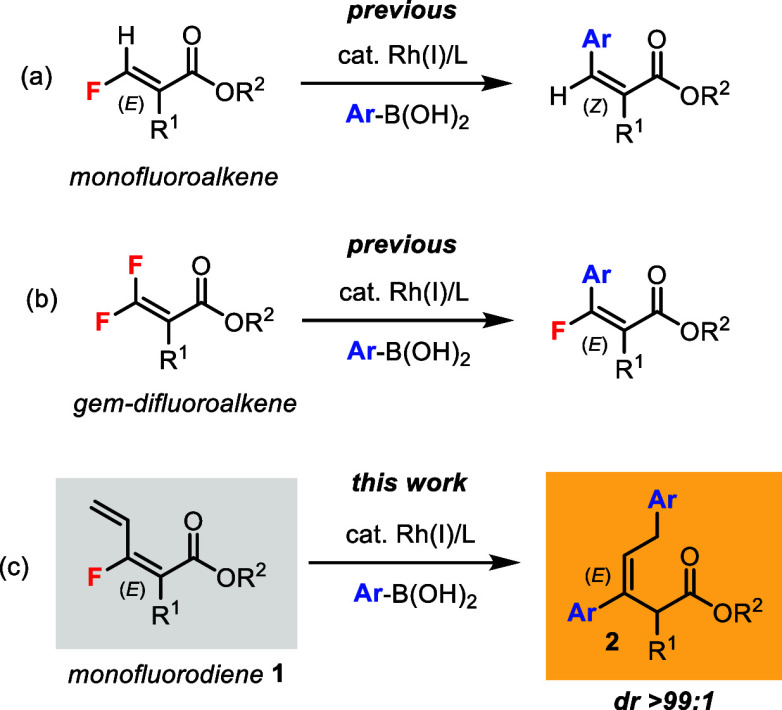
Rh(I)-Catalyzed Coupling Reactions of Fluoroalkenes with Boronic Acids

Monofluorinated 1,3-diene substrate (*E*)-**1a** was prepared from the corresponding β,β-difluoroacrylate via our Pd-catalyzed stereoselective C–F bond vinylation (Stille coupling) protocol.^6^ On closer inspection, **1a** is also a special type of *α,β,γ,δ*-diunsaturated ester with α-phenyl and β-fluoro substituent groups and well-defined tetrasubstituted alkene geometry. While Rh(I)-catalyzed 1,4-addition of organoboron reagents to *α,β*-unsaturated esters has been reported,^7,1^ the corresponding 1,6-addition to 2,4-dienoate esters is less studied.^8^ In the literature, Rh(I)-catalyzed reactions of arylboronic acids with 2,4-dienoate esters could lead to a mixture of 1,4- and 1,6-addition products.^9^ To the best of our knowledge, no example of addition reactions to *monofluorinated* dienoate ester is known.

When reacting **1a** with PhB(OH)_2_ under previously developed Rh(I)-catalyzed coupling conditions,^5a^ the 1,6-addition product **3a** was only observed in trace amounts. To our surprise, a *bisphenylated* product (*E*)-**2a** was obtained in excellent yield (94%) and dr (>99:1) (Table 1, entry 1). The effects of various reaction parameters on the formation of **2a** were subsequently investigated.^10^ The reaction yield dropped dramatically without the ligand BINAP, and no reaction took place without the Rh(I) catalyst (entries 2–3). Replacing [Rh(COD)(OH)]_2_ with [Rh(COD)Cl]_2_ or Rh(COD)_2_OTf led to lower yields (entries 4–5). Boronic acid gave higher yields than trifluoroborate or boronic ester (entries 6–7). Reactions run in DMF or toluene led to lower yields (entries 8–9). Compound **3a** was detected as a major side product under these conditions. At 25 °C, **3a** was formed exclusively (entry 10).

**Table 1:**
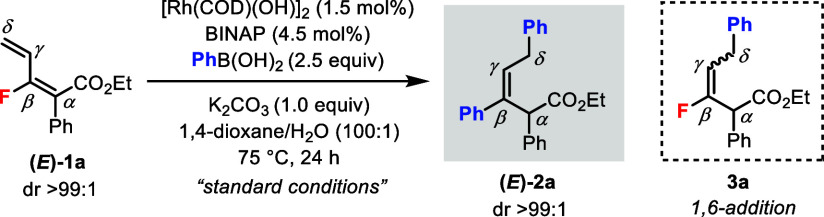
Effects of Reaction Parameters on the Formation of **2a**^a^

entry	deviation from standard conditions	yield of **2a** (%)^b^
1	none	**94 (90)**^c^
2	no BINAP	23
3	no [Rh(COD)(OH)]_2_	0
4	[Rh(COD)Cl]_2_, instead of [Rh(COD)(OH)]_2_	15
5	Rh(COD)_2_OTf (3.0 mol %), instead of [Rh(COD)(OH)]_2_	44
6	PhBF_3_K, instead of PhB(OH)_2_	34
7	PhBpin, instead of PhB(OH)_2_	<5
8	DMF, instead of 1,4-dioxane	23
9	toluene, instead of 1,4-dioxane	54
10	25 °C	0^d^

a Unless specified otherwise, reactions were carried out using (*E*)-**1a** (0.1 mmol) under argon.

b Yield was determined by GC-MS.

c Isolated yield at 0.2 mmol scale. Diastereomeric ratio of **2a** (dr >99:1) was determined by GC-MS and ^1^H NMR analyses.

d Isolated **3a** in 93% yield as a mixture of *E*/*Z* isomers.

The generality of the bisarylation reaction was subsequently studied by varying the boronic acids and monofluorodienes **1** (Scheme 2). The wide commercial availability of aryl and heteroaryl boronic acids was advantageous for diverse functionalization. For instance, aromatic rings containing electron-poor (**2b**), electron-rich (**2c**), halogen (**2d**–**f**), cyano (**2g**), nitro (**2i**), aldehyde (**2k**), and ester (**2l**) substituents were tolerated. Heteroaromatic groups such as thiophene (**2m**) and furan (**2n**) were also compatible. The reaction could be performed at a 1.0 mmol scale in good yield (86%, **2a**). The *E*-alkene geometry was established by NOESY NMR experiments through **2c**.^10^ The α-substituent R^1^ of monofluorodiene **1** could be varied including arenes containing electron-poor (**2o**)/electron-rich (**2p**)/halogen (**2q**–**r**) groups, naphthalene (**2t**), and thiophene (**2u**). The ester substituent group R^2^ could also be changed including isopropyl (**2v**) and benzyl (**2w**) groups. These variations did not affect the diastereoselectivity of the reaction, affording only one diastereomer (*E*)-**2**. Furthermore, a modular approach by choosing different combinations of boronic acids and substrates allowed us to synthesize various polyaromatic compounds **2x**–**2aa** with unique structural features.

**Scheme 2:**
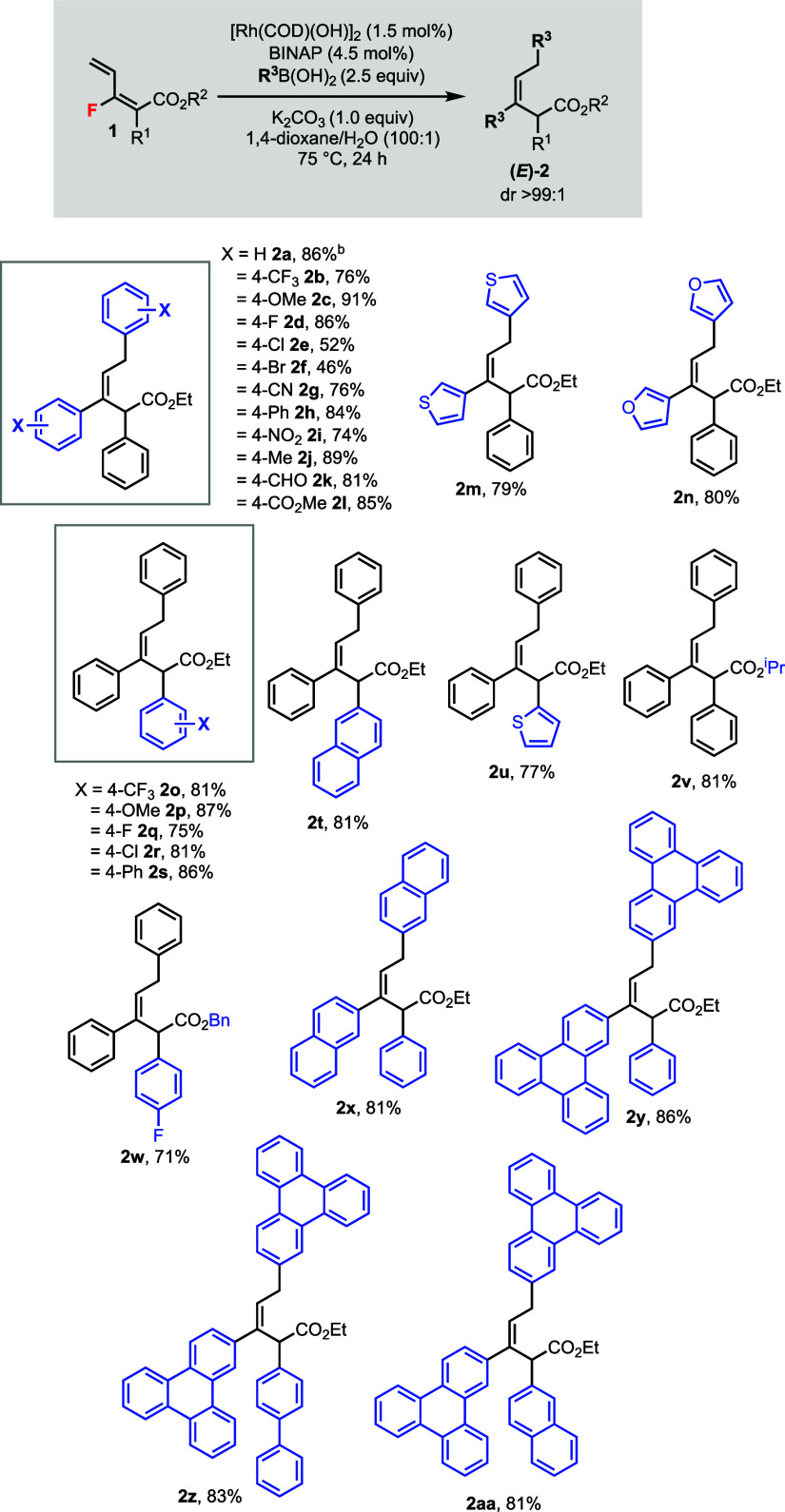
Rh(I)-Catalyzed Bisarylation of Monofluorodienes **1** Unless specified otherwise, reactions were carried out using **1** (0.2 mmol) under argon. Isolated yields. Diastereomeric ratios of **2** (dr >99:1) were determined by GC-MS and ^1^H NMR analyses. At 1.0 mmol scale.

During the optimization studies, the 1,6-addition product **3a** was found to be a side product in the reaction (cf. Table 1).^10^ We subsequently learned that using the catalyst [Rh(COD)(OH)]_2_ without BINAP at 25 °C could generate **3a** (80% iso. yield) exclusively from **1a** (Scheme 3a). Other boronic acids were also effective in the 1,6-addition providing products **3b**–**e** in good yields. However, the diastereoselectivities of **3** were rather low, ranging from ∼1:1 to 7:1. Intriguingly, by resubmitting **3a** (dr = 1.2:1) to the standard conditions, the bisarylated product **2a** was obtained in >99:1 dr (Scheme 3b), which indicated that the 1,6-addition product was a potential intermediate for the bisarylation product.

**Scheme 3:**
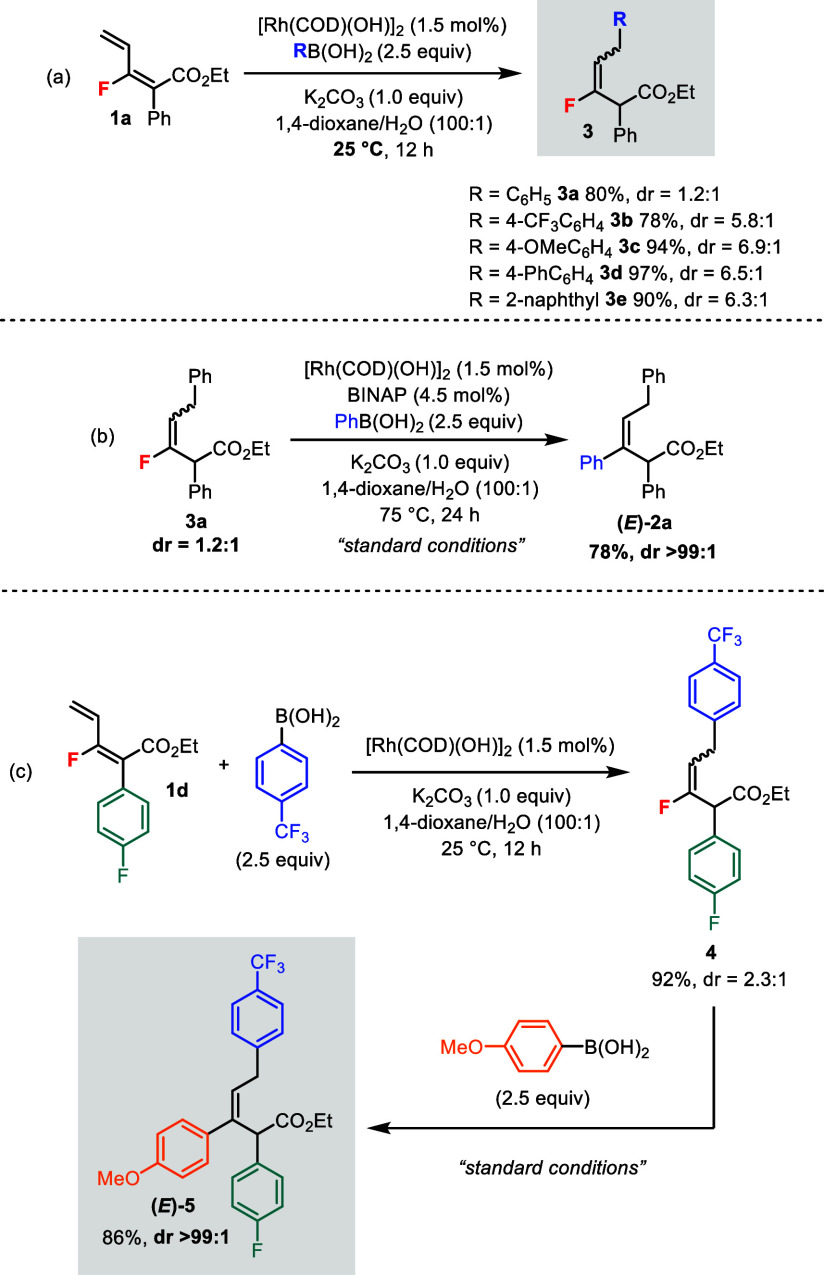
Rh(I)-Catalyzed 1,6-Addition of Monofluorodienes **1** and Application in Modular Synthesis

Based on this observation, we devised a modular synthesis of a triaryl compound **5** where each aryl substituent group was different (Scheme 3c). Monofluorodiene **1d** containing a 4-fluorobenzene unit was reacted with 4-(trifluoromethyl)phenylboronic acid in Rh(I)-catalyzed 1,6-addition to give intermediate **4** (dr = 2.3:1). Arylation of **4** using 4-methoxyphenylboronic acid under the standard conditions gave the final product (*E*)-**5** in excellent dr (>99:1). Thus, each of the three aromatic rings of **5** could be tuned for desirable electronic and steric properties, which should be attractive to medicinal chemists for lead compound screening.

The bisarylation reaction was not limited to dienoate esters. Monofluorodiene **6** containing an *amide* moiety was prepared and subjected to the standard conditions (eq 1). The bisarylated product (*E*)-**7** could be isolated in excellent dr (>99:1).


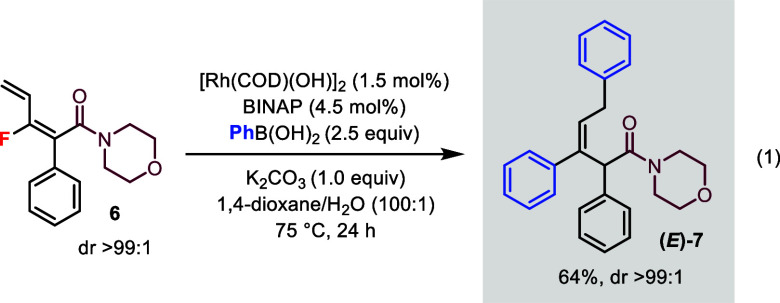
1

Further experiments were conducted to understand the reaction mechanisms (Scheme 4). Monofluorodiene **8** without the ester group or **9** without the terminal alkene did not react under the standard conditions (Scheme 4a). Using a *chiral* ligand (*R*)-BINAP gave the product (*E*)-**2a** in 24% *ee* (Scheme 4b). We have also screened a variety of other chiral ligands under the standard conditions, the highest *ee* (34%) was obtained from (*R*)-DTBM-SEGPHOS.^10^ Moreover, switching the substrate to (*Z*)-**1a** under identical conditions provided the same product (*E*)-**2a** in excellent dr (>99:1) and yield, with 15% *ee* (Scheme 4c). Thus, the *E*/*Z* configuration of monofluorodiene **1** does not influence the diastereomeric outcome of product **2**. By subjecting the 1,6-addition product **3a** as a diastereomeric mixture (dr = 1.4:1) to the Rh-catalyzed conditions without the boronic acid, we observed an *isomerization* process favoring the (*Z*)-product in excellent dr (>99:1) (Scheme 4d). The same trend was also observed for analogues **3b**–**e**. Resubmitting (*Z*)-**3a** to the standard conditions led to the bisphenylated product (*E*)-**2a** in good yield and excellent dr (>99:1) (Scheme 4e).

**Scheme 4:**
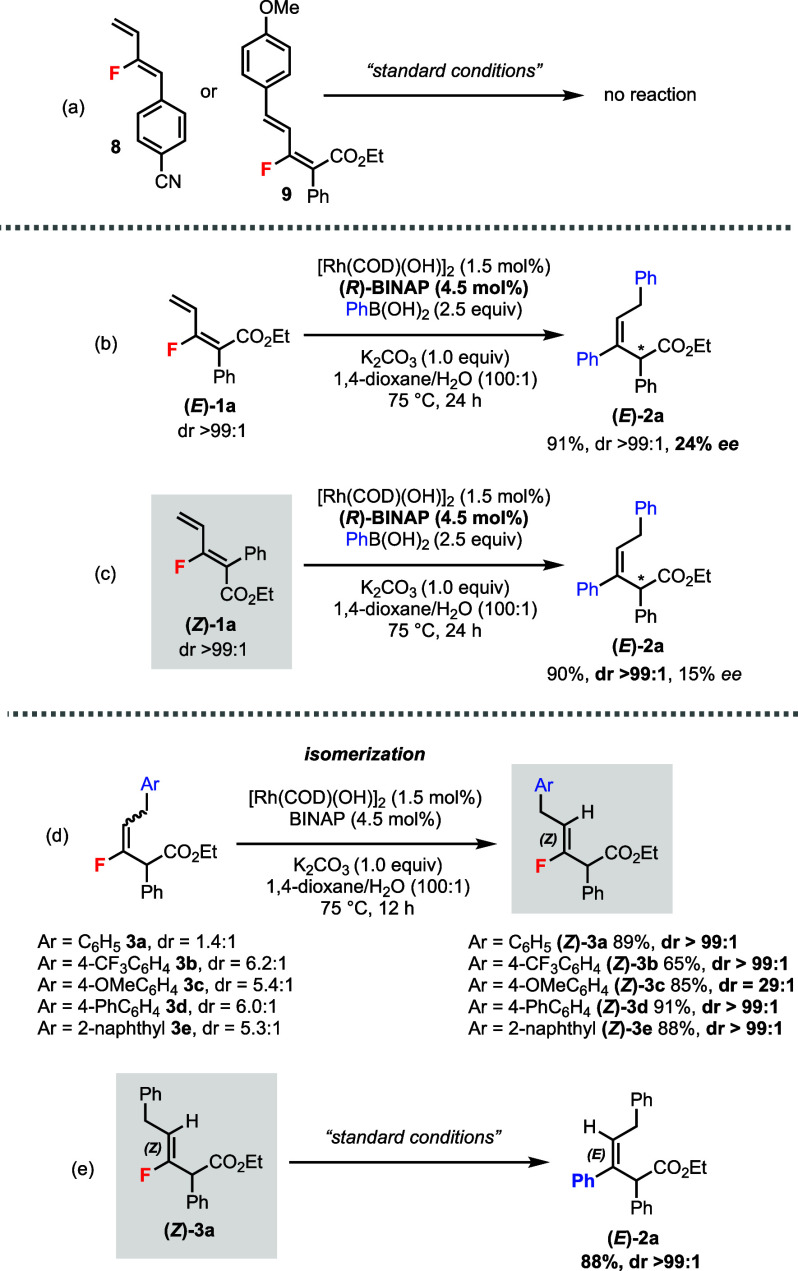
Further Experiments To Gain Mechanistic Insights

Based on the above studies and known literature reports, we proposed the following plausible reaction mechanism for the stereoselective Rh(I)-catalyzed defluorinative bisarylation of monofluorodiene **1** (Scheme 5). Transmetalation between the Rh(I) catalyst and arylboronic acid generates the Rh(I)-Ar species.^1^ Regioselective migratory insertion of Rh(I)-Ar to the terminal double bond of **1** gives the alkyl-Rh(I) intermediate **A**. Isomerization of **A** leads to the oxo-π-pentadienyl-Rh(I) complex **B**.^9^ Protonolysis of **B** with water forms the 1,6-addition product **3** and regenerates the Rh(I) catalyst.^11^ There are two possibilities for the outcome of the alkene geometry of **3**. (1) The isomerization of **B** favors (*Z*)-**3** at 75 °C.^12^ (2) Both (*E*)- and (*Z*)-**3** were formed; however, under the reaction conditions (*E*)-**3** can isomerize to (*Z*)-**3**. We have experimental evidence for such isomerization (cf. Scheme 4d) although the exact mechanism is unclear at the moment. The acidic α-proton under the basic reaction conditions likely facilitates the isomerization.

**Scheme 5:**
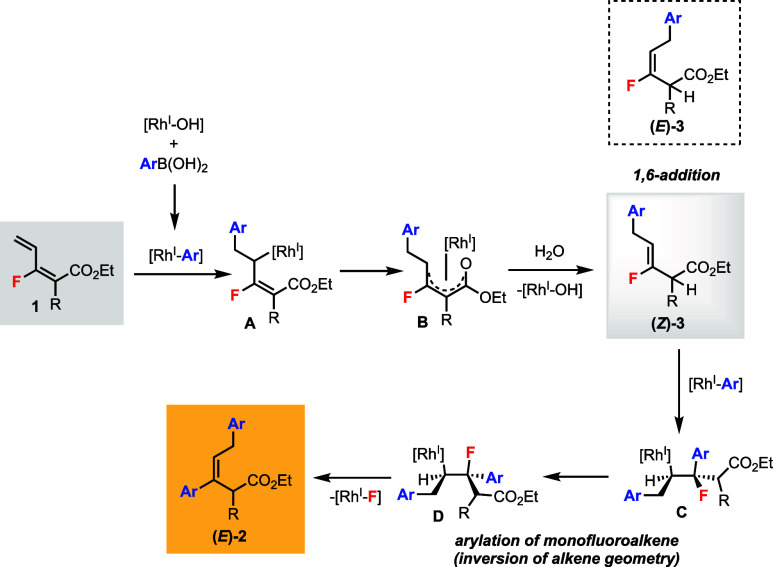
Proposed Mechanism

Monofluoroalkene (*Z*)-**3** undergoes migratory insertion with another 1 equiv of Rh(I)-Ar to generate alkyl-Rh(I) **C**. Bond rotation leads to conformer **D**, which is set up for *syn*-β-F elimination. Final product **2** is therefore formed in the *E*-alkene configuration, and Rh(I)-F is eliminated to re-enter the catalytic cycle. Control experiment showed (*Z*)-**3a** can generate (*E*)-**2a** in good yield and excellent dr (cf. Scheme 4e). Overall, there is an *inversion* of alkene geometry from (*Z*)-**3** to (*E*)-**2**, which is consistent with our previous Rh(I)-catalyzed defluorinative coupling of monofluoroalkenes.^5a^ This could be a situation of dynamic kinetic resolution (DKR) in the case where both (*E*)- and (*Z*)-**3** are present but only (*Z*)-**3** continues to react and (*E*)-**3** isomerizes to (*Z*)-**3**.^13^ This explains why the *E*/*Z* mixtures of monofluoroalkenes **3**/**4** only gave the (*E*)-products (cf. Scheme 3b-c). Also, substrates (*E*)- and (*Z*)-**1a** can give the same product (*E*)-**2a** (cf. Scheme 4b-c) because they converge to the same intermediate (*Z*)-**3a**.

In conclusion, we have discovered a novel Rh(I)-catalyzed bisarylation reaction of monofluorodienes **1** using arylboronic acids. The method allowed the synthesis of an array of trisubstituted alkene products (*E*)-**2** containing two newly installed aryl groups with excellent diastereoselectivities. The reaction mechanism presumably involves the combination of two Rh(I)-catalyzed sequences: (1) 1,6-addition of monofluorodiene **1** to generate the monofluoroalkene **3**; (2) defluorinative arylation of monofluoroalkene **3** to form product **2**. The enantioselective version of this reaction is ongoing in our laboratories.

## Data Availability

The data underlying this study are available in the published article and its Supporting Information.

## Supporting Information Available

Experimental procedures, optimization data, characterization data, and spectral data (PDF)
