# Supplementary material for: Rhodium(I)-Catalyzed Defluorinative Bisarylation of Monofluorodienes with Boronic Acids
Source: Org Lett. 2024 Mar 11;26(11):2223–7. doi: 10.1021/acs.orglett.4c00383 (PMC10964235; doi:10.1021/acs.orglett.4c00383)
Supplement: Supplementary file 1 — ol4c00383_si_001.pdf [file ol4c00383_si_001.pdf]

Supporting Information

Rhodium(I)-Catalyzed Defluorinative Bisarylation of Monofluorodienes with Boronic Acids

*Min Li and Gavin Chit Tsui**
** Department of Chemistry, The Chinese University of Hong Kong, Shatin, New Territories, Hong Kong SAR*
E-mail: gctsui@cuhk.edu.hk

Experimental Procedures and Spectral Data

Table of Contents:

I.	General Experimental	**S2**
II.	Materials	**S2**
III.	Instrumentation	**S2**
IV.	Experimental Procedures	**S4**
V.	^{19}H-^{1}H NOESY NMR Studies	**S6**
VI.	The DEPT 135 experiments	**S7**
VII.	Optimization studies	**S8**
VIII.	Screening of chiral ligands	**S9**
IX.	Application	**S10**
X.	Further experiment	**S11**
XI.	References	**S13**
XII.	Characterization Data	**S14**
XIII.	Spectra	**S30**

I. General Experimental.

Unless otherwise noted, cross-coupling reactions were carried out under argon in a 10 mL glass tube with magnetic stirring. Reactions that require heating were carried out in the oil bath. Analytical thin layer chromatography (TLC) was performed with Merck silica gel 60 F_{254} aluminum plates. Visualization was done under a UV lamp (254 nm) and by immersion in potassium permanganate ($KMnO_4$), followed by heating using a heat gun. Organic solutions were concentrated by rotary evaporation at 23-35 °C. Purification of reaction products were generally done by flash column chromatography with Silicycle 60-230 mesh silica gel.

II. Materials.

Anhydrous K_2CO_3 was purchased from Farco. BINAP was purchased from J&K Scientific. BINAP was purchased from J&K. $[Rh(COD)(OH)]_2$ was purchased from TCI. Boronic acids were purchased from Energy Chemical and J&K Scientific. Monofluorodienes for substrates synthesis were prepared according to literature procedure. Other chemicals for substrates preparation were purchased from Acros, J&K Scientific, Aldrich and Dieckmann.

III. Instrumentation.

Proton nuclear magnetic resonance spectra (^{1}H NMR), carbon nuclear magnetic resonance spectra (^{13}C NMR) and fluorine nuclear magnetic resonance spectra (^{19}F NMR) were recorded at 23 °C on Bruker 400 MHz or 500 MHz spectrometer in $CDCl_3$. Chemical shifts of ^{1}H NMR spectra were reported as parts per million in δ scale using residual solvent signal ($CDCl_3$: 7.26 ppm) or tetramethylsilane (0.00 ppm) as internal standard. Chemical shifts of ^{13}C NMR spectra were reported using residual solvent signal of $CDCl_3$ (77.16 ppm) on the δ scale. Chemical shifts of ^{19}F NMR were reported as parts per million in δ scale using benzotrifluoride (-63.72 ppm) as internal standard. Data are represented as follows: chemical shift (δ ppm), multiplicity (s = singlet, d = doublet, t = triplet, q = quartet, m = multiplet), coupling constant (J, Hz) and integration. GC-MS analysis results were obtained on a Shimadzu GCMSQP2010 SE GC-MS Spectrometer. High resolution mass spectra (HRMS) were obtained on a Finnigan MAT 95XL GC Mass Spectrometer or a Thermo Scientific Q Exactive Focus Mass Spectrometer or a Bruker SolariX 9.4T FTMS (mass analyzer type: orbitrap)

Substrates 1

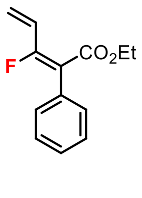

(*E*)-**1a**

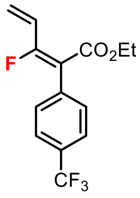

(*E*)-**1o**

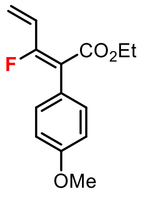

(*E*)-**1p**

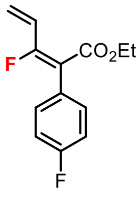

(*E*)-**1q**

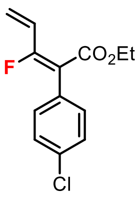

(*E*)-**1r**

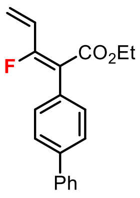

(*E*)-**1s**

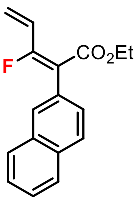

(*E*)-**1t**

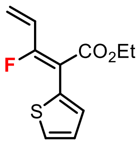

(*E*)-**1u**

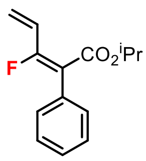

(*E*)-**1v**

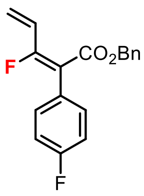

(*E*)-**1w**

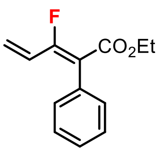

(*Z*)-**1a**

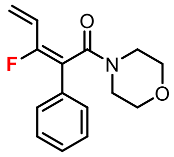

(*E*)-**6**

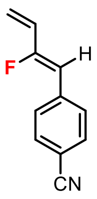

8

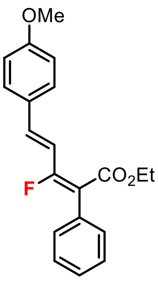

9

Note: Substrates **1** and **6** are known compounds and prepared according to the literature procedure.[1, 2] Substarte **8** was synthesized according to literature procedures.[3] Substrates **9** was synthesized according to literature procedures.[4]

IV. Experimental Procedures.

General procedure (I) for the synthesis of monofluorodienes 1 (using 1a as an example):

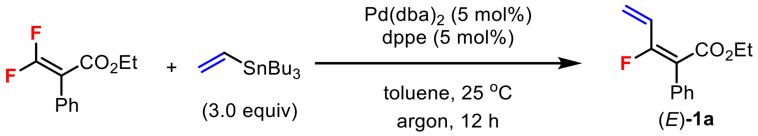

Monofluorodienes **1** were synthesized according to literature procedure.[1] For example, to an oven-dried glass tube equipped with a stir bar was added Pd(dba)$_2$ (5.8 mg, 0.01 mmol) and dppe (4.0 mg, 0.01 mmol). The tube was sealed with a septum, evacuated and refilled with argon three times. A solution of *gem*-difluoroalkene (0.2 mmol) in 1.0 mL toluene was added under argon through syringe, followed by the addition of tributyl(vinyl)tin (190.3 mg, 0.6 mmol) under argon through syringe. The resulting mixture was stirred at 25 °C for 12 h. The crude sample was analyzed by ^{19}F NMR using benzotrifluoride (12 µL, 0.1 mmol) as internal standard and the *E/Z* ratio (>99:1) was determined. The crude mixture was extracted with CH_2Cl_2 (3 × 10 mL), the combined organic layers were washed with H_2O (2 × 10 mL) then brine (2 × 10 mL), dried over $MgSO_4$ and concentrated *in vacuo*. The residue was purified by flash column chromatography on silica gel to afford monofluorodiene (*E*)-**1a** as a colorless oil (39.8 mg, 94% yield).

General procedure (II) for the synthesis of monofluorodienes (*Z*)-1a:

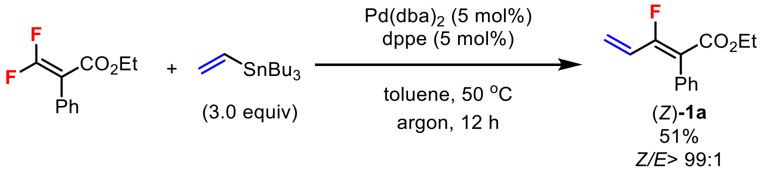

According to literature procedure[1], to an oven-dried glass tube equipped with a stir bar was added Pd(dba)$_2$ (5.8 mg, 0.01 mmol) and dppe (4.0 mg, 0.01 mmol). The tube was sealed with a septum, evacuated and refilled with argon three times. A solution of *gem*-difluoroalkene (0.2 mmol) in 1.0 mL toluene was added under argon through syringe, followed by the addition of tributyl(vinyl)tin (190.3 mg, 0.6 mmol) under argon through syringe. The resulting mixture was stirred at 50 °C for 12 h. The crude sample was analyzed by ^{19}F NMR using benzotrifluoride (12 µL, 0.1 mmol) as internal standard and the *E/Z* ratio was determined. The crude mixture was extracted with CH_2Cl_2 (3 × 10 mL), the combined organic layers were washed with H_2O (2 × 10 mL) then brine (2 × 10 mL), dried over $MgSO_4$ and concentrated *in vacuo*. The residue was purified by flash column chromatography on silica gel to afford monofluorodiene (*Z*)-**1a** as a colorless oil (22.4 mg, 51% yield).

General procedure (III) for the Rhodium(I)-catalyzed defluorinative bisarylation of (het)aryl-substituted monofluorodienes:

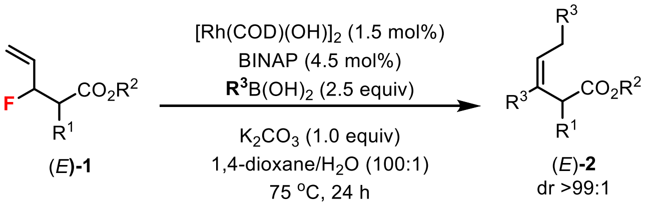

To an oven-dried glass tube equipped with a stir bar was added K_2CO_3 (27.6 mg, 0.2 mmol), BINAP (5.6 mg, 0.009 mmol) and [Rh(COD)(OH)]$_2$ (1.4 mg, 0.003 mmol). The tube was sealed with a septum, evacuated and refilled with argon three times. Then 1.4-dioxane (0.6 mL) was added and the mixture was stirred for 10 min in a 50 °C oil bath, the solution turned reddish. (*E*)-**1** (0.2 mmol) and boronic acid (0.5 mmol) were added together as a solution in 1,4-dioxane (1.0 mL) and H_2O (16 µL). The resulting mixture was heated at 75 °C with stirring in an oil bath for 24 h. After cooling to room temperature, the crude sample was analyzed by GC-MS and the *E/Z* ratio (>99:1) was determined. The crude mixture was extracted with CH_2Cl_2 (3 × 10 mL), the combined organic layers were washed with H_2O (2 × 10 mL) then brine (2 × 10 mL), dried over $MgSO_4$ and concentrated *in vacuo*. The residue was purified by flash column chromatography on silica gel to afford products (*E*)-**2** (*E/Z* >99:1), dr was determined by ^{1}H NMR.

General procedure (IV) for the Rhodium(I)-catalyzed defluorinative 1,6 addition of aryl-substituted monofluorodienes:

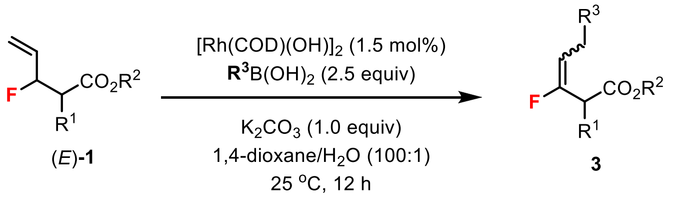

To an oven-dried glass tube equipped with a stir bar was added K_2CO_3 (27.6 mg, 0.2 mmol) and $[Rh(COD)(OH)]_2$ (1.4 mg, 0.003 mmol). The tube was sealed with a septum, evacuated and refilled with argon three times. Then 1.4-dioxane (0.6 mL) was added and (*E*)-**1** (0.2 mmol) and boronic acid (0.5 mmol) were added together as a solution in 1,4-dioxane (1.0 mL) and H_2O (16 μL). The resulting mixture was stirred at 25 °C for 12 h. The crude sample was analyzed by ^{19}F NMR using benzotrifluoride (12 μL, 0.1 mmol) as internal standard and the *E/Z* ratio was determined. The crude mixture was extracted with CH_2Cl_2 (3 × 10 mL), the combined organic layers were washed with H_2O (2 × 10 mL) then brine (2 × 10 mL), dried over $MgSO_4$ and concentrated *in vacuo*. The residue was purified by flash column chromatography on silica gel to afford products **3**.

1.0 mmol scale synthesis of (*E*)-2a:

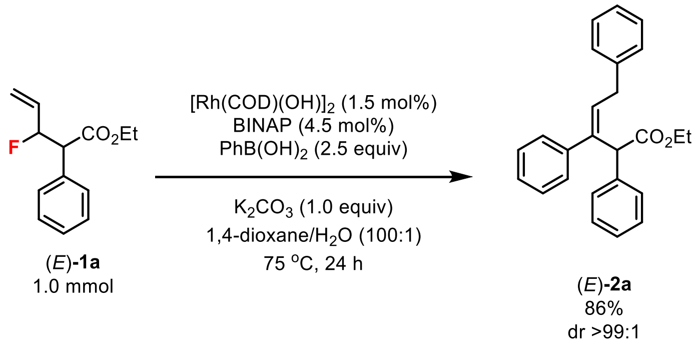

To an oven-dried 25 mL flask equipped with a stir bar was added K_2CO_3 (138.2 mg, 1.0 mmol), BINAP (28.0 mg, 0.045 mmol) and $[Rh(COD)(OH)]_2$ (6.8 mg, 0.015 mmol). The tube was sealed with a septum, evacuated and refilled with argon three times. Then 1.4-dioxane (3.0 mL) was added and the mixture was stirred for 10 min in a 50 °C oil bath, the solution turned reddish. (*E*)-**1** (220.1 mg, 1.0 mmol) and boronic acid (305.1 mg, 2.5 mmol) were added together as a solution in 1,4-dioxane (5.0 mL) and H_2O (80 μL). The resulting mixture was heated at 75 °C with stirring in an oil bath for 24 h. After cooling to room temperature, the crude sample was analyzed by GC-MS and the *E/Z* ratio (>99:1) was determined. The crude mixture was extracted with CH_2Cl_2 (3 × 20 mL), the combined organic layers were washed with H_2O (2 × 20 mL) then brine (2 × 20 mL), dried over $MgSO_4$ and concentrated *in vacuo*. The residue was purified by flash column chromatography on silica gel (DCM : hexane = 1 : 5) to afford (*E*)-**2a** as a colorless oil (306.6 mg, 86%, *E/Z* >99:1).

V. ^{1}H-^{1}H NOESY NMR Studies.

2D NMR experiments were carried out on a Bruker 400 MHz spectrometer in $CDCl_3$ at 23 °C.

^{1}N-^{1}H HOESY NMR experiment for product (*E*)-2c:

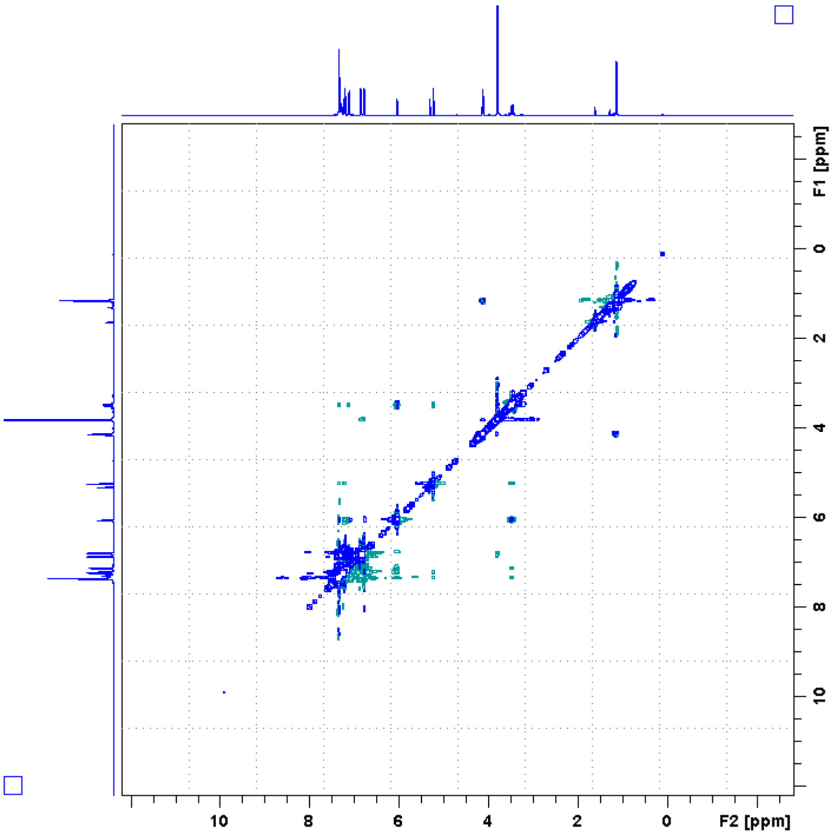

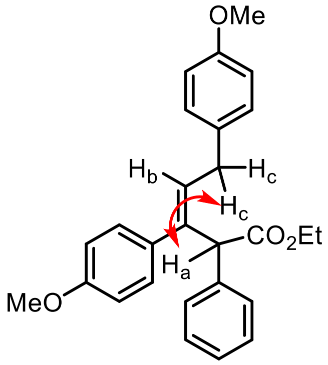

Observed ^{1}H-^{1}H NOE

(*E*)-2c

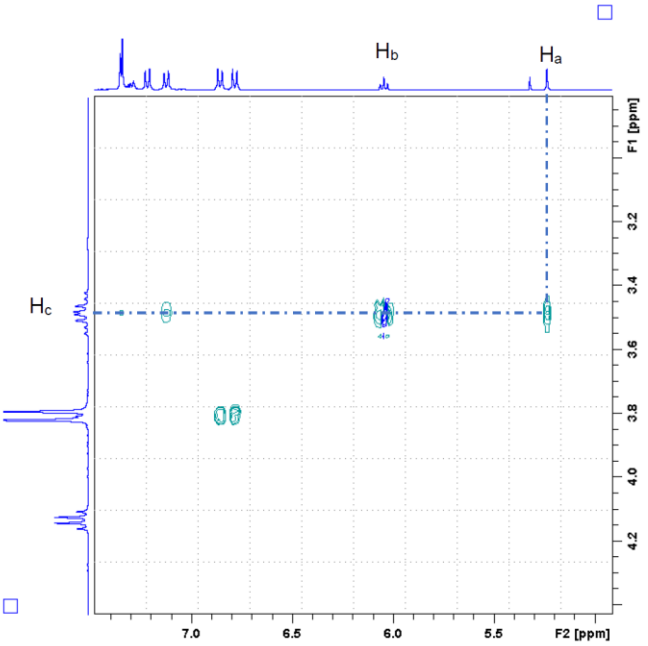

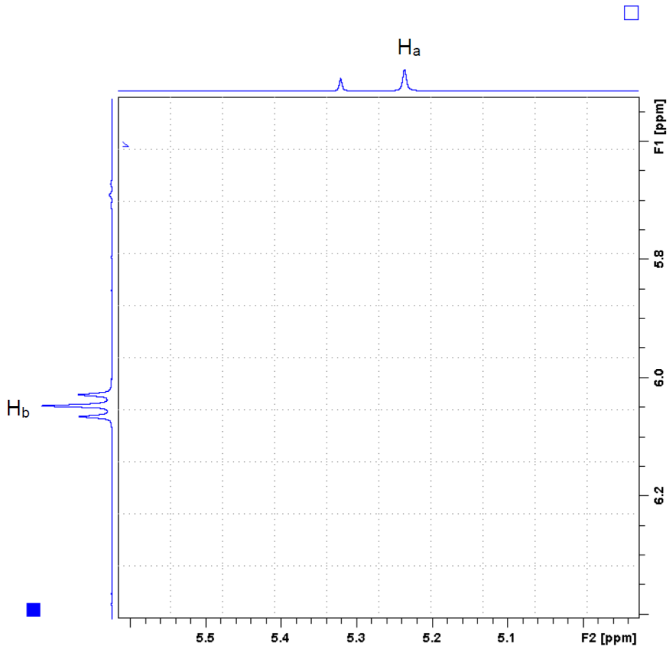

VI. The DEPT 135 (Distortionless Enhancement by Polarization Transfer) experiment:

The DEPT 135 experiments were carried out on a Bruker 500 MHz spectrometer in $CDCl_3$ at 23 °C.

DEPT 135 experiment for product (*E*)-2b:

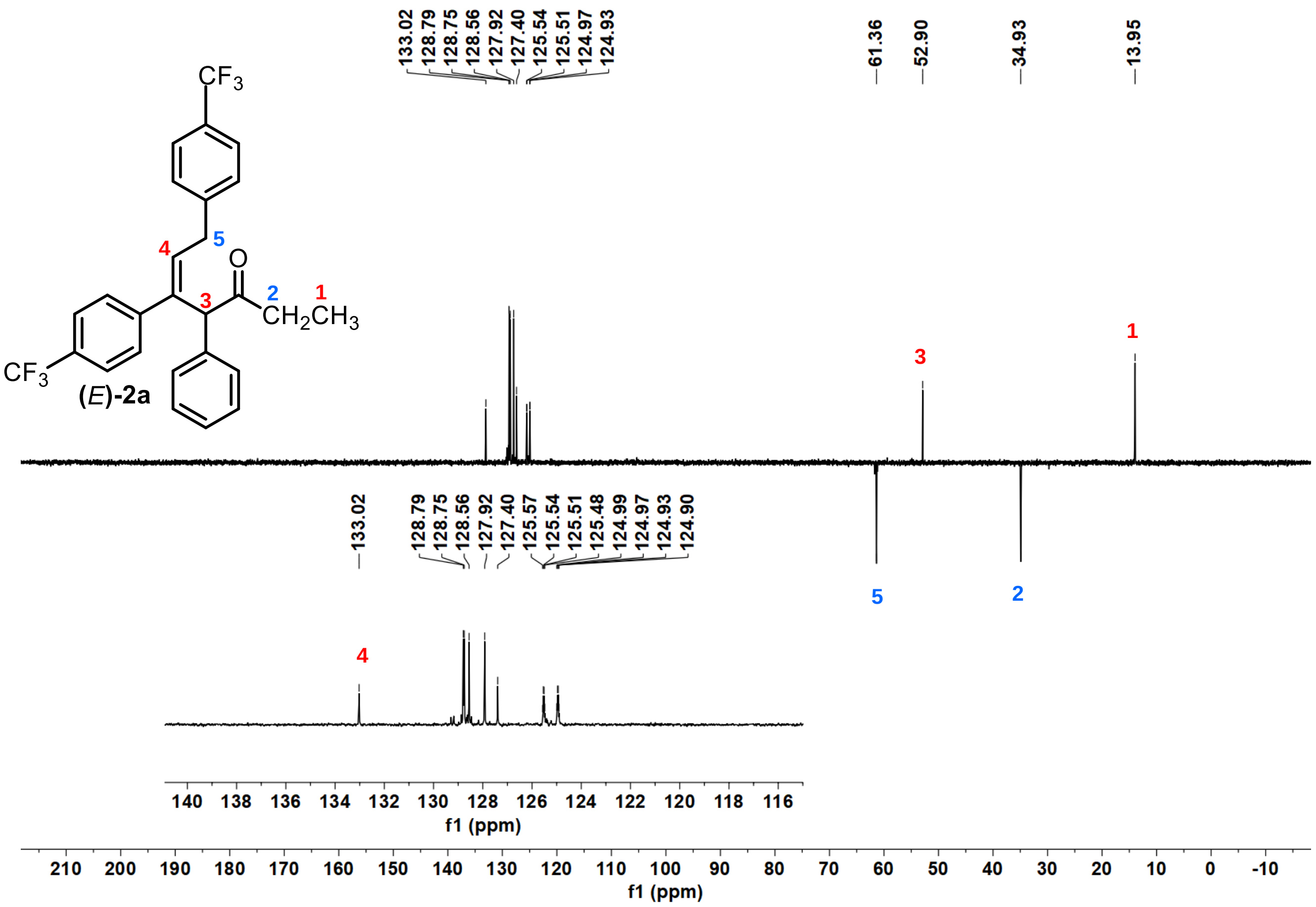

VII. Optimization studies. [a]

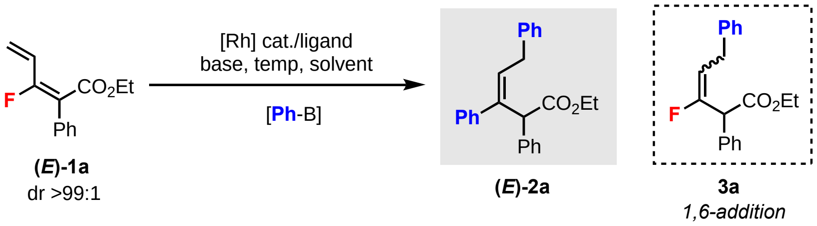

entry	[Ph-B] (equiv)	[Rh] (x mol %)	Ligand (x mol %)	Base (x equiv)	Solvent	Temp (°C)	**2a** (%)[b]
1	$PhB(OH)_2$ (2.5)	$[Rh(COD)(OH)]_2$ (1.5)	BINAP (4.5)	K_2CO_3(1)	Dioxane: H_2O=100:1	75	**94 (90)**[c]
2	$PhB(OH)_2$ (2.5)	none	BINAP (4.5)	K_2CO_3(1)	Dioxane: H_2O=100:1	75	0
3	$PhB(OH)_2$ (2.5)	$[Rh(COD)(OH)]_2$ (1.5)	none	K_2CO_3(1)	Dioxane: H_2O=100:1	75	23
4	$PhB(OH)_2$ (2.5)	$[Rh(COD)(Cl)]_2$ (1.5)	BINAP (4.5)	K_2CO_3(1)	Dioxane: H_2O=100:1	75	15
5	$PhB(OH)_2$ (2.5)	$Rh(CO)_2acac$ (3.0)	BINAP (4.5)	K_2CO_3(1)	Dioxane: H_2O=100:1	75	77
6	$PhB(OH)_2$ (2.5)	$Rh(COD)_2BF_4$ (3.0)	BINAP (4.5)	K_2CO_3(1)	Dioxane: H_2O=100:1	75	56
7	$PhB(OH)_2$ (2.5)	$Rh(COD)_2OTf$ (3.0)	BINAP (4.5)	K_2CO_3(1)	Dioxane: H_2O=100:1	75	44
8	$PhBF_3K$(2.5)	$[Rh(COD)(OH)]_2$ (1.5)	BINAP (4.5)	K_2CO_3(1)	Dioxane: H_2O=100:1	75	34
9	PhBpin(2.5)	$[Rh(COD)(OH)]_2$ (1.5)	BINAP (4.5)	K_2CO_3(1)	Dioxane: H_2O=100:1	75	<5
10	$PhB(OH)_2$ (1.2)	$[Rh(COD)(OH)]_2$ (1.5)	BINAP (4.5)	K_2CO_3(1)	Dioxane: H_2O=100:1	75	51
11	$PhB(OH)_2$ (5.0)	$[Rh(COD)(OH)]_2$ (1.5)	BINAP (4.5)	K_2CO_3(1)	Dioxane: H_2O=100:1	75	91
12	$PhB(OH)_2$ (2.5)	$[Rh(COD)(OH)]_2$ (1.5)	dppm (4.5)	K_2CO_3(1)	Dioxane: H_2O=100:1	75	37
13	$PhB(OH)_2$ (2.5)	$[Rh(COD)(OH)]_2$ (1.5)	dppe (4.5)	K_2CO_3(1)	Dioxane: H_2O=100:1	75	37
14	$PhB(OH)_2$ (2.5)	$[Rh(COD)(OH)]_2$ (1.5)	dppp (4.5)	K_2CO_3(1)	Dioxane: H_2O=100:1	75	56
15	$PhB(OH)_2$ (2.5)	$[Rh(COD)(OH)]_2$ (1.5)	dppb (4.5)	K_2CO_3(1)	Dioxane: H_2O=100:1	75	29
16	$PhB(OH)_2$ (2.5)	$[Rh(COD)(OH)]_2$ (1.5)	dppf (4.5)	K_2CO_3(1)	Dioxane: H_2O=100:1	75	82
17	$PhB(OH)_2$ (2.5)	$[Rh(COD)(OH)]_2$ (1.5)	BINAP (4.5)	K_2CO_3(1)	DMF: H_2O=100:1	75	23
18	$PhB(OH)_2$ (2.5)	$[Rh(COD)(OH)]_2$ (1.5)	BINAP (4.5)	K_2CO_3(1)	Toluene: H_2O=100:1	75	54
19	$PhB(OH)_2$ (2.5)	$[Rh(COD)(OH)]_2$ (1.5)	BINAP (4.5)	none	Toluene: H_2O=100:1	75	23
20	$PhB(OH)_2$ (2.5)	$[Rh(COD)(OH)]_2$ (1.5)	none	none	Toluene: H_2O=100:1	75	20
21	$PhB(OH)_2$ (2.5)	$[Rh(COD)(OH)]_2$ (1.5)	BINAP (4.5)	K_2CO_3(1)	Dioxane: H_2O=100:1	25	0[d]
22	$PhB(OH)_2$ (2.5)	$[Rh(COD)(OH)]_2$ (1.5)	none	K_2CO_3(1)	Dioxane: H_2O=100:1	25	0
23	$PhB(OH)_2$ (2.5)	$[Rh(COD)(OH)]_2$ (1.5)	none	K_2CO_3(1)	Dioxane: H_2O=100:1	100	33
24	$PhB(OH)_2$ (2.5)	$[Rh(COD)(OH)]_2$ (1.5)	BINAP (4.5)	none	Dioxane: H_2O=100:1	25	0

[a]Unless specified otherwise, reactions were carried out using (*E*)-**1a** (0.1 mmol) under argon. [b]Yield was determined by GC-MS. [c]Isolated yield. Diastereomeric ratio of **2a** (dr >99:1) was determined by GC-MS and ^{1}H NMR analyses. [d]Isolated **3a** in 93% yield as a mixture of *E*/*Z* isomers.

VIII. Screening of chiral ligands.

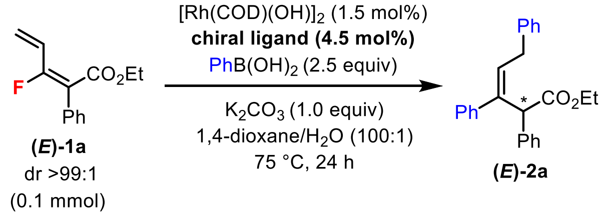

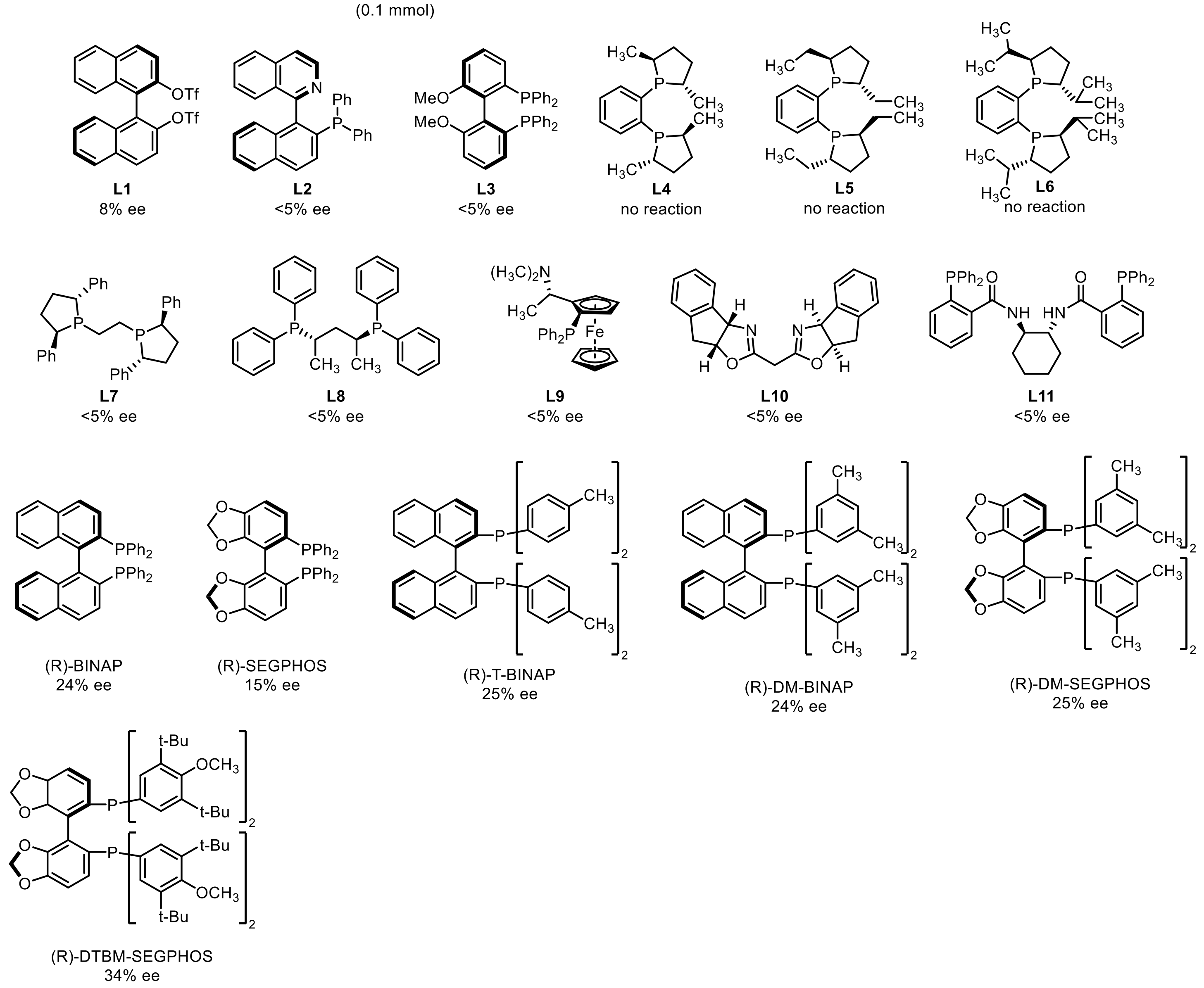

IX. Modular synthesis.

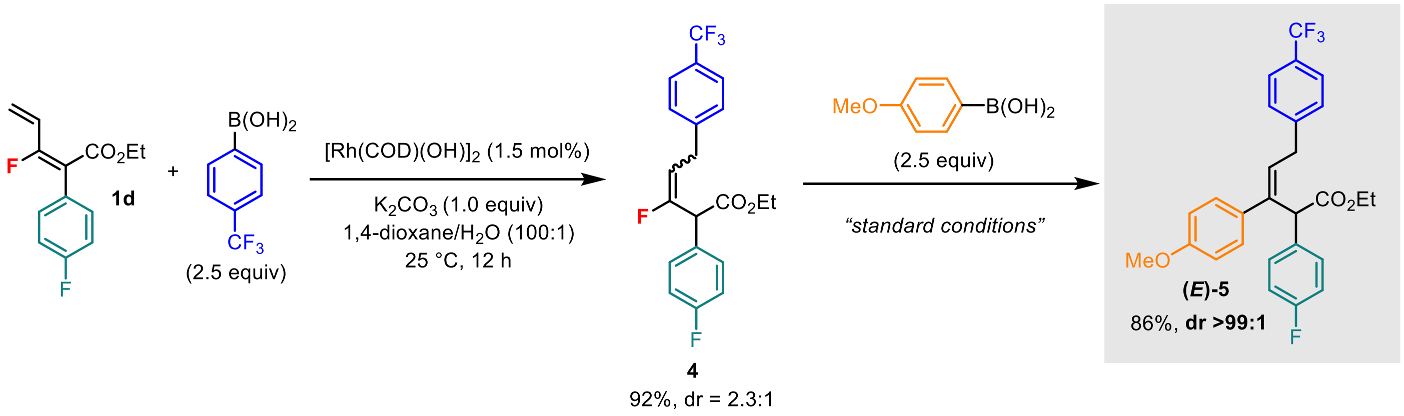

To an oven-dried glass tube equipped with a stir bar was added K_2CO_3 (41.4 mg, 0.3 mmol) and $[Rh(COD)(OH)]_2$ (2.1 mg, 0.0045 mmol). The tube was sealed with a septum, evacuated and refilled with argon three times. Then 1.4-dioxane (0.9 mL) was added and (*E*)-**1d** (71.4 mg, 0.3 mmol) and 4-trifluoromethylphenylboronic acid (142.5 mg, 0.75 mmol) were added together as a solution in 1,4-dioxane (1.5 mL) and H_2O (24 μL). The resulting mixture was stirred at 25 °C for 12 h. The crude sample was analyzed by ^{19}F NMR using benzotrifluoride (12 μL, 0.1 mmol) as internal standard and the *E/Z* ratio was determined. The crude mixture was extracted with CH_2Cl_2 (3 × 10 mL), the combined organic layers were washed with H_2O (2 × 10 mL) then brine (2 × 10 mL), dried over $MgSO_4$ and concentrated *in vacuo*. The residue was purified by flash column chromatography on silica gel (DCM : hexane = 1 : 3) to afford **4** as a colorless oil (106.1 mg, 92%, dr =2.3:1).

To an oven-dried glass tube equipped with a stir bar was added K_2CO_3 (27.6 mg, 0.2 mmol), BINAP (5.6 mg, 0.009 mmol) and $[Rh(COD)(OH)]_2$ (1.4 mg, 0.003 mmol). The tube was sealed with a septum, evacuated and refilled with argon three times. Then 1.4-dioxane (0.6 mL) was added and the mixture was stirred for 10 min in a 50 °C oil bath, the solution turned reddish. **4** (76.8 mg, 0.2 mmol) and 4-methoxyphenylboronic acid (76.0 mg, 0.5 mmol) were added together as a solution in 1,4-dioxane (1.0 mL) and H_2O (16 μL). The resulting mixture was heated at 75 °C with stirring in an oil bath for 24 h. After cooling to room temperature, the crude sample was analyzed by GC-MS and the *E/Z* ratio (>99:1) was determined. The crude mixture was extracted with CH_2Cl_2 (3 × 10 mL), the combined organic layers were washed with H_2O (2 × 10 mL) then brine (2 × 10 mL), dried over $MgSO_4$ and concentrated *in vacuo*. The residue was purified by flash column chromatography on silica gel (DCM : hexane = 1 : 2) to afford products (*E*)-**5** as a colorless oil (81.2 mg, 86% yield, dr >99:1).

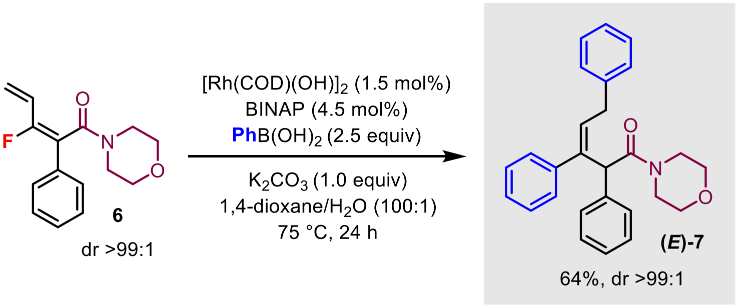

To an oven-dried glass tube equipped with a stir bar was added K_2CO_3 (27.6 mg, 0.2 mmol), BINAP (5.6 mg, 0.009 mmol) and $[Rh(COD)(OH)]_2$ (1.4 mg, 0.003 mmol). The tube was sealed with a septum, evacuated and refilled with argon three times. Then 1.4-dioxane (0.6 mL) was added and the mixture was stirred for 10 min in a 50 °C oil bath, the solution turned reddish. (*E*)-**6** (52.2 mg, 0.2 mmol) and phenylboronic acid (61.0 mg, 0.5 mmol) were added together as a solution in 1,4-dioxane (1.0 mL) and H_2O (16 μL). The resulting mixture was heated at 75 °C with stirring in an oil bath for 24 h. After cooling to room temperature, the crude sample was analyzed by GC-MS and the *E/Z* ratio (>99:1) was determined. The crude mixture was extracted with CH_2Cl_2 (3 × 10 mL), the combined organic layers were washed with H_2O (2 × 10 mL) then brine (2 × 10 mL), dried over $MgSO_4$ and concentrated *in vacuo*. The residue was purified by flash column chromatography on silica gel (DCM : hexane = 1 : 3) to afford (*E*)-**7** as a colorless oil (50.8 mg, 64%, dr >99:1).

X. Further experiments.

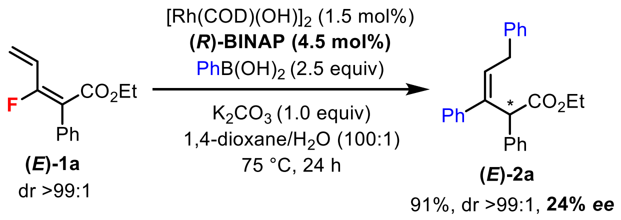

To an oven-dried glass tube equipped with a stir bar was added K_2CO_3 (13.8 mg, 0.1 mmol), (R)-BINAP (2.8 mg, 0.0045 mmol) and $[Rh(COD)(OH)]_2$ (0.7 mg, 0.0015 mmol). The tube was sealed with a septum, evacuated and refilled with argon three times. Then 1.4-dioxane (0.3 mL) was added and the mixture was stirred for 10 min in a 50 °C oil bath, the solution turned reddish. (*E*)-**1a** (22.1 mg, 0.1 mmol) and phenylboronic acid (31.9 mg, 0.25 mmol) were added together as a solution in 1,4-dioxane (0.5 mL) and H_2O (8 µL). The resulting mixture was heated at 75 °C with stirring in an oil bath for 24 h. After cooling to room temperature, the crude sample was analyzed by GC-MS and the *E/Z* ratio (>99:1) was determined. The crude mixture was extracted with CH_2Cl_2 (3 × 10 mL), the combined organic layers were washed with H_2O (2 × 10 mL) then brine (2 × 10 mL), dried over $MgSO_4$ and concentrated *in vacuo*. The residue was purified by flash column chromatography on silica gel (DCM : hexane = 1 : 5) to afford products (*E*)-**2a** as a colorless oil (32.4 mg, 91% yield, dr >99:1, 24% ee). The enantiomeric excess was measured by HPLC: Chiralcel IG column, flow rate 0.5 mL/min, hexane/isopropanol= 99.5/0.5, 23 °C, 254 nm, t_{R1}= 13.3 min (minor), t_{R2}= 17.1 min (major).

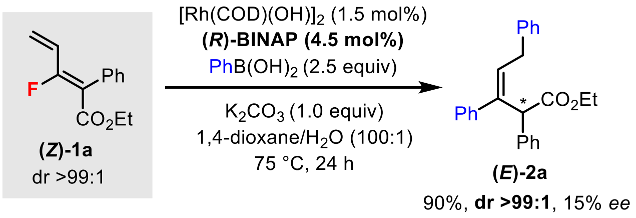

To an oven-dried glass tube equipped with a stir bar was added K_2CO_3 (13.8 mg, 0.1 mmol), (R)-BINAP (2.8 mg, 0.0045 mmol) and $[Rh(COD)(OH)]_2$ (0.7 mg, 0.0015 mmol). The tube was sealed with a septum, evacuated and refilled with argon three times. Then 1.4-dioxane (0.3 mL) was added and the mixture was stirred for 10 min in a 50 °C oil bath, the solution turned reddish. (*E*)-**1a** (22.1 mg, 0.1 mmol) and phenylboronic acid (31.9 mg, 0.25 mmol) were added together as a solution in 1,4-dioxane (0.5 mL) and H_2O (8 µL). The resulting mixture was heated at 75 °C with stirring in an oil bath for 24 h. After cooling to room temperature, the crude sample was analyzed by GC-MS and the *E/Z* ratio (>99:1) was determined. The crude mixture was extracted with CH_2Cl_2 (3 × 10 mL), the combined organic layers were washed with H_2O (2 × 10 mL) then brine (2 × 10 mL), dried over $MgSO_4$ and concentrated *in vacuo*. The residue was purified by flash column chromatography on silica gel (DCM : hexane = 1 : 5) to afford products (*E*)-**2a** as a colorless oil (32.0 mg, 90% yield, dr >99:1, 15% ee). The enantiomeric excess was measured by HPLC: Chiralcel IG column, flow rate 0.5 mL/min, hexane/isopropanol= 99.5/0.5, 23 °C, 254 nm, t_{R1}= 15.2 min (minor), t_{R2}= 20.6 min (major).

Isomerization studies.

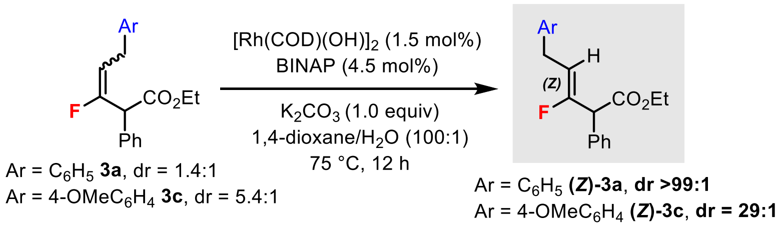

To an oven-dried glass tube equipped with a stir bar was added K_2CO_3 (27.6 mg, 0.2 mmol), BINAP (5.6 mg, 0.009 mmol) and $[Rh(COD)(OH)]_2$ (1.4 mg, 0.003 mmol). The tube was sealed with a septum, evacuated and refilled with argon three times. Then 1.4-dioxane (0.6 mL) was added and the mixture was stirred for 10 min in a 50 °C oil bath, the solution turned reddish. **3a** (59.6 mg, 0.2 mmol) was added as a solution in 1,4-dioxane (1.0 mL) and H_2O (16 µL). The resulting mixture was heated at

75 °C with stirring in an oil bath for 12 h. After cooling to room temperature, The crude sample was analyzed by ^{19}F NMR using benzotrifluoride (12 μL, 0.1 mmol) as internal standard and the *E/Z* ratio was determined. The crude mixture was extracted with CH_2Cl_2 (3 × 10 mL), the combined organic layers were washed with H_2O (2 × 10 mL) then brine (2 × 10 mL), dried over $MgSO_4$ and concentrated *in vacuo*. The residue was purified by flash column chromatography on silica gel (DCM : hexane = 1 : 5) to afford products (*E*)-**3a** as a colorless oil (53.0 mg, 89% yield, dr >99:1).

To an oven-dried glass tube equipped with a stir bar was added K_2CO_3 (27.6 mg, 0.2 mmol), BINAP (5.6 mg, 0.009 mmol) and $[Rh(COD)(OH)]_2$ (1.4 mg, 0.003 mmol). The tube was sealed with a septum, evacuated and refilled with argon three times. Then 1.4-dioxane (0.6 mL) was added and the mixture was stirred for 10 min in a 50 °C oil bath, the solution turned reddish. **3c** (65.6 mg, 0.2 mmol) was added as a solution in 1,4-dioxane (1.0 mL) and H_2O (16 μL). The resulting mixture was heated at 75 °C with stirring in an oil bath for 12 h. After cooling to room temperature, The crude sample was analyzed by ^{19}F NMR using benzotrifluoride (12 μL, 0.1 mmol) as internal standard and the *E/Z* ratio was determined. The crude mixture was extracted with CH_2Cl_2 (3 × 10 mL), the combined organic layers were washed with H_2O (2 × 10 mL) then brine (2 × 10 mL), dried over $MgSO_4$ and concentrated *in vacuo*. The residue was purified by flash column chromatography on silica gel (DCM : hexane = 1 : 3) to afford products (*E*)-**2c** as a colorless oil (55.8 mg, 85% yield, dr =29:1).

XI. References.

1. Li, M.; Wang, Y.; Tsui, G. C. Palladium-Catalyzed Stereoselective C−F Bond Vinylation and Allylation of Tetrasubstituted *gem*-Difluoroalkenes via Stille Coupling: Synthesis of Monofluorinated 1,3- and 1,4-Dienes. *Org. Lett.* **2021**, *23*, 8072-8076.
2. Ma, Q.; Wang, Y.; Tsui, G. C. Stereoselective Palladium-Catalyzed C-F Bond Alkynylation of Tetrasubstituted *gem*-Difluoroalkenes. *Angew. Chem., Int. Ed.* **2020**, *59*, 11293-11297.
3. Yang, L.; Ji, W.-W.; Lin, E.; Li, J.-L.; Fan, W.-X.; Li, Q.; Wang, H. Synthesis of Alkylated Monofluoroalkenes via Fe-Catalyzed Defluorinative Cross Coupling of Donor Alkenes with gem-Difluoroalkenes. *Org. Lett.* **2018**, *20*, 1924-1927.
4. Wang, Y.; Tsui, G. C. Stereoselective Palladium-Catalyzed C–F Bond Alkenylation of Tetrasubstituted *gem*-Difluoroalkenes via Mizoroki–Heck Reaction. *Org. Lett.* **2023**, *25*, 6217-6221.

XII. Characterization Data.

ethyl (*Z*)-3-fluoro-2-phenylpenta-2,4-dienoate (1a)

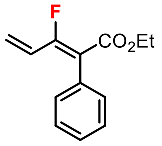

Following the general procedure (II), reaction was run using *gem*-difluoroalkene (42.4 mg, 0.2 mmol), tributyl(vinyl)tin (95.1 mg, 0.3 mmol), Pd(dba)$_2$ (5.8 mg, 0.01 mmol) and dppe (4.0 mg, 0.01 mmol). in 1.0 mL toluene at 50 °C for 12 h. The product was purified by flash column chromatography on silica gel (DCM : hexane = 1: 10) and obtained as a colorless oil (22.4 mg, 51% yield), R_f = 0.31 (DCM : hexane = 1: 5). **^{1}H NMR** (500 MHz, CDCl$_3$): δ (ppm) 7.43 - 7.32 (m, 3H), 7.28 (dd, J = 7.7, 1.8 Hz, 2H), 6.16 (ddd, J = 25.6, 17.2, 11.1 Hz, 1H), 5.90 (d, J = 17.2 Hz, 1H), 5.42 (dt, J = 11.1, 1.6 Hz, 1H), 4.26 (q, J = 7.2 Hz, 2H), 1.28 (t, J = 7.1 Hz, 3H). **^{13}C NMR** (126 MHz, CDCl$_3$): δ (ppm) 165.6, 159.8 (d, J = 270.3 Hz), 132.7 (d, J = 6.2 Hz), 130.4 (d, J = 3.0 Hz), 128.4, 128.2, 121.4 (d, J = 6.6 Hz), 115.9 (d, J = 13.1 Hz), 61.2, 14.2. **^{19}F NMR** (471MHz, CDCl$_3$): δ (ppm) -110.29 (d, J = 25.5 Hz). **HRMS** m/z (ESI): calcd. for $C_{13}H_{13}FO_2Na$ [M+Na]$^+$: 243.0790; found: 243.0793.

(*E*)-3-fluoro-1-morpholino-2-phenylpenta-2,4-dien-1-one (6)

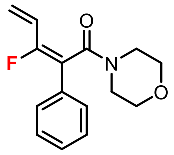

Following the general procedure (I), reaction was run using *gem*-difluoroalkene (50.6 mg, 0.2 mmol), tributyl(vinyl)tin (95.1 mg, 0.3 mmol), Pd(dba)$_2$ (5.8 mg, 0.01 mmol) and dppe (4.0 mg, 0.01 mmol). in 1.0 mL toluene at 25 °C for 12 h. The product was purified by flash column chromatography on silica gel (DCM : hexane = 1: 5) and obtained as a colorless oil (39.7 mg, 76% yield), R_f = 0.36 (DCM : hexane = 1: 3). **^{1}H NMR** (500 MHz, CDCl$_3$): δ (ppm) 7.54 - 7.44 (m, 2H), 7.42 - 7.34 (m, 2H), 7.34 - 7.26 (m, 1H), 6.36 (ddd, J = 26.7, 17.0, 11.1 Hz, 1H), 5.79 (dd, J = 17.0, 0.9 Hz, 1H), 5.43 (d, J = 11.1 Hz, 1H), 3.81 - 3.66 (m, 4H), 3.40 (ddd, J = 42.0, 5.9, 4.3 Hz, 4H). **^{13}C NMR** (126 MHz, CDCl$_3$): δ (ppm) 166.3 (d, J = 12.9 Hz), 154.6 (d, J = 263.7 Hz), 131.6 (d, J = 1.8 Hz), 128.9, 128.5, 128.3 (d, J = 6.5 Hz), 126.5 (d, J = 24.6 Hz), 118.9 (d, J = 5.8 Hz), 117.7 (d, J = 12.3 Hz), 66.8 (d, J = 4.4 Hz), 44.7 (d, J = 622.2 Hz), 27.5 (d, J = 125.3 Hz), 15.7 (d, J = 493.2 Hz). **^{19}F NMR** (471 MHz, CDCl$_3$): δ (ppm) -122.97 (d, J = 26.7 Hz). **HRMS** m/z (ESI): calcd. for $C_{15}H_{16}FNO_2Na$ [M+Na]$^+$: 284.1063; found: 284.1068.

ethyl (*E*)-2,3,5-triphenylpent-3-enoate (2a)

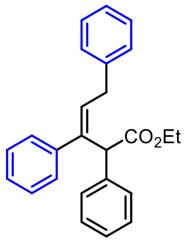

Following the general procedure (III), reaction was run using monofluorodiene **1a** (220.2 mg, 1.0 mmol), phenylboronic acid (305.1 mg, 2.5 mmol), [Rh(COD)(OH)]$_2$ (6.8 mg, 0.015 mmol), BINAP (28.0 mg, 0.045 mmol) and K_2CO_3 (138.2 mg, 1.0 mmol) in 8.0 mL 1,4-dioxane and 80 μL H_2O for 24 h. The product was purified by flash column chromatography on silica gel (DCM : hexane = 1: 5) and obtained as a colorless oil (306.1 mg, 86% yield), R_f = 0.36 (DCM : hexane = 1: 3). **^{1}H NMR** (500 MHz, CDCl$_3$): δ (ppm) 7.71 - 6.95 (m, 15H), 6.14 (t, J = 7.4 Hz, 1H), 5.25 (s, 1H), 4.12 (q, J = 7.1 Hz, 2H), 3.54 (h, J = 8.1, 7.3 Hz, 2H), 1.12 (t, J = 7.1 Hz, 3H). **^{13}C NMR** (126 MHz, CDCl$_3$): δ (ppm) 172.5, 142.5, 140.1, 138.0, 137.0, 132.8, 129.1, 128.6, 128.4, 128.1, 127.6, 127.1, 127.1, 126.3, 61.2, 53.2, 35.2, 14.1. **HRMS** m/z (ESI): calcd. for $C_{25}H_{24}O_2Na$ [M+Na]$^+$: 379.1670; found: 379.1669.

ethyl (*E*)-2-phenyl-3,5-bis(4-(trifluoromethyl)phenyl)pent-3-enoate (2b)

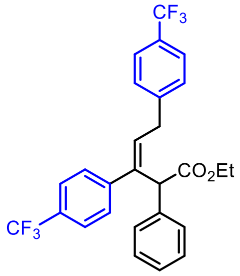

Following the general procedure (III), reaction was run using monofluorodiene **1a** (44.1 mg, 0.2 mmol), 4-trifluoromethylphenylboronic acid (95.0 mg, 0.5 mmol), $[Rh(COD)(OH)]_2$ (1.4 mg, 0.003 mmol), BINAP (5.6 mg, 0.009 mmol) and K_2CO_3 (27.6 mg, 0.2 mmol) in 1.6 mL 1,4-dioxane and 16 μL H_2O for 24 h. The product was purified by flash column chromatography on silica gel (DCM : hexane = 1: 5) and obtained as a colorless oil (74.8 mg, 76% yield), R_f = 0.30 (DCM : hexane = 1: 3). **^{1}H NMR** (500 MHz, $CDCl_3$): δ (ppm) 7.55 (dd, J = 31.7, 8.1 Hz, 4H), 7.34 (dq, J = 32.2, 8.0 Hz, 9H), 6.13 (t, J = 7.4 Hz, 1H), 5.26 (s, 1H), 4.16 (q, J = 7.1 Hz, 2H), 3.75 - 3.41 (m, 2H), 1.15 (t, J = 7.1 Hz, 3H). **^{13}C NMR** (126 MHz, $CDCl_3$): δ (ppm) 171.9, 145.8, 143.7, 138.2, 136.4, 133.1, 129.6 - 129.2 (m), 129.5 - 128.2 (m)128.9, 128.9, 128.7, 128.0, 127.5, 125.6 (q, J = 3.7 Hz), 125.1 (q, J = 3.8 Hz), 61.5, 53.0, 35.0, 14.1. **^{19}F NMR** (471 MHz, $CDCl_3$): δ (ppm) -62.38 (s, 3F), -62.50 (s, 3F). **HRMS** m/z (ESI): calcd. for $C_{27}H_{22}F_6O_2Na$ $[M+Na]^+$: 515.1421; found: 515.1416.

ethyl (*E*)-3,5-bis(4-methoxyphenyl)-2-phenylpent-3-enoate (2c)

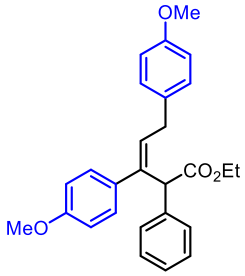

Following the general procedure (III), reaction was run using monofluorodiene **1a** (44.1 mg, 0.2 mmol), 4-methoxyphenylboronic acid (76.0 mg, 0.5 mmol), $[Rh(COD)(OH)]_2$ (1.4 mg, 0.003 mmol), BINAP (5.6 mg, 0.009 mmol) and K_2CO_3 (27.6 mg, 0.2 mmol) in 1.6 mL 1,4-dioxane and 16 μL H_2O for 24 h. The product was purified by flash column chromatography on silica gel (DCM : hexane = 1: 3) and obtained as a colorless oil (75.7 mg, 91% yield), R_f = 0.28 (DCM : hexane = 1: 1). **^{1}H NMR** (500 MHz, $CDCl_3$): δ (ppm) 7.37 - 7.25 (m, 5H), 7.22 - 7.16 (m, 2H), 7.13 - 7.07 (m, 2H), 6.83 (d, J = 8.6 Hz, 2H), 6.76 (d, J = 8.8 Hz, 2H), 6.02 (t, J = 7.4 Hz, 1H), 5.21 (s, 1H), 4.11 (q, J = 7.1 Hz, 2H), 3.79 (s, 3H), 3.77 (s, 3H), 3.61 - 3.28 (m, 2H), 1.12 (t, J = 7.1 Hz, 3H). **^{13}C NMR** (126 MHz, $CDCl_3$): δ (ppm) 172.6, 158.7, 158.1, 137.1, 137.0, 134.9, 132.3, 132.1, 129.5, 129.1, 128.8, 128.4, 127.1, 114.0, 113.4, 61.1, 55.4, 55.3, 53.0, 34.3, 14.1. **HRMS** m/z (ESI): calcd. for $C_{27}H_{28}O_4Na$ $[M+Na]^+$: 439.1883; found: 439.1880.

ethyl (*E*)-3,5-bis(4-fluorophenyl)-2-phenylpent-3-enoate (2d)

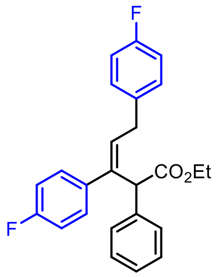

Following the general procedure (III), reaction was run using monofluorodiene **1a** (44.1 mg, 0.2 mmol), 4-fluorobenzeneboronic acid (70.0 mg, 0.5 mmol), $[Rh(COD)(OH)]_2$ (1.4 mg, 0.003 mmol), BINAP (5.6 mg, 0.009 mmol) and K_2CO_3 (27.6 mg, 0.2 mmol) in 1.6 mL 1,4-dioxane and 16 μL H_2O for 24 h. The product was purified by flash column chromatography on silica gel (DCM : hexane = 1: 6) and obtained as a colorless oil (67.4 mg, 86% yield), R_f = 0.31 (DCM : hexane = 1: 5). **^{1}H NMR** (500 MHz, $CDCl_3$): δ (ppm) 7.39 - 7.24 (m, 5H), 7.23 - 7.17 (m, 2H), 7.15 - 7.09 (m, 2H), 7.01 - 6.85 (m, 4H), 5.99 (t, J = 7.4 Hz, 1H), 5.19 (s, 1H), 4.11 (q, J = 7.1 Hz, 2H), 3.71 -3.28 (m, 2H), 1.12 (t, J = 7.1 Hz, 3H). **^{13}C NMR**

(126 MHz, $CDCl_3$): δ (ppm) 172.2, 163.1 (d, J = 53.3 Hz), 160.7 (d, J = 51.7 Hz), 138.2 (d, J = 3.3 Hz), 137.4, 136.7, 135.6 (d, J = 3.2 Hz), 132.6, 129.9 (d, J = 7.8 Hz), 129.4 (d, J = 7.9 Hz), 129.0, 128.5, 127.3, 115.4 (d, J = 21.2 Hz), 114.8 (d, J = 21.3 Hz), 61.3, 53.1, 34.3, 14.1. **^{19}F NMR** (471 MHz, $CDCl_3$): δ (ppm) -115.75 - -115.80 (m, 2F). **HRMS** m/z (ESI): calcd. for $C_{25}H_{22}F_2O_2Na$ $[M+Na]^+$: 415.1482; found: 415.1480.

ethyl (*E*)-3,5-bis(4-chlorophenyl)-2-phenylpent-3-enoate (2e)

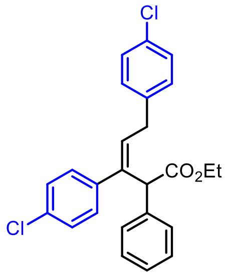

Following the general procedure (III), reaction was run using monofluorodiene **1a** (44.1 mg, 0.2 mmol), 4-chlorophenylboronic acid (78.2 mg, 0.5 mmol), $[Rh(COD)(OH)]_2$ (1.4 mg, 0.003 mmol), BINAP (5.6 mg, 0.009 mmol) and K_2CO_3 (27.6 mg, 0.2 mmol) in 1.6 mL 1,4-dioxane and 16 μL H_2O for 24 h. The product was purified by flash column chromatography on silica gel (DCM : hexane = 1: 5) and obtained as a colorless oil (44.1 mg, 52% yield), R_f = 0.33 (DCM : hexane = 1: 3). **^{1}H NMR** (500 MHz, $CDCl_3$): δ (ppm) 7.43 - 7.24 (m, 8H), 7.22 - 7.16 (m, 3H), 7.11 (d, J = 8.0 Hz, 2H), 6.03 (t, J = 7.4 Hz, 1H), 5.20 (s, 1H), 4.13 (q, J = 7.1 Hz, 2H), 3.52 (h, J = 8.9, 8.2 Hz, 2H), 1.15 (t, J = 7.1 Hz, 3H). **^{13}C NMR** (126 MHz, $CDCl_3$): δ (ppm) 172.1, 140.6, 138.3, 137.6, 136.6, 133.1, 132.6, 132.2, 129.9, 129.1, 128.9, 128.8, 128.5, 128.2, 127.3, 61.3, 52.9, 34.5, 14.1. **HRMS** m/z (ESI): calcd. for $C_{25}H_{22}Cl_2O_2Na$ $[M+Na]^+$: 447.0892; found: 447.0889.

ethyl (*E*)-3,5-bis(4-bromophenyl)-2-phenylpent-3-enoate (2f)

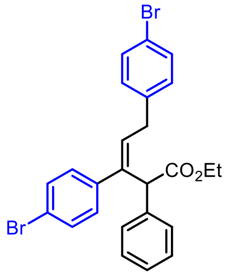

Following the general procedure (III), reaction was run using monofluorodiene **1a** (44.1 mg, 0.2 mmol), 4-bromophenylboronic acid (100.4 mg, 0.5 mmol), $[Rh(COD)(OH)]_2$ (1.4 mg, 0.003 mmol), BINAP (5.6 mg, 0.009 mmol) and K_2CO_3 (27.6 mg, 0.2 mmol) in 1.6 mL 1,4-dioxane and 16 μL H_2O for 24 h. The product was purified by flash column chromatography on silica gel (DCM : hexane = 1: 5) and obtained as a yellow oil (47.1 mg, 46% yield), R_f = 0.32 (DCM : hexane = 1: 3). **^{1}H NMR** (500 MHz, $CDCl_3$): δ (ppm) 7.58 - 7.24 (m, 10H), 7.21 - 6.96 (m, 3H), 6.02 (t, J = 7.4 Hz, 1H), 5.19 (s, 1H), 4.13 (q, J = 7.1 Hz, 2H), 3.49 (h, J = 8.8, 8.2 Hz, 2H), 1.15 (t, J = 7.1 Hz, 3H). **^{13}C NMR** (126 MHz, $CDCl_3$): δ (ppm) 172.1, 141.1, 138.8, 137.7, 136.6, 132.5, 131.7, 131.2, 130.3, 129.4, 128.9, 128.6, 127.4, 121.3, 120.2, 61.4, 52.9, 34.6, 14.1. **HRMS** m/z (ESI): calcd. for $C_{25}H_{22}Br_2O_2Na$ $[M+Na]^+$: 536.9865; found: 536.9860.

ethyl (*E*)-3,5-bis(4-cyanophenyl)-2-phenylpent-3-enoate (2g)

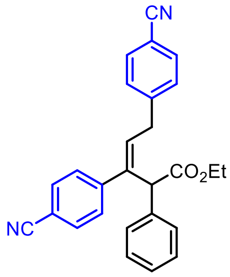

Following the general procedure (III), reaction was run using monofluorodiene **1a** (44.1 mg, 0.2 mmol), 4-cyanophenylboronic acid (73.5 mg, 0.5 mmol), $[Rh(COD)(OH)]_2$ (1.4 mg, 0.003 mmol), BINAP (5.6 mg, 0.009 mmol) and K_2CO_3 (27.6 mg, 0.2 mmol) in 1.6 mL 1,4-dioxane and 16 μL H_2O for 24 h. The product was purified by flash column chromatography on silica gel

(DCM : hexane = 1: 3) and obtained as a yellow oil (61.7 mg, 76% yield), R_f = 0.38 (DCM : hexane = 1: 1). **^{1}H NMR** (500 MHz, $CDCl_3$): δ (ppm) 7.56 (d, J = 7.9 Hz, 2H), 7.49 (d, J = 8.1 Hz, 2H), 7.34 - 7.07 (m, 9H), 6.05 (t, J = 7.5 Hz, 1H), 5.17 (s, 1H), 4.11 (qq, J = 6.5, 3.6 Hz, 2H), 3.72 - 3.43 (m, 2H), 1.11 (t, J = 7.1 Hz, 3H). **^{13}C NMR** (126 MHz, $CDCl_3$): δ (ppm) 171.7, 146.6, 144.9, 138.5, 136.1, 133.1, 132.6, 132.0, 129.3, 128.8, 128.7, 128.4, 127.6, 118.9, 118.9, 111.0, 110.6, 61.6, 52.8, 35.3, 14.1. **HRMS** m/z (ESI): calcd. for $C_{27}H_{22}N_2O_2Na$ $[M+Na]^+$: 429.1575; found: 429.1574.

ethyl (*E*)-3,5-di([1,1'-biphenyl]-4-yl)-2-phenylpent-3-enoate (2h)

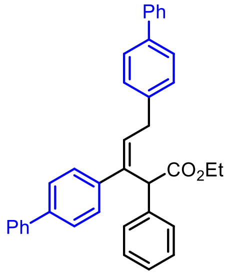

Following the general procedure (III), reaction was run using monofluorodiene **1a** (44.1 mg, 0.2 mmol), 4-biphenylboronic acid (99.0 mg, 0.5 mmol), $[Rh(COD)(OH)]_2$ (1.4 mg, 0.003 mmol), BINAP (5.6 mg, 0.009 mmol) and K_2CO_3 (27.6 mg, 0.2 mmol) in 1.6 mL 1,4-dioxane and 16 μL H_2O for 24 h. The product was purified by flash column chromatography on silica gel (DCM : hexane = 1: 3) and obtained as a colorless oil (85.4 mg, 84% yield), R_f = 0.34 (DCM : hexane = 1: 1). **^{1}H NMR** (500 MHz, $CDCl_3$): δ (ppm) 7.74 - 7.18 (m, 23H), 6.24 (t, J = 7.4 Hz, 1H), 5.31 (s, 1H), 4.16 (q, J = 7.0 Hz, 2H), 3.77 – 3.41 (m, 2H), 1.14 (t, J = 7.1 Hz, 3H). **^{13}C NMR** (126 MHz, $CDCl_3$): δ (ppm) 172.5, 141.5, 141.1, 140.8, 139.9, 139.3, 139.2, 137.7, 137.0, 132.7, 129.1, 129.1, 128.9, 128.5, 128.0, 127.4, 127.4, 127.3, 127.2, 127.2, 127.1, 126.8, 61.3, 53.1, 35.0, 14.1. **HRMS** m/z (ESI): calcd. for $C_{37}H_{32}O_2Na$ $[M+Na]^+$: 531.2301; found: 531.2295.

ethyl (*E*)-3,5-bis(4-nitrophenyl)-2-phenylpent-3-enoate (2i)

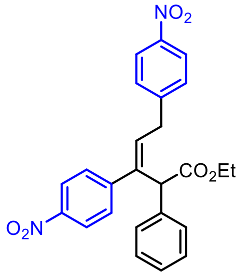

Following the general procedure (III), reaction was run using monofluorodiene **1a** (44.1 mg, 0.2 mmol), 4-nitrophenylboronic acid (83.5mg, 0.5 mmol), $[Rh(COD)(OH)]_2$ (1.4 mg, 0.003 mmol), BINAP (5.6 mg, 0.009 mmol) and K_2CO_3 (27.6 mg, 0.2 mmol) in 1.6 mL 1,4-dioxane and 16 μL H_2O for 24 h. The product was purified by flash column chromatography on silica gel (DCM : hexane = 1: 2) and obtained as a yellow oil (66.0 mg, 74% yield), R_f = 0.34 (DCM : hexane = 1: 1). **^{1}H NMR** (500 MHz, $CDCl_3$): δ (ppm) 8.27 - 7.98 (m, 4H), 7.60 - 7.07 (m, 9H), 5.81 (t, J = 7.6 Hz, 1H), 4.73 (s, 1H), 4.15 (dqt, J = 11.0, 7.1, 3.2 Hz, 2H), 3.35 (d, J = 7.6 Hz, 2H), 1.19 (t, J = 7.0 Hz, 3H). **^{13}C NMR** (126 MHz, $CDCl_3$): δ (ppm) 171.8, 140.4, 138.0, 137.3, 136.3, 132.8, 132.3, 131.9, 129.6, 128.8, 128.7, 128.5, 128.3, 127.9, 127.1, 61.1, 52.7, 34.2, 13.9. **HRMS** m/z (ESI): calcd. for $C_{25}H_{22}N_2O_6Na$ $[M+Na]^+$: 469.1374; found: 469.1370.

ethyl (*E*)-2-phenyl-3,5-di-p-tolylpent-3-enoate (2j)

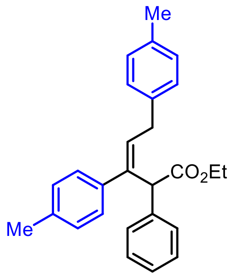

Following the general procedure (III), reaction was run using monofluorodiene **1a** (44.1 mg, 0.2 mmol), 4-tolylboronic acid (68.0 mg, 0.5 mmol), $[Rh(COD)(OH)]_2$ (1.4 mg, 0.003 mmol), BINAP (5.6 mg, 0.009 mmol) and K_2CO_3 (27.6 mg, 0.2 mmol)

in 1.6 mL 1,4-dioxane and 16 μL H_2O for 24 h. The product was purified by flash column chromatography on silica gel (DCM : hexane = 1: 10) and obtained as a colorless oil (68.4 mg, 89% yield), R_f = 0.30 (DCM : hexane = 1: 5). **^{1}H NMR** (500 MHz, $CDCl_3$): δ (ppm) 7.40 - 7.24 (m, 5H), 7.18 - 6.99 (m, 8H), 6.06 (t, J = 7.4 Hz, 1H), 5.20 (s, 1H), 4.10 (q, J = 7.0 Hz, 2H), 3.47 (qd, J = 16.3, 7.4 Hz, 2H), 2.32 (s, 3H), 2.29 (s, 3H), 1.11 (t, J = 7.1 Hz, 3H). **^{13}C NMR** (126 MHz, $CDCl_3$): δ (ppm) 172.6, 139.6, 137.5, 137.1, 136.7, 135.7, 132.5, 129.3, 129.1, 128.7, 128.5, 128.4, 127.4, 127.1, 61.1, 53.1, 34.8, 21.2, 21.1, 14.1. **HRMS** m/z (ESI): calcd. for $C_{27}H_{28}O_2Na$ $[M+Na]^+$: 407.1986; found: 407.1982.

ethyl (*E*)-3,5-bis(4-formylphenyl)-2-phenylpent-3-enoate (2k)

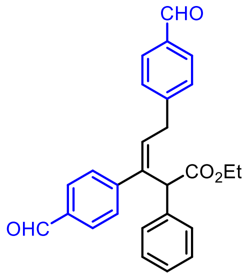

Following the general procedure (III), reaction was run using monofluorodiene **1a** (44.1 mg, 0.2 mmol), 4-formylphenylboronic acid (75.0 mg, 0.5 mmol), $[Rh(COD)(OH)]_2$ (1.4 mg, 0.003 mmol), BINAP (5.6 mg, 0.009 mmol) and K_2CO_3 (27.6 mg, 0.2 mmol) in 1.6 mL 1,4-dioxane and 16 μL H_2O for 24 h. The product was purified by flash column chromatography on silica gel (DCM : hexane = 1: 5) and obtained as a colorless oil (66.9 mg, 81% yield), R_f = 0.33 (DCM : hexane = 1: 3). **^{1}H NMR** (500 MHz, $CDCl_3$): δ (ppm) 9.98 (s, 1H), 9.96 (s, 1H), 7.81 (d, J = 8.2 Hz, 2H), 7.75 (d, J = 8.3 Hz, 2H), 7.42 (d, J = 8.4 Hz, 1H), 7.34 - 7.22 (m, 8H), 6.16 (t, J = 7.5 Hz, 1H), 5.24 (s, 1H), 4.12 (qd, J = 7.2, 1.3 Hz, 2H), 3.79 - 3.46 (m, 2H), 1.11 (t, J = 7.1 Hz, 3H). **^{13}C NMR** (126 MHz, $CDCl_3$): δ (ppm) 172.0, 167.1, 167.0, 146.9, 145.1, 138.3, 136.5, 133.1, 130.0, 129.5, 128.9, 128.6, 128.6, 127.6, 127.4, 61.4, 53.0, 52.2, 35.3, 14.1. **HRMS** m/z (ESI): calcd. for $C_{27}H_{24}O_4Na$ $[M+Na]^+$: 435.1571; found: 435.1567.

dimethyl 4,4'-(5-ethoxy-5-oxo-4-phenylpent-2-ene-1,3-diyl)(*E*)-dibenzoate (2l)

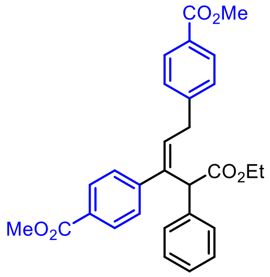

Following the general procedure (III), reaction was run using monofluorodiene **1a** (44.1 mg, 0.2 mmol), 4-(methoxycarbonyl)phenylboronic acid (90.0 mg, 0.5 mmol), $[Rh(COD)(OH)]_2$ (1.4 mg, 0.003 mmol), BINAP (5.6 mg, 0.009 mmol) and K_2CO_3 (27.6 mg, 0.2 mmol) in 1.6 mL 1,4-dioxane and 16 μL H_2O for 24 h. The product was purified by flash column chromatography on silica gel (DCM : hexane = 1: 3) and obtained as a colorless oil (80.3 mg, 85% yield), R_f = 0.38 (DCM : hexane = 1: 1). **^{1}H NMR** (500 MHz, $CDCl_3$): δ (ppm) 7.92 (dd, J = 26.0, 8.4 Hz, 4H), 7.52 - 7.14 (m, 9H), 6.12 (t, J = 7.4 Hz, 1H), 5.21 (s, 1H), 4.11 (q, J = 7.2 Hz, 2H), 3.90 (s, 3H), 3.88 (s, 3H), 3.65 - 3.47 (m, 2H), 1.10 (t, J = 7.1 Hz, 3H). **^{13}C NMR** (126 MHz, $CDCl_3$): δ (ppm) 172.3, 163.0, 161.0, 159.0, 144.2, 138.4, 134.4, 132.5, 132.5, 130.7, 130.7, 130.3, 128.9, 128.7, 125.5, 115.4, 115.2, 113.6, 61.4, 55.4, 52.3, 34.9, 14.1. **HRMS** m/z (ESI): calcd. for $C_{29}H_{28}O_6Na$ $[M+Na]^+$: 495.1782; found: 495.1778.

ethyl (*E*)-2-phenyl-3,5-di(thiophen-3-yl)pent-3-enoate (2m)

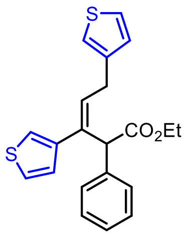

Following the general procedure (III), reaction was run using monofluorodiene **1a** (44.1 mg, 0.2 mmol), 3-thiopheneboronic acid (64.0 mg, 0.5 mmol), $[Rh(COD)(OH)]_2$ (1.4 mg, 0.003 mmol), BINAP (5.6 mg, 0.009 mmol) and K_2CO_3 (27.6 mg, 0.2 mmol) in 1.6 mL 1,4-dioxane and 16 μL H_2O for 24 h. The product was purified by flash column chromatography on silica gel (DCM : hexane = 1: 10) and obtained as a yellow oil (58.2 mg, 79% yield), R_f = 0.33 (DCM : hexane = 1: 5). **^{1}H NMR** (500 MHz, $CDCl_3$): δ (ppm) 7.41 - 7.23 (m, 6H), 7.18 (dd, *J* = 5.1, 2.9 Hz, 1H), 7.11 - 7.04 (m, 2H), 6.99 - 6.90 (m, 2H), 6.26 (t, *J* = 7.5 Hz, 1H), 5.21 (s, 1H), 4.13 (q, *J* = 7.1 Hz, 2H), 3.70 - 3.42 (m, 2H), 1.13 (t, *J* = 7.1 Hz, 3H). **^{13}C NMR** (126 MHz, $CDCl_3$): δ (ppm) 172.4, 142.5, 140.0, 136.9, 132.9, 130.8, 128.9, 128.4, 128.3, 127.2, 127.2, 125.8, 124.9, 121.6, 121.0, 61.3, 52.3, 29.7, 14.1. **HRMS** m/z (ESI): calcd. for $C_{21}H_{20}O_2S_2Na$ $[M+Na]^+$: 391.0798; found: 391.0797.

ethyl (*E*)-3,5-di(furan-3-yl)-2-phenylpent-3-enoate (2n)

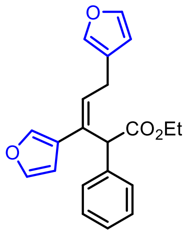

Following the general procedure (III), reaction was run using monofluorodiene **1a** (44.1 mg, 0.2 mmol), 3-furanboronic acid (60.0 mg, 0.5 mmol), $[Rh(COD)(OH)]_2$ (1.4 mg, 0.003 mmol), BINAP (5.6 mg, 0.009 mmol) and K_2CO_3 (27.6 mg, 0.2 mmol) in 1.6 mL 1,4-dioxane and 16 μL H_2O for 24 h. The product was purified by flash column chromatography on silica gel (DCM : hexane = 1: 10) and obtained as a colorless oil (53.8 mg, 80% yield), R_f = 0.33 (DCM : hexane = 1: 5). **^{1}H NMR** (500 MHz, $CDCl_3$): δ (ppm) 7.49 - 7.14 (m, 9H), 6.50 - 6.39 (m, 1H), 6.31 - 6.24 (m, 1H), 6.10 (t, *J* = 7.5 Hz, 1H), 5.13 (s, 1H), 4.18 (q, *J* = 7.1 Hz, 2H), 3.52 - 3.27 (m, 2H), 1.20 (t, *J* = 7.1 Hz, 3H). **^{13}C NMR** (126 MHz, $CDCl_3$): δ (ppm) 172.3, 143.1, 142.5, 139.9, 139.4, 136.8, 129.4, 129.2, 128.7, 128.5, 127.2, 126.1, 123.0, 111.2, 109.5, 61.3, 51.7, 24.3, 14.2. **HRMS** m/z (ESI): calcd. for $C_{21}H_{20}O_4Na$ $[M+Na]^+$: 359.1259; found: 359.1254.

ethyl (*E*)-3,5-diphenyl-2-(4-(trifluoromethyl)phenyl)pent-3-enoate (2o)

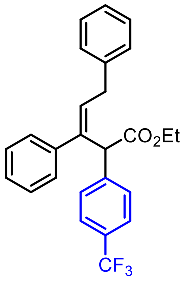

Following the general procedure (III), reaction was run using monofluorodiene **1o** (47.6 mg, 0.2 mmol), phenylboronic acid (61.3 mg, 0.5 mmol), $[Rh(COD)(OH)]_2$ (1.4 mg, 0.003 mmol), BINAP (5.6 mg, 0.009 mmol) and K_2CO_3 (27.6 mg, 0.2 mmol) in 1.6 mL 1,4-dioxane and 16 μL H_2O for 24 h. The product was purified by flash column chromatography on silica gel (DCM : hexane = 1: 8) and obtained as a colorless oil (68.7 mg, 81% yield), R_f = 0.30 (DCM : hexane = 1: 5). **^{1}H NMR** (500 MHz, $CDCl_3$): δ (ppm) 7.59 (d, *J* = 8.1 Hz, 2H), 7.47 (d, *J* = 8.1 Hz, 2H), 7.38 - 7.23 (m, 7H), 7.22 - 7.16 (m, 3H), 6.20 (t, *J* = 7.5 Hz, 1H), 5.28 (s, 1H), 4.13 (q, *J* = 7.1 Hz, 2H), 3.54 (qd, *J* = 16.2, 7.5 Hz, 2H), 1.12 (t, *J* = 7.1 Hz, 3H). **^{13}C NMR** (126 MHz, $CDCl_3$): δ (ppm) 171.8, 141.9, 141.0, 139.7, 137.3, 133.2, 129.6, 128.9 (m), 128.7, 128.5, 128.3, 127.5, 127.4, 126.4, 125.3 (q, *J* = 3.7 Hz), 122.1 (q, *J* = 271.9 Hz), 61.5, 52.8, 35.2, 14.0. **^{19}F NMR** (471 MHz, $CDCl_3$): δ (ppm) -62.44 (s, 1F). **HRMS** m/z (ESI): calcd. for $C_{26}H_{23}F_3O_2Na$ $[M+Na]^+$: 447.1543; found: 447.1542.

ethyl (*E*)-2-(4-methoxyphenyl)-3,5-diphenylpent-3-enoate (2p)

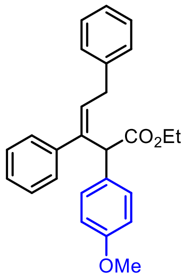

Following the general procedure (III), reaction was run using monofluorodiene **1p** (50.2 mg, 0.2 mmol), phenylboronic acid (61.3 mg, 0.5 mmol), $[Rh(COD)(OH)]_2$ (1.4 mg, 0.003 mmol), BINAP (5.6 mg, 0.009 mmol) and K_2CO_3 (27.6 mg, 0.2 mmol) in 1.6 mL 1,4-dioxane and 16 μL H_2O for 24 h. The product was purified by flash column chromatography on silica gel (DCM : hexane = 1: 3) and obtained as a colorless oil (67.2 mg, 87% yield), R_f = 0.35 (DCM : hexane = 1: 1). **^{1}H NMR** (500 MHz, $CDCl_3$): δ (ppm) 7.37 - 7.15 (m, 12H), 6.96 – 6.78 (m, 2H), 6.10 (t, J = 7.4 Hz, 1H), 5.18 (s, 1H), 4.09 (q, J = 7.1 Hz, 2H), 3.82 (s, 3H), 3.54 (qd, J = 16.3, 7.5 Hz, 2H), 1.09 (t, J = 7.1 Hz, 3H). **^{13}C NMR** (126 MHz, $CDCl_3$): δ (ppm) 172.8, 158.7, 142.5, 140.2, 138.2, 132.5, 130.2, 128.9, 128.6, 128.1, 127.6, 127.1, 126.3, 113.8, 61.1, 55.4, 52.4, 35.2, 14.1. **HRMS** m/z (ESI): calcd. for $C_{26}H_{26}O_3Na$ $[M+Na]^+$: 409.1778; found: 409.1774.

ethyl (*E*)-2-(4-fluorophenyl)-3,5-diphenylpent-3-enoate (2q)

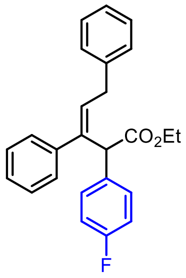

Following the general procedure (III), reaction was run using monofluorodiene **1q** (47.6 mg, 0.2 mmol), phenylboronic acid (61.3 mg, 0.5 mmol), $[Rh(COD)(OH)]_2$ (1.4 mg, 0.003 mmol), BINAP (5.6 mg, 0.009 mmol) and K_2CO_3 (27.6 mg, 0.2 mmol) in 1.6 mL 1,4-dioxane and 16 μL H_2O for 24 h. The product was purified by flash column chromatography on silica gel (DCM : hexane = 1: 10) and obtained as a yellow oil (56.1 mg, 75% yield), R_f = 0.29 (DCM : hexane = 1: 5). **^{1}H NMR** (500 MHz, $CDCl_3$): 7.41 - 7.17 (m, 12H), 7.09 - 7.00 (m, 8.5 Hz, 2H), 6.14 (t, J = 7.4 Hz, 1H), 5.21 (s, 1H), 4.12 (q, J = 7.2 Hz, 2H), 3.56 (tt, J = 16.3, 8.2 Hz, 2H), 1.12 (t, J = 7.1 Hz, 3H). **^{13}C NMR** (126 MHz, $CDCl_3$): δ (ppm) 172.4, 162.0 (d, J = 245.9 Hz), 142.2, 139.9, 138.0, 132.7, 130.8 (d, J = 7.9 Hz), 128.7, 128.6, 128.1, 127.6, 127.2, 126.4, 115.2 (d, J = 21.3 Hz), 108.4 (d, J = 14.1 Hz), 61.3, 52.3, 35.2, 14.0. **^{19}F NMR** (471 MHz, $CDCl_3$): δ (ppm) -114.75 - -114.80 (m, 1F). **HRMS** m/z (ESI): calcd. for $C_{25}H_{23}FO_2Na$ $[M+Na]^+$: 397.1576; found: 397.1574.

ethyl (*E*)-2-(4-chlorophenyl)-3,5-diphenylpent-3-enoate (2r)

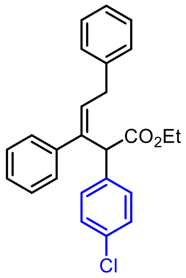

Following the general procedure (III), reaction was run using monofluorodiene **1r** (50.8 mg, 0.2 mmol), phenylboronic acid (61.3 mg, 0.5 mmol), $[Rh(COD)(OH)]_2$ (1.4 mg, 0.003 mmol), BINAP (5.6 mg, 0.009 mmol) and K_2CO_3 (27.6 mg, 0.2 mmol) in 1.6 mL 1,4-dioxane and 16 μL H_2O for 24 h. The product was purified by flash column chromatography on silica gel (DCM : hexane = 1: 8) and obtained as a colorless oil (63.2 mg, 81% yield), R_f = 0.36 (DCM : hexane = 1: 5). **^{1}H NMR** (500 MHz, $CDCl_3$): 7.38 - 7.20 (m, 14H), 6.14 (t, J = 7.4 Hz, 1H), 5.19 (s, 1H), 4.11 (q, J = 7.1 Hz, 2H), 3.76 - 3.35 (m, 2H), 1.10 (t, J = 7.1 Hz, 3H). **^{13}C NMR** (126 MHz, $CDCl_3$): δ (ppm) 172.1, 142.1, 139.9, 137.7, 135.4, 133.1, 132.9, 130.6, 128.7, 128.6, 128.5,

128.2, 127.5, 127.3, 126.4, 61.4, 52.4, 35.2, 14.0. **HRMS** m/z (ESI): calcd. for $C_{25}H_{23}ClO_2Na$ [M+Na]$^+$: 413.1281; found: 413.1279.

ethyl (*E*)-2-([1,1'-biphenyl]-4-yl)-3,5-diphenylpent-3-enoate (2s)

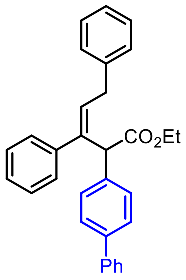

Following the general procedure (III), reaction was run using monofluorodiene **1s** (59.2 mg, 0.2 mmol), phenylboronic acid (61.3 mg, 0.5 mmol), $[Rh(COD)(OH)]_2$ (1.4 mg, 0.003 mmol), BINAP (5.6 mg, 0.009 mmol) and K_2CO_3 (27.6 mg, 0.2 mmol) in 1.6 mL 1,4-dioxane and 16 μL H_2O for 24 h. The product was purified by flash column chromatography on silica gel (DCM : hexane = 1: 3) and obtained as a yellow oil (74.3 mg, 86% yield), R_f = 0.30 (DCM : hexane = 1: 1). **^{1}H NMR** (500 MHz, $CDCl_3$): 7.64 - 6.86 (m, 19H), 6.23 (t, *J* = 7.4 Hz, 1H), 5.34 (s, 1H), 4.18 (q, *J* = 7.1 Hz, 2H), 3.63 (qd, *J* = 16.2, 7.4 Hz, 2H), 1.17 (t, *J* = 7.1 Hz, 3H). **^{13}C NMR** (126 MHz, $CDCl_3$): δ (ppm) 172.4, 141.3, 141.0, 140.7, 139.8, 139.2, 139.1, 137.6, 136.9, 132.6, 129.0, 128.9, 128.8, 128.4, 127.8, 127.3, 127.0, 127.0, 126.7, 61.2, 53.0, 34.8, 14.0. **HRMS** m/z (ESI): calcd. for $C_{31}H_{28}O_2Na$ [M+Na]$^+$: 455.1983; found: 455.1982.

ethyl (*E*)-2-(naphthalen-2-yl)-3,5-diphenylpent-3-enoate (2t)

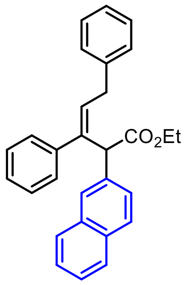

Following the general procedure (III), reaction was run using monofluorodiene **1t** (54.0 mg, 0.2 mmol), phenylboronic acid (61.3 mg, 0.5 mmol), $[Rh(COD)(OH)]_2$ (1.4 mg, 0.003 mmol), BINAP (5.6 mg, 0.009 mmol) and K_2CO_3 (27.6 mg, 0.2 mmol) in 1.6 mL 1,4-dioxane and 16 μL H_2O for 24 h. The product was purified by flash column chromatography on silica gel (DCM : hexane = 1: 10) and obtained as a yellow oil (65.8 mg, 81% yield), R_f = 0.32 (DCM : hexane = 1: 5). **^{1}H NMR** (500 MHz, $CDCl_3$): 7.90 - 7.85 (M, 4H), 7.61 - 7.46 (m, 3H), 7.42 - 7.15 (m, 10H), 6.25 (t, *J* = 7.4 Hz, 1H), 5.43 (s, 1H), 4.17 (q, *J* = 7.1 Hz, 2H), 3.61 (qd, *J* = 16.2, 7.4 Hz, 2H), 1.15 (t, *J* = 7.1 Hz, 3H). **^{13}C NMR** (126 MHz, $CDCl_3$): δ (ppm) 142.5, 140.0, 137.7, 134.5, 133.4, 133.0, 132.6, 128.6, 128.1, 127.9, 127.7, 127.6, 127.5, 127.2, 126.3, 126.1, 126.0, 61.2, 53.3, 35.3, 14.1. **HRMS** m/z (ESI): calcd. for $C_{29}H_{26}O_2Na$ [M+Na]$^+$: 429.1827; found: 429.1825.

ethyl (*E*)-3,5-diphenyl-2-(thiophen-2-yl)pent-3-enoate (2u)

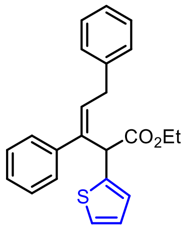

Following the general procedure (III), reaction was run using monofluorodiene **1u** (45.2 mg, 0.2 mmol), phenylboronic acid (61.3 mg, 0.5 mmol), $[Rh(COD)(OH)]_2$ (1.4 mg, 0.003 mmol), BINAP (5.6 mg, 0.009 mmol) and K_2CO_3 (27.6 mg, 0.2 mmol) in 1.6 mL 1,4-dioxane and 16 μL H_2O for 24 h. The product was purified by flash column chromatography on silica gel (DCM :

hexane = 1: 6) and obtained as a yellow oil (55.8 mg, 77% yield), R_f = 0.37 (DCM : hexane = 1: 3). **^{1}H NMR** (500 MHz, $CDCl_3$): 7.55 - 7.20 (m, 12H), 7.09 (dd, J = 5.0, 1.4 Hz, 1H), 6.13 (t, J = 7.4 Hz, 1H), 5.21 (d, J = 1.2 Hz, 1H), 4.13 (qd, J = 7.1, 2.7 Hz, 2H), 3.59 (qd, J = 16.2, 7.5 Hz, 2H), 1.13 (t, J = 7.1 Hz, 3H). **^{13}C NMR** (126 MHz, $CDCl_3$): δ (ppm) 172.0, 142.2, 140.1, 138.2, 137.3, 132.2, 128.8, 128.6, 128.6, 128.2, 127.3, 127.2, 126.3, 125.3, 123.2, 61.2, 48.7, 35.1, 14.1. **HRMS** m/z (ESI): calcd. for $C_{23}H_{22}O_2SNa$ [M+Na]$^+$: 385.1233; found: 385.1233.

isopropyl (*E*)-2,3,5-triphenylpent-3-enoate (2v)

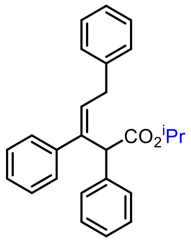

Following the general procedure (III), reaction was run using monofluorodiene **1v** (46.8 mg, 0.2 mmol), phenylboronic acid (61.3 mg, 0.5 mmol), $[Rh(COD)(OH)]_2$ (1.4 mg, 0.003 mmol), BINAP (5.6 mg, 0.009 mmol) and K_2CO_3 (27.6 mg, 0.2 mmol) in 1.6 mL 1,4-dioxane and 16 μL H_2O for 24 h. The product was purified by flash column chromatography on silica gel (DCM : hexane = 1: 6) and obtained as a colorless oil (56.0 mg, 81% yield), R_f = 0.29 (DCM : hexane = 1: 3). **^{1}H NMR** (500 MHz, $CDCl_3$): 7.36 - 7.12 (m, 13H), 7.04 - 6.92 (m, 2H), 6.12 (t, J = 7.4 Hz, 1H), 5.16 (s, 1H), 4.95 (p, J = 6.3 Hz, 1H), 3.66 - 3.30 (m, 2H), 1.19 (d, J = 6.3 Hz, 3H), 0.93 (d, J = 6.3 Hz, 3H). **^{13}C NMR** (126 MHz, $CDCl_3$): δ (ppm) 171.8, 162.9, 161.0, 142.1, 139.9, 138.0, 132.6, 130.9, 130.8, 128.7, 128.6, 128.1, 127.6, 127.2, 126.4, 68.9, 68.8, 52.4, 35.1. **HRMS** m/z (ESI): calcd. for $C_{26}H_{26}O_2Na$ [M+Na]$^+$: 393.1823; found: 393.1825.

benzyl (*E*)-2-(4-fluorophenyl)-3,5-diphenylpent-3-enoate (2w)

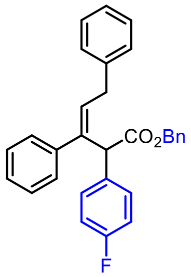

Following the general procedure (III), reaction was run using monofluorodiene **1w** (60.0 mg, 0.2 mmol), phenylboronic acid (61.3 mg, 0.5 mmol), $[Rh(COD)(OH)]_2$ (1.4 mg, 0.003 mmol), BINAP (5.6 mg, 0.009 mmol) and K_2CO_3 (27.6 mg, 0.2 mmol) in 1.6 mL 1,4-dioxane and 16 μL H_2O for 24 h. The product was purified by flash column chromatography on silica gel (DCM : hexane = 1: 10) and obtained as a colorless oil (61.9 mg, 71% yield), R_f = 0.30 (DCM : hexane = 1: 5). **^{1}H NMR** (500 MHz, $CDCl_3$): 7.41 - 7.25 (m, 15H), 7.19 - 7.08 (m, 2H), 7.02 (t, J = 8.7 Hz, 2H), 6.12 (t, J = 7.4 Hz, 1H), 5.27 (s, 1H), 5.09 (q, J = 12.3 Hz, 2H), 3.76 - 3.30 (m, 2H). **^{13}C NMR** (126 MHz, $CDCl_3$): δ (ppm) 172.2, 162.0 (d, J = 245.8 Hz), 142.0, 139.8, 137.7, 135.6, 132.9, 132.4 (d, J = 3.3 Hz), 130.8 (d, J = 8.0 Hz), 128.7, 128.6, 128.6, 128.5, 128.4, 128.2, 127.5, 127.3, 126.3, 115.3 (d, J = 21.3 Hz), 67.2, 52.2, 35.1. **^{19}F NMR** (471 MHz, $CDCl_3$): δ (ppm) -115.59 - -115.65 (m, 1F). **HRMS** m/z (ESI): calcd. for $C_{30}H_{25}FO_2Na$ [M+Na]$^+$: 459.1734; found: 459.1731.

ethyl (*E*)-3,5-di(naphthalen-2-yl)-2-phenylpent-3-enoate (2x)

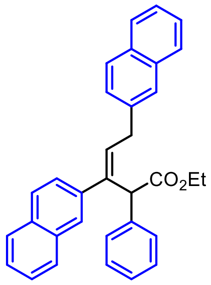

Following the general procedure (III), reaction was run using monofluorodiene **1a** (44.2 mg, 0.2 mmol), 2-naphthaleneboronic acid (86.0 mg, 0.5 mmol), $[Rh(COD)(OH)]_2$ (1.4 mg, 0.003 mmol), BINAP (5.6 mg, 0.009 mmol) and K_2CO_3 (27.6 mg, 0.2 mmol) in 1.6 mL 1,4-dioxane and 16 μL H_2O for 24 h. The product was purified by flash column chromatography on silica gel (DCM : hexane = 1: 10) and obtained as a colorless oil (73.9 mg, 81% yield), R_f = 0.26 (DCM : hexane = 1: 5). **^{1}H NMR** (500 MHz, $CDCl_3$): 7.90 - 7.72 (m, 7H), 7.65 (s, 1H), 7.55 - 7.43 (m, 7H), 7.43 - 7.32 (m, 4H), 6.35 (t, *J* = 7.3 Hz, 1H), 5.42 (s, 1H), 4.15 (qd, *J* = 7.1, 4.2 Hz, 2H), 3.79 (h, *J* = 8.5, 8.0 Hz, 2H), 1.12 (t, *J* = 7.1 Hz, 3H). **^{13}C NMR** (126 MHz, $CDCl_3$): δ (ppm) 172.5, 140.0, 138.2, 137.5, 137.1, 133.8, 133.3, 133.2, 132.6, 132.2, 129.2, 128.5, 128.3, 128.2, 127.8, 127.6, 127.6, 127.4, 127.2, 126.8, 126.2, 126.1, 126.1, 125.9, 125.5, 61.3, 53.3, 35.6, 14.1. **HRMS** m/z (ESI): calcd. for $C_{33}H_{28}O_2Na$ $[M+Na]^+$: 479.1986; found: 479.1982.

ethyl (*E*)-2-phenyl-3,5-di(triphenylen-2-yl)pent-3-enoate (2y)

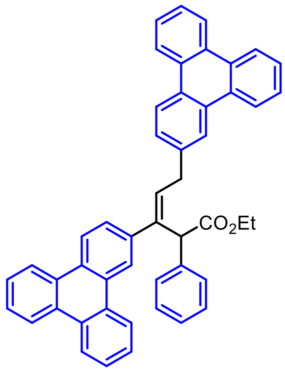

Following the general procedure (III), reaction was run using monofluorodiene **1a** (44.2 mg, 0.2 mmol), 2-triphenylenylboronic acid (136.1 mg, 0.5 mmol), $[Rh(COD)(OH)]_2$ (1.4 mg, 0.003 mmol), BINAP (5.6 mg, 0.009 mmol) and K_2CO_3 (27.6 mg, 0.2 mmol) in 1.6 mL 1,4-dioxane and 16 μL H_2O for 24 h. The product was purified by flash column chromatography on silica gel (DCM : hexane = 1: 5) and obtained as a white solid (112.9 mg, 86% yield), R_f = 0.31 (DCM : hexane = 1: 3). **^{1}H NMR** (500 MHz, $CDCl_3$): 8.67 - 8.47 (m, 12H), 7.69 - 7.52 (m, 12H), 7.47 - 7.32 (m, 3H)., 6.49 (t, *J* = 7.5 Hz, 1H), 5.55 (s, 1H), 4.16 (q, *J* = 7.1 Hz, 2H), 4.02 - 3.72 (m, 2H), 1.08 (t, *J* = 7.1 Hz, 3H). **^{13}C NMR** (126 MHz, $CDCl_3$): δ (ppm) 172.6, 140.9, 138.8, 138.5, 137.3, 133.1, 130.1, 130.0, 130.0, 129.9, 129.8, 129.8, 129.8, 129.7, 129.5, 129.2, 128.8, 128.7, 128.3, 128.1, 127.4, 127.3, 127.3, 127.3, 127.2, 127.1, 126.9, 123.8, 123.5, 123.5, 123.4, 123.4, 123.4, 123.3, 123.3, 123.2, 123.1, 122.4, 61.4, 53.3, 35.7, 14.2. **HRMS** m/z (ESI): calcd. for $C_{49}H_{36}O_2Na$ $[M+Na]^+$: 679.2610; found: 679.2613.

ethyl (*E*)-2-([1,1'-biphenyl]-4-yl)-3,5-di(triphenylen-2-yl)pent-3-enoate (2z)

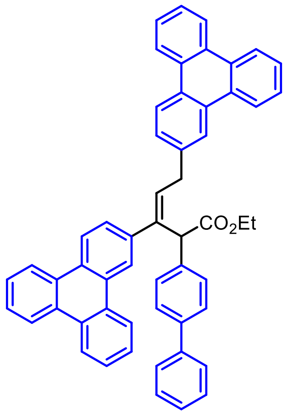

Following the general procedure (III), reaction was run using monofluorodiene **1s** (59.2 mg, 0.2 mmol), 2-triphenylenylboronic acid (136.1 mg, 0.5 mmol), $[Rh(COD)(OH)]_2$ (1.4 mg, 0.003 mmol), BINAP (5.6 mg, 0.009 mmol) and K_2CO_3 (27.6 mg, 0.2 mmol) in 1.6 mL 1,4-dioxane and 16 μL H_2O for 24 h. The product was purified by flash column chromatography on silica gel (DCM : hexane = 1: 6) and obtained as a white solid (121.9 mg, 83% yield), R_f = 0.33 (DCM : hexane = 1: 4). **^{1}H NMR** (500 MHz, $CDCl_3$): 8.84 - 8.27 (m, 12H), 7.88 - 7.53 (m, 16H), 7.46 (dd, J = 8.4, 6.9 Hz, 2H), 7.41 - 7.34 (m, 1H), 6.52 (t, J = 7.5 Hz, 1H), 5.59 (s, 1H), 4.58 - 4.15 (m, 2H), 3.93 (qd, J = 16.0, 7.5 Hz, 2H), 1.10 (t, J = 7.1 Hz, 3H). **^{13}C NMR** (126 MHz, $CDCl_3$): δ (ppm) 172.6, 140.9, 140.7, 140.1, 138.8, 138.4, 136.3, 133.1, 130.1, 130.0, 130.0, 129.9, 129.8, 129.7, 129.7, 129.5, 128.9, 128.3, 128.1, 127.4, 127.3, 127.3, 127.2, 127.1, 126.9, 123.8, 123.4, 123.3, 123.3, 123.2, 123.1, 122.3, 61.4, 53.0, 35.7, 14.2. **HRMS** m/z (ESI): calcd. for $C_{55}H_{40}O_2Na$ $[M+Na]^+$: 756.2929; found: 756.2921.

ethyl (*E*)-2-(naphthalen-2-yl)-3,5-di(triphenylen-2-yl)pent-3-enoate (2aa)

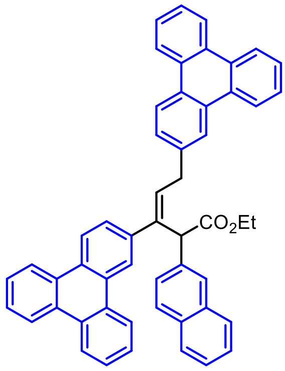

Following the general procedure (III), reaction was run using monofluorodiene **1t** (54.0 mg, 0.2 mmol), 2-triphenylenylboronic acid (136.1 mg, 0.5 mmol), $[Rh(COD)(OH)]_2$ (1.4 mg, 0.003 mmol), BINAP (5.6 mg, 0.009 mmol) and K_2CO_3 (27.6 mg, 0.2 mmol) in 1.6 mL 1,4-dioxane and 16 μL H_2O for 24 h. The product was purified by flash column chromatography on silica gel (DCM : hexane = 1: 5) and obtained as a white solid (114.4 mg, 81% yield), R_f = 0.31 (DCM : hexane = 1: 3). **^{1}H NMR** (500 MHz, $CDCl_3$): 8.80 - 8.39 (m, 14H), 8.03 (s, 1H), 7.88 (t, J = 8.0 Hz, 2H), 7.79 (d, J = 8.1 Hz, 1H), 7.72 (dd, J = 8.6, 1.9 Hz, 1H), 7.68 - 7.41 (m, 10H), 6.57 (t, J = 7.5 Hz, 1H), 5.70 (s, 1H), 4.19 (qd, J = 7.2, 2.1 Hz, 2H), 3.92 (qd, J = 16.0, 7.5 Hz, 2H), 1.09 (t, J = 7.1 Hz, 3H). **^{13}C NMR** (126 MHz, $CDCl_3$): δ (ppm) 172.6, 141.0, 138.8, 138.3, 134.8, 133.5, 133.4, 132.7, 130.1, 130.0, 130.0, 129.8, 129.8, 129.7, 129.7, 129.6, 129.6, 129.6, 128.8, 128.3, 128.2, 128.1, 127.7, 127.7, 127.7, 127.4, 127.3, 127.2, 127.1, 126.8, 126.3, 126.2, 123.8, 123.4, 123.4, 123.3, 123.3, 123.2, 123.1, 61.5, 53.5, 35.8, 14.2. **HRMS** m/z (ESI): calcd. for $C_{53}H_{38}O_2Na$ $[M+Na]^+$: 729.2774; found: 729.2764.

ethyl 3-fluoro-2,5-diphenylpent-3-enoate (3a)

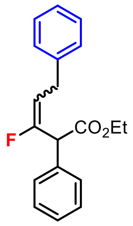

Following the general procedure (IV), reaction was run using monofluorodiene **1a** (44.1 mg, 0.2 mmol), phenylboronic acid (61.3 mg, 0.5 mmol), $[Rh(COD)(OH)]_2$ (1.4 mg, 0.003 mmol) and K_2CO_3 (27.6 mg, 0.2 mmol) in 1.6 mL 1,4-dioxane and 16 μL H_2O for 12 h. The product was purified by flash column chromatography on silica gel (DCM : hexane = 1: 10) and obtained as a colorless oil (47.7 mg, 80% yield, dr= 1.2 : 1), R_f = 0.32 (DCM : hexane = 1: 5). Major product: **^{1}H NMR** (500 MHz, $CDCl_3$): *δ* (ppm) 7.44 - 7.16 (m, 10H), 5.49 (dt, *J* = 20.5, 8.2 Hz, 1H), 4.89 (d, *J* = 30.3 Hz, 1H), 4.30 - 4.24 (m, 2H), 3.52 - 3.43 (m, 2H), 1.31 (t, *J* = 7.1 Hz, 3H). **^{13}C NMR** (126 MHz, $CDCl_3$): *δ* (ppm) 169.9, 156.6 (d, *J* = 256.9 Hz), 139.9 (d, *J* = 1.8 Hz), 134.4 (d, *J* = 3.6 Hz), 128.8, 128.8, 128.5, 128.3, 128.0, 126.1, 108.2 (d, *J* = 14.1 Hz), 61.6, 54.8 (d, *J* = 28.9 Hz), 29.9 (d, *J* = 5.5 Hz), 14.1. **^{19}F NMR** (471 MHz, $CDCl_3$): *δ* (ppm) -109.12 (dd, *J* = 30.3, 20.6 Hz). Minor product: **^{1}H NMR** (500 MHz, $CDCl_3$): *δ* (ppm) 7.44 - 7.16 (m, 10H), δ 4.88 (dt, *J* = 35.9, 7.7 Hz, 1H), 4.53 (dd, *J* = 12.8 Hz, 1H). 4.30 - 4.18 (m, 2H), 3.59 - 3.48 (m, 2H), 1.27 (t, *J* = 7.2 Hz, 3H). **^{19}F NMR** (471 MHz, $CDCl_3$): *δ* (ppm) -62.38 (s, 3F), -112.19 (dd, *J* = 35.7, 12.9 Hz). **HRMS** m/z (ESI): calcd. for $C_{19}H_{19}FO_2Na$ $[M+Na]^+$: 321.1263; found: 321.1261.

ethyl (*Z*)-3-fluoro-2,5-diphenylpent-3-enoate ((*Z*)-3a)

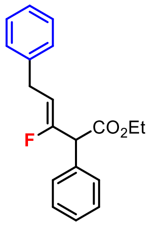

Followed the procedure in the isomerization studies (page S11-12). Product was obtained in 53.0 mg, 89% yield, dr > 99:1, colorless oil, R_f = 0.33 (DCM : hexane = 1: 5). **^{1}H NMR** (500 MHz, $CDCl_3$): *δ* (ppm) 7.43 - 7.08 (m, 10H), 4.88 (dt, *J* = 35.9, 7.7 Hz, 1H), 4.53 (d, *J* = 12.8, 1H), 4.32 - 4.05 (m, 2H), 3.47 (d, *J* = 7.6 Hz, 2H), 1.27 (t, *J* = 7.1 Hz, 3H). **^{13}C NMR** (126 MHz, $CDCl_3$): *δ* (ppm) 170.0, 156.7 (d, *J* = 257.0 Hz), 140.0 (d, *J* = 1.8 Hz), 134.5 (d, *J* = 3.7 Hz), 128.9, 128.9, 128.6, 128.4, 128.2, 126.2, 108.3 (d, *J* = 14.0 Hz), 61.7, 54.9 (d, *J* = 28.7 Hz), 30.0 (d, *J* = 5.5 Hz), 14.2. **^{19}F NMR** (471 MHz, $CDCl_3$): *δ* (ppm) -112.19 (dd, *J* = 35.7, 13.0 Hz). **HRMS** m/z (ESI): calcd. for $C_{19}H_{19}FO_2Na$ $[M+Na]^+$: 321.1263; found: 321.1261. The spectral data are in accordance with the literature example.[1]

ethyl 3-fluoro-2-phenyl-5-(4-(trifluoromethyl)phenyl)pent-3-enoate (3b)

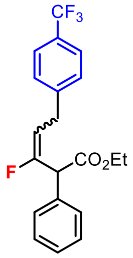

Following the general procedure (IV), reaction was run using monofluorodiene **1a** (44.1 mg, 0.2 mmol), 4-trifluoromethylphenylboronic acid (95.0 mg, 0.5 mmol), $[Rh(COD)(OH)]_2$ (1.4 mg, 0.003 mmol) and K_2CO_3 (27.6 mg, 0.2 mmol) in 1.6 mL 1,4-dioxane and 16 μL H_2O for 12 h. The product was purified by flash column chromatography on silica gel (DCM : hexane = 1: 10) and obtained as a colorless oil (57.1 mg, 78% yield, dr= 5.8 : 1), R_f = 0.30 (DCM : hexane = 1: 5). Major product: **^{1}H NMR** (500 MHz, $CDCl_3$): *δ* (ppm) 7.53 (d, *J* = 7.9 Hz, 2H), 7.39 - 7.21 (m, 7H). 4.86 (dt, *J* = 33.1, 6.6 Hz, 1H), 4.54 (d, *J* = 12.5 Hz, 1H), 4.29 - 4.16 (m, 2H), 3.48 (m, 2H), 1.27 (t, *J* = 7.3 Hz, 3H). **^{13}C NMR** (126 MHz, $CDCl_3$): *δ* (ppm) 169.9, 157.5 (d, *J* = 258.0 Hz), 144.1, 134.3 (d, *J* = 3.7 Hz), 129.0, 128.9, 128.7, 129.5 - 127.2 (m), 128.3, 125.5 (q, *J* = 3.9 Hz), 127.7 - 119.8 (m), 107.2 (d, *J* = 14.0 Hz), 61.8, 54.9 (d, *J* = 28.6 Hz), 29.8 (d, *J* = 5.7 Hz), 14.2. **^{19}F NMR** (471 MHz,

$CDCl_3$): δ (ppm) -62.34 (s, 3F), -109.59 (dd, J = 35.5, 12.4 Hz, 1F). Minor product: **^{1}H NMR** (500 MHz, $CDCl_3$): δ (ppm) 7.53 (d, J = 7.9 Hz, 2H), 7.39 - 7.21 (m, 7H), 5.46 (dt, J = 20.2, 8.2 Hz, 1H), 4.84 (d, J = 32.4 Hz, 1H), 4.32 - 4.15 (m, 2H), 3.48 (dd, J = 25.9, 8.6 Hz, 2H), 1.28 (t, J = 7.3 Hz, 3H). **^{19}F NMR** (471 MHz, $CDCl_3$): δ (ppm) -62.40 (s, 3F), -106.20 (dd, J = 30.1, 20.1 Hz, 1F). **HRMS** m/z (ESI): calcd. for $C_{20}H_{18}F_4O_2Na$ $[M+Na]^+$: 389.1136; found: 389.1135.

ethyl (*Z*)-3-fluoro-2-phenyl-5-(4-(trifluoromethyl)phenyl)pent-3-enoate ((*Z*)-3b)

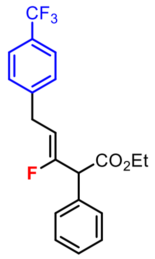

Followed the procedure in the isomerization studies (page S11-12). Product was obtained in 47.6 mg, 65% yield, dr > 99:1, colorless oil, R_f = 0.30 (DCM : hexane = 1: 5). **^{1}H NMR** (500 MHz, $CDCl_3$): δ (ppm) 7.53 (d, J = 8.0 Hz, 2H), 7.40 - 7.24 (m, 7H), 4.85 (dt, J = 35.5, 7.7 Hz, 1H), 4.52 (d, J = 12.5 Hz, 1H), 4.37 - 4.10 (m, 2H), 3.50 (m, 2H), 1.27 (t, J = 7.1 Hz, 3H). **^{13}C NMR** (126 MHz, $CDCl_3$): δ (ppm) 169.9, 157.5 (d, J = 258.1 Hz), 144.1, 134.3 (d, J = 3.9 Hz), 129.0, 128.9, 128.7, 129.5 - 127.2 (m), 128.3, 125.5 (q, J = 3.8 Hz), 127.7 - 119.8 (m), 107.2 (d, J = 14.1 Hz), 61.8, 54.9 (d, J = 28.6 Hz), 29.8 (d, J = 5.8 Hz), 14.2. **^{19}F NMR** (471 MHz, $CDCl_3$): δ (ppm) -62.35 (s, 3F), -109.59 (dd, J = 35.5, 12.4 Hz, 1F). **HRMS** m/z (ESI): calcd. for $C_{20}H_{18}F_4O_2Na$ $[M+Na]^+$: 389.1136; found: 389.1135. The spectral data are in accordance with the literature example.[1]

ethyl 3-fluoro-5-(4-methoxyphenyl)-2-phenylpent-3-enoate (3c)

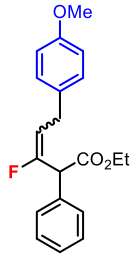

Following the general procedure (IV), reaction was run using monofluorodiene **1a** (44.1 mg, 0.2 mmol), 4-methoxyphenylboronic acid (76.0 mg, 0.5 mmol), $[Rh(COD)(OH)]_2$ (1.4 mg, 0.003 mmol) and K_2CO_3 (27.6 mg, 0.2 mmol) in 1.6 mL 1,4-dioxane and 16 μL H_2O for 12 h. The product was purified by flash column chromatography on silica gel (DCM : hexane = 1: 3) and obtained as a colorless oil (61.7 mg, 94% yield, dr= 6.9 : 1), R_f = 0.35 (DCM : hexane = 1: 1). Major product: **^{1}H NMR** (500 MHz, $CDCl_3$): δ (ppm) 7.43 - 7.30 (m, 5H), 7.10 (d, J = 8.1 Hz, 2H), 6.89 - 6.79 (m, 2H), 4.86 (dt, J = 35.9, 7.7 Hz, 1H), 4.52 (d, J = 12.9 Hz, 1H), 4.31 - 4.14 (m, 2H), 3.79 (s, 3H), 3.46 – 3.29 (m, 2H), 1.28 (t, J = 7.1 Hz, 3H). **^{13}C NMR** (126 MHz, $CDCl_3$): δ (ppm) 170.0, 158.1, 156.4 (d, J = 256.5 Hz), 134.6 (d, J = 3.6 Hz), 132.0 (d, J = 1.8 Hz), 129.3, 128.9, 128.9, 128.1, 114.0, 108.7 (d, J = 14.0 Hz), 61.7, 55.4, 54.9 (d, J = 29.0 Hz), 29.1 (d, J = 5.6 Hz), 14.2. **^{19}F NMR** (471 MHz, $CDCl_3$): δ (ppm) -111.48 (dd, J = 35.8, 12.9 Hz, 1F). Minor product: **^{1}H NMR** (500 MHz, $CDCl_3$): δ (ppm) 7.42 - 7.24 (m, 5H), 7.20 (dd, J = 8.9, 3.2 Hz, 2H), 6.76 (d, J = 8.1 Hz, 2H), 5.45 (dt, J = 20.6, 8.1 Hz, 1H), 4.87 (d, J = 31.7 Hz, 1H), 4.33 - 4.15 (m, 2H), 3.80 (s, 3H), 3.52 - 3.27 (m, 2H), 1.36 - 1.28 (m, 3H). **^{19}F NMR** (471 MHz, $CDCl_3$): δ (ppm) -108.36 (dd, J = 30.3, 20.6 Hz, 1F). **HRMS** m/z (ESI): calcd. for $C_{20}H_{21}FO_3Na$ $[M+Na]^+$: 351.1367; found: 351.1367.

ethyl (*Z*)-3-fluoro-5-(4-methoxyphenyl)-2-phenylpent-3-enoate ((*Z*)-3c)

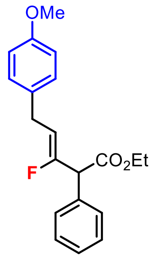

Followed the procedure in the isomerization studies (page S11-12). Product was obtained in 55.8 mg, 85% yield, dr = 29:1, colorless oil, R_f = 0.36 (DCM : hexane = 1: 3). **^{1}H NMR** (500 MHz, $CDCl_3$): δ (ppm) 7.35 - 7.27 (m, 4H), 7.25 - 7.16 (m, 3H), 6.91 (d, J = 8.7 Hz, 2H), 4.88 (dt, J = 35.9, 7.7 Hz, 1H), 4.49 (d, J = 12.2 Hz, 1H), 4.33 - 4.11 (m, 2H), 3.83 (s, 3H), 3.49 (d, J = 7.6 Hz, 2H), 1.29 (t, J = 7.2 Hz, 3H). **^{13}C NMR** (126 MHz, $CDCl_3$): δ (ppm) 170.3, 159.4, 157.0 (d, J = 256.8 Hz), 140.1 (d, J = 1.8 Hz), 130.1, 128.6, 128.4, 126.5 (d, J = 4.1 Hz), 126.2, 114.3, 108.0 (d, J = 14.1 Hz), 61.7, 55.4, 54.1 (d, J = 29.0 Hz), 30.0 (d, J = 5.5 Hz), 14.2. **^{19}F NMR** (471 MHz, $CDCl_3$): δ (ppm) -111.14 (dd, J = 35.8, 12.1 Hz, 1F). **HRMS** m/z (ESI): calcd. for $C_{20}H_{21}FO_3Na$ $[M+Na]^+$: 351.1367; found: 351.1367.

ethyl 5-([1,1'-biphenyl]-4-yl)-3-fluoro-2-phenylpent-3-enoate (3d)

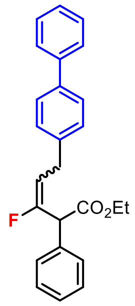

Following the general procedure (IV), reaction was run using monofluorodiene **1a** (44.1 mg, 0.2 mmol), 4-biphenylboronic acid (99.0 mg, 0.5 mmol), $[Rh(COD)(OH)]_2$ (1.4 mg, 0.003 mmol) and K_2CO_3 (27.6 mg, 0.2 mmol) in 1.6 mL 1,4-dioxane and 16 μL H_2O for 12 h. The product was purified by flash column chromatography on silica gel (DCM : hexane = 1: 2) and obtained as a colorless oil (72.6 mg, 97% yield, dr= 6.5 : 1), R_f = 0.30 (DCM : hexane = 1: 3). Major product: **^{1}H NMR** (500 MHz, $CDCl_3$): δ (ppm) 7.63 - 7.20 (m, 14H), 4.96 (dt, J = 35.5, 7.5 Hz, 1H), 4.59 (d, J = 12.8 Hz, 1H), 4.40 - 4.20 (m, 2H), 3.77 - 3.33 (m, 2H), 1.32 (t, J = 7.2 Hz, 3H). **^{13}C NMR** (126 MHz, $CDCl_3$): δ (ppm) 170.0, 156.7 (d, J = 257.0 Hz), 141.0, 139.2, 139.0 (d, J = 1.8 Hz), 134.5 (d, J = 3.8 Hz), 128.9, 128.8, 128.8, 128.1, 127.4, 127.3, 127.2, 108.1 (d, J = 14.0 Hz), 61.7, 54.9 (d, J = 28.8 Hz), 29.6 (d, J = 5.7 Hz), 14.1. **^{19}F NMR** (471 MHz, $CDCl_3$): δ (ppm) -110.78 (dd, J = 35.8, 12.8 Hz, 1F). Minor product: **^{1}H NMR** (500 MHz, $CDCl_3$): δ (ppm) 7.63 - 7.20 (m, 14H), 5.56 (dt, J = 20.5, 8.2 Hz, 1H), 4.94 (d, J = 30.5 Hz, 1H), 4.40 - 4.20 (m, 2H), 3.77 - 3.33 (m, 2H), 1.34 (t, J = 7.1 Hz, 3H). **^{19}F NMR** (471 MHz, $CDCl_3$): δ (ppm) -107.63 (dd, J = 30.3, 20.6 Hz, 1F). **HRMS** m/z (ESI): calcd. for $C_{25}H_{23}FO_2Na$ $[M+Na]^+$: 397.1577; found: 397.1574.

ethyl (*Z*)-5-([1,1'-biphenyl]-4-yl)-3-fluoro-2-phenylpent-3-enoate ((*Z*)-3d)

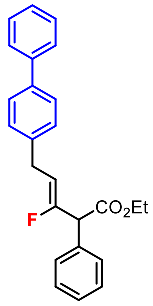

Followed the procedure in the isomerization studies (page S11-12). Product was obtained in 68.2 mg, 91% yield, dr > 99:1, colorless oil, R_f = 0.31 (DCM : hexane = 1: 3). **^{1}H NMR** (500 MHz, $CDCl_3$): δ (ppm) 7.61 - 7.50 (m, 4H), 7.47 - 7.32 (m, 8H), 7.27 (d, J = 8.0 Hz, 2H), 4.92 (dt, J = 35.8, 7.7 Hz, 1H), 4.55 (d, J = 12.8 Hz, 1H), 4.38 - 4.11 (m, 2H), 3.52 (d, J = 7.7 Hz, 2H),

1.29 (t, J = 7.1 Hz, 3H). **^{13}C NMR** (126 MHz, $CDCl_3$): δ (ppm) 170.0, 156.8 (d, J = 257.0 Hz), 141.1, 139.3, 139.1 (d, J = 1.8 Hz), 134.5 (d, J = 3.6 Hz), 129.0, 128.9, 128.9, 128.2, 127.3, 127.2, 127.1, 108.2 (d, J = 14.0 Hz), 61.8, 55.0 (d, J = 28.9 Hz), 29.6 (d, J = 5.6 Hz), 14.2. **^{19}F NMR** (471 MHz, $CDCl_3$): δ (ppm) -110.80 (dd, J = 36.0, 12.9 Hz, 1F). **HRMS** m/z (ESI): calcd. for $C_{25}H_{23}FO_2Na$ $[M+Na]^+$: 397.1577; found: 397.1574. The spectral data are in accordance with the literature example.[1]

ethyl 3-fluoro-5-(naphthalen-2-yl)-2-phenylpent-3-enoate (3e)

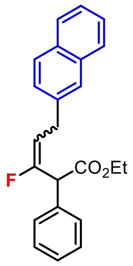

Following the general procedure (IV), reaction was run using monofluorodiene **1a** (44.1 mg, 0.2 mmol), 2-naphthaleneboronic acid (86.0 mg, 0.5 mmol), $[Rh(COD)(OH)]_2$ (1.4 mg, 0.003 mmol) and K_2CO_3 (27.6 mg, 0.2 mmol) in 1.6 mL 1,4-dioxane and 16 μL H_2O for 12 h. The product was purified by flash column chromatography on silica gel (DCM : hexane = 1: 3) and obtained as a colorless oil (62.7 mg, 90% yield, dr= 6.3 : 1), R_f = 0.35 (DCM : hexane = 1: 1). Major product: **^{1}H NMR** (500 MHz, $CDCl_3$): δ (ppm) 7.85 - 7.33 (m, 12H), 4.99 (dt, J = 35.8, 7.7 Hz, 1H), 4.59 (d, J = 12.9 Hz, 1H), 4.39 - 4.08 (m, 2H), 3.75 - 3.41 (m, 2H), 1.32 (t, J = 7.1 Hz, 3H). **^{13}C NMR** (126 MHz, $CDCl_3$): δ (ppm) 170.0, 156.8 (d, J = 257.0 Hz), 137.5 (d, J = 1.9 Hz), 134.5 (d, J = 3.6 Hz), 133.7, 132.2, 128.9, 128.9, 128.2, 128.2, 127.7, 127.6, 127.2, 126.5, 126.1, 125.5, 108.2 (d, J = 14.0 Hz), 61.7, 55.0 (d, J = 28.8 Hz), 30.2 (d, J = 5.6 Hz), 14.2. **^{19}F NMR** (471 MHz, $CDCl_3$): δ (ppm) -110.66 (dd, J = 35.7, 12.9 Hz, 1F). Minor product: **^{1}H NMR** (500 MHz, $CDCl_3$): δ (ppm) 7.85 - 7.33 (m, 12H), 5.60 (dt, J = 20.5, 8.2 Hz, 1H), 4.96 (d, J = 30.3 Hz, 1H), 4.39 - 4.08 (m, 2H), 3.75 - 3.41 (m, 2H), 1.32 (t, J = 7.2 Hz, 3H). **^{19}F NMR** (471 MHz, $CDCl_3$): δ (ppm) -107.42 (dd, J = 30.2, 20.5 Hz, 1F). **HRMS** m/z (ESI): calcd. for $C_{23}H_{21}FO_2Na$ $[M+Na]^+$: 371.1419; found: 371.1418.

ethyl (*Z*)-3-fluoro-5-(naphthalen-2-yl)-2-phenylpent-3-enoate ((*Z*)-3e)

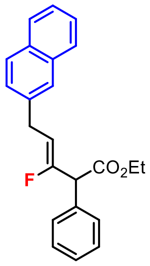

Followed the procedure in the isomerization studies (page S11-12). Product was obtained in 61.3 mg, 88% yield, dr > 99:1, colorless oil, R_f = 0.33 (DCM : hexane = 1: 1). **^{1}H NMR** (500 MHz, $CDCl_3$): δ (ppm) 7.85 - 7.74 (m, 3H), 7.61 (s, 1H), 7.49 – 7.30 (m, 8H), 4.96 (dt, J = 35.8, 7.7 Hz, 1H), 4.56 (d, J = 12.9 Hz, 1H), 4.34 - 4.06 (m, 2H), 3.63 (d, J = 7.6 Hz, 2H), 1.29 (t, J = 7.1 Hz, 3H). **^{13}C NMR** (126 MHz, $CDCl_3$): δ (ppm) 170.0, 156.8 (d, J = 257.0 Hz), 137.5 (d, J = 1.8 Hz), 134.5 (d, J = 3.6 Hz), 133.7, 132.2, 128.9, 128.9, 128.2, 128.2, 127.7, 127.6, 127.2, 126.5, 126.1, 125.5, 108.2 (d, J = 14.0 Hz), 61.8, 55.0 (d, J = 28.7 Hz), 30.2 (d, J = 5.5 Hz), 14.2. **^{19}F NMR** (471 MHz, $CDCl_3$): δ (ppm) -110.68 (dd, J = 35.9, 12.9 Hz, 1F). **HRMS** m/z (ESI): calcd. for $C_{23}H_{21}FO_2Na$ $[M+Na]^+$: 371.1419; found: 371.1418. The spectral data are in accordance with the literature example.[1]

ethyl 3-fluoro-2-(4-fluorophenyl)-5-(4-(trifluoromethyl)phenyl)pent-3-enoate (4)

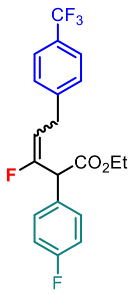

106.0 mg, 92% yield, dr = 2.3:1, colorless oil, R_f = 0.37 (DCM : hexane = 1: 3). Major product: **^{1}H NMR** (500 MHz, $CDCl_3$): δ (ppm) 7.53 (d, *J* = 8.0 Hz, 2H), 7.40 - 7.21 (m, 6H), 4.86 (dt, *J* = 33.3, 6.7 Hz, 1H), 4.54 (d, *J* = 12.6 Hz, 1H), 4.23 (dtt, *J* = 17.9, 7.1, 3.7 Hz, 2H), 3.49 (dd, *J* = 20.2, 6.0 Hz, 2H), 1.27 (t, *J* = 7.2 Hz, 3H). **^{13}C NMR** (126 MHz, $CDCl_3$): δ (ppm) 169.8, 157.4 (d, *J* = 258.0 Hz), 144.0, 134.2 (d, *J* = 3.9 Hz), 128.9, 128.8 - 128.5 (m), 128.8, 128.6 - 127.5 (m), 128.2, 128.0, 125.4 (q, *J* = 3.8 Hz), 107.1 (d, *J* = 14.1 Hz), 61.7, 54.8 (d, *J* = 28.7 Hz), 29.7 (d, *J* = 5.7 Hz), 14.1. **^{19}F NMR** (471 MHz, $CDCl_3$): δ (ppm) -62.35(s, 3F), -111.99 (dd, *J* = 35.8, 13.3 Hz, 1F), -115.59 (dt, *J* = 9.0, 5.3 Hz, 1F). Minor product: **^{1}H NMR** (500 MHz, $CDCl_3$): δ (ppm) 7.53 (d, *J* = 8.0 Hz, 2H), 7.40 - 7.21 (m, 6H), 5.46 (dt, *J* = 20.2, 8.2 Hz, 1H), 4.84 (d, *J* = 32.4 Hz, 1H), 4.23 (dtt, *J* = 17.9, 7.1, 3.7 Hz, 2H), 3.49 (dd, *J* = 20.2, 6.0 Hz, 2H), 1.28 (t, *J* = 7.2 Hz, 3H). **^{19}F NMR** (471 MHz, $CDCl_3$): δ (ppm) -62.30, -108.98 (dd, *J* = 30.2, 20.5 Hz), -114.17 (dq, *J* = 9.0, 5.3 Hz). **HRMS** m/z (ESI): calcd. for $C_{20}H_{17}F_5O_2Na$ $[M+Na]^+$: 407.0997; found: 407.0998.

ethyl (*E*)-2-(4-fluorophenyl)-3-(4-methoxyphenyl)-5-(4-(trifluoromethyl)phenyl)pent-3-enoate (5)

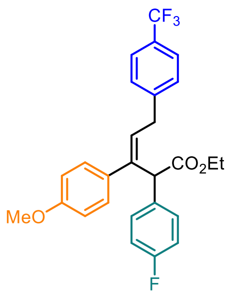

81.2 mg, 86% yield, dr > 99:1, colorless oil, R_f = 0.30 (DCM : hexane = 1: 3). **^{1}H NMR** (500 MHz, $CDCl_3$): δ (ppm) 7.56 (d, *J* = 8.1 Hz, 2H), 7.43 - 7.27 (m, 4H), 7.19 (d, *J* = 8.8 Hz, 2H), 7.02 (t, *J* = 8.7 Hz, 2H), 6.80 (d, *J* = 8.8 Hz, 2H), 6.02 (t, *J* = 7.5 Hz, 1H), 5.16 (s, 1H), 4.11 (qd, *J* = 7.1, 2.0 Hz, 2H), 3.80 (s, 3H), 3.71 - 3.34 (m, 2H), 1.13 (t, *J* = 7.1 Hz, 3H). **^{13}C NMR** (126 MHz, $CDCl_3$): δ (ppm) 172.3, 162.0 (d, *J* = 246.0 Hz), 159.0, 144.2, 138.4, 134.4, 132.5 (d, *J* = 3.2 Hz), 130.7, 130.7, 130.3, 130.0, 128.8 (d, *J* = 19.9 Hz), 125.5 (q, *J* = 3.7 Hz), 121.6 (d, *J* = 423.8 Hz), 115.3 (d, *J* = 21.3 Hz), 113.6, 61.4, 55.4, 52.3, 34.9, 14.1. **^{19}F NMR** (471 MHz, $CDCl_3$): δ (ppm) -62.35 (s, 3F), -115.59 (dq, *J* = 9.1, 5.4 Hz, 1F). **HRMS** m/z (ESI): calcd. for $C_{27}H_{24}F_4O_3Na$ $[M+Na]^+$: 495.1558; found: 495.1554.

(*E*)-1-morpholino-2,3,5-triphenylpent-3-en-1-one (7)

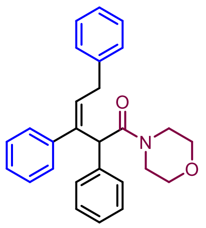

50.8 mg, 64% yield, dr > 99:1, colorless oil, R_f = 0.33 (DCM : hexane = 1: 5). **^{1}H NMR** (500 MHz, $CDCl_3$): δ (ppm) 7.44 - 6.96 (m, 15H), 6.25 (t, *J* = 7.5 Hz, 1H), 5.14 (s, 1H), 4.26 - 3.14 (m, 10H). **^{13}C NMR** (126 MHz, $CDCl_3$): δ (ppm) 170.8, 142.6, 140.1, 138.4, 138.0, 133.0, 129.3, 128.7, 128.6, 128.5, 128.2, 127.4, 127.3, 127.2, 126.4, 66.9, 66.4, 52.3, 46.3, 42.5, 34.9. **HRMS** m/z (ESI): calcd. for $C_{27}H_{27}NO_2Na$ $[M+Na]^+$: 420.1935; found: 420.1934.

XIII. Spectra.

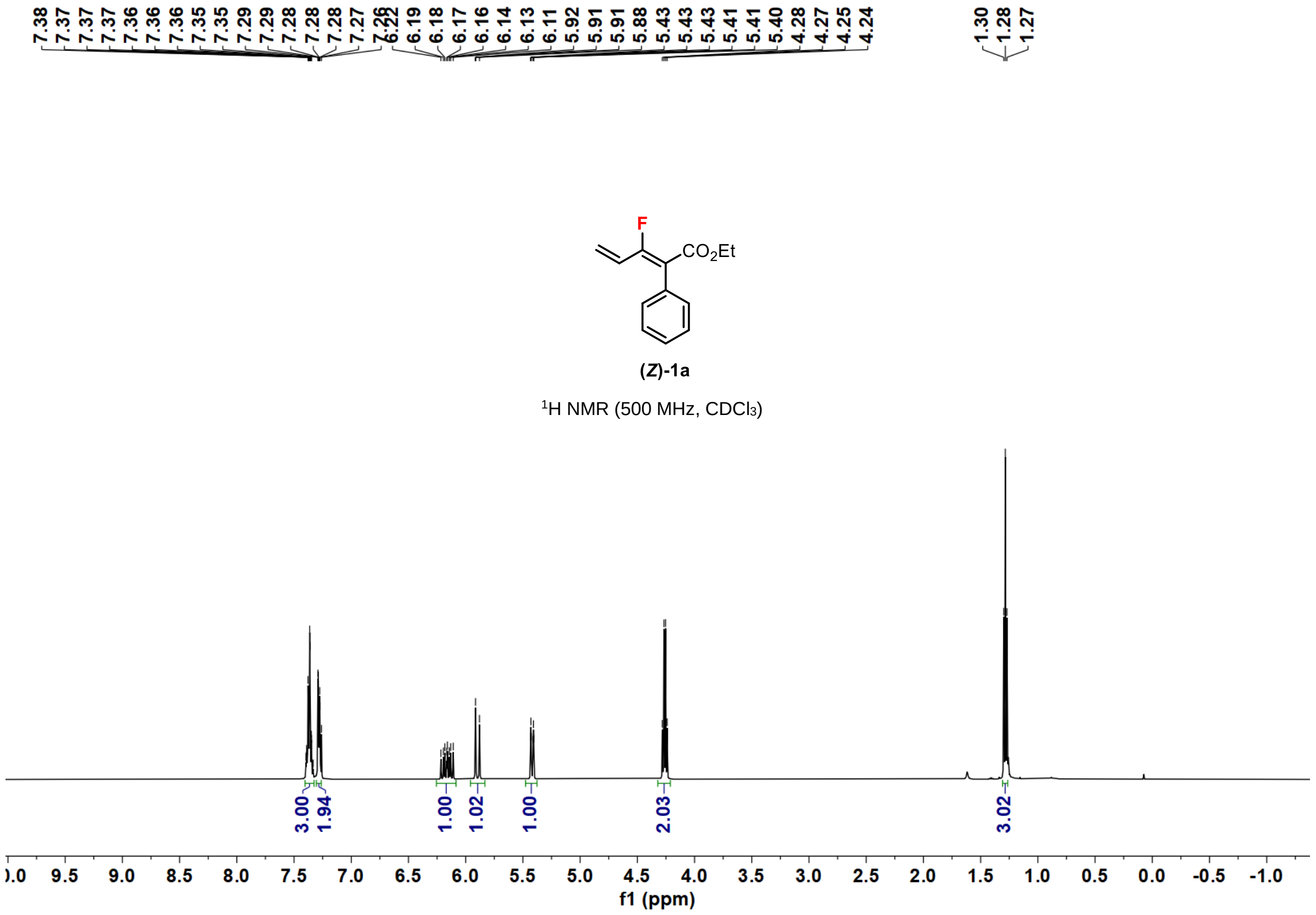

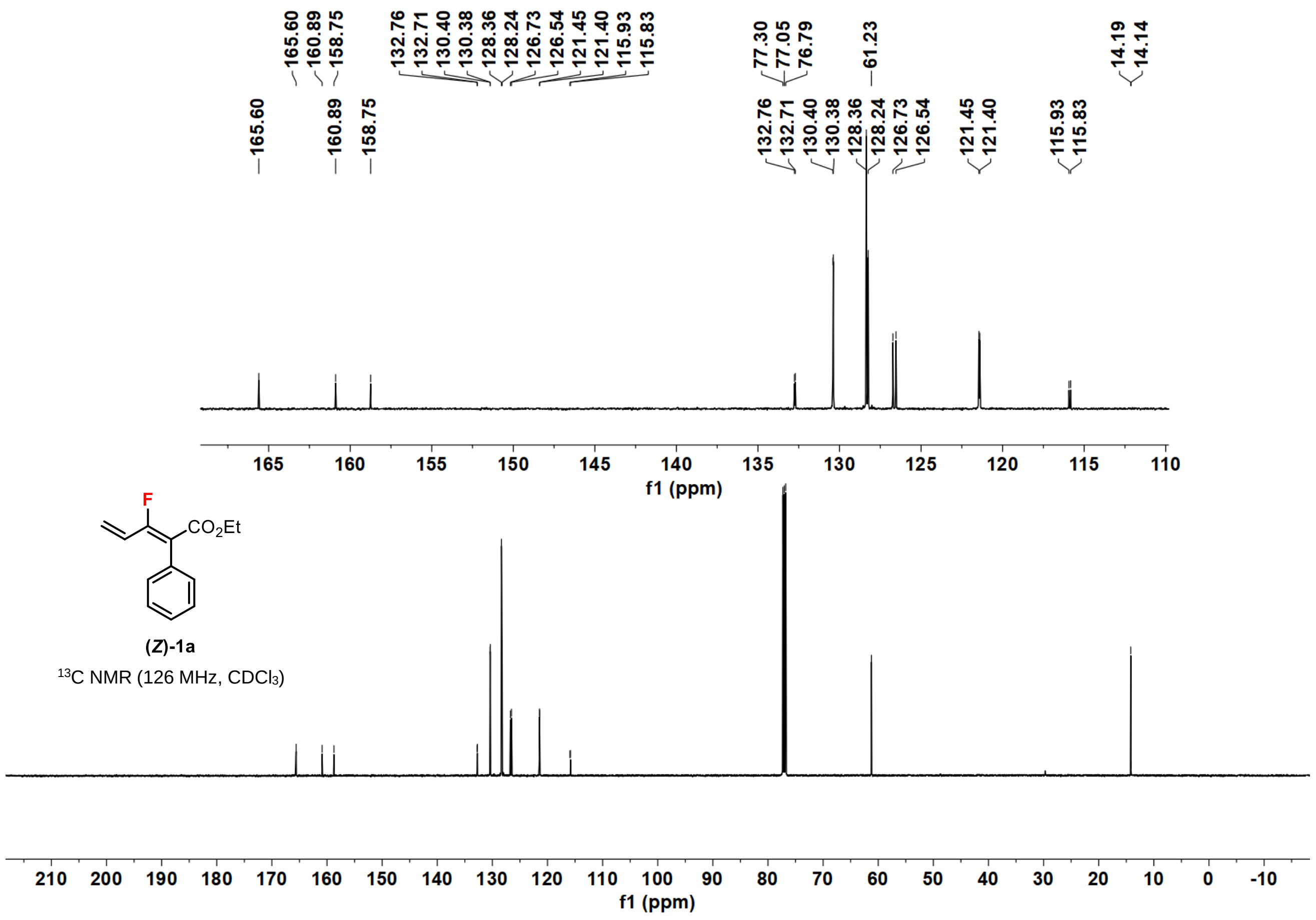

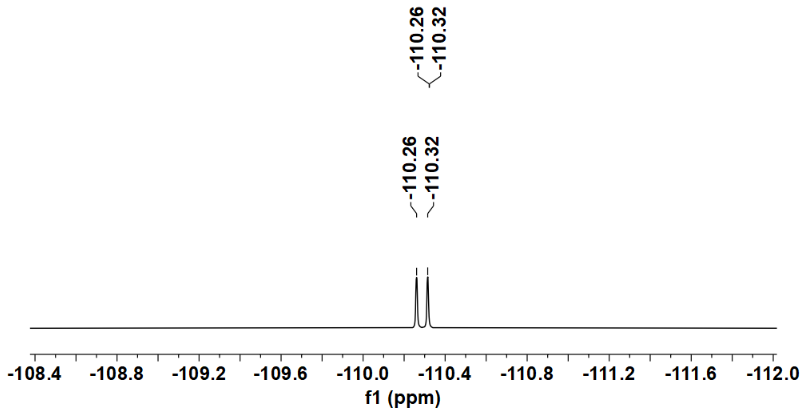

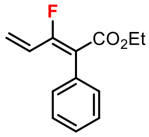

(*Z*)-1a

^{19}F NMR (471MHz, $CDCl_3$)

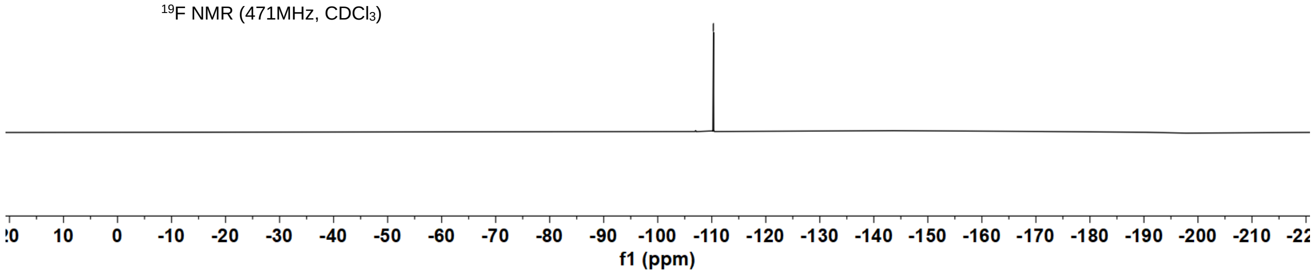

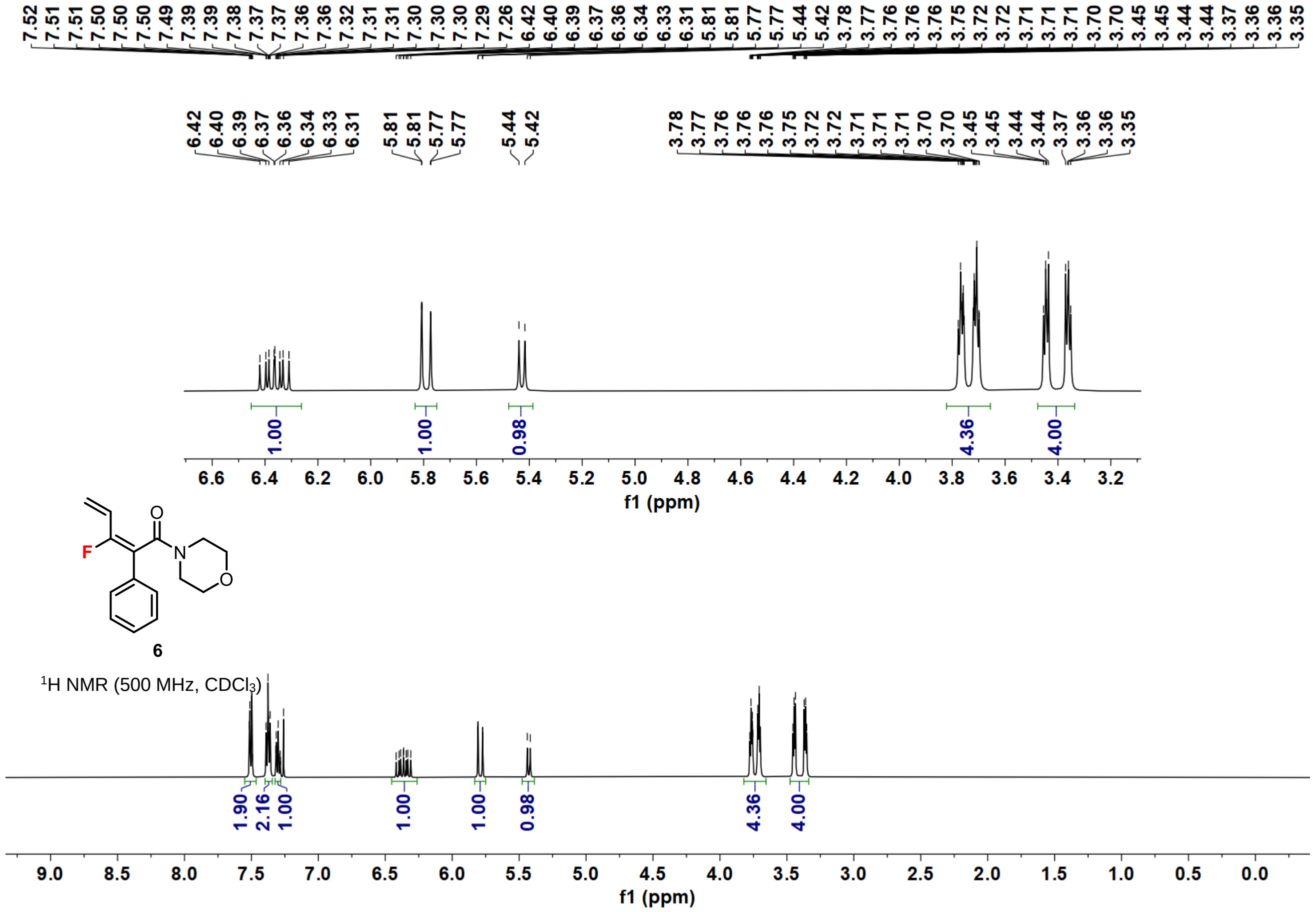

6

^{1}H NMR (500 MHz, $CDCl_3$)

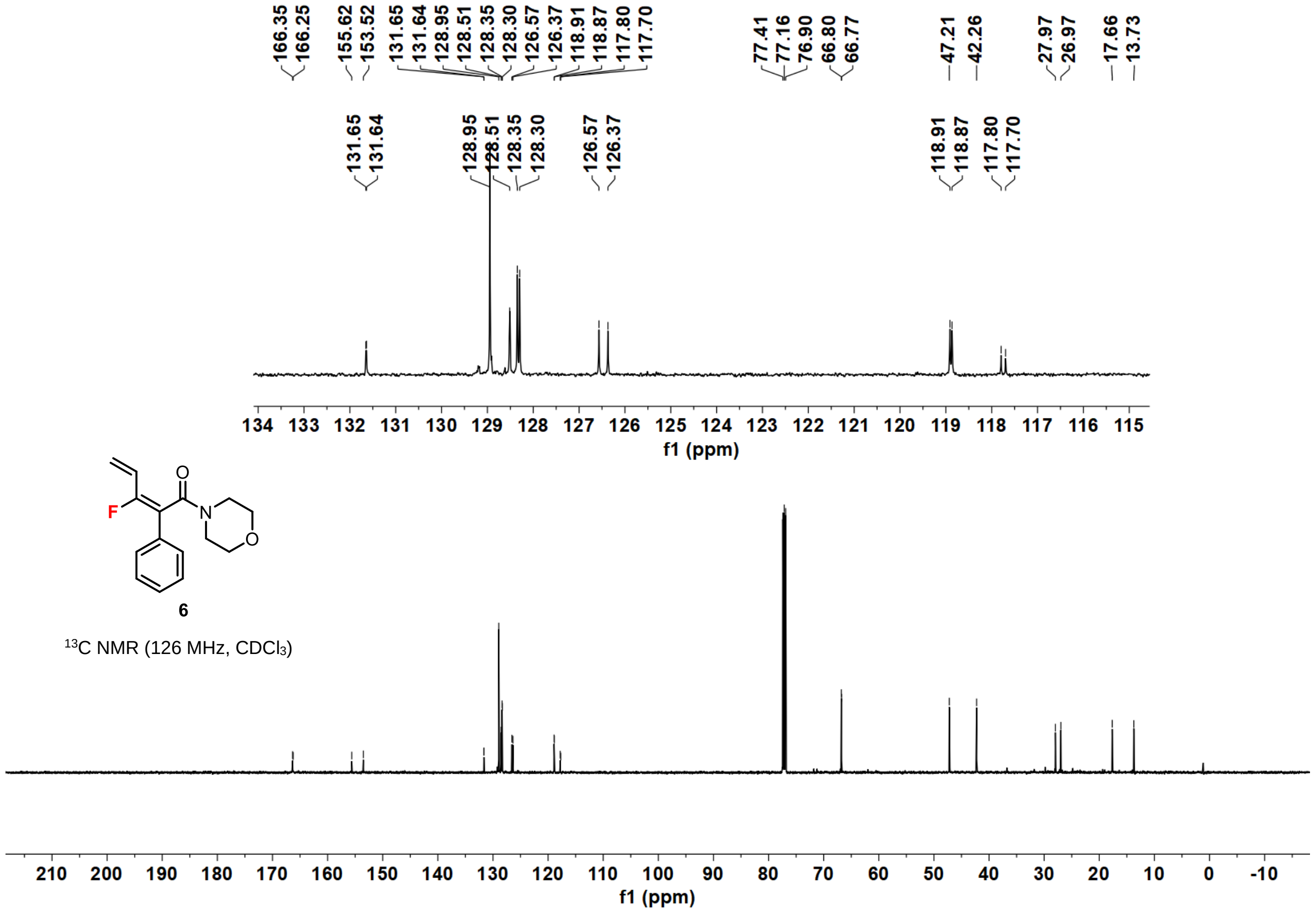

6

^{13}C NMR (126 MHz, $CDCl_3$)

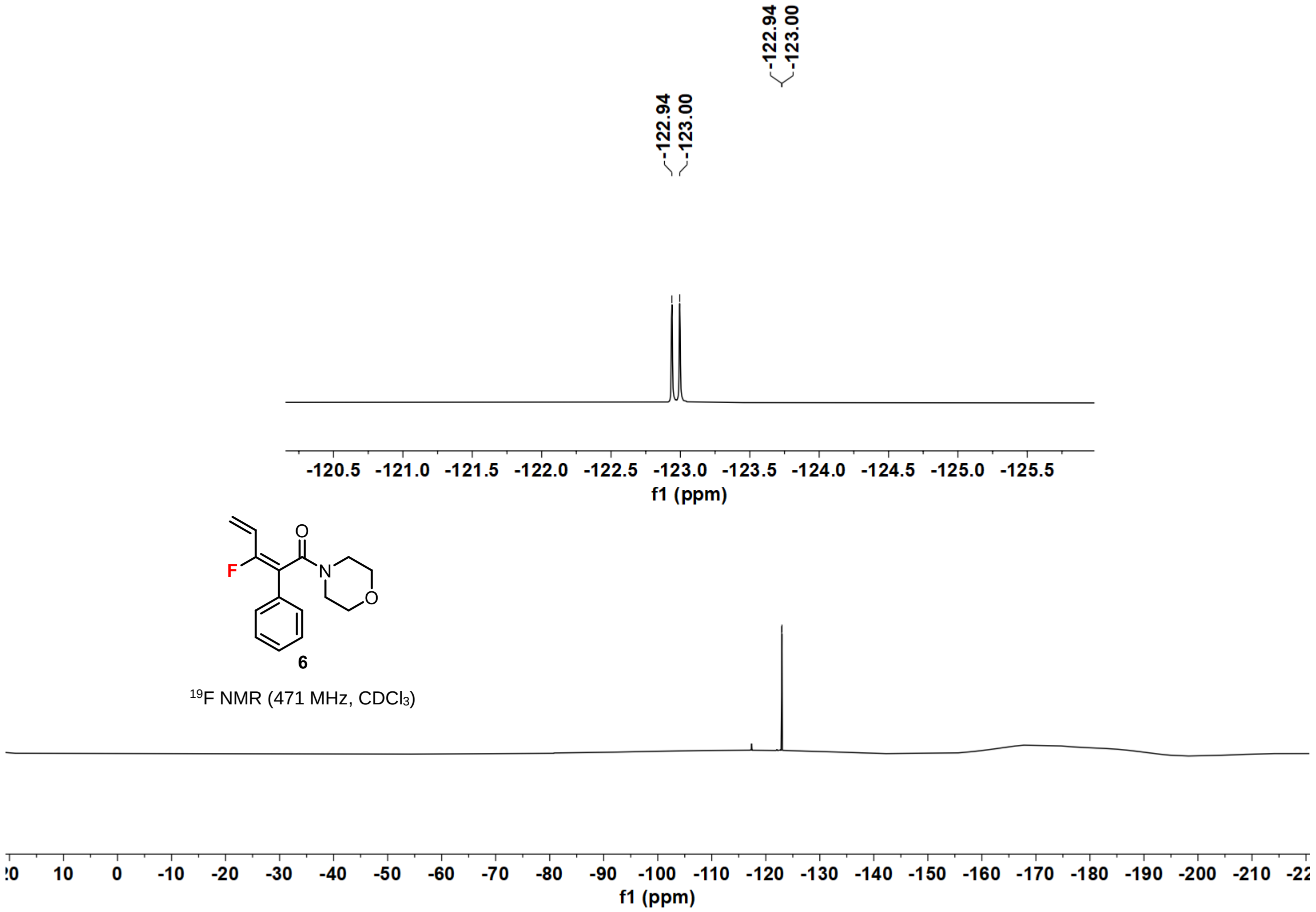

6

^{19}F NMR (471 MHz, $CDCl_3$)

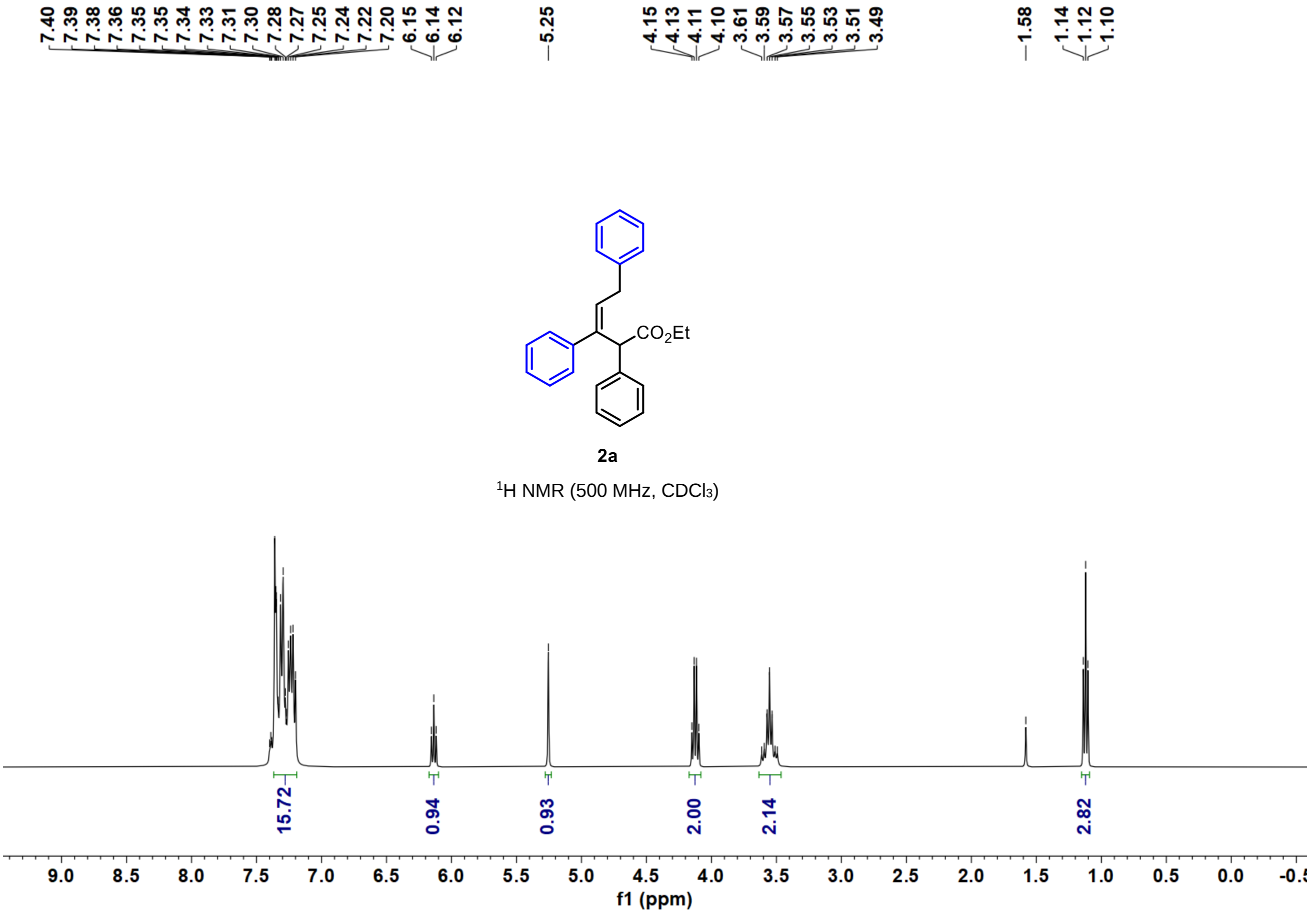

2a

^{1}H NMR (500 MHz, CDCl3)

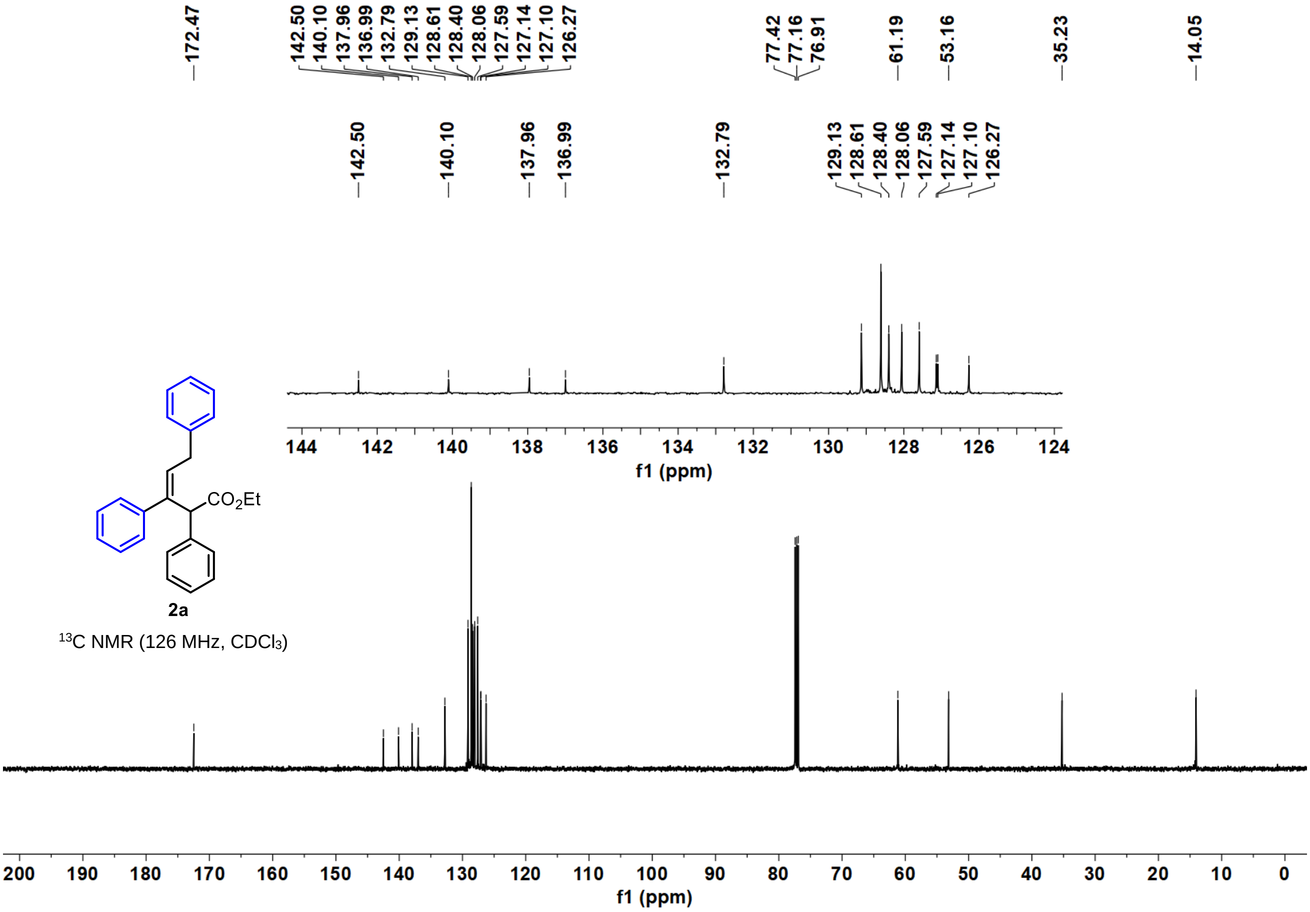

2a

^{13}C NMR (126 MHz, CDCl3)

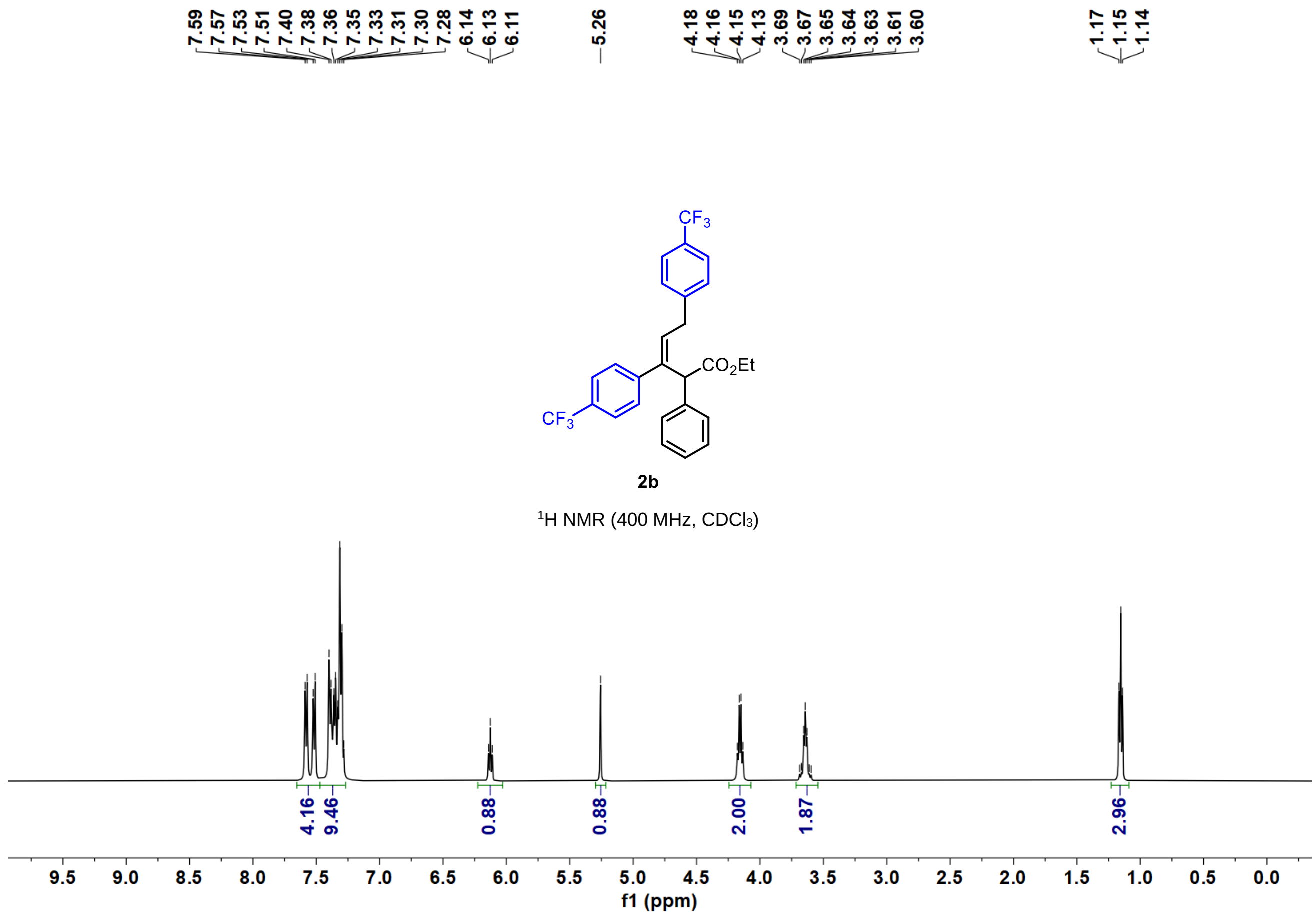

2b

^{1}H NMR (400 MHz, $CDCl_3$)

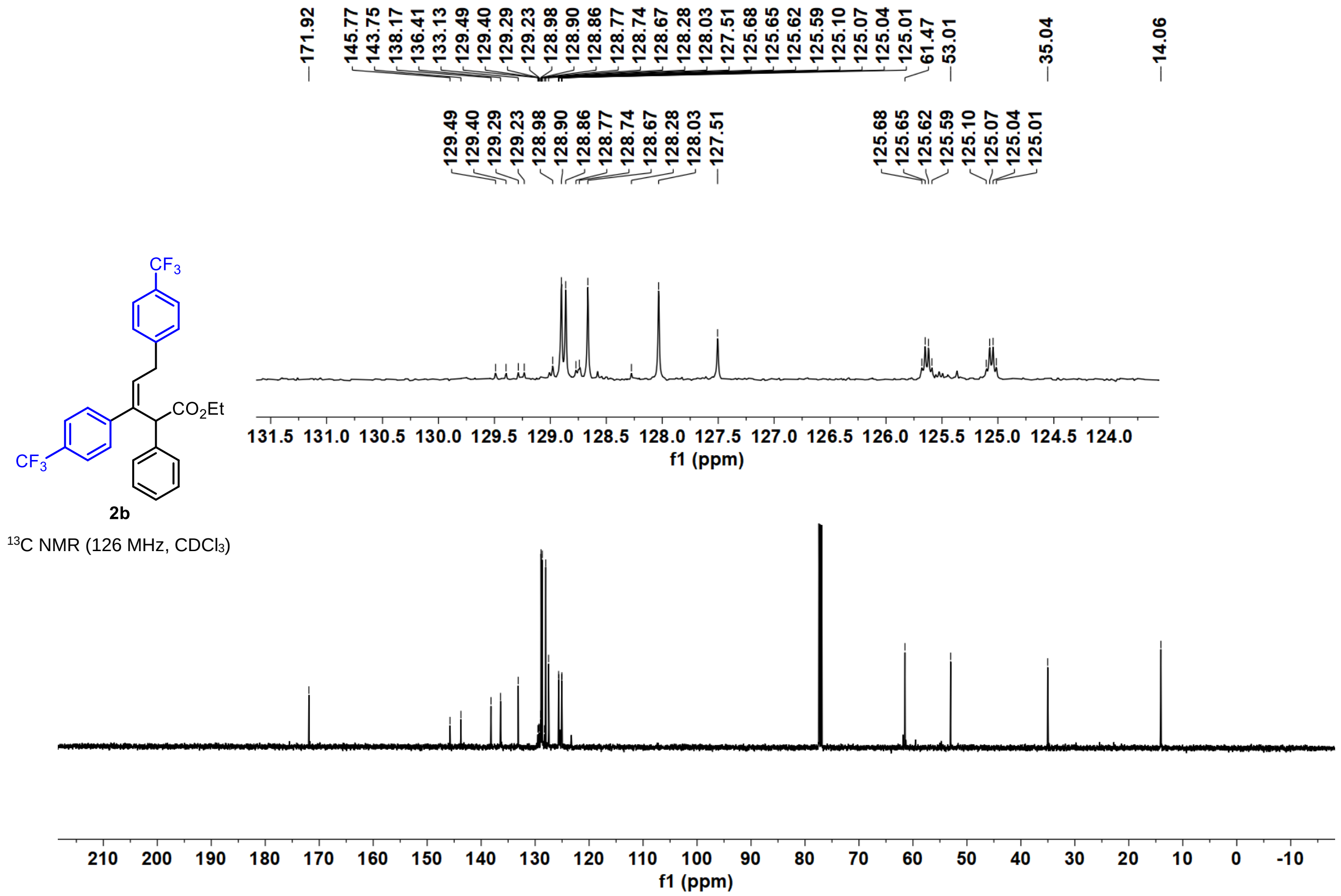

2b

^{13}C NMR (126 MHz, $CDCl_3$)

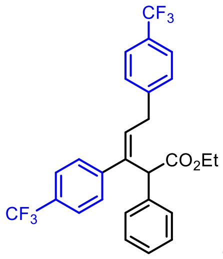

2b

^{19}F NMR (377 MHz, $CDCl_3$)

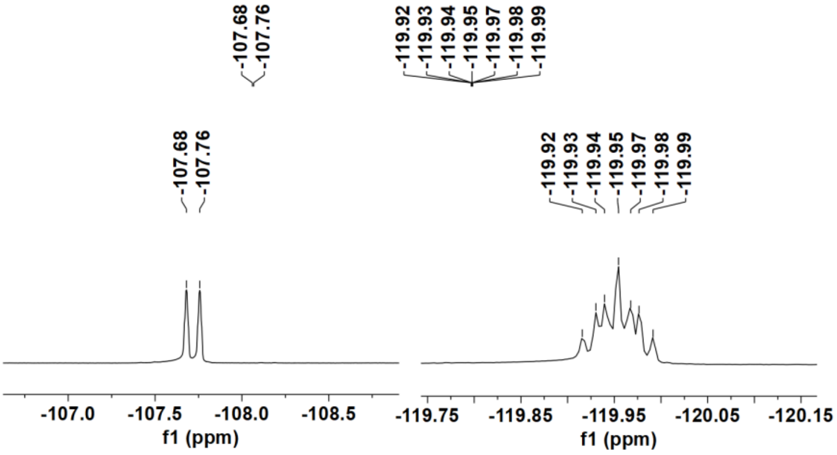

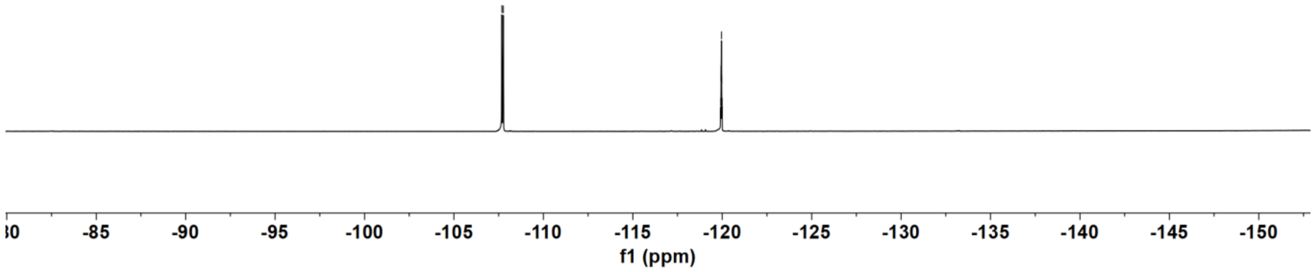

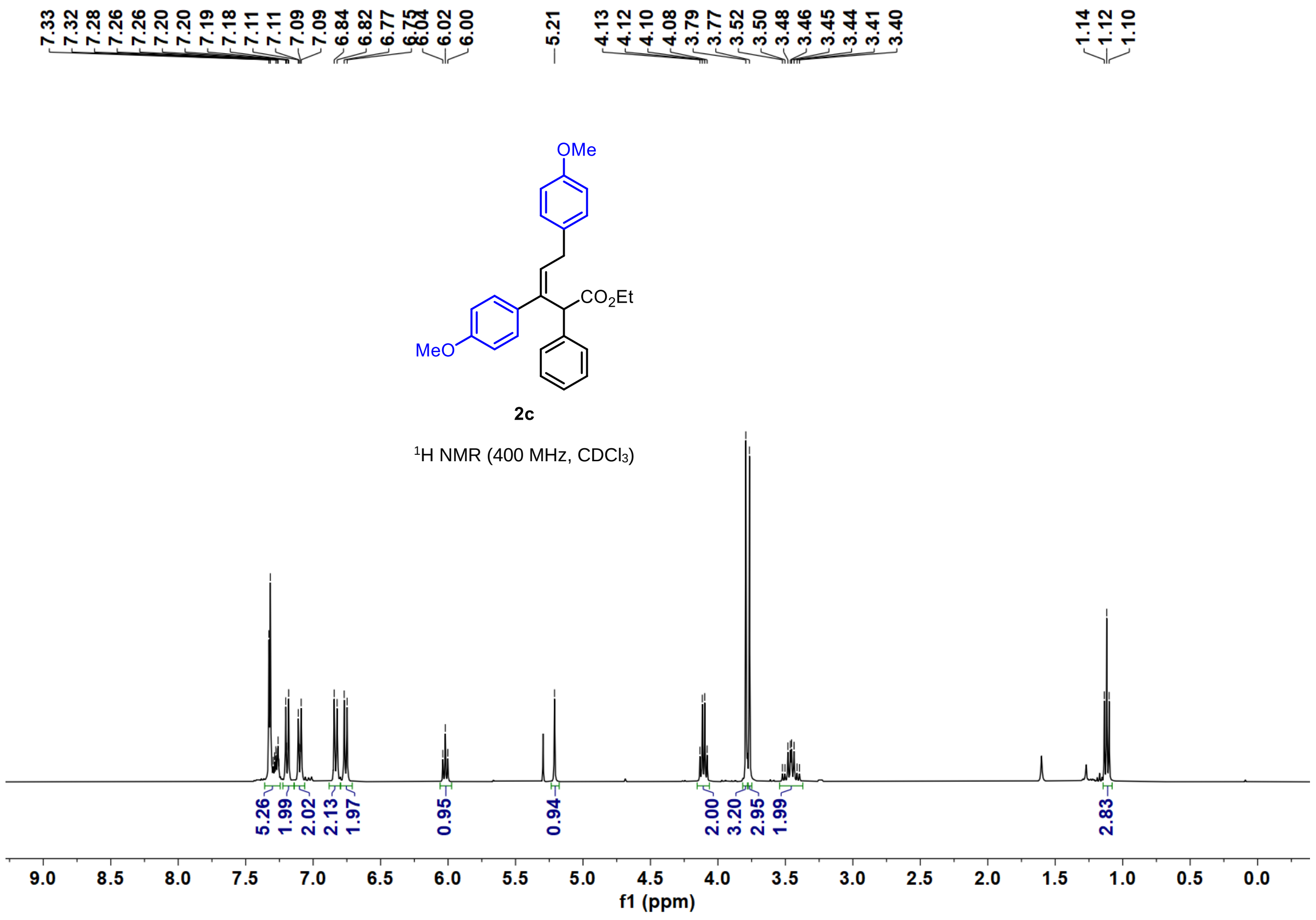
7.33 7.32 7.28 7.26 7.26 7.20 7.20 7.19 7.18 7.11 7.11 7.09 7.09 6.84 6.82 6.77 6.75 6.04 6.02 6.00
5.21
4.13 4.12 4.10 4.08 3.79 3.77 3.52 3.50 3.48 3.46 3.45 3.44 3.41 3.40
1.14 1.12 1.10
OMe
CO_2Et
MeO
2c
^{1}H NMR (400 MHz, $CDCl_3$)
5.26 1.99 2.02 2.13 1.97 0.95 0.94 2.00 3.20 2.95 1.99 2.83
9.0 8.5 8.0 7.5 7.0 6.5 6.0 5.5 5.0 4.5 4.0 3.5 3.0 2.5 2.0 1.5 1.0 0.5 0.0
f1 (ppm)

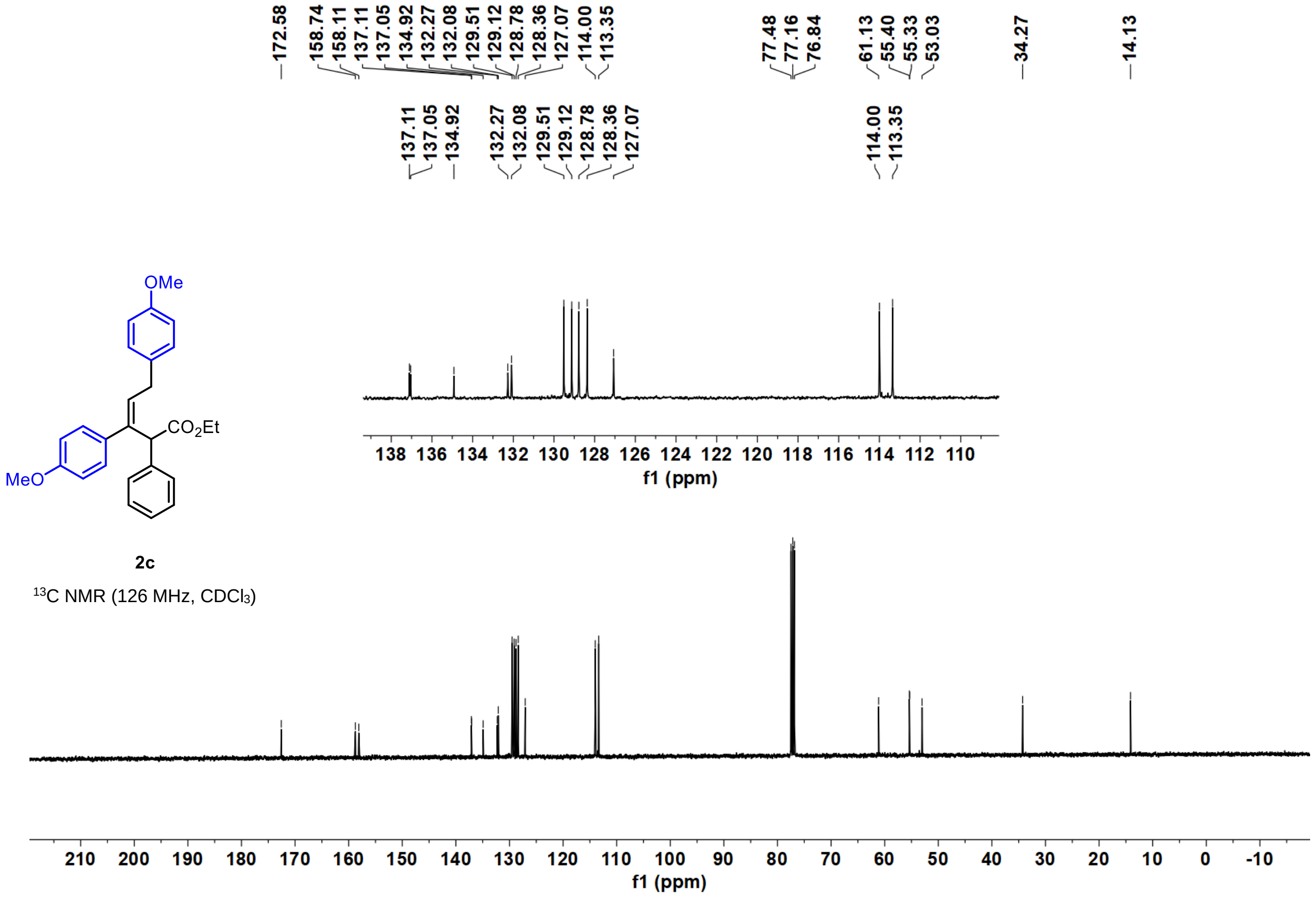
172.58
158.74 158.11 137.11 137.05 134.92 132.27 132.08 129.51 129.12 128.78 128.36 127.07 114.00 113.35
77.48 77.16 76.84
61.13 55.40 55.33 53.03
34.27
14.13
137.11 137.05 134.92 132.27 132.08 129.51 129.12 128.78 128.36 127.07
114.00 113.35
138 136 134 132 130 128 126 124 122 120 118 116 114 112 110
f1 (ppm)
OMe
CO_2Et
MeO
2c
^{13}C NMR (126 MHz, $CDCl_3$)
210 200 190 180 170 160 150 140 130 120 110 100 90 80 70 60 50 40 30 20 10 0 -10
f1 (ppm)

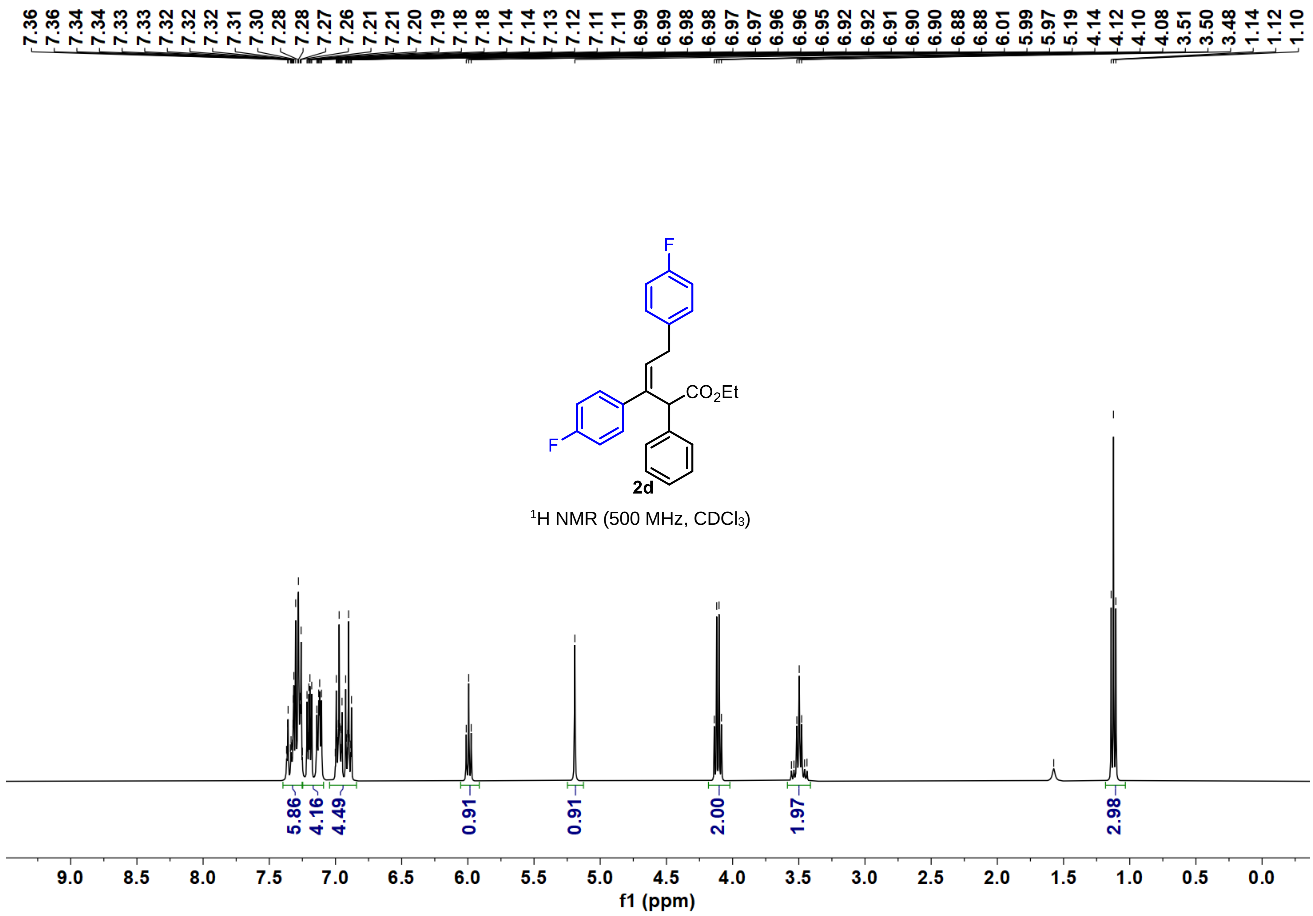
2d
^{1}H NMR (500 MHz, CDCl3)
f1 (ppm)

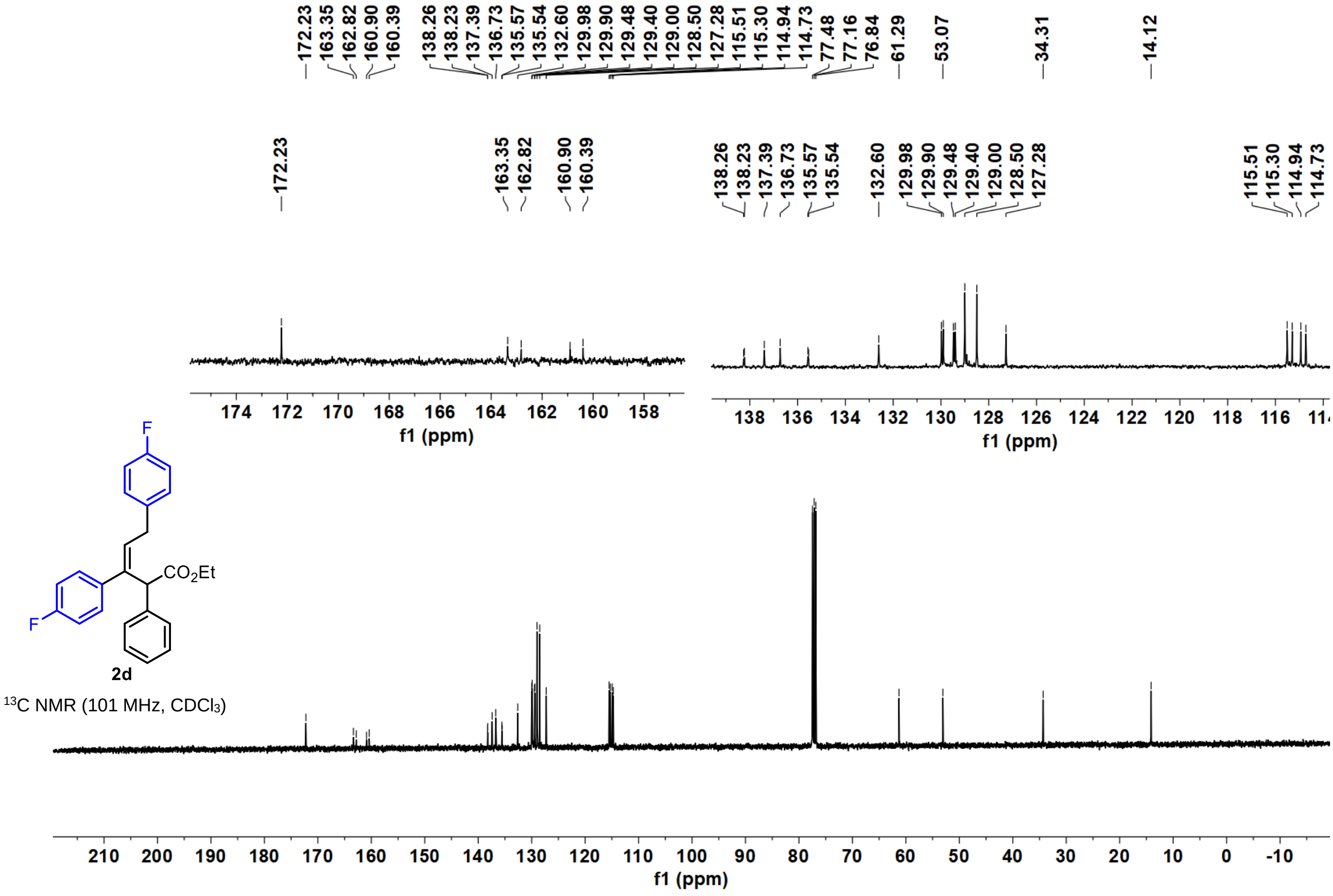
2d
^{13}C NMR (101 MHz, CDCl3)
f1 (ppm)

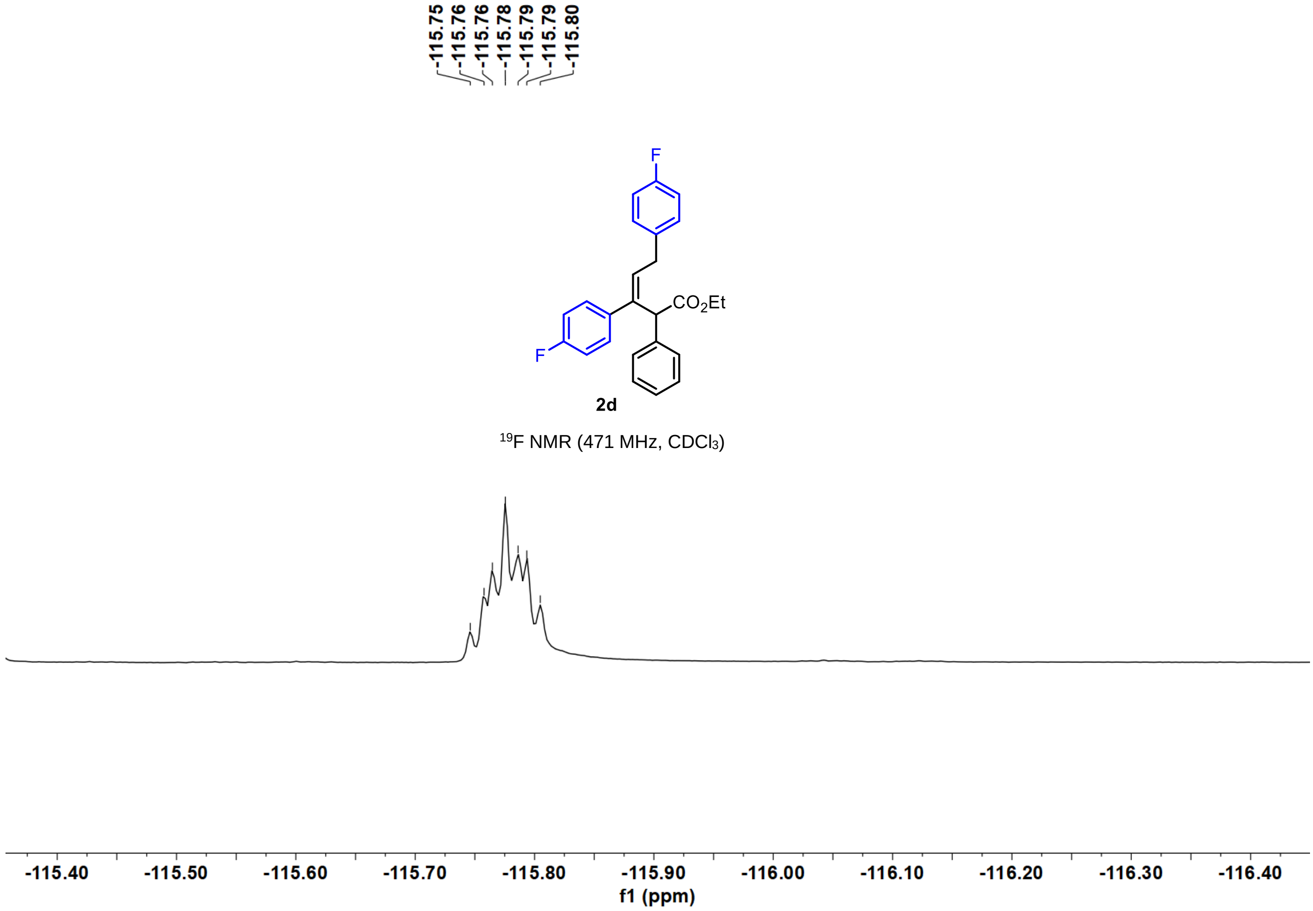
-115.75
-115.76
-115.76
-115.78
-115.79
-115.79
-115.80
F
CO2Et
F
2d
19F NMR (471 MHz, CDCl3)
-115.40
-115.50
-115.60
-115.70
-115.80
-115.90
-116.00
-116.10
-116.20
-116.30
-116.40
f1 (ppm)

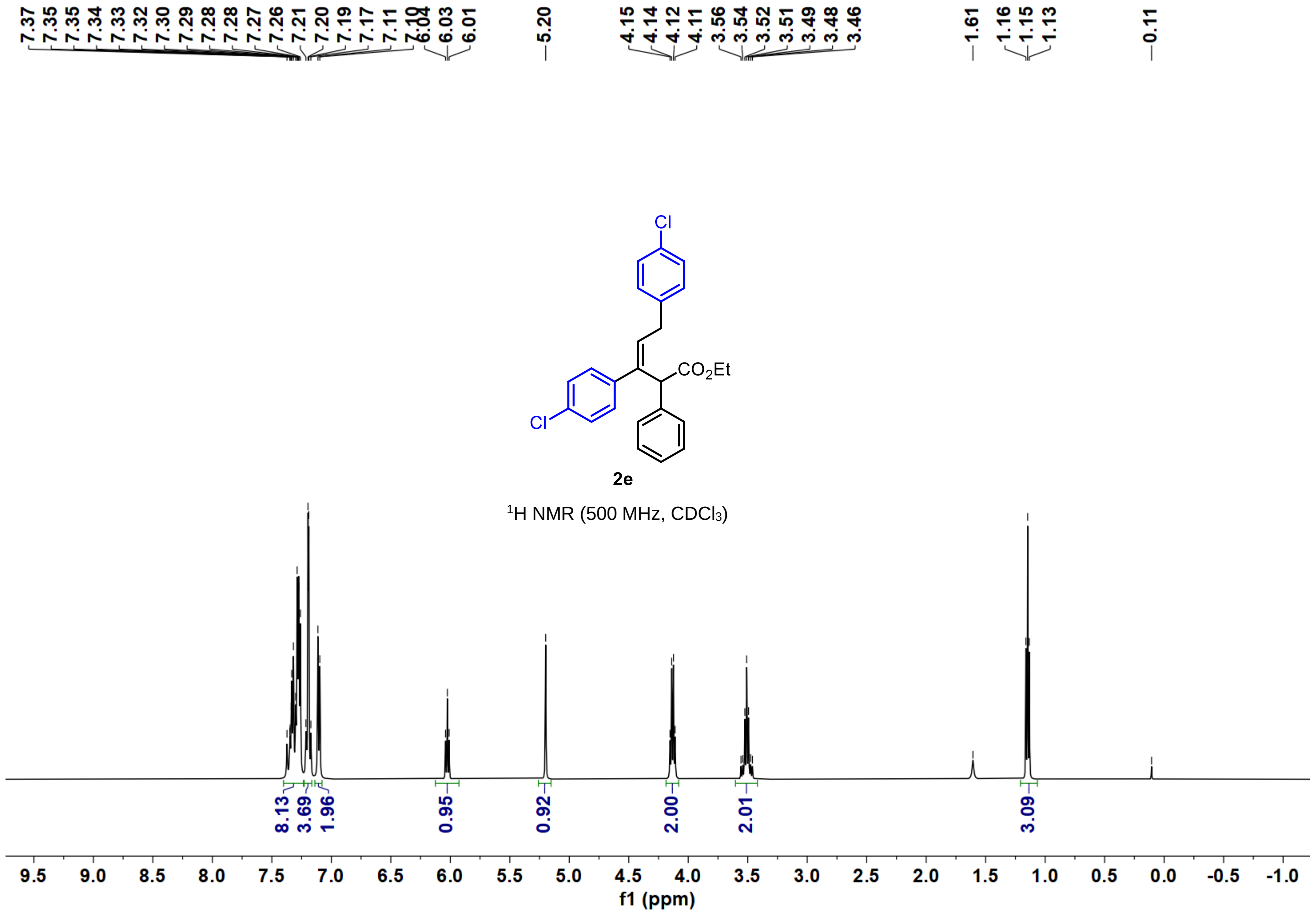
7.37
7.35
7.35
7.34
7.33
7.32
7.30
7.29
7.28
7.28
7.27
7.26
7.21
7.20
7.19
7.17
7.11
7.10
6.04
6.03
6.01
5.20
4.15
4.14
4.12
4.11
3.56
3.54
3.52
3.51
3.49
3.48
3.46
1.61
1.16
1.15
1.13
0.11
Cl
CO2Et
Cl
2e
1H NMR (500 MHz, CDCl3)
8.13
3.69
1.96
0.95
0.92
2.00
2.01
3.09
f1 (ppm)

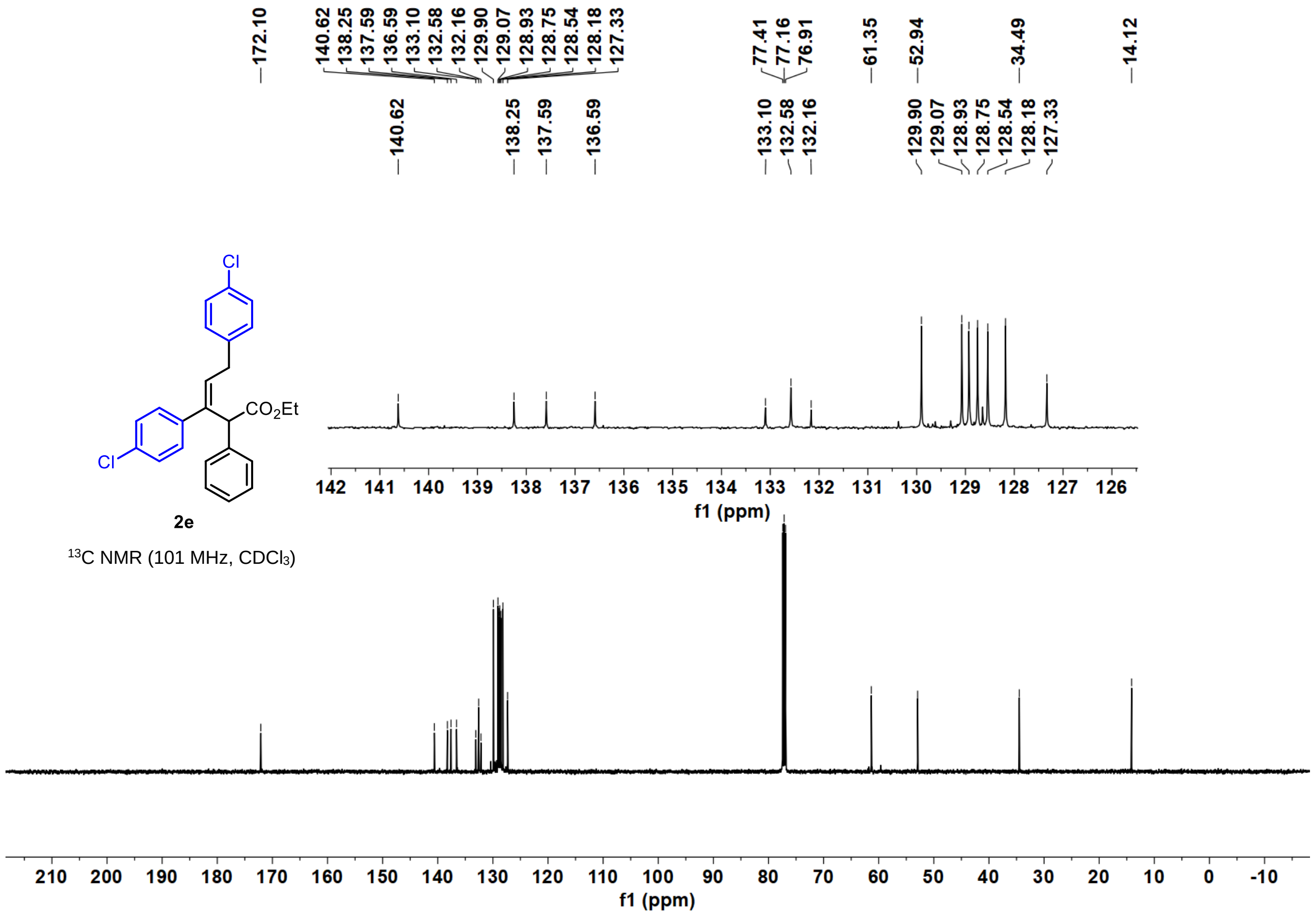
172.10
140.62
138.25
137.59
136.59
133.10
132.58
132.16
129.90
129.07
128.93
128.75
128.54
128.18
127.33
77.41
77.16
76.91
61.35
52.94
34.49
14.12
Cl
CO2Et
Cl
2e
13C NMR (101 MHz, CDCl3)
f1 (ppm)
f1 (ppm)

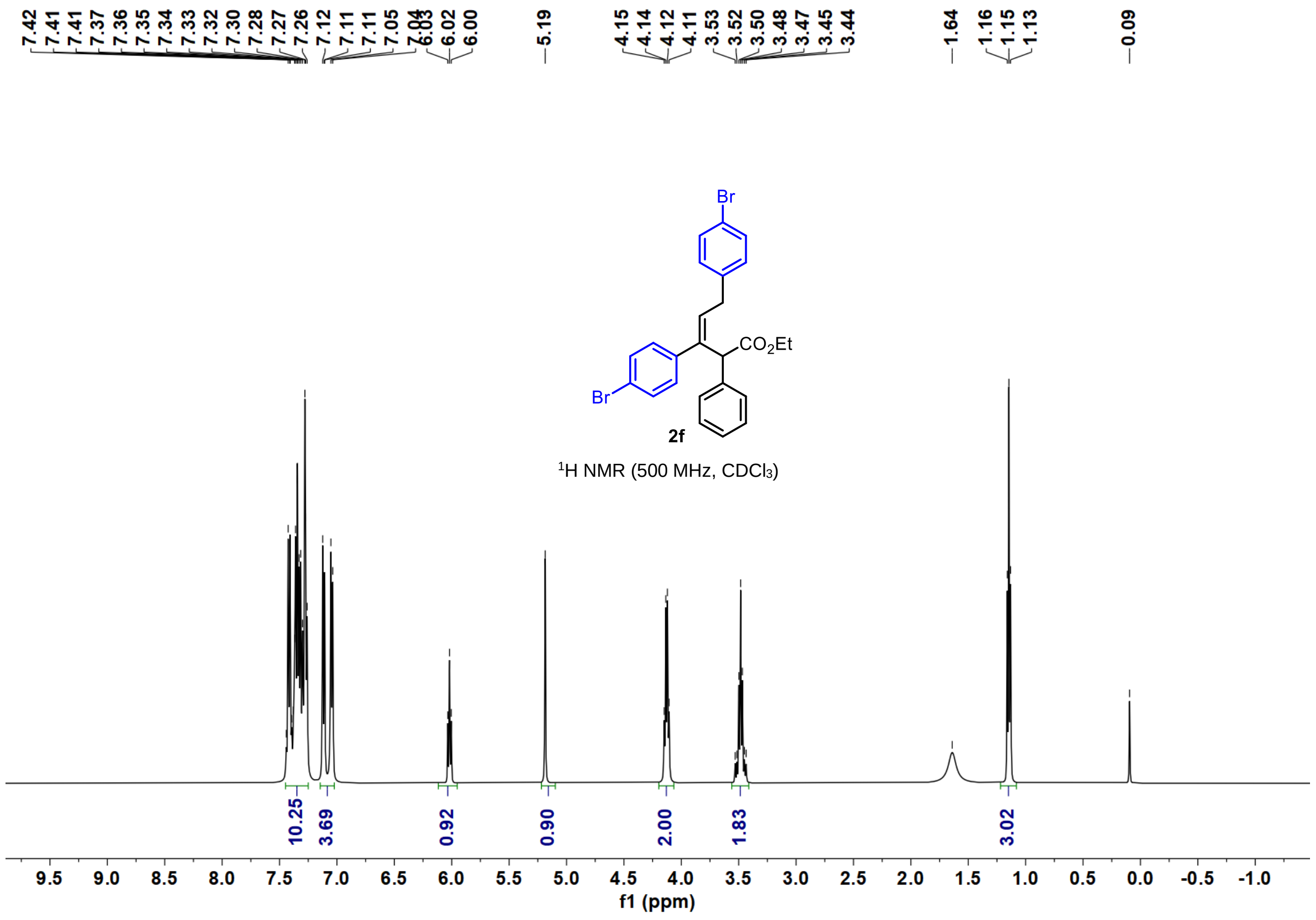
7.42
7.41
7.41
7.37
7.36
7.35
7.34
7.33
7.32
7.30
7.28
7.27
7.26
7.12
7.11
7.11
7.05
7.04
6.03
6.02
6.00
5.19
4.15
4.14
4.12
4.11
3.53
3.52
3.50
3.48
3.47
3.45
3.44
1.64
1.16
1.15
1.13
0.09
Br
CO2Et
Br
2f
1H NMR (500 MHz, CDCl3)
10.25
3.69
0.92
0.90
2.00
1.83
3.02
f1 (ppm)

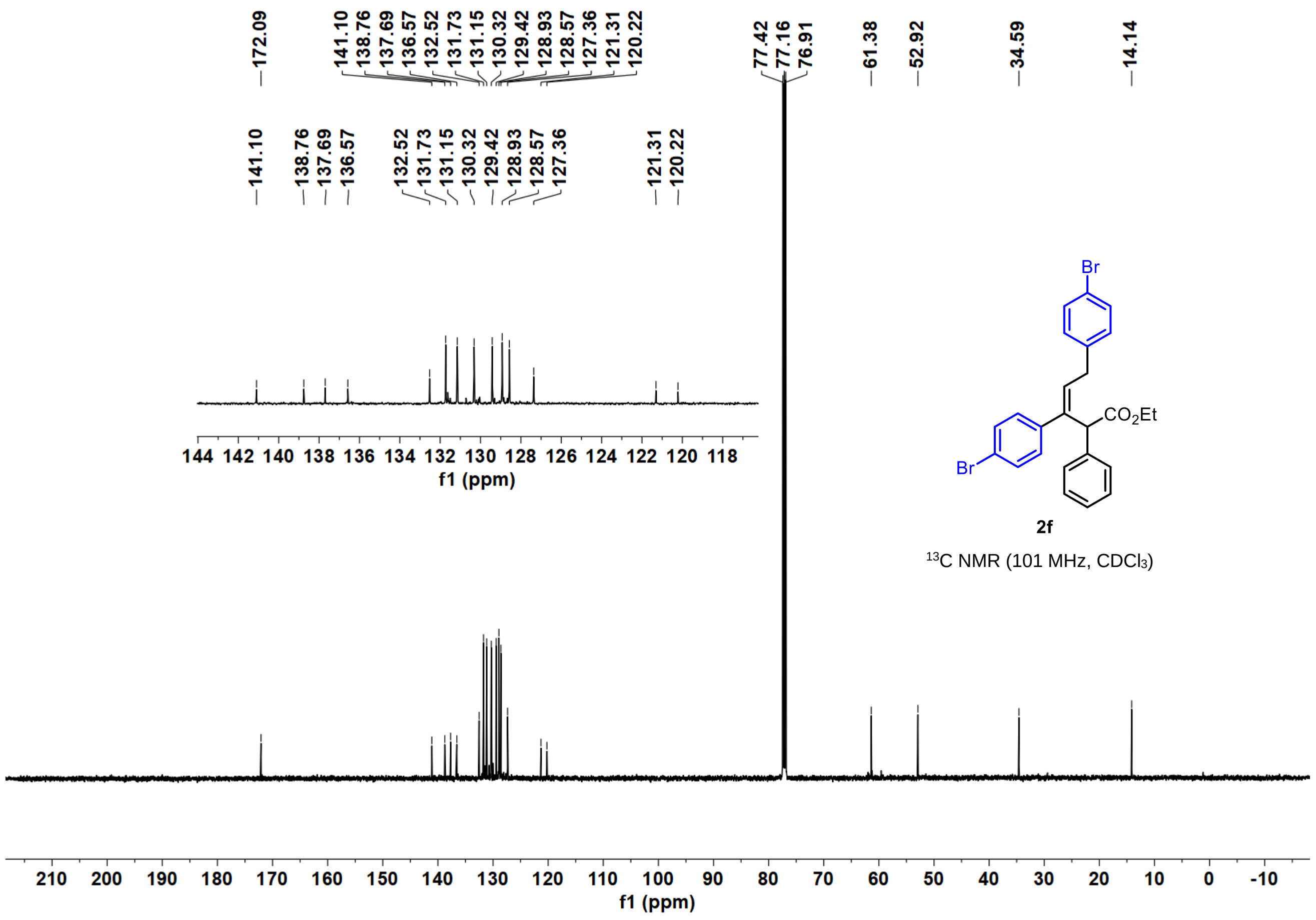
172.09
141.10
138.76
137.69
136.57
132.52
131.73
131.15
130.32
129.42
128.93
128.57
127.36
121.31
120.22
77.42
77.16
76.91
61.38
52.92
34.59
14.14
f1 (ppm)
Br
CO2Et
Br
2f
13C NMR (101 MHz, CDCl3)
f1 (ppm)

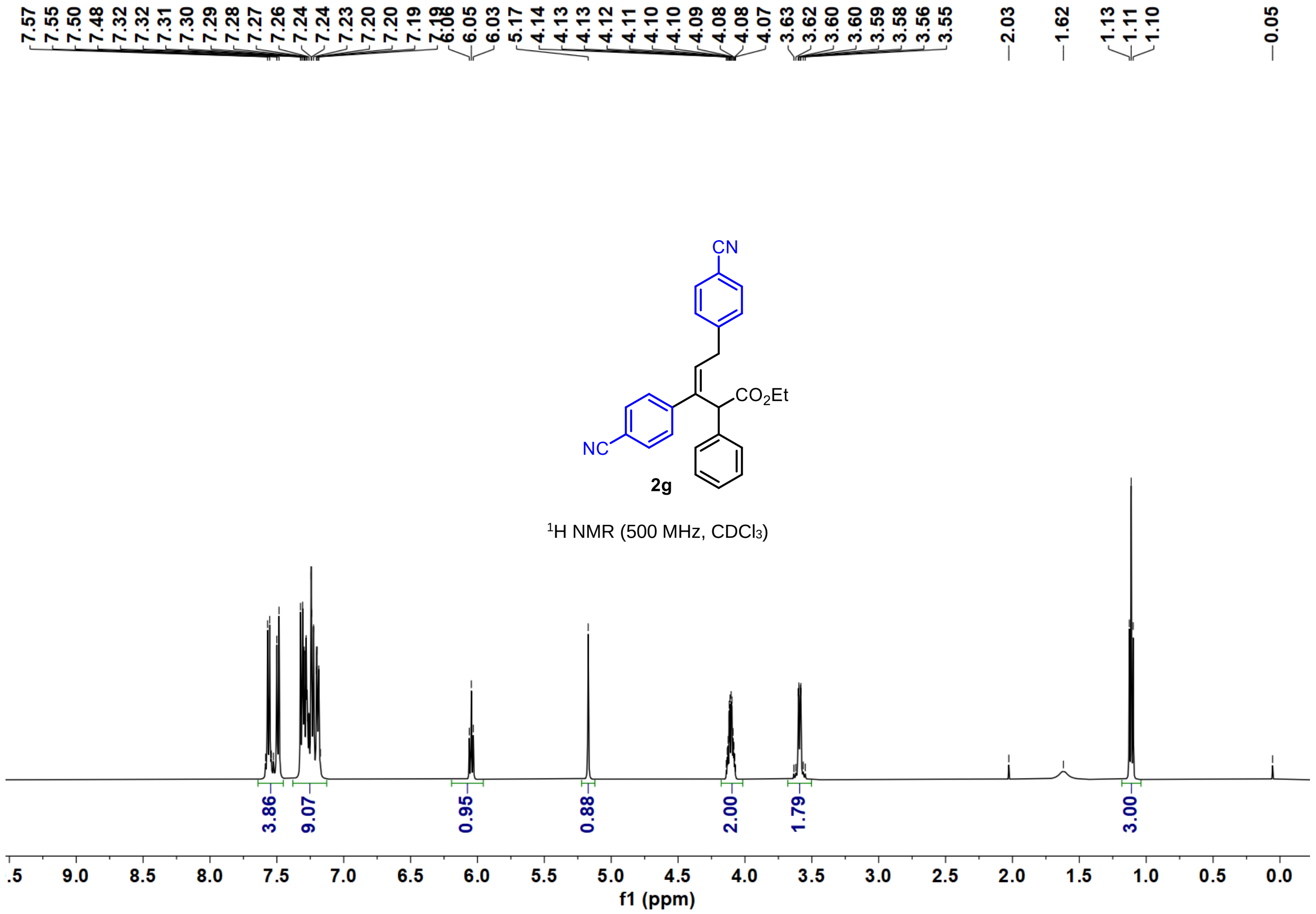

2g

^1H NMR (500 MHz, $CDCl_3$)

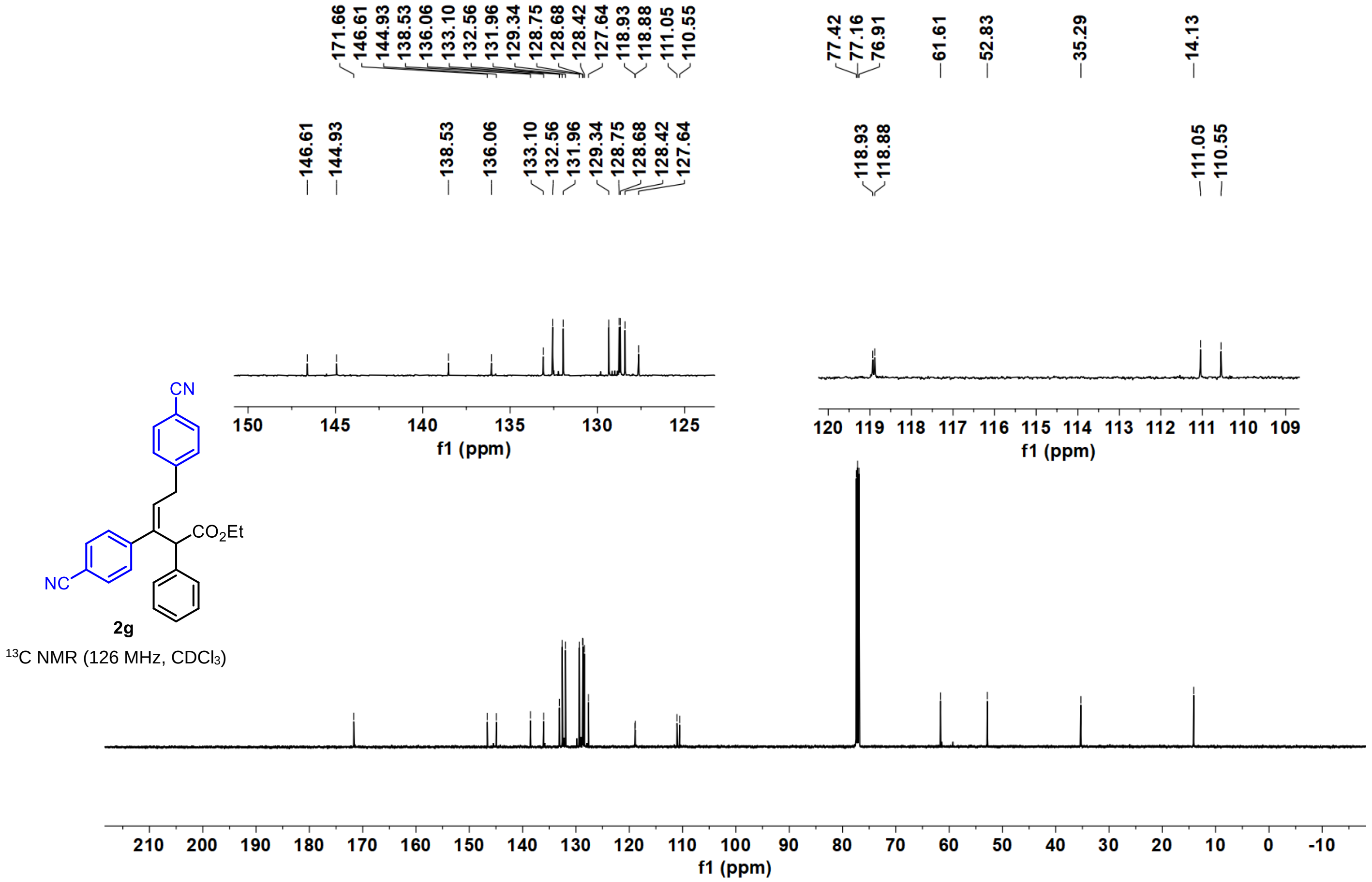

2g

^{13}C NMR (126 MHz, $CDCl_3$)

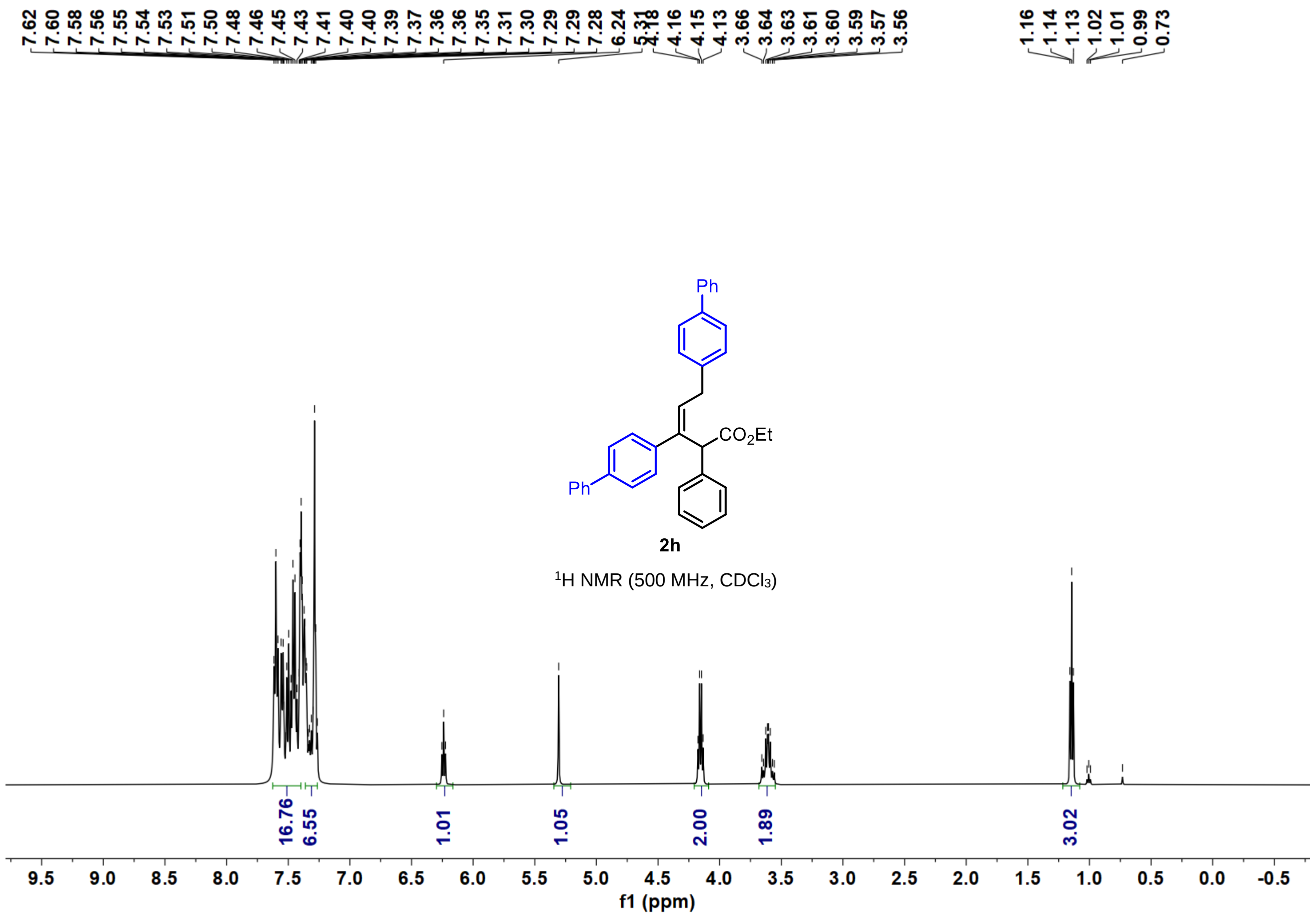
7.62 7.60 7.58 7.56 7.55 7.54 7.53 7.51 7.50 7.48 7.46 7.45 7.43 7.41 7.40 7.40 7.39 7.37 7.36 7.36 7.35 7.31 7.30 7.29 7.29 7.28 6.24 5.31 4.18 4.16 4.15 4.13 3.66 3.64 3.63 3.61 3.60 3.59 3.57 3.56 1.16 1.14 1.13 1.02 1.01 0.99 0.73
Ph
CO2Et
Ph
2h
1H NMR (500 MHz, CDCl3)
16.76 6.55 1.01 1.05 2.00 1.89 3.02
9.5 9.0 8.5 8.0 7.5 7.0 6.5 6.0 5.5 5.0 4.5 4.0 3.5 3.0 2.5 2.0 1.5 1.0 0.5 0.0 -0.5
f1 (ppm)

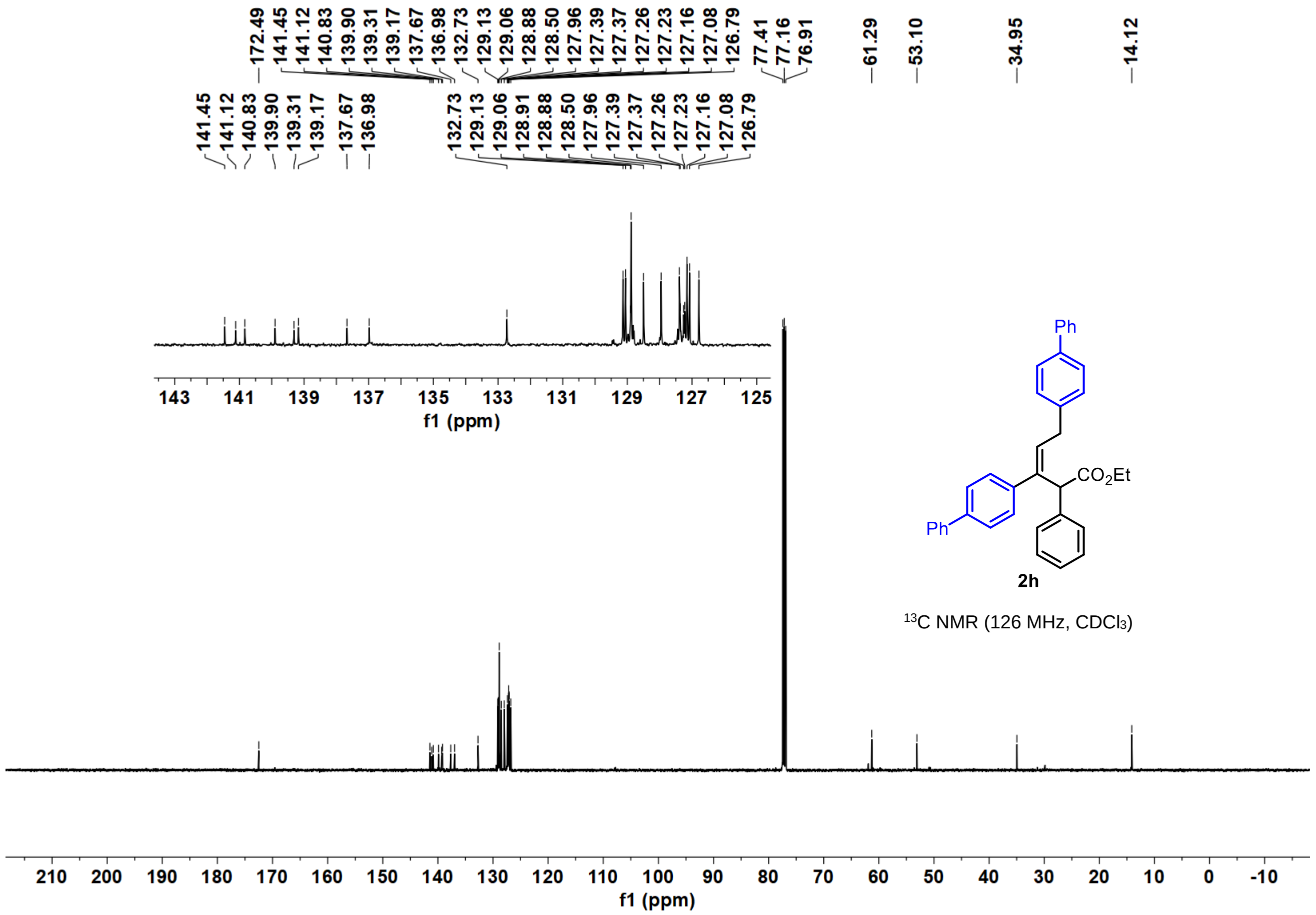
172.49 141.45 141.12 140.83 139.90 139.31 139.17 137.67 136.98 132.73 129.13 129.06 128.88 128.50 127.96 127.39 127.37 127.26 127.23 127.16 127.08 126.79 77.41 77.16 76.91 61.29 53.10 34.95 14.12
141.45 141.12 140.83 139.90 139.31 139.17 137.67 136.98 132.73 129.13 129.06 128.91 128.88 128.50 127.96 127.39 127.37 127.26 127.23 127.16 127.08 126.79
143 141 139 137 135 133 131 129 127 125
f1 (ppm)
Ph
CO2Et
Ph
2h
13C NMR (126 MHz, CDCl3)
210 200 190 180 170 160 150 140 130 120 110 100 90 80 70 60 50 40 30 20 10 0 -10
f1 (ppm)

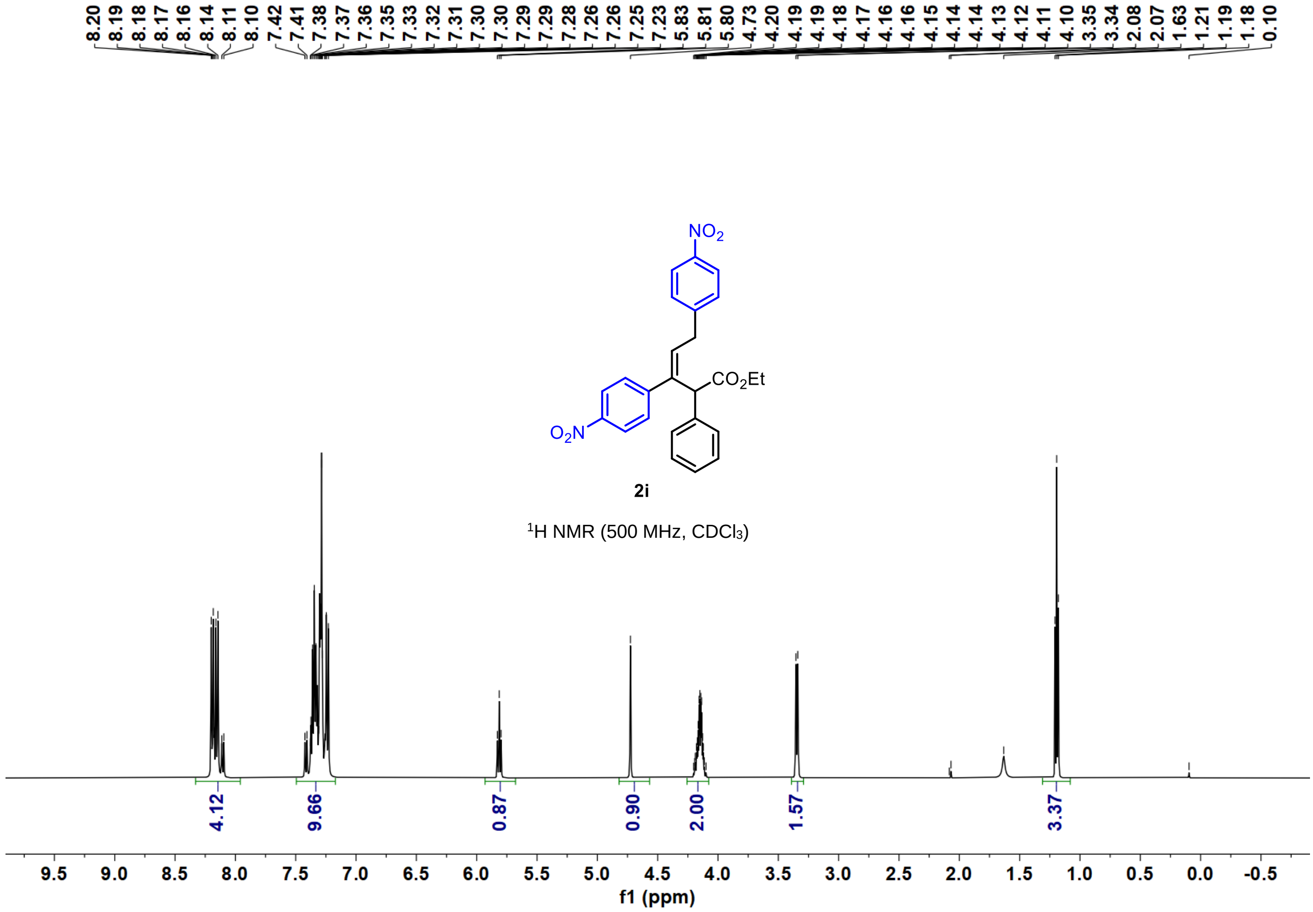
8.20 8.19 8.18 8.17 8.16 8.14 8.11 8.10 7.42 7.41 7.38 7.37 7.36 7.35 7.33 7.32 7.31 7.30 7.30 7.29 7.29 7.28 7.26 7.26 7.25 7.23 5.83 5.81 5.80 4.73 4.20 4.19 4.19 4.18 4.17 4.16 4.16 4.15 4.14 4.14 4.13 4.12 4.11 4.10 3.35 3.34 2.08 2.07 1.63 1.21 1.19 1.18 0.10
NO2
CO2Et
O2N
2i
1H NMR (500 MHz, CDCl3)
4.12 9.66 0.87 0.90 2.00 1.57 3.37
9.5 9.0 8.5 8.0 7.5 7.0 6.5 6.0 5.5 5.0 4.5 4.0 3.5 3.0 2.5 2.0 1.5 1.0 0.5 0.0 -0.5
f1 (ppm)

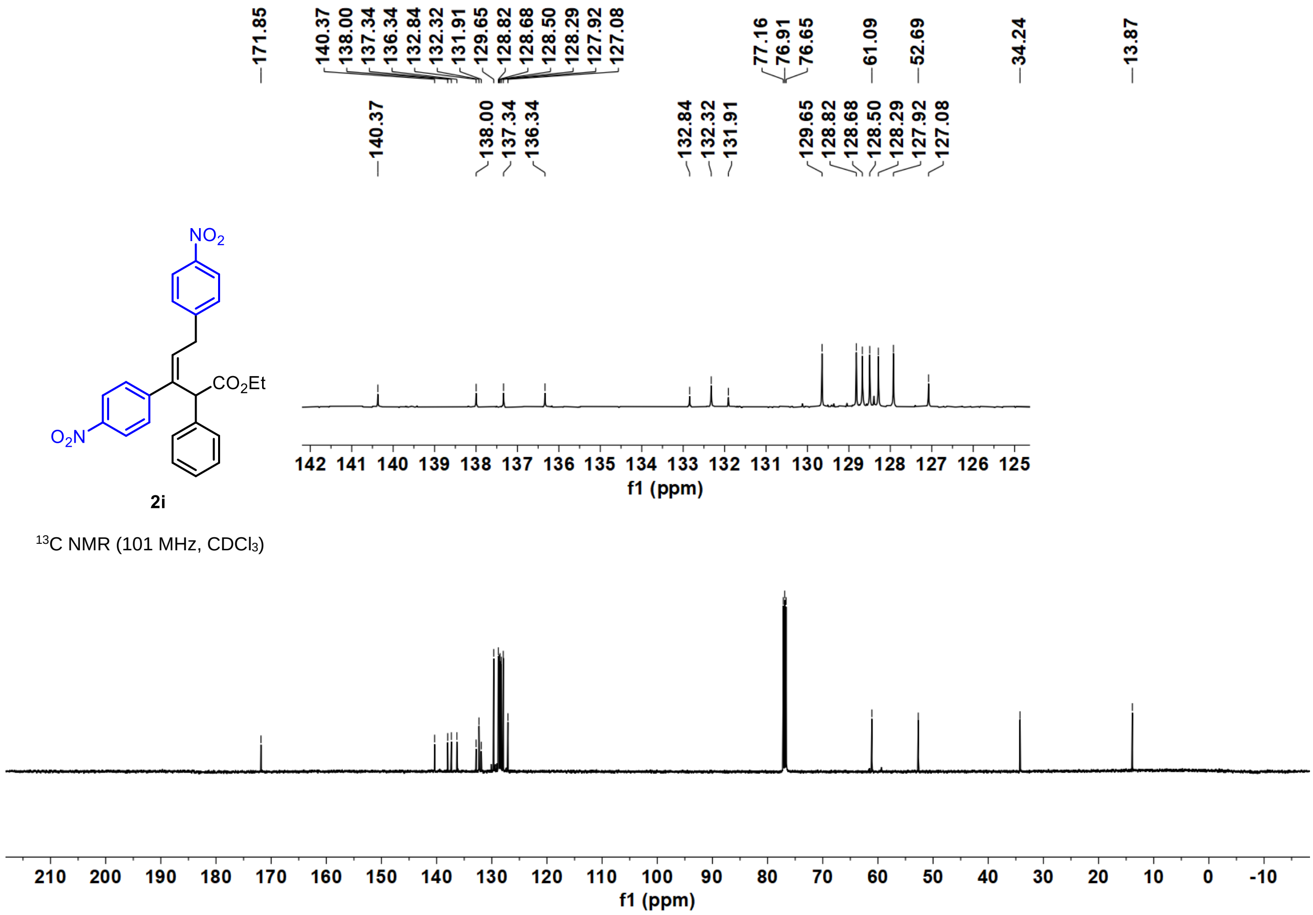
171.85 140.37 138.00 137.34 136.34 132.84 132.32 131.91 129.65 128.82 128.68 128.50 128.29 127.92 127.08 77.16 76.91 76.65 61.09 52.69 34.24 13.87
NO2
CO2Et
O2N
2i
13C NMR (101 MHz, CDCl3)
142 141 140 139 138 137 136 135 134 133 132 131 130 129 128 127 126 125
f1 (ppm)
210 200 190 180 170 160 150 140 130 120 110 100 90 80 70 60 50 40 30 20 10 0 -10
f1 (ppm)

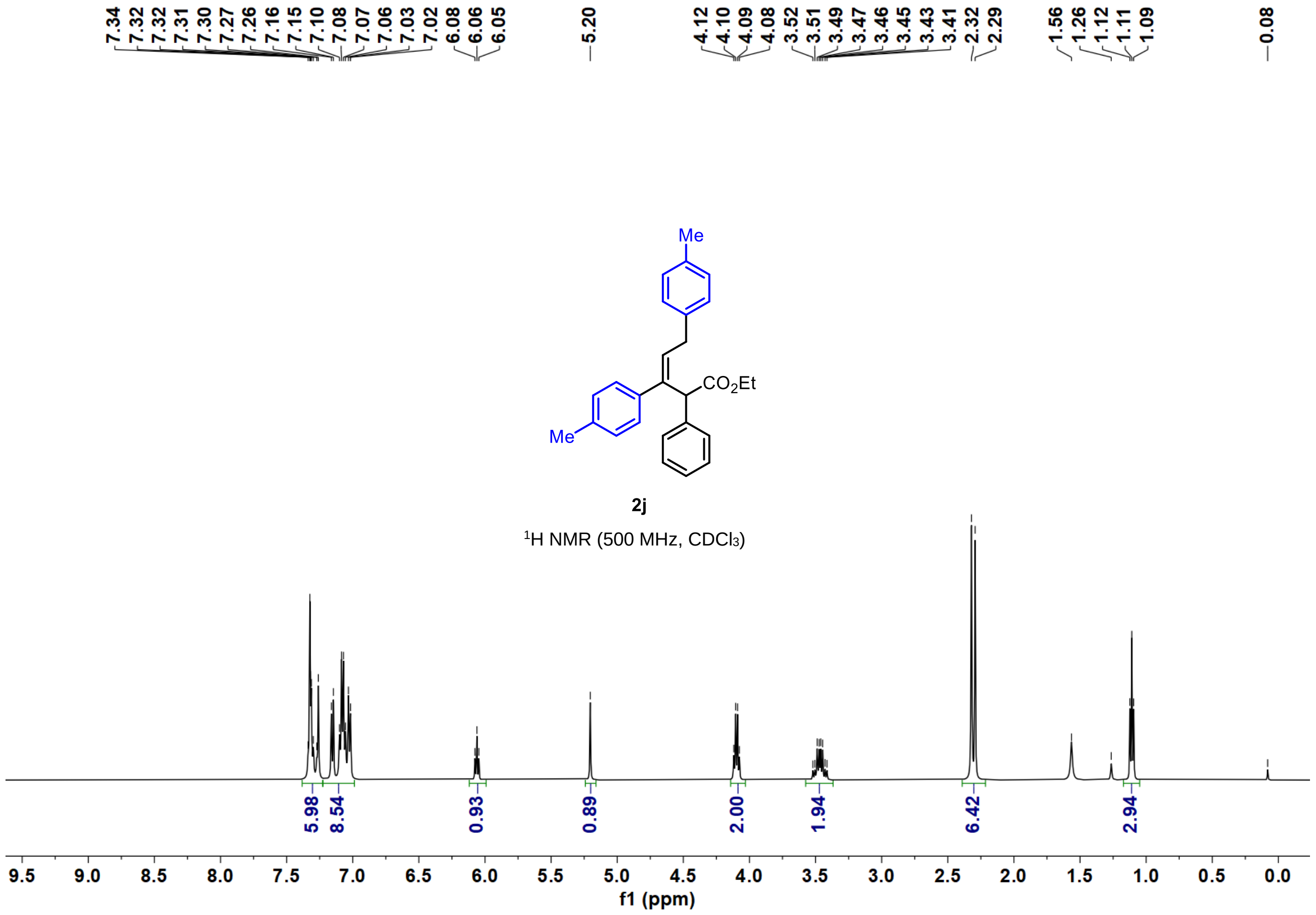
7.34 7.32 7.32 7.31 7.30 7.27 7.26 7.16 7.15 7.10 7.08 7.07 7.06 7.03 7.02 6.08 6.06 6.05
5.20
4.12 4.10 4.09 4.08 3.52 3.51 3.49 3.47 3.46 3.45 3.43 3.41 2.32 2.29
1.56 1.26 1.12 1.11 1.09
0.08
Me
CO_2Et
Me
2j
^{1}H NMR (500 MHz, CDCl3)
5.98 8.54 0.93 0.89 2.00 1.94 6.42 2.94
9.5 9.0 8.5 8.0 7.5 7.0 6.5 6.0 5.5 5.0 4.5 4.0 3.5 3.0 2.5 2.0 1.5 1.0 0.5 0.0
f1 (ppm)

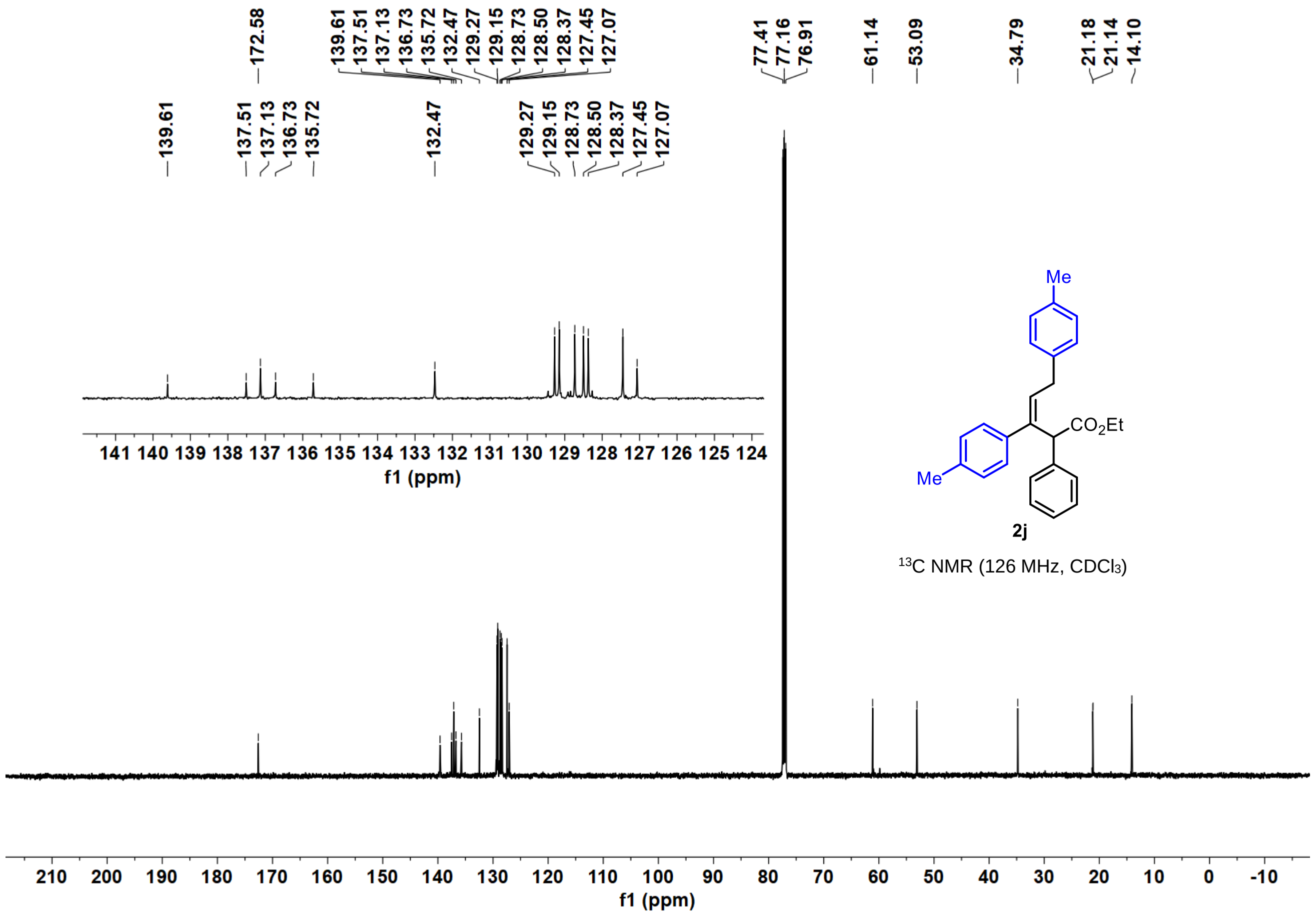
172.58
139.61 137.51 137.13 136.73 135.72 132.47 129.27 129.15 128.73 128.50 128.37 127.45 127.07
77.41 77.16 76.91
61.14
53.09
34.79
21.18 21.14 14.10
141 140 139 138 137 136 135 134 133 132 131 130 129 128 127 126 125 124
f1 (ppm)
Me
CO_2Et
Me
2j
^{13}C NMR (126 MHz, CDCl3)
210 200 190 180 170 160 150 140 130 120 110 100 90 80 70 60 50 40 30 20 10 0 -10
f1 (ppm)

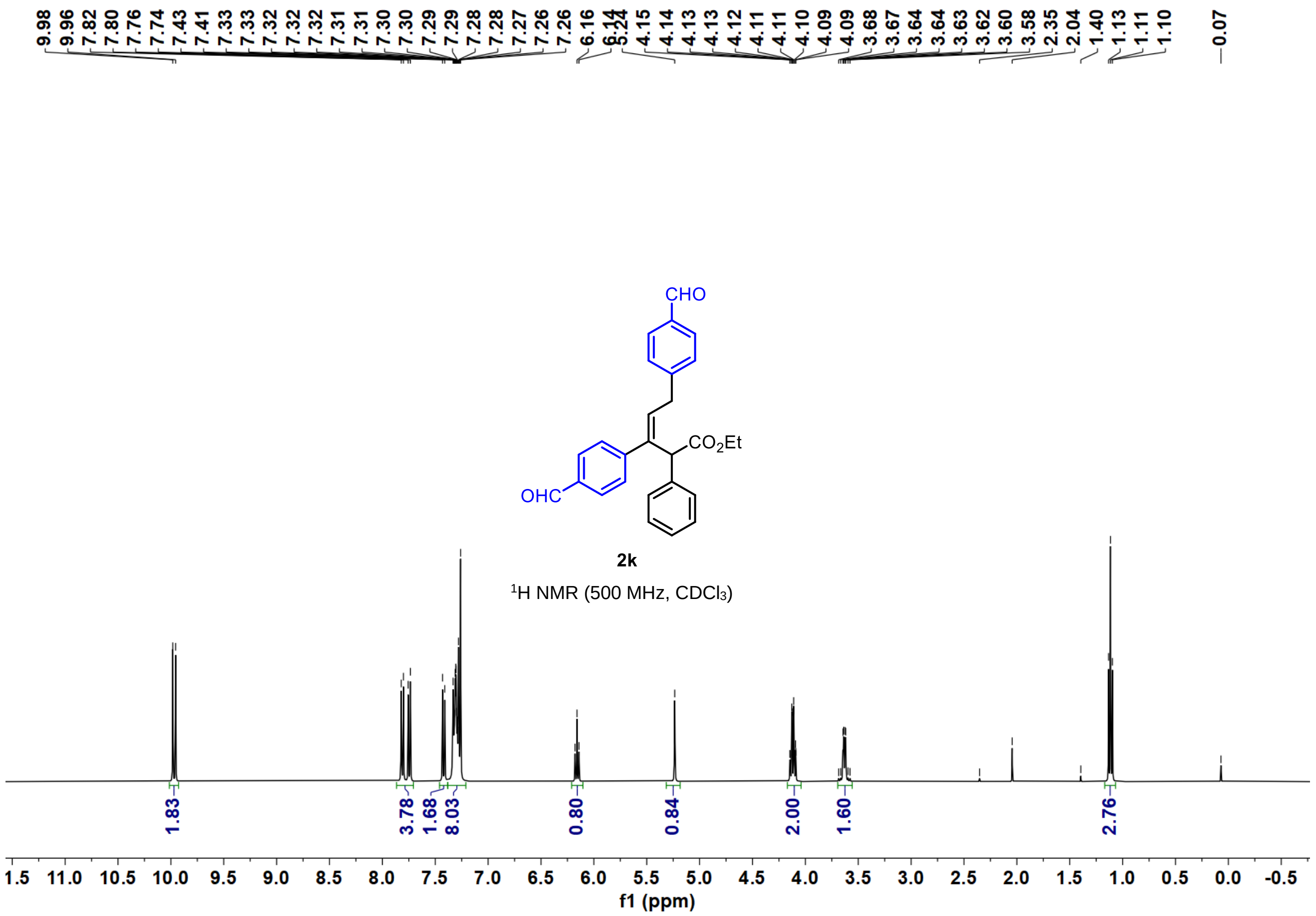
CHO
CO$_2$Et
OHC
2k
^{1}H NMR (500 MHz, CDCl$_3$)
f1 (ppm)

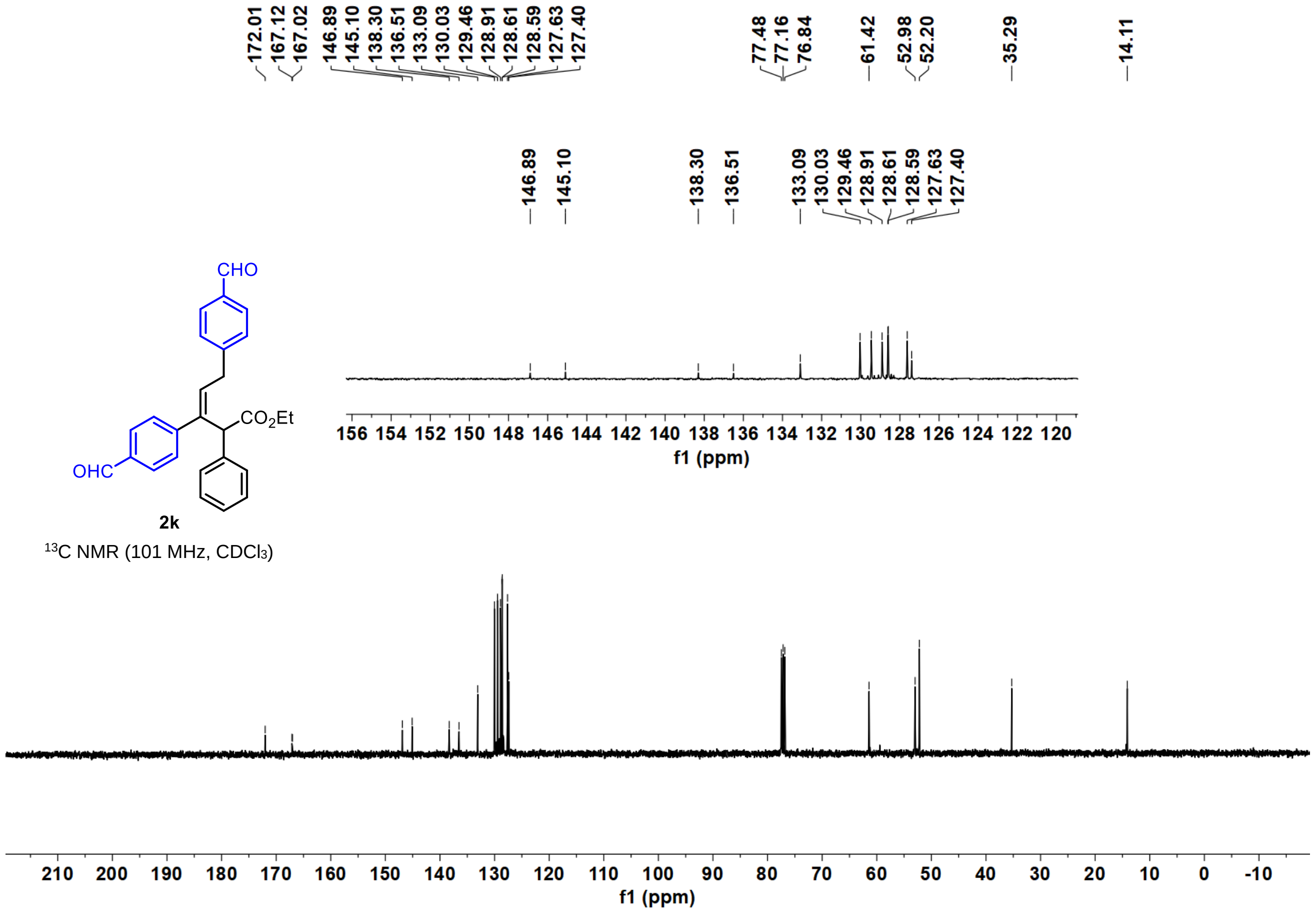
CHO
CO$_2$Et
OHC
2k
^{13}C NMR (101 MHz, CDCl$_3$)
f1 (ppm)

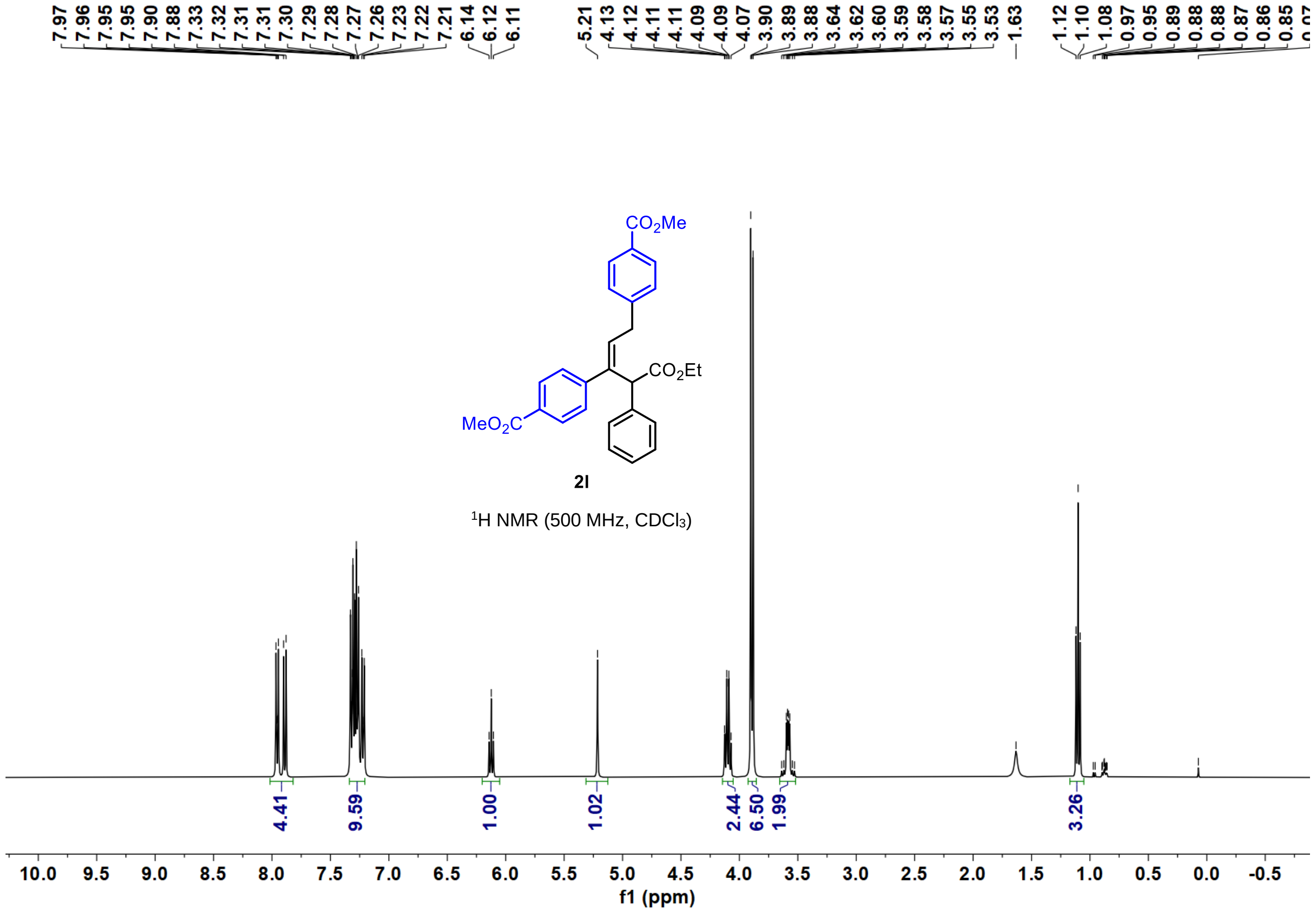
7.97 7.96 7.95 7.95 7.90 7.88 7.33 7.32 7.31 7.31 7.30 7.29 7.28 7.27 7.26 7.23 7.22 7.21 6.14 6.12 6.11 5.21 4.13 4.12 4.11 4.11 4.09 4.09 4.07 3.90 3.89 3.88 3.64 3.62 3.60 3.59 3.58 3.57 3.55 3.53 1.63 1.12 1.10 1.08 0.97 0.95 0.89 0.88 0.88 0.87 0.86 0.85
CO2Me
CO2Et
MeO2C
2l
1H NMR (500 MHz, CDCl3)
4.41 9.59 1.00 1.02 2.44 6.50 1.99 3.26
f1 (ppm)

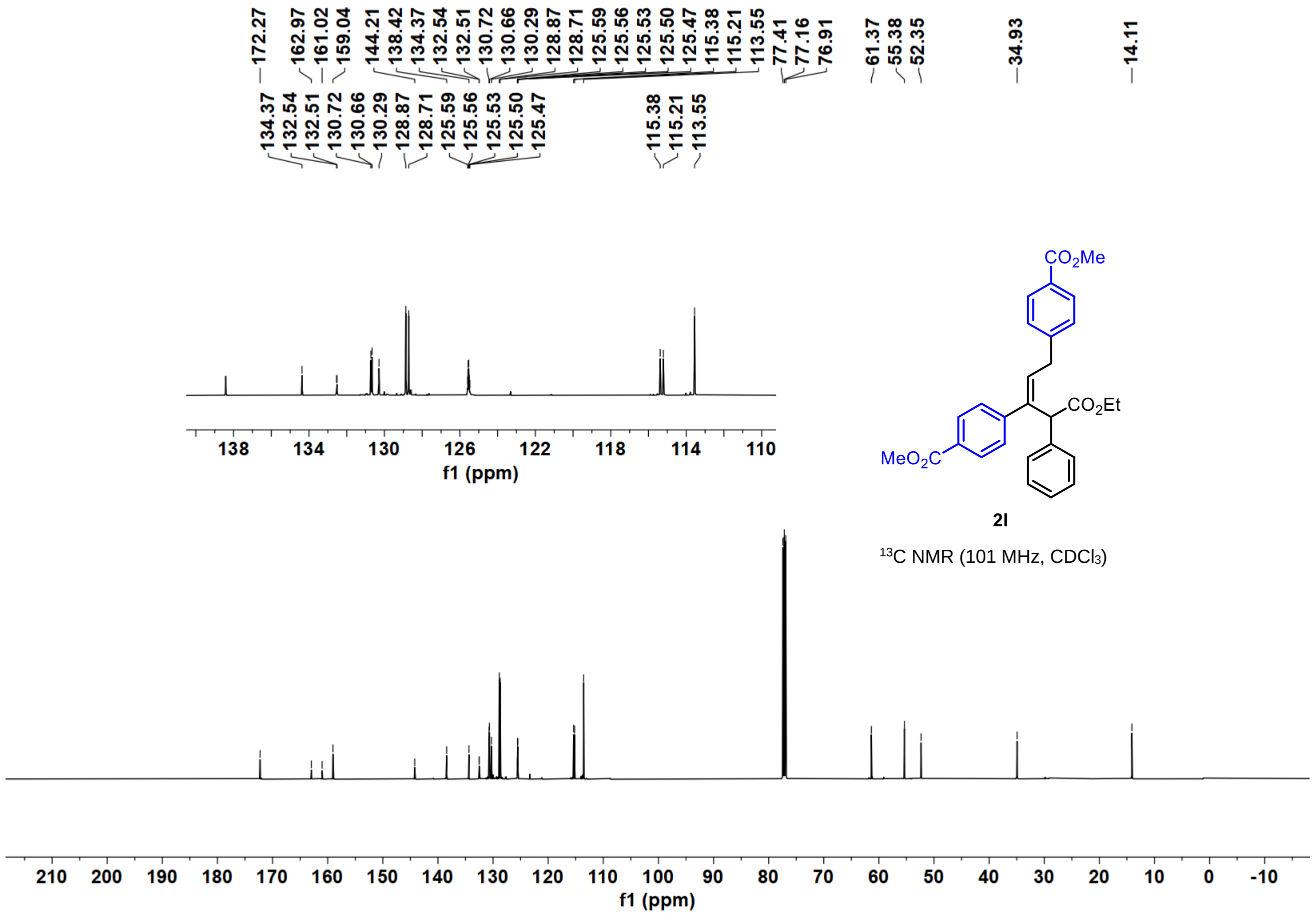
172.27 162.97 161.02 159.04 144.21 138.42 134.37 132.54 132.51 130.72 130.66 130.29 128.87 128.71 125.59 125.56 125.53 125.50 125.47 115.38 115.21 113.55 77.41 77.16 76.91 61.37 55.38 52.35 34.93 14.11
CO2Me
CO2Et
MeO2C
2l
13C NMR (101 MHz, CDCl3)
f1 (ppm)

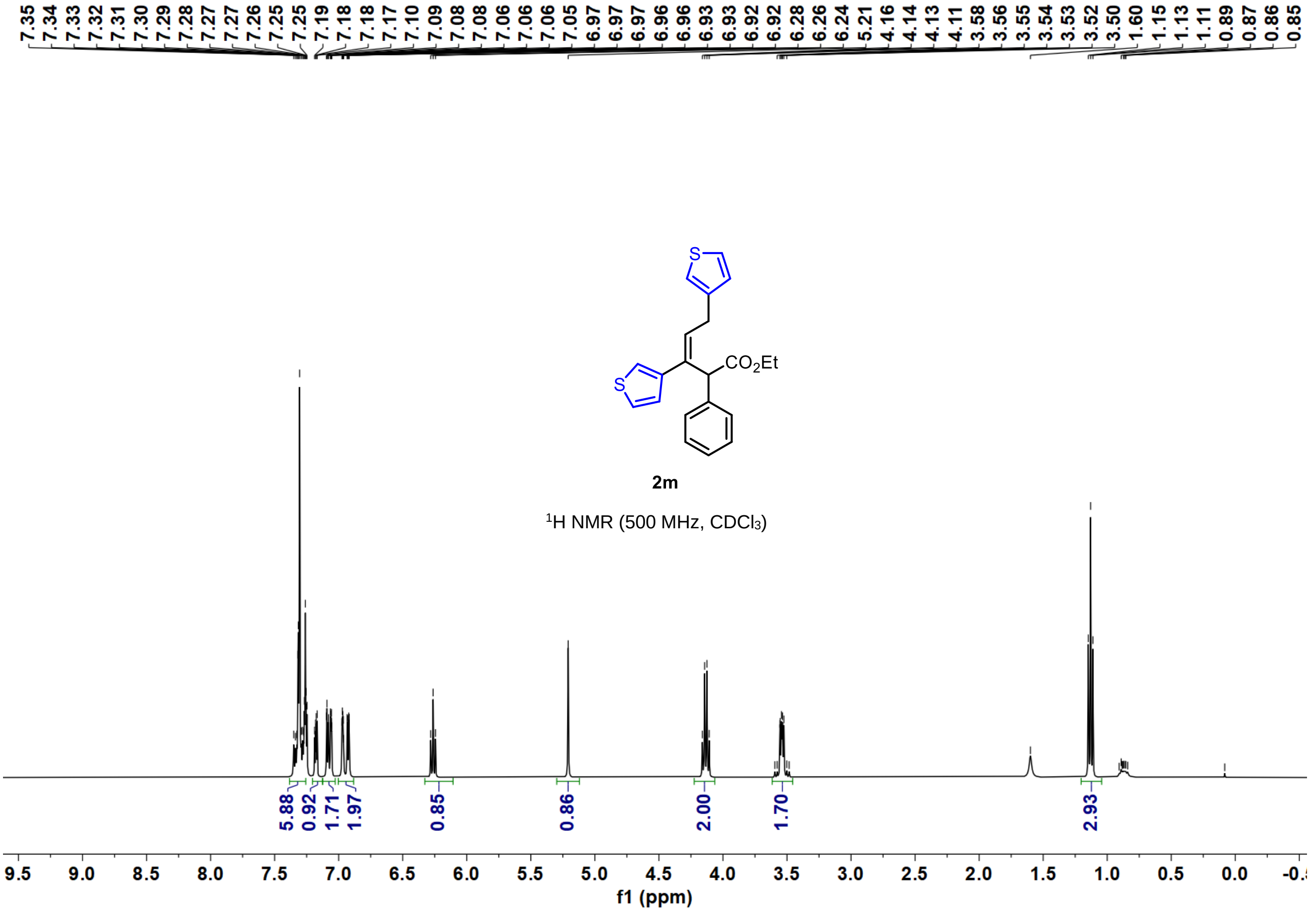
7.35 7.34 7.33 7.32 7.31 7.30 7.29 7.28 7.27 7.27 7.26 7.25 7.25 7.19 7.18 7.18 7.17 7.10 7.09 7.08 7.08 7.06 7.06 7.06 7.05 6.97 6.97 6.96 6.96 6.93 6.93 6.92 6.92 6.28 6.26 6.24 5.21 4.16 4.14 4.13 4.11 3.58 3.56 3.55 3.54 3.53 3.52 3.50 1.60 1.15 1.13 1.11 0.89 0.87 0.86 0.85
S
S
CO2Et
2m
1H NMR (500 MHz, CDCl3)
5.88 0.92 1.71 1.97 0.85 0.86 2.00 1.70 2.93
f1 (ppm)

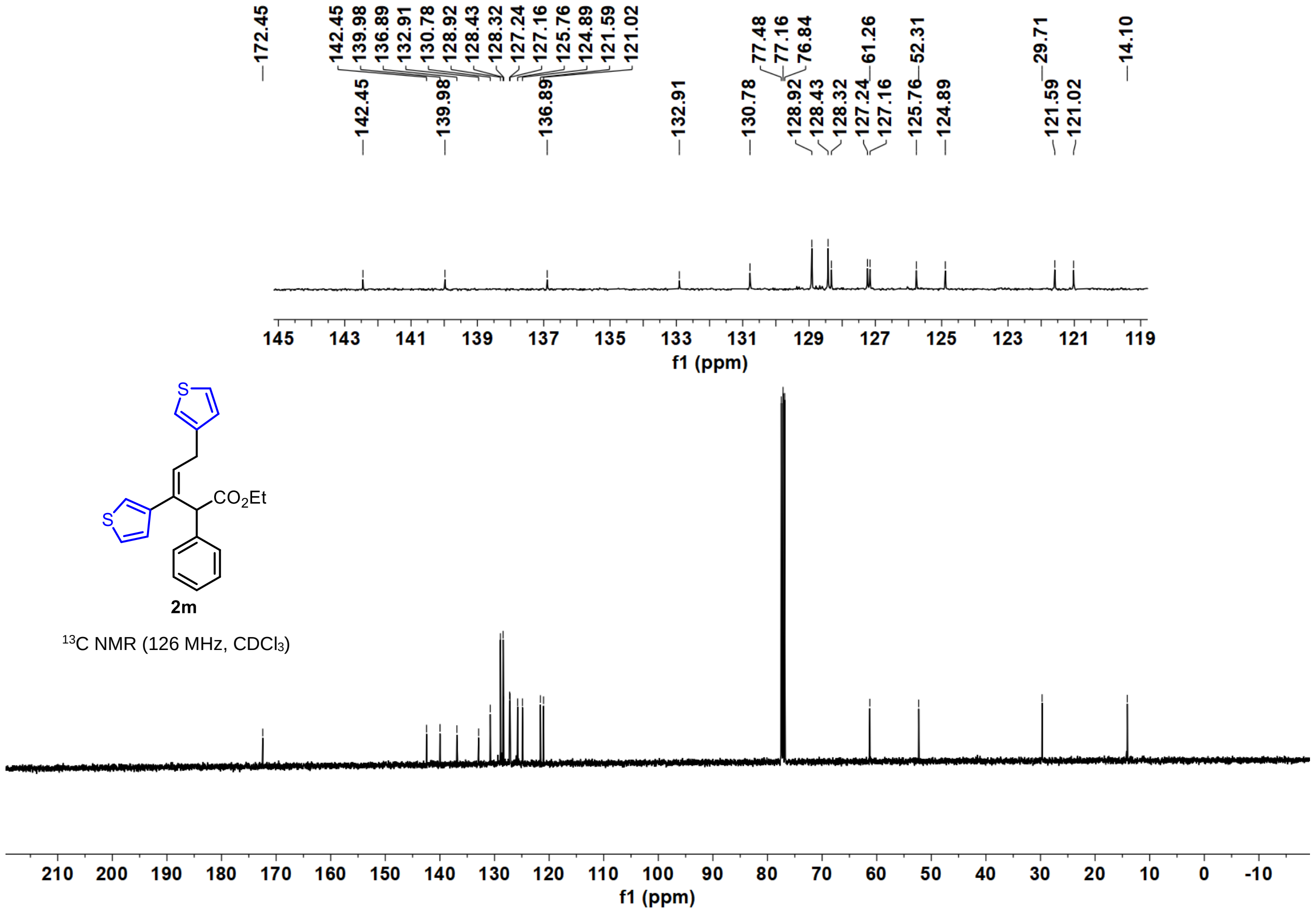
172.45 142.45 139.98 136.89 132.91 130.78 128.92 128.43 128.32 127.24 127.16 125.76 124.89 121.59 121.02 77.48 77.16 76.84 61.26 52.31 29.71 14.10
f1 (ppm)
S
S
CO2Et
2m
13C NMR (126 MHz, CDCl3)
f1 (ppm)

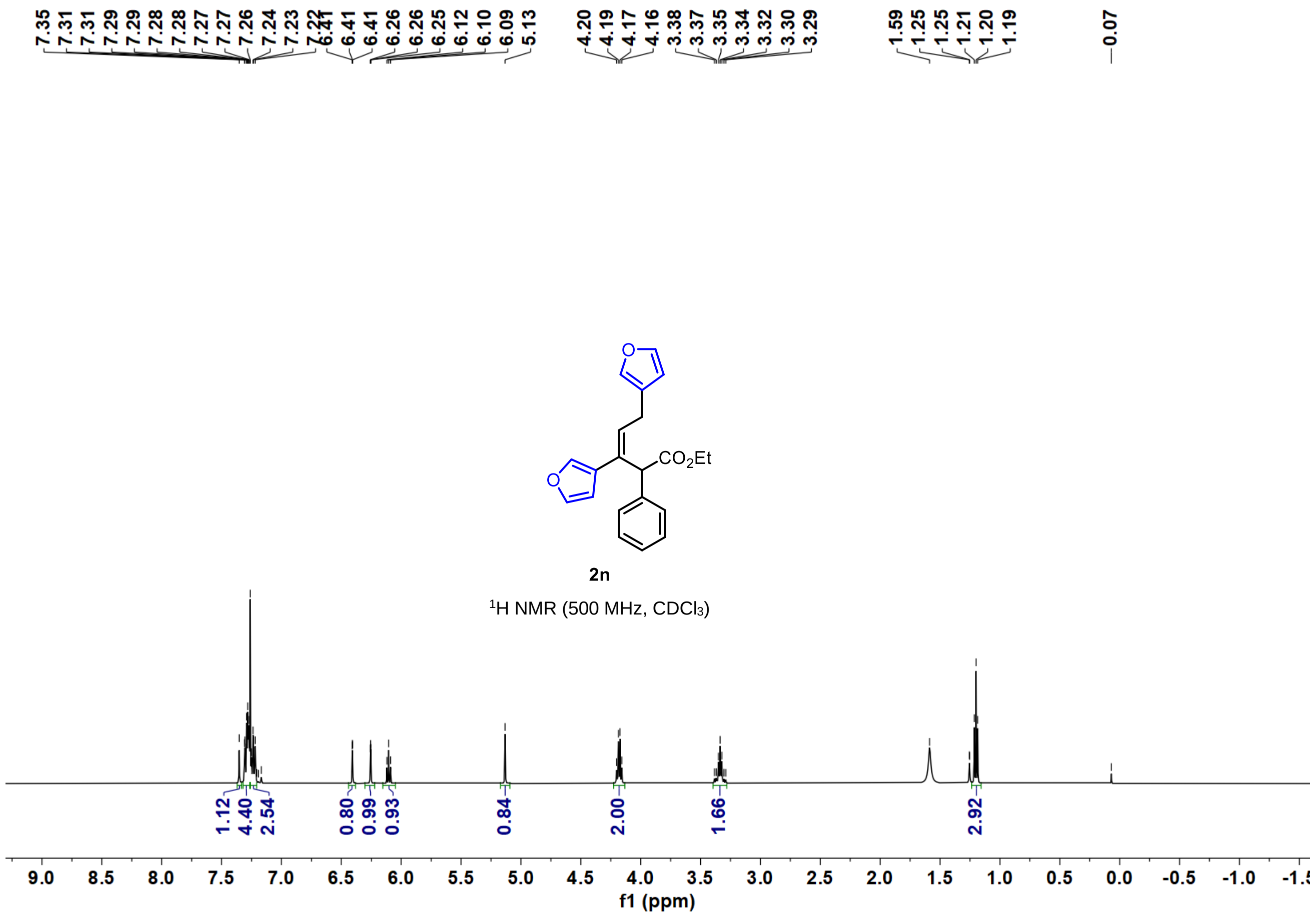

2n

^{1}H NMR (500 MHz, $CDCl_3$)

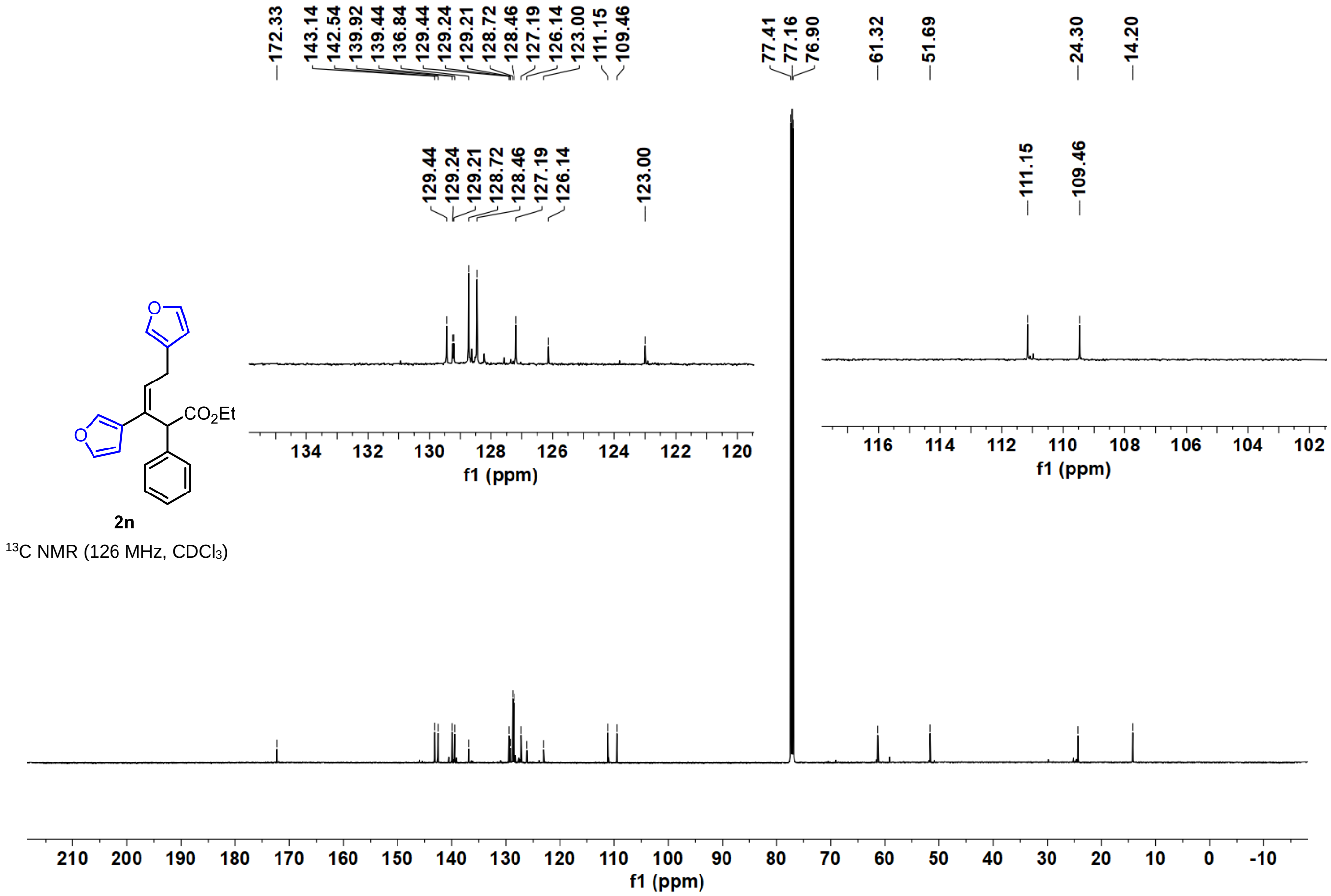

2n

^{13}C NMR (126 MHz, $CDCl_3$)

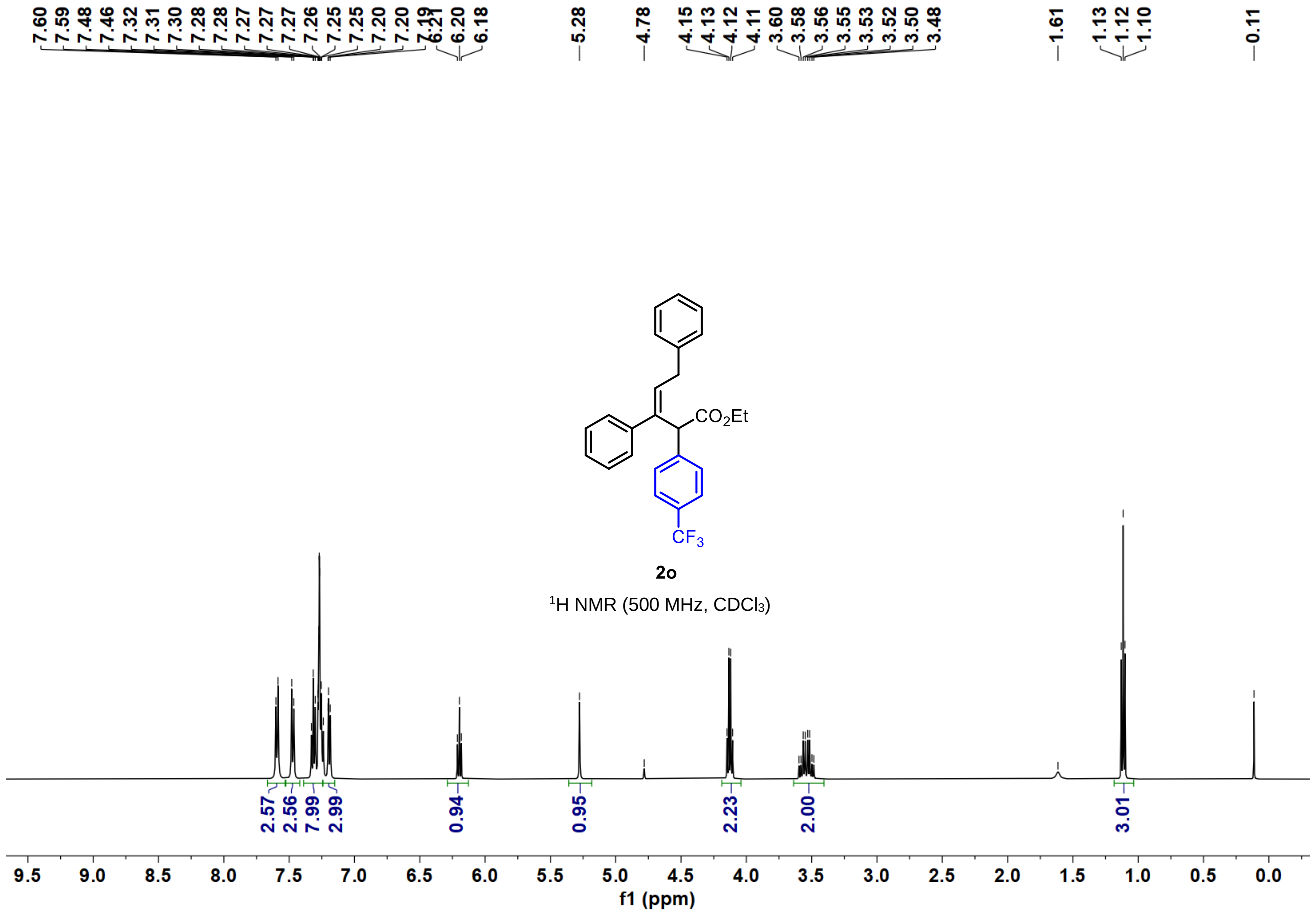
7.60 7.59 7.48 7.46 7.32 7.31 7.30 7.28 7.28 7.27 7.27 7.27 7.26 7.25 7.25 7.20 7.20 7.19 6.21 6.20 6.18 5.28 4.78 4.15 4.13 4.12 4.11 3.60 3.58 3.56 3.55 3.53 3.52 3.50 3.48 1.61 1.13 1.12 1.10 0.11
CO2Et
CF3
2o
1H NMR (500 MHz, CDCl3)
2.57 2.56 7.99 2.99 0.94 0.95 2.23 2.00 3.01
f1 (ppm)
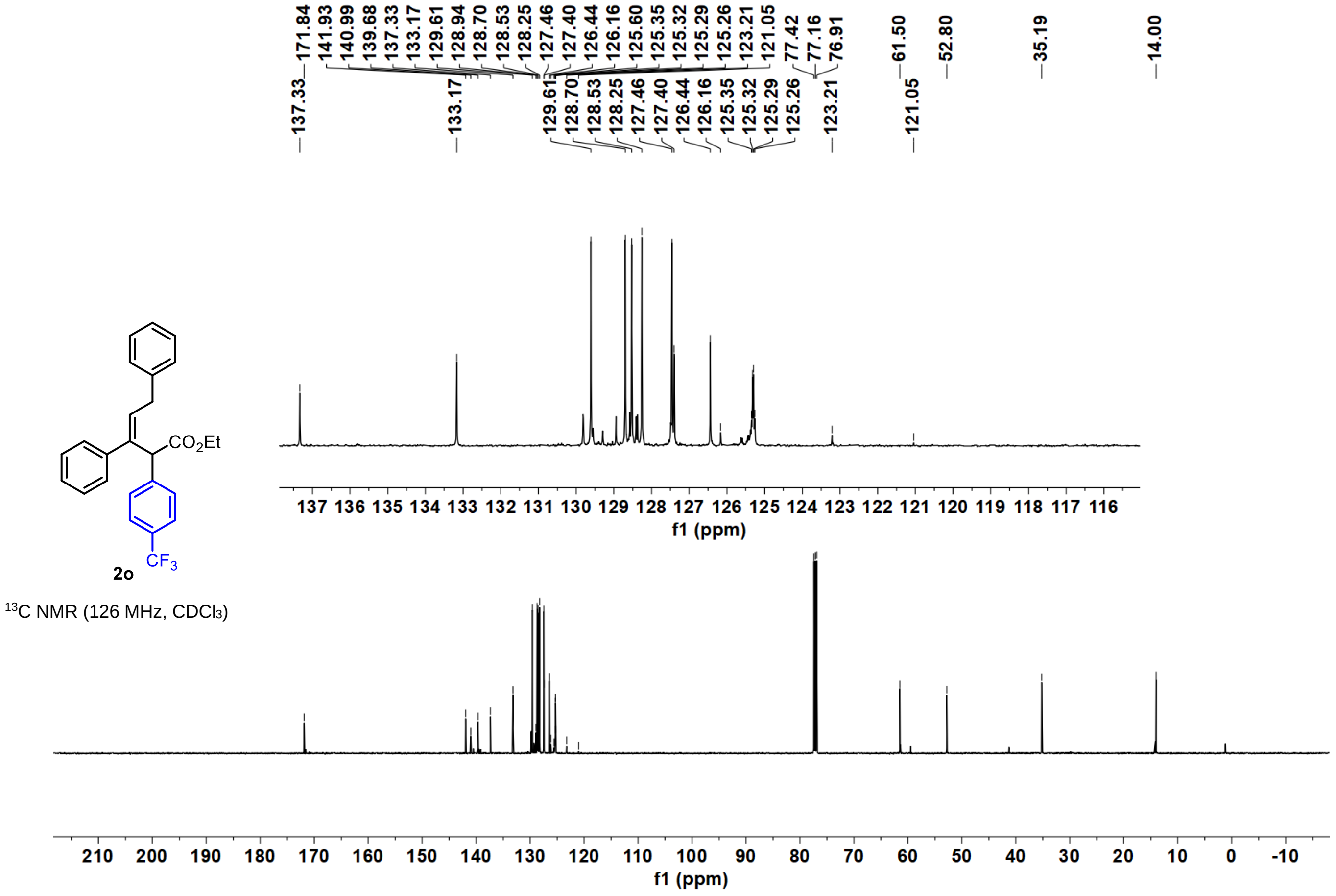
171.84 141.93 140.99 139.68 137.33 133.17 129.61 128.94 128.70 128.53 128.25 127.46 127.40 126.44 126.16 125.60 125.35 125.32 125.29 125.26 123.21 121.05 77.42 77.16 76.91 61.50 52.80 35.19 14.00
137.33 133.17 129.61 128.70 128.53 128.25 127.46 127.40 126.44 126.16 125.35 125.32 125.29 125.26 123.21 121.05
CO2Et
CF3
2o
13C NMR (126 MHz, CDCl3)
f1 (ppm)

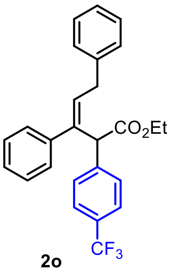

2o

^{19}F NMR (471 MHz, $CDCl_3$)

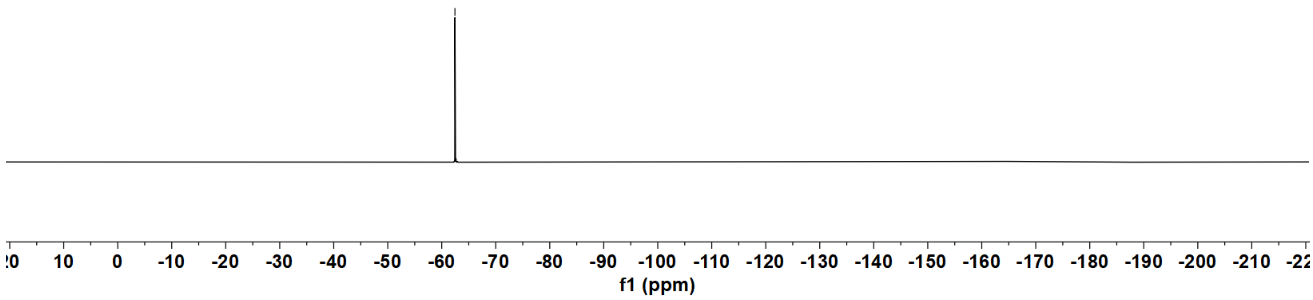

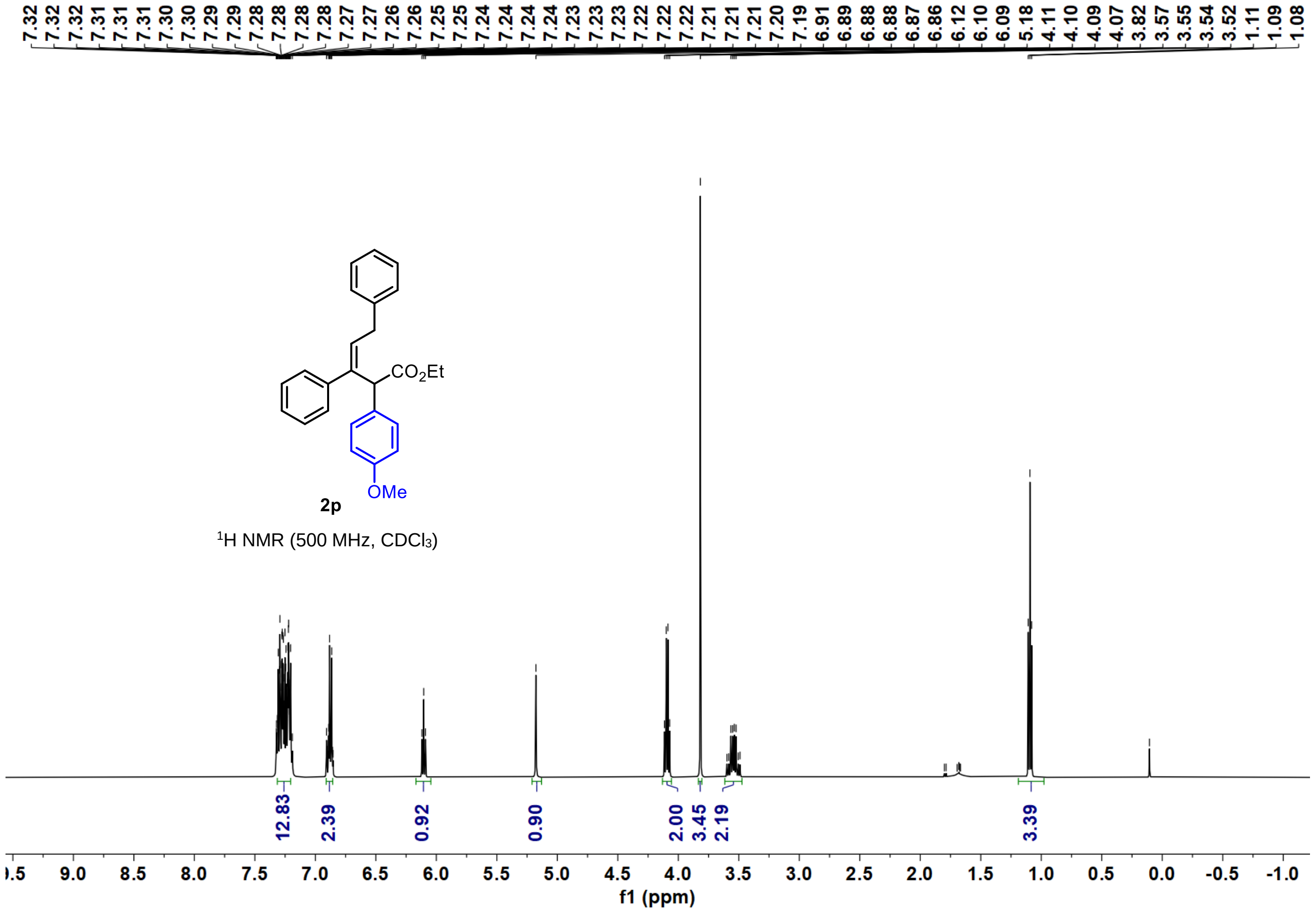
CO2Et
OMe
2p
1H NMR (500 MHz, CDCl3)
f1 (ppm)

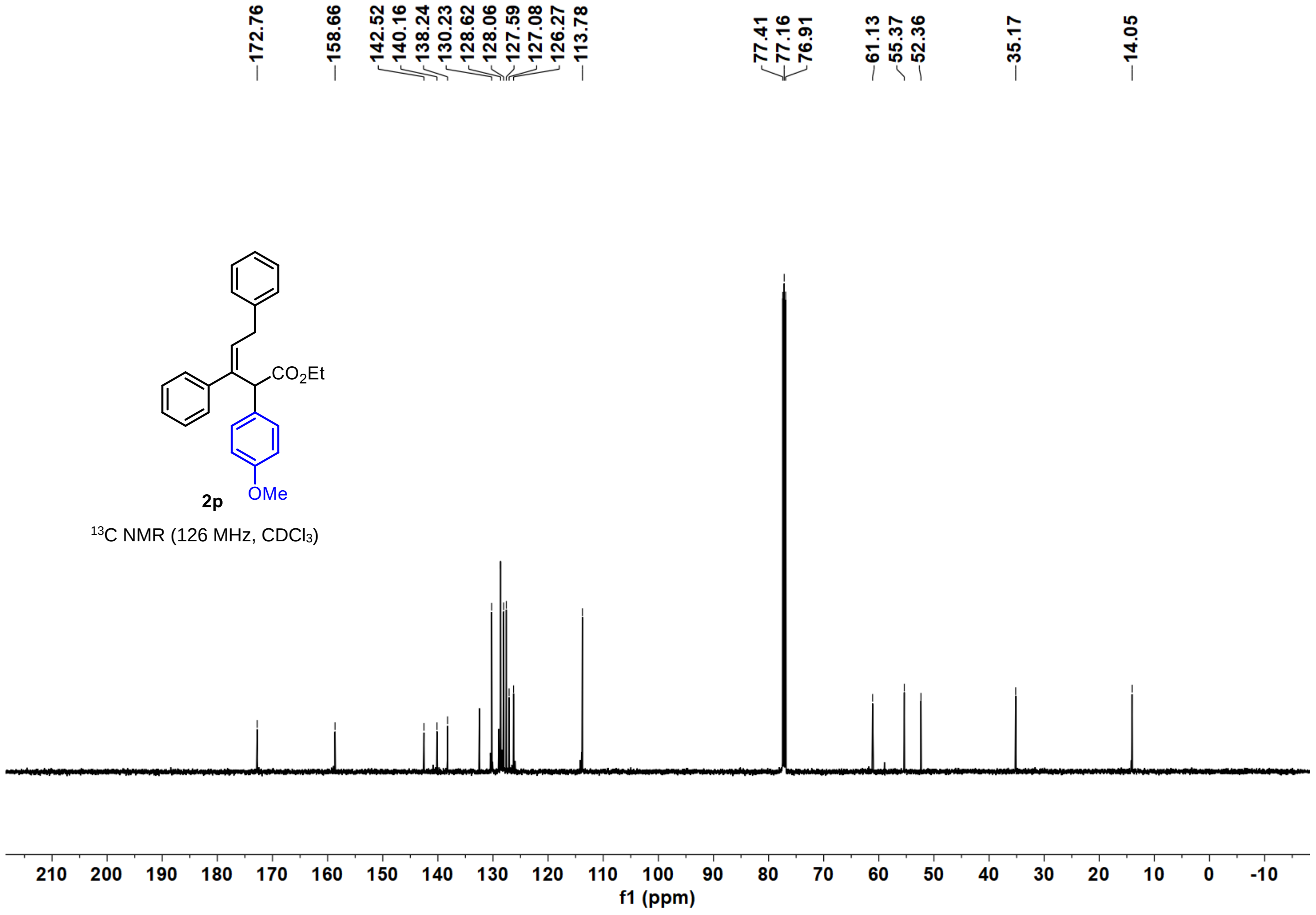
CO2Et
OMe
2p
13C NMR (126 MHz, CDCl3)
f1 (ppm)

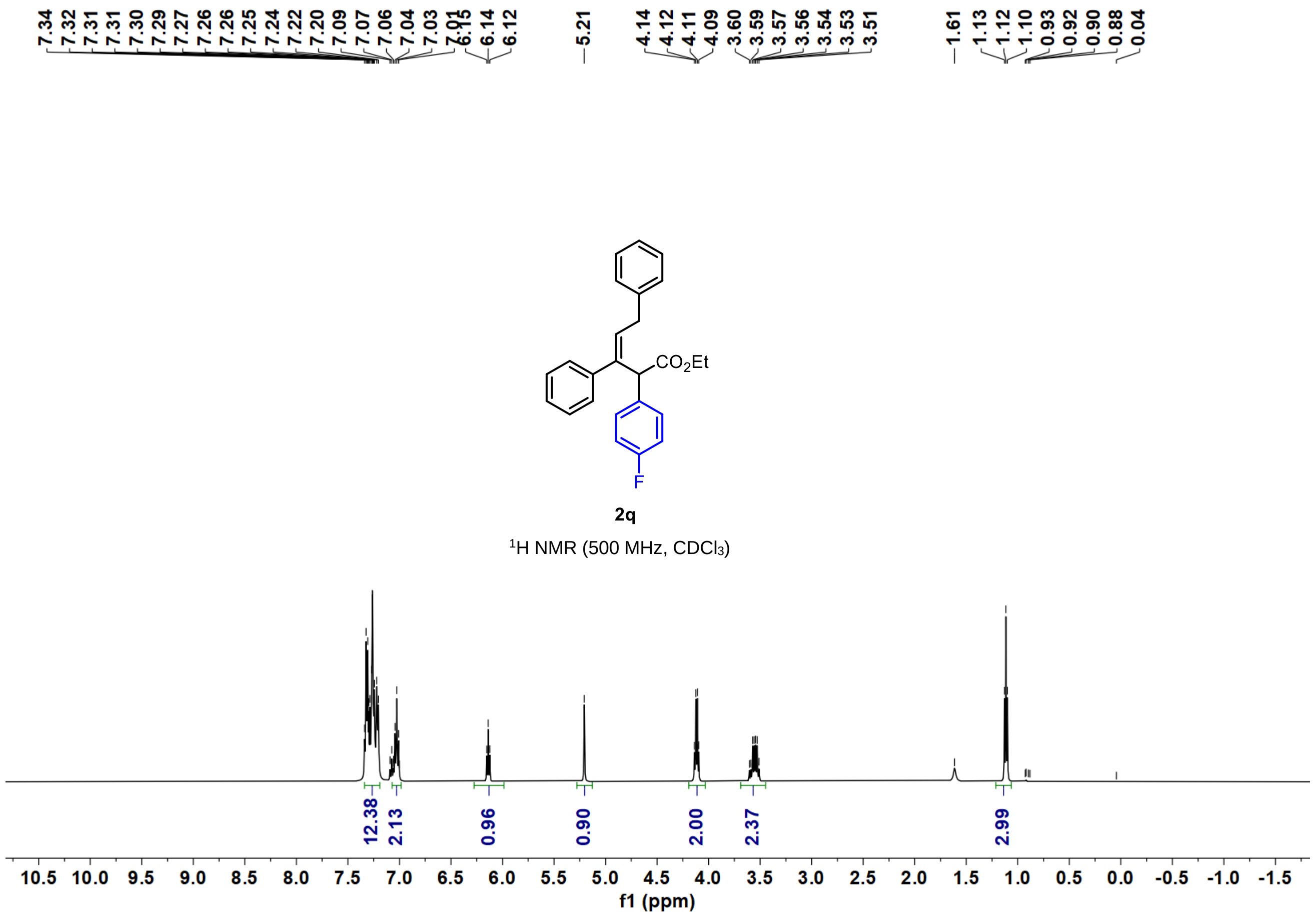

2q

^{1}H NMR (500 MHz, $CDCl_3$)

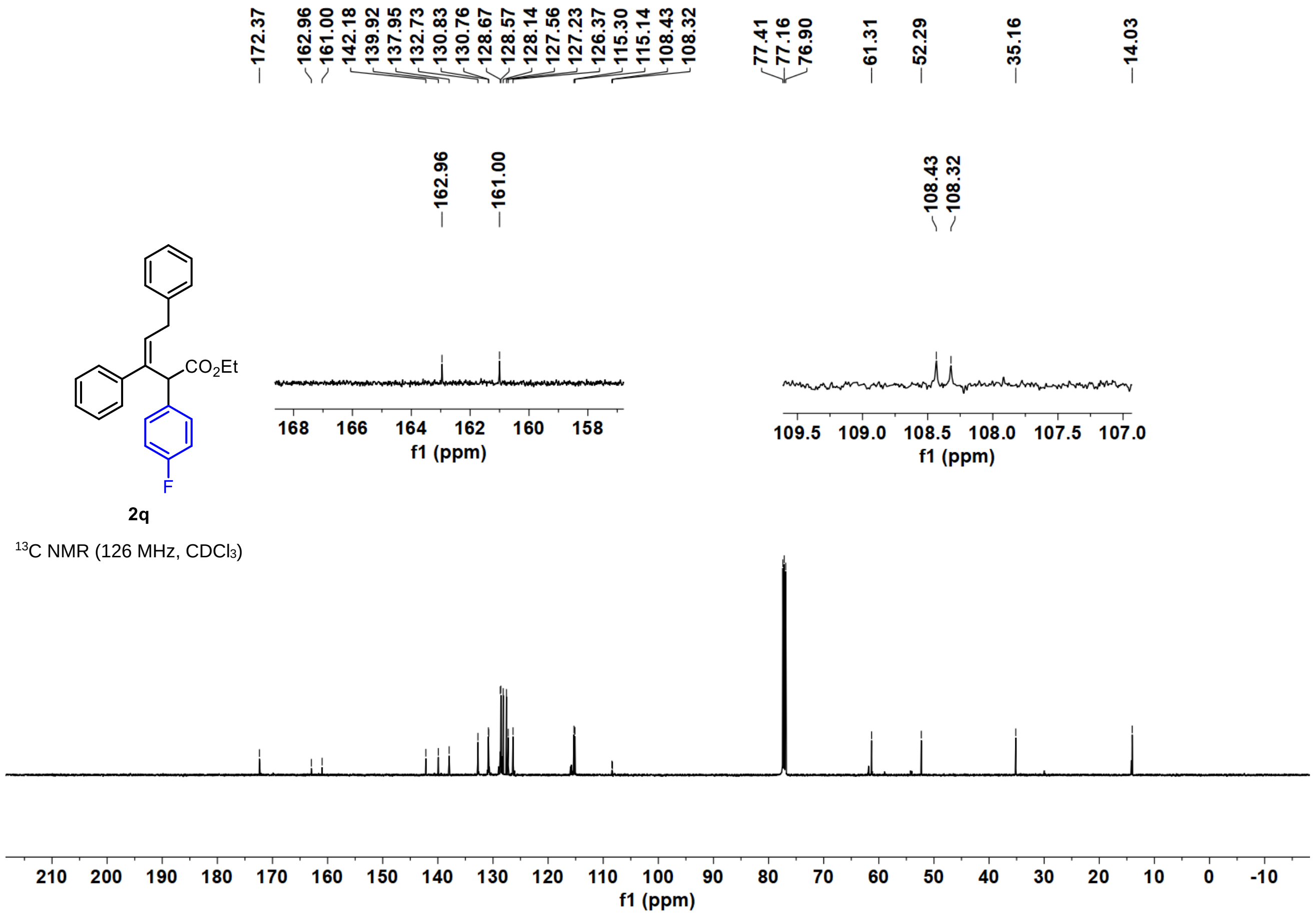

2q

^{13}C NMR (126 MHz, $CDCl_3$)

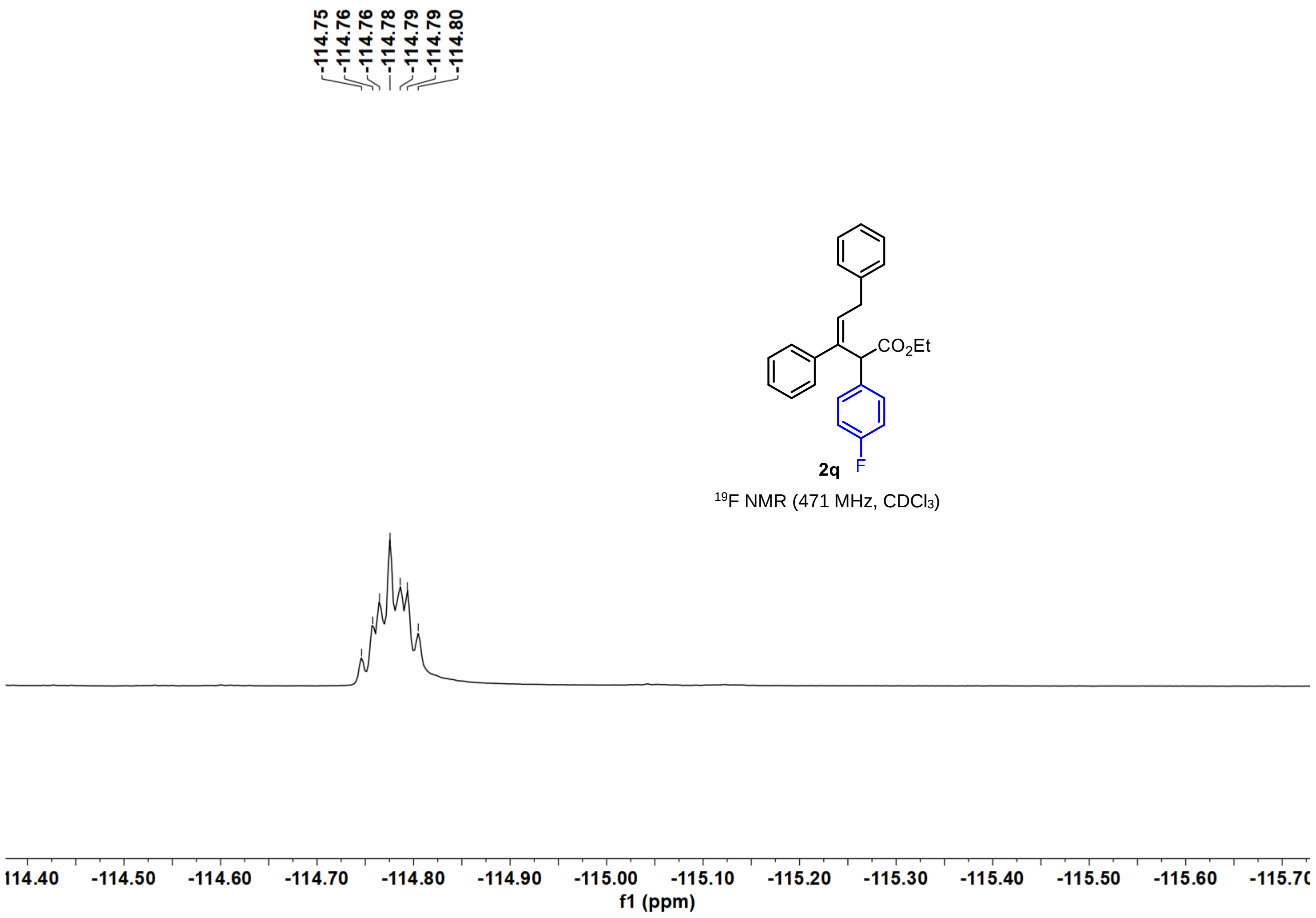
-114.75
-114.76
-114.76
-114.78
-114.79
-114.79
-114.80
CO2Et
F
2q
19F NMR (471 MHz, CDCl3)
-114.40
-114.50
-114.60
-114.70
-114.80
-114.90
-115.00
-115.10
-115.20
-115.30
-115.40
-115.50
-115.60
-115.70
f1 (ppm)

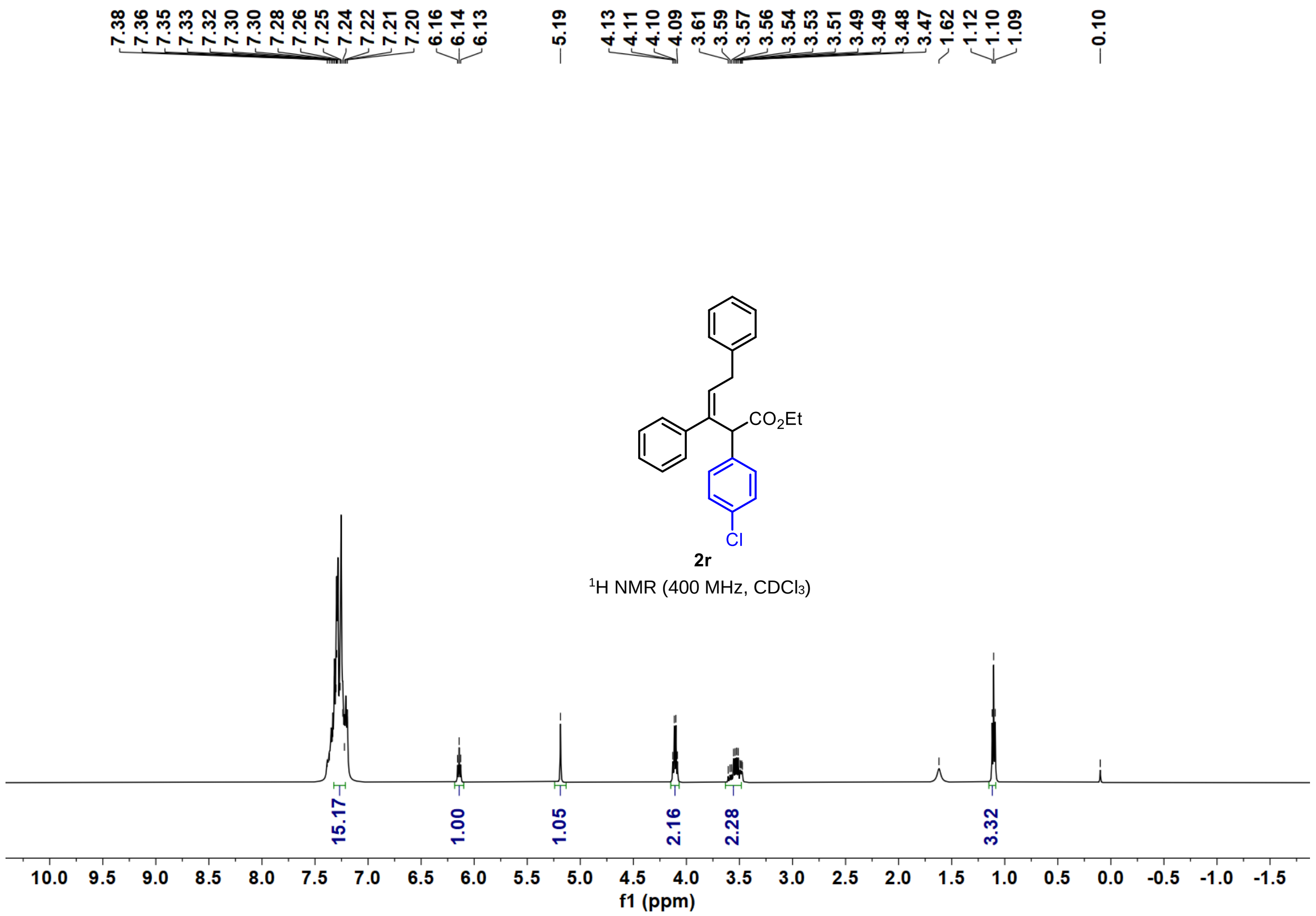
7.38 7.36 7.35 7.33 7.32 7.30 7.30 7.28 7.26 7.25 7.24 7.22 7.21 7.20 6.16 6.14 6.13 5.19 4.13 4.11 4.10 4.09 3.61 3.59 3.57 3.56 3.54 3.53 3.51 3.49 3.49 3.48 3.47 1.62 1.12 1.10 1.09 0.10
CO_2Et
Cl
2r
^1H NMR (400 MHz, $CDCl_3$)
15.17 1.00 1.05 2.16 2.28 3.32
10.0 9.5 9.0 8.5 8.0 7.5 7.0 6.5 6.0 5.5 5.0 4.5 4.0 3.5 3.0 2.5 2.0 1.5 1.0 0.5 0.0 -0.5 -1.0 -1.5
f1 (ppm)

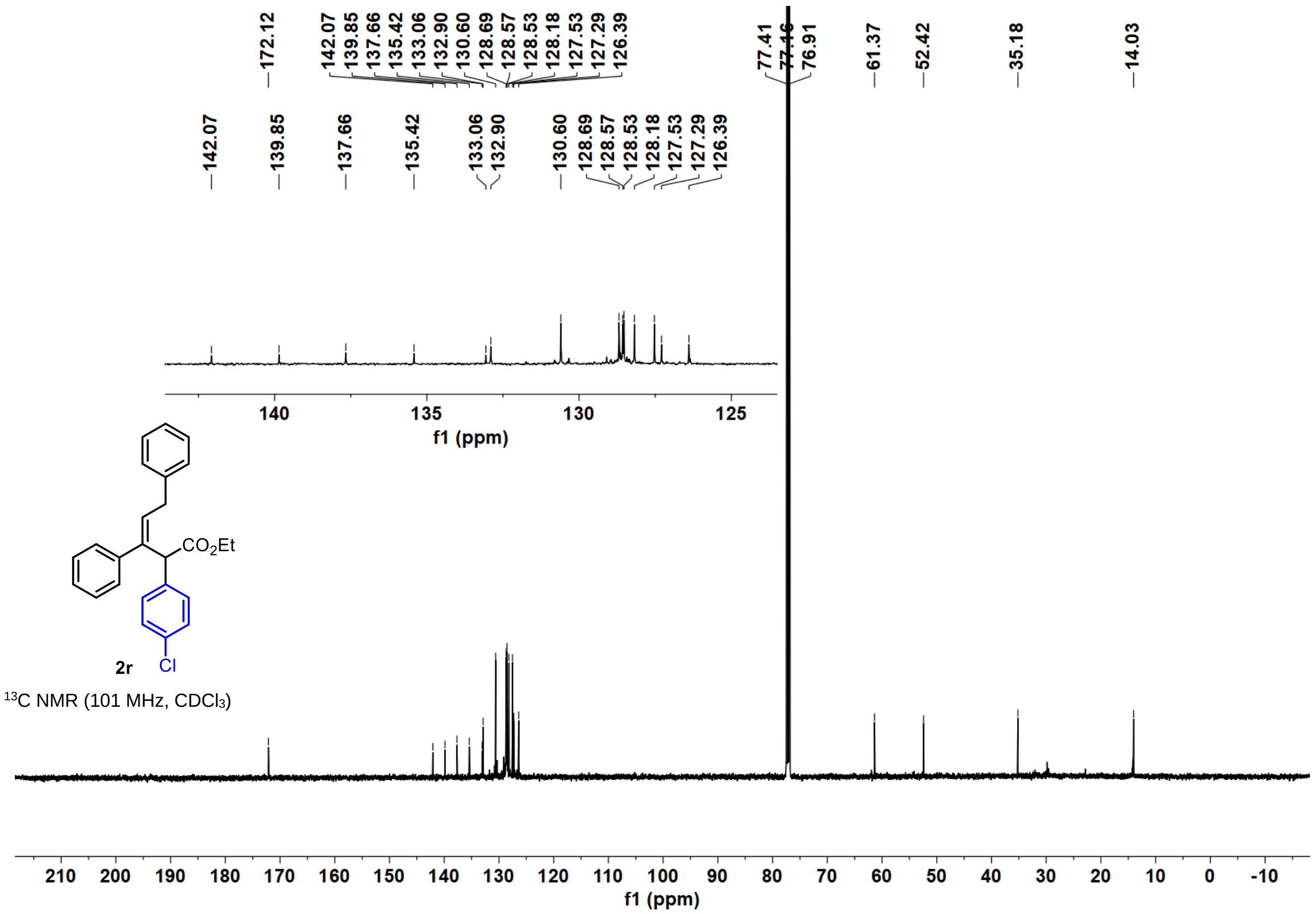
172.12 142.07 139.85 137.66 135.42 133.06 132.90 130.60 128.69 128.57 128.53 128.18 127.53 127.29 126.39 77.41 77.16 76.91 61.37 52.42 35.18 14.03
142.07 139.85 137.66 135.42 133.06 132.90 130.60 128.69 128.57 128.53 128.18 127.53 127.29 126.39
140 135 130 125
f1 (ppm)
CO_2Et
2r
Cl
^{13}C NMR (101 MHz, $CDCl_3$)
210 200 190 180 170 160 150 140 130 120 110 100 90 80 70 60 50 40 30 20 10 0 -10
f1 (ppm)

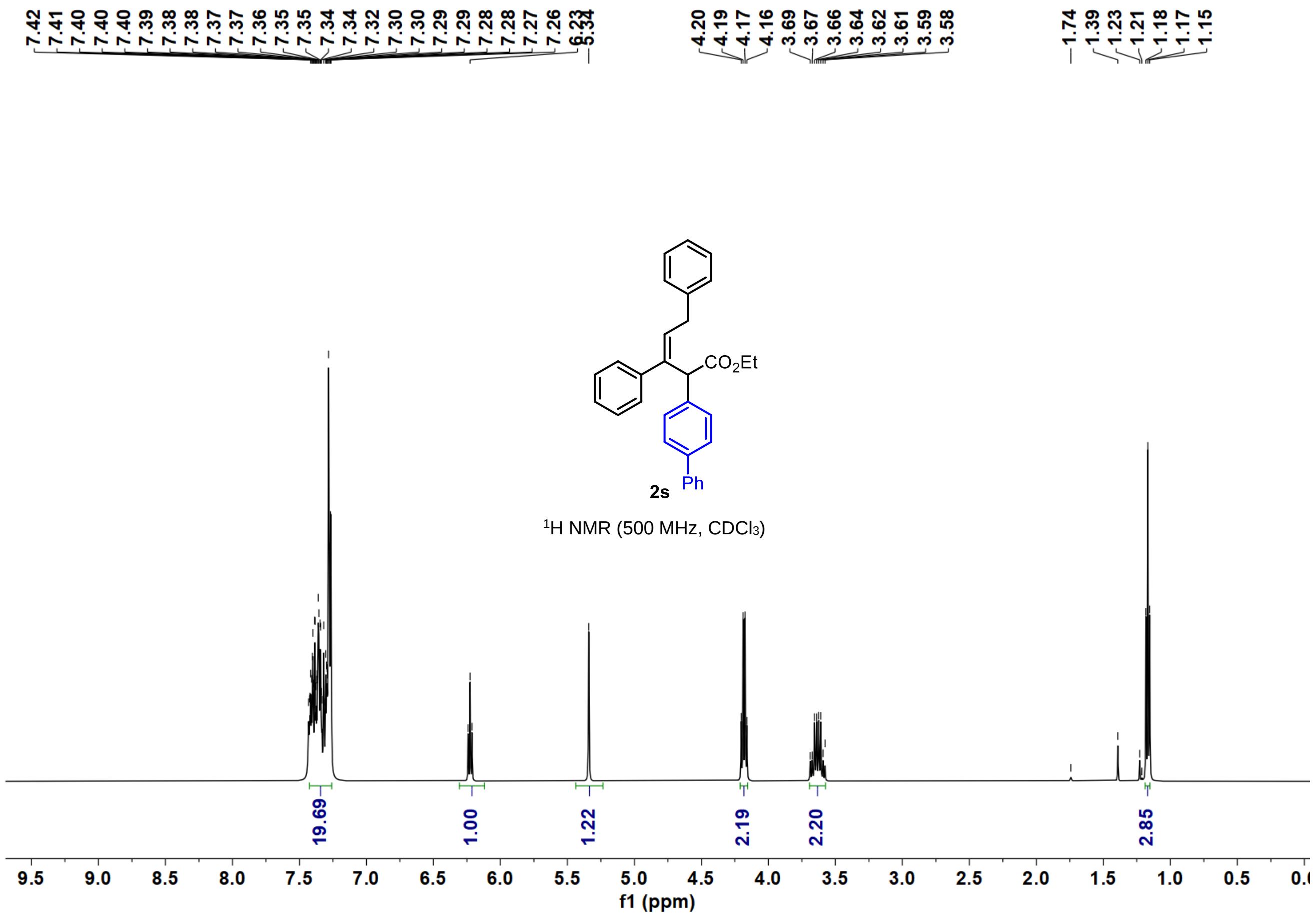
7.42 7.41 7.40 7.40 7.39 7.38 7.38 7.37 7.37 7.36 7.35 7.35 7.34 7.34 7.32 7.30 7.30 7.29 7.29 7.28 7.28 7.27 7.26 6.23 5.34
4.20 4.19 4.17 4.16 3.69 3.67 3.66 3.64 3.62 3.61 3.59 3.58
1.74 1.39 1.23 1.21 1.18 1.17 1.15
CO2Et
Ph
2s
1H NMR (500 MHz, CDCl3)
19.69 1.00 1.22 2.19 2.20 2.85
f1 (ppm)

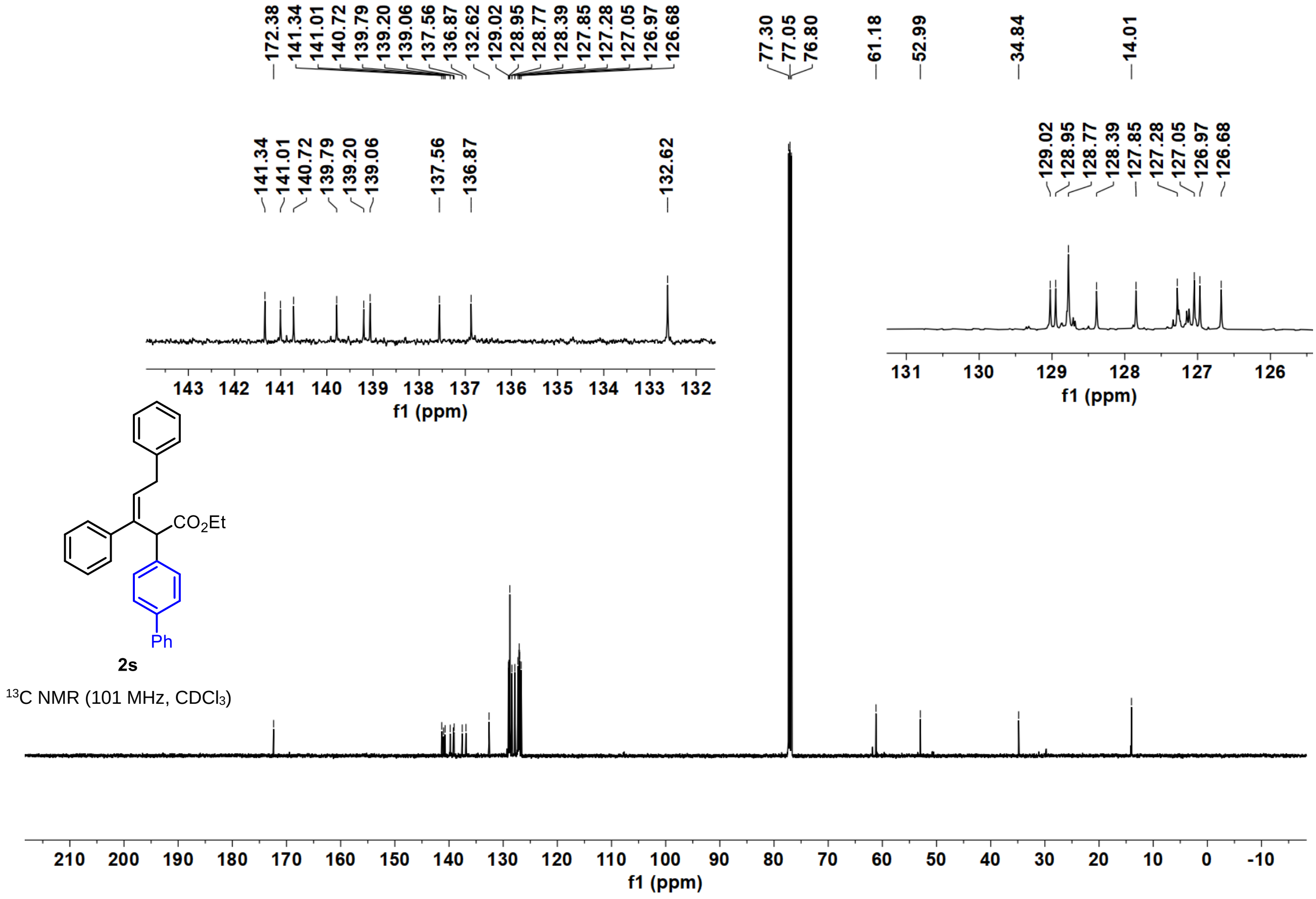
172.38 141.34 141.01 140.72 139.79 139.20 139.06 137.56 136.87 132.62 129.02 128.95 128.77 128.39 127.85 127.28 127.05 126.97 126.68
77.30 77.05 76.80
61.18
52.99
34.84
14.01
f1 (ppm)
CO2Et
Ph
2s
13C NMR (101 MHz, CDCl3)

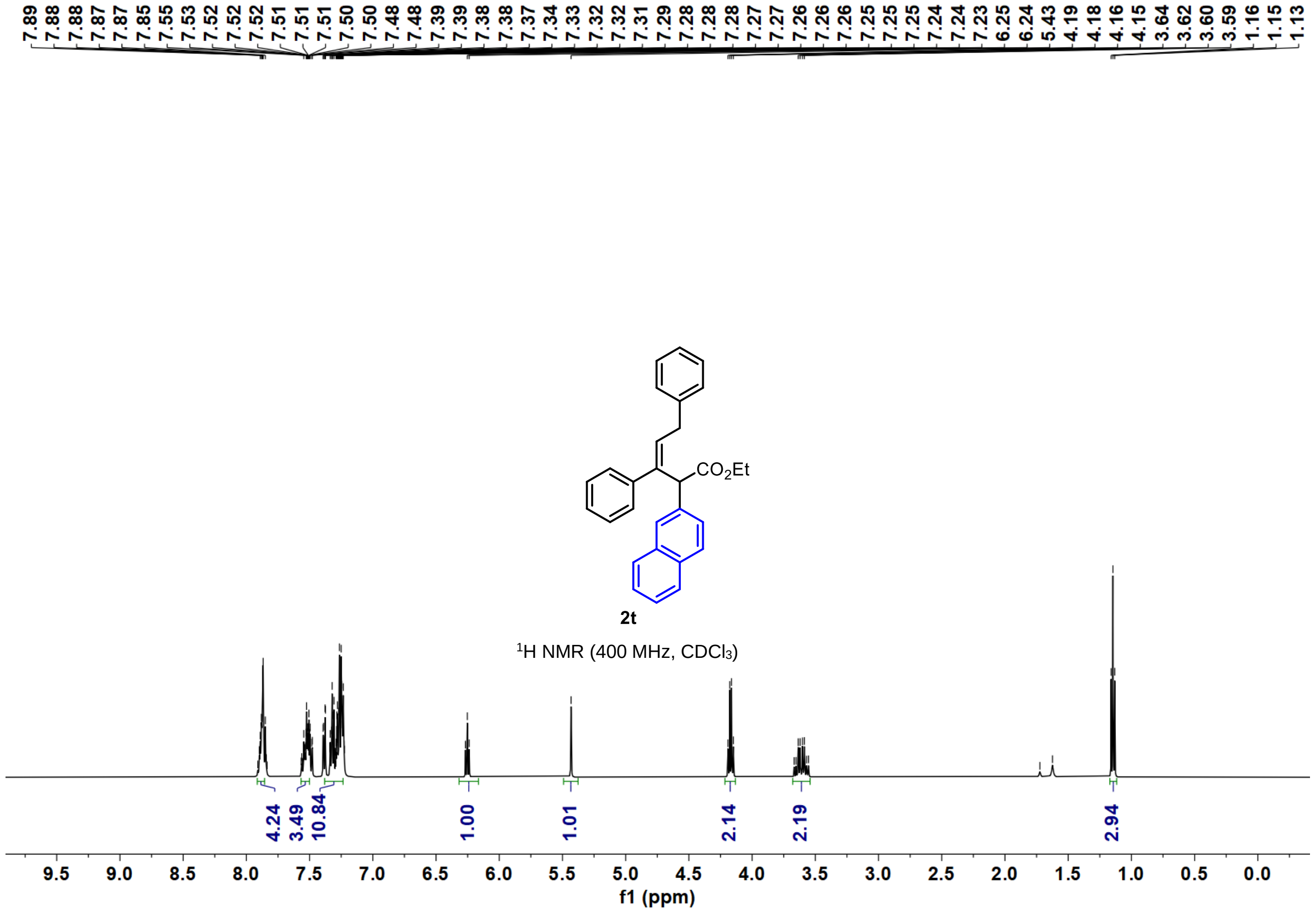

2t

^{1}H NMR (400 MHz, $CDCl_3$)

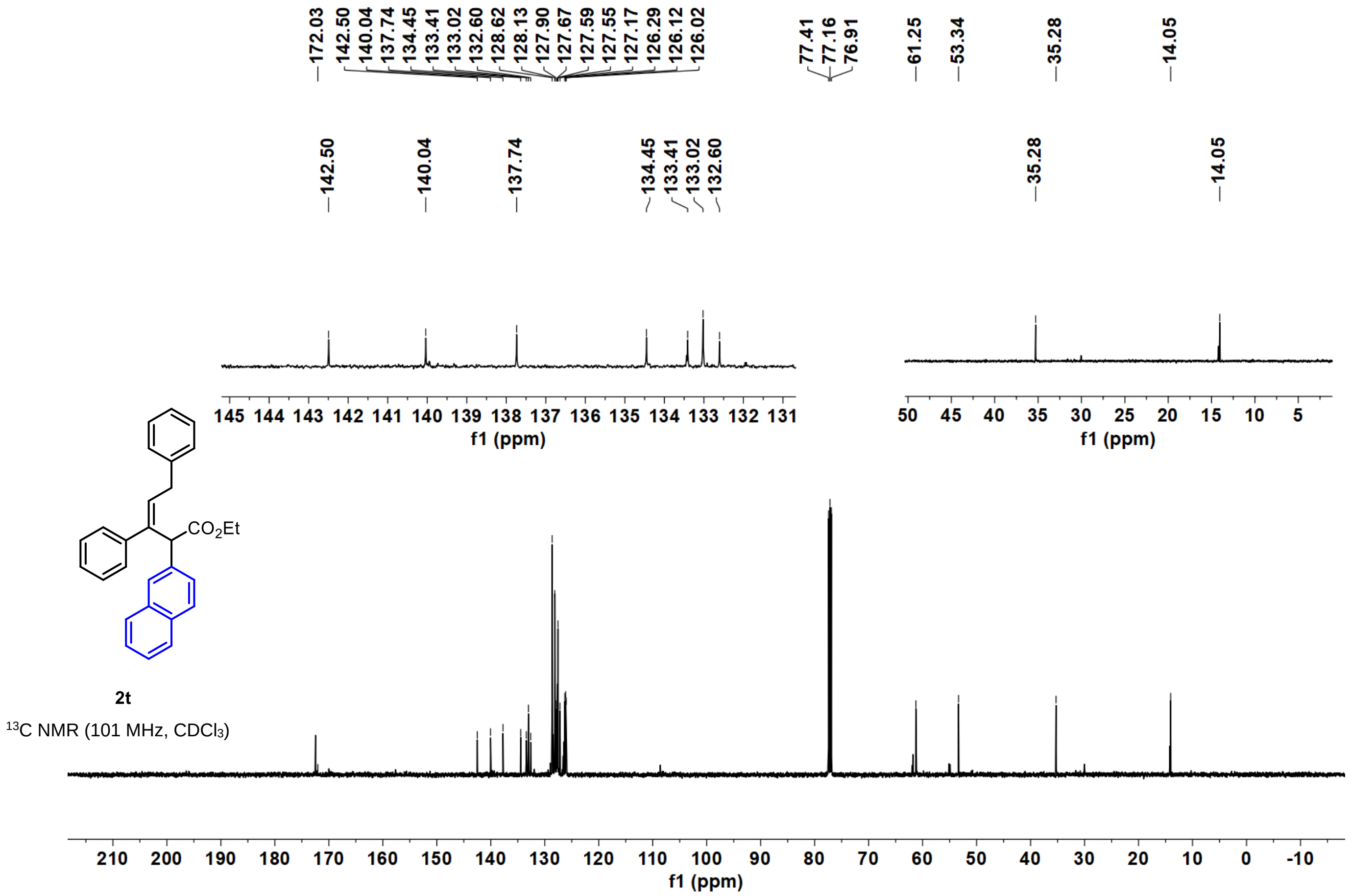

2t

^{13}C NMR (101 MHz, $CDCl_3$)

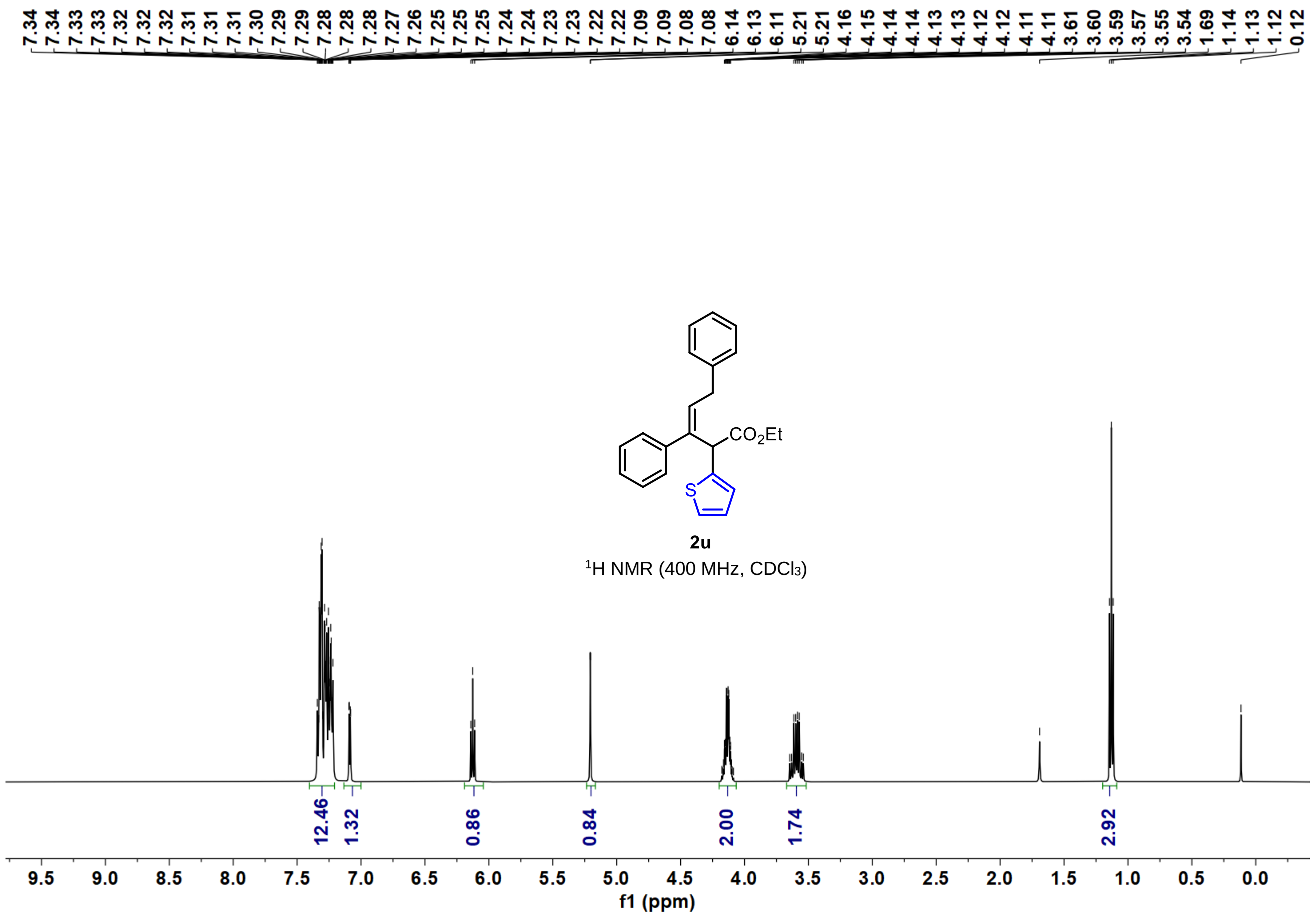

7.34 7.34 7.33 7.32 7.32 7.32 7.31 7.31 7.31 7.30 7.29 7.29 7.28 7.28 7.28 7.27 7.26 7.25 7.25 7.25 7.24 7.24 7.23 7.23 7.22 7.22 7.09 7.09 7.08 7.08 6.14 6.13 6.11 5.21 5.21 4.16 4.15 4.14 4.14 4.13 4.13 4.12 4.12 4.11 4.11 3.61 3.60 3.59 3.57 3.55 3.54 1.69 1.14 1.13 1.12 0.12
CO_2Et
S
2u
^1H NMR (400 MHz, $CDCl_3$)
12.46 1.32 0.86 0.84 2.00 1.74 2.92
9.5 9.0 8.5 8.0 7.5 7.0 6.5 6.0 5.5 5.0 4.5 4.0 3.5 3.0 2.5 2.0 1.5 1.0 0.5 0.0
f1 (ppm)

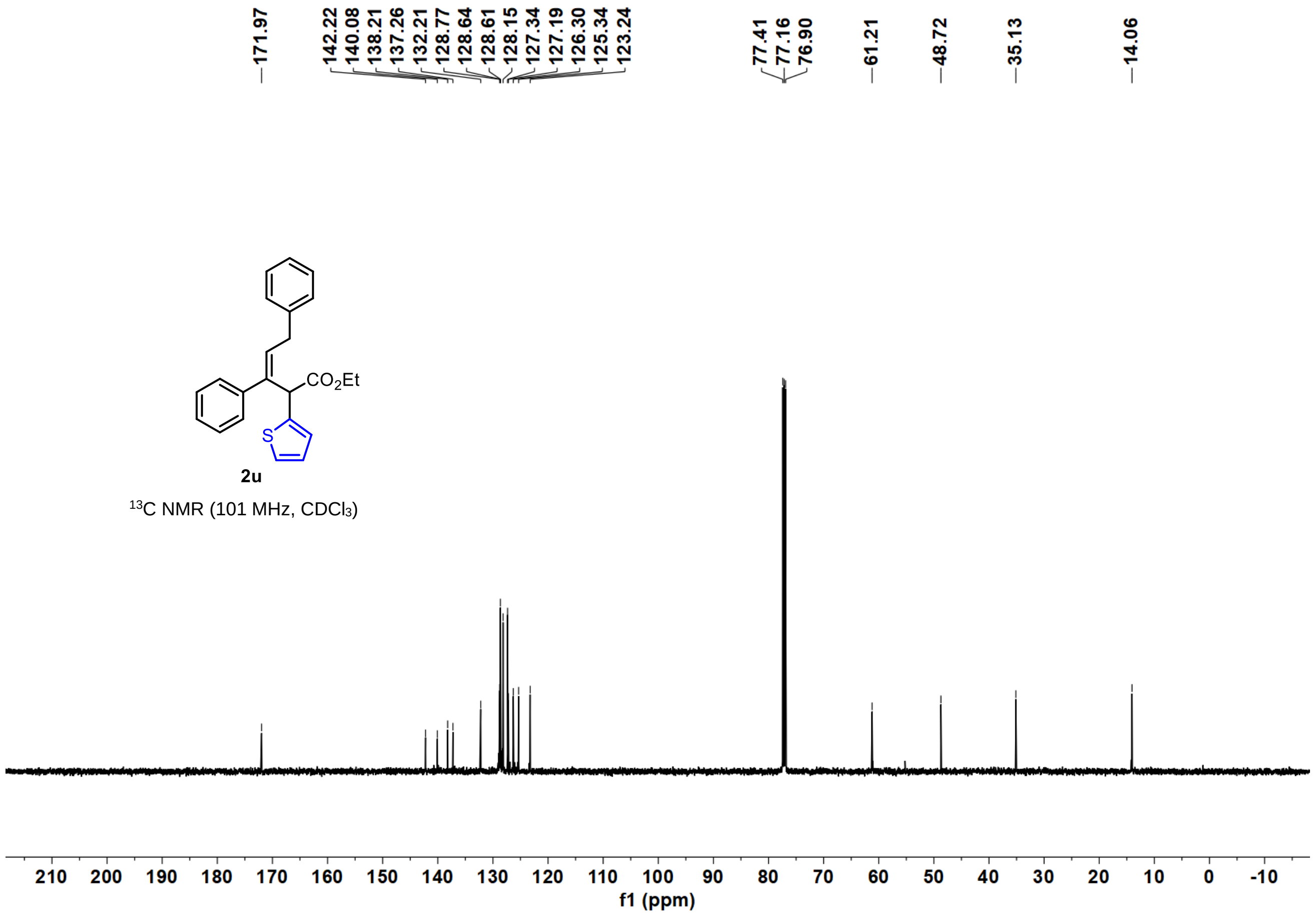

171.97
142.22 140.08 138.21 137.26 132.21 128.77 128.64 128.61 128.15 127.34 127.19 126.30 125.34 123.24
77.41 77.16 76.90
61.21
48.72
35.13
14.06
CO_2Et
S
2u
^{13}C NMR (101 MHz, $CDCl_3$)
210 200 190 180 170 160 150 140 130 120 110 100 90 80 70 60 50 40 30 20 10 0 -10
f1 (ppm)

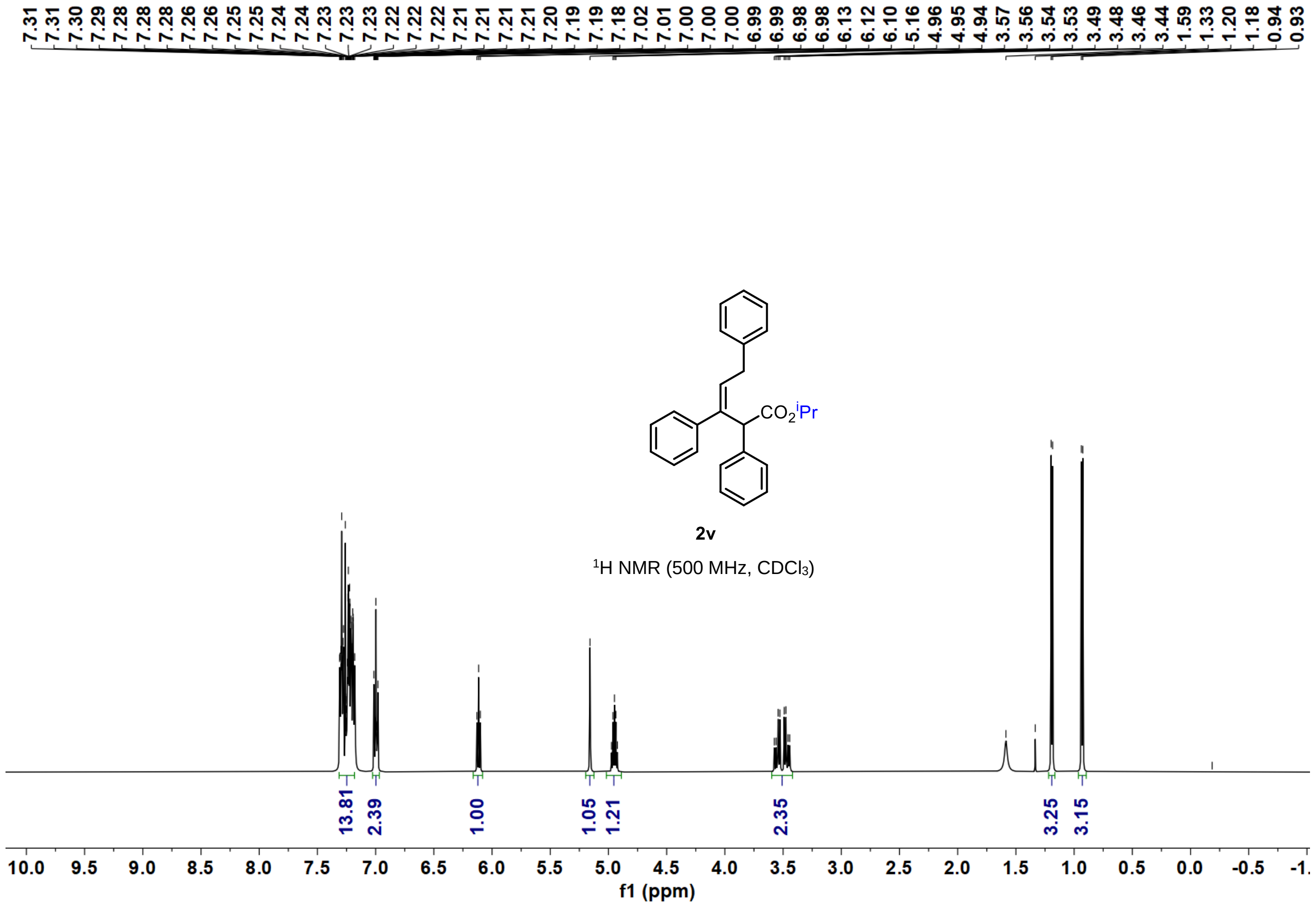

7.31 7.31 7.30 7.29 7.28 7.28 7.28 7.26 7.25 7.25 7.24 7.24 7.23 7.23 7.23 7.22 7.22 7.22 7.21 7.21 7.21 7.21 7.20 7.19 7.19 7.18 7.02 7.01 7.00 7.00 6.99 6.99 6.98 6.98 6.13 6.12 6.10 5.16 4.96 4.95 4.94 3.57 3.56 3.54 3.53 3.49 3.48 3.46 3.44 1.59 1.33 1.20 1.18 0.94 0.93
CO2iPr
2v
1H NMR (500 MHz, CDCl3)
13.81 2.39 1.00 1.05 1.21 2.35 3.25 3.15
f1 (ppm)

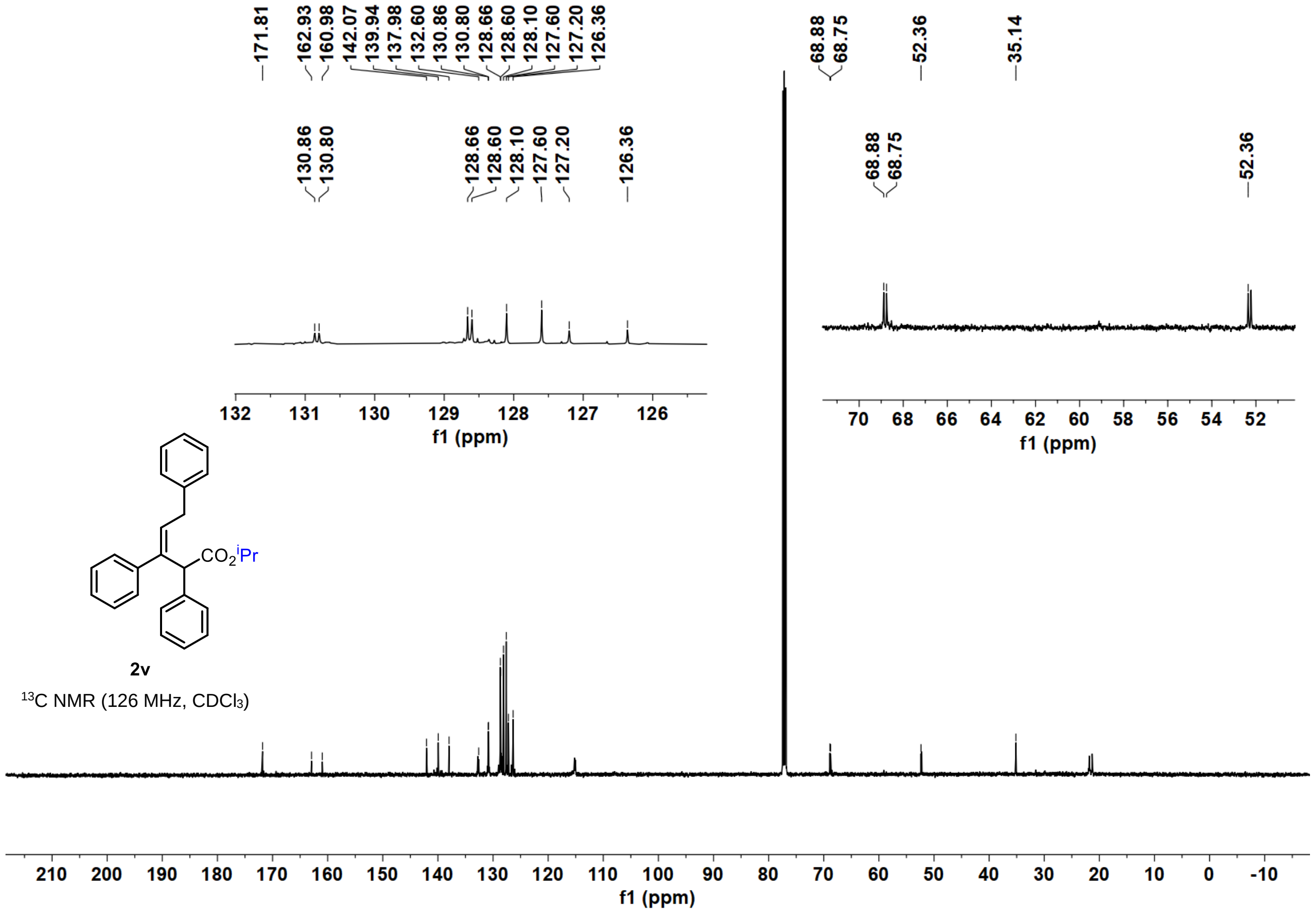

171.81 162.93 160.98 142.07 139.94 137.98 132.60 130.86 130.80 128.66 128.60 128.10 127.60 127.20 126.36
68.88 68.75 52.36 35.14
f1 (ppm)
CO2iPr
2v
13C NMR (126 MHz, CDCl3)

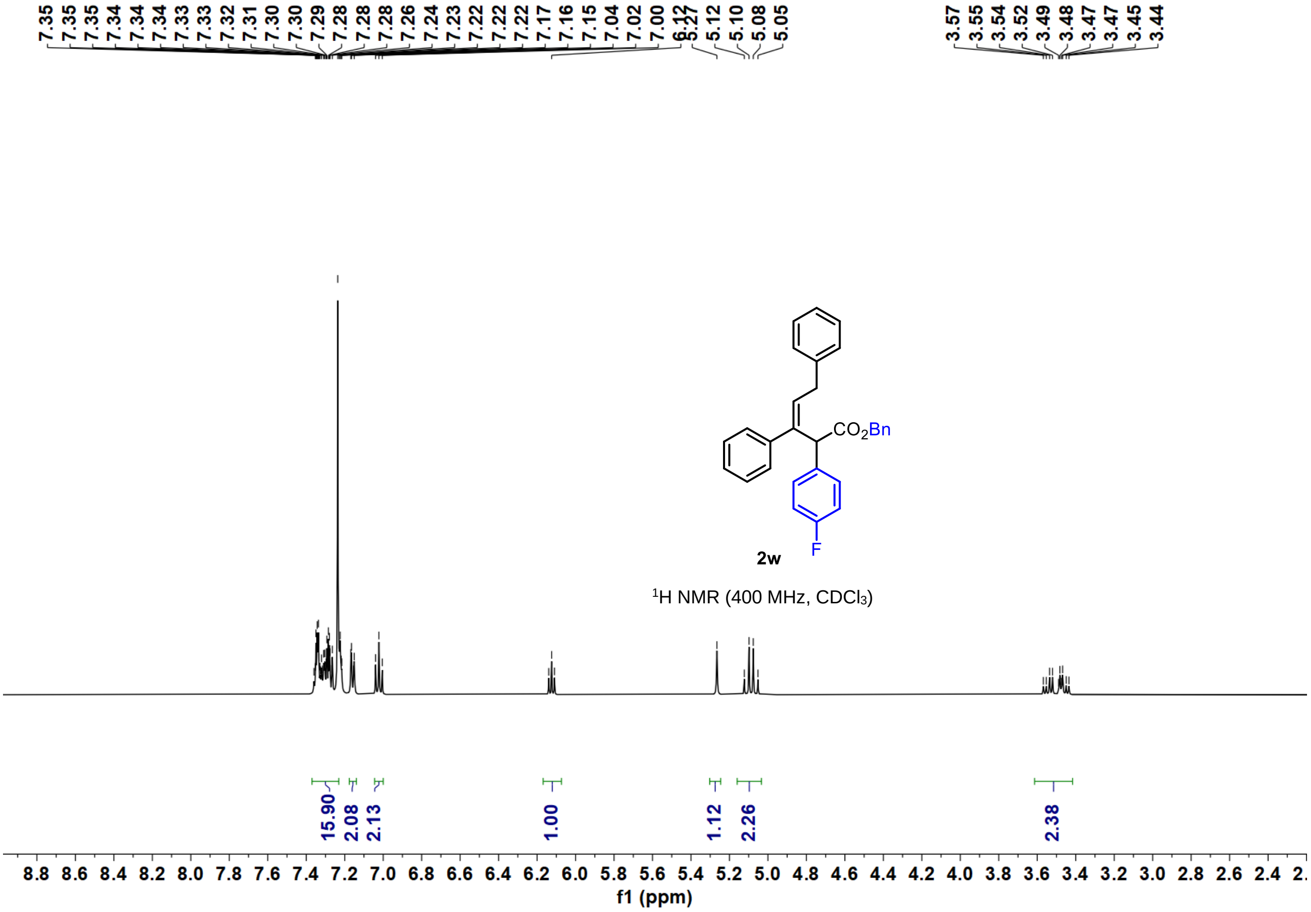
7.35 7.35 7.35 7.34 7.34 7.34 7.33 7.33 7.32 7.31 7.30 7.30 7.29 7.28 7.28 7.28 7.26 7.24 7.23 7.22 7.22 7.22 7.22 7.17 7.16 7.15 7.04 7.02 7.00 6.12 5.27 5.12 5.10 5.08 5.05
3.57 3.55 3.54 3.52 3.49 3.48 3.47 3.47 3.45 3.44
CO2Bn
F
2w
1H NMR (400 MHz, CDCl3)
15.90 2.08 2.13 1.00 1.12 2.26 2.38
8.8 8.6 8.4 8.2 8.0 7.8 7.6 7.4 7.2 7.0 6.8 6.6 6.4 6.2 6.0 5.8 5.6 5.4 5.2 5.0 4.8 4.6 4.4 4.2 4.0 3.8 3.6 3.4 3.2 3.0 2.8 2.6 2.4 2.
f1 (ppm)

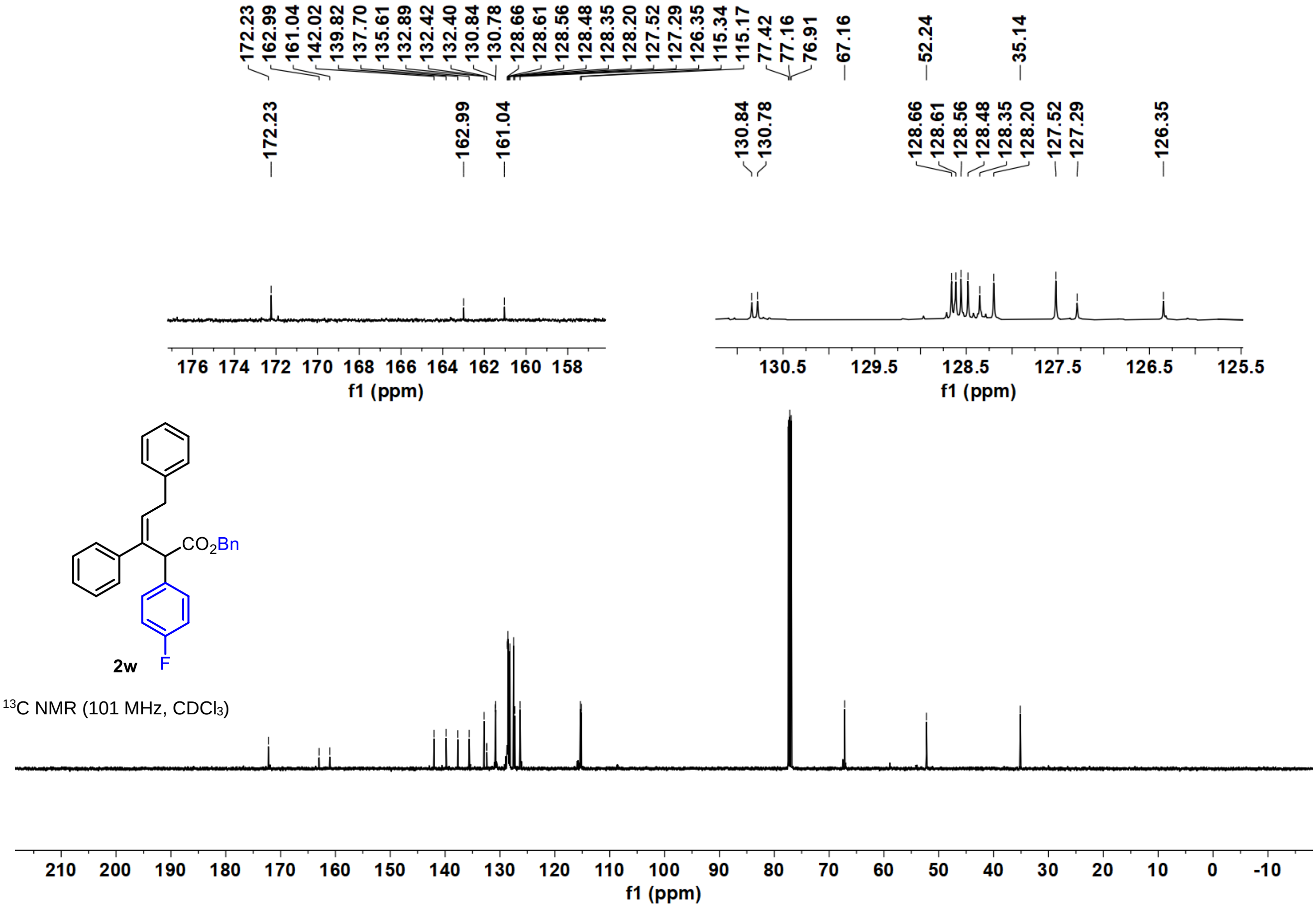
172.23 162.99 161.04 142.02 139.82 137.70 135.61 132.89 132.42 132.40 130.84 130.78 128.66 128.61 128.56 128.48 128.35 128.20 127.52 127.29 126.35 115.34 115.17 77.42 77.16 76.91 67.16 52.24 35.14
172.23 162.99 161.04
130.84 130.78 128.66 128.61 128.56 128.48 128.35 128.20 127.52 127.29 126.35
176 174 172 170 168 166 164 162 160 158
f1 (ppm)
130.5 129.5 128.5 127.5 126.5 125.5
f1 (ppm)
CO2Bn
F
2w
13C NMR (101 MHz, CDCl3)
210 200 190 180 170 160 150 140 130 120 110 100 90 80 70 60 50 40 30 20 10 0 -10
f1 (ppm)

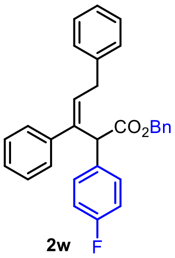

2w

^{19}F NMR (377 MHz, $CDCl_3$)

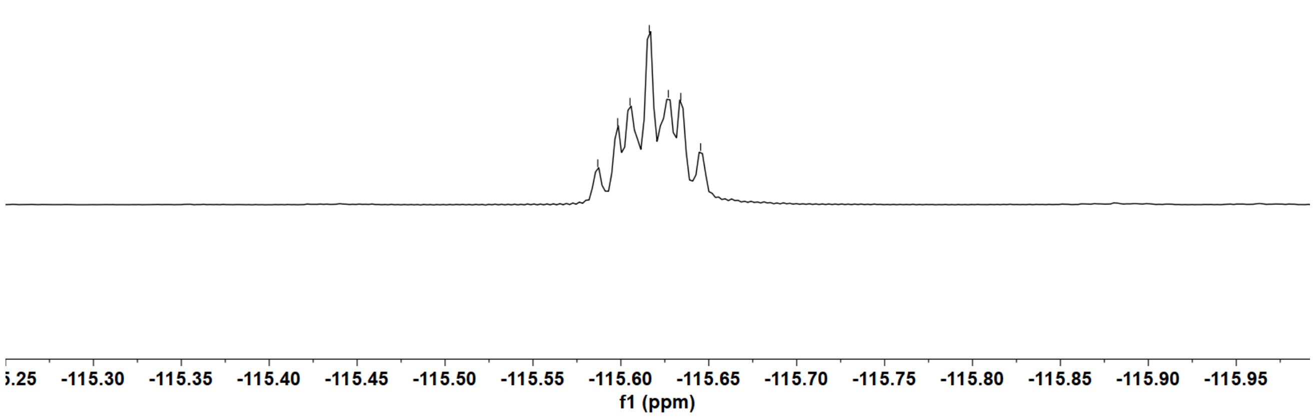

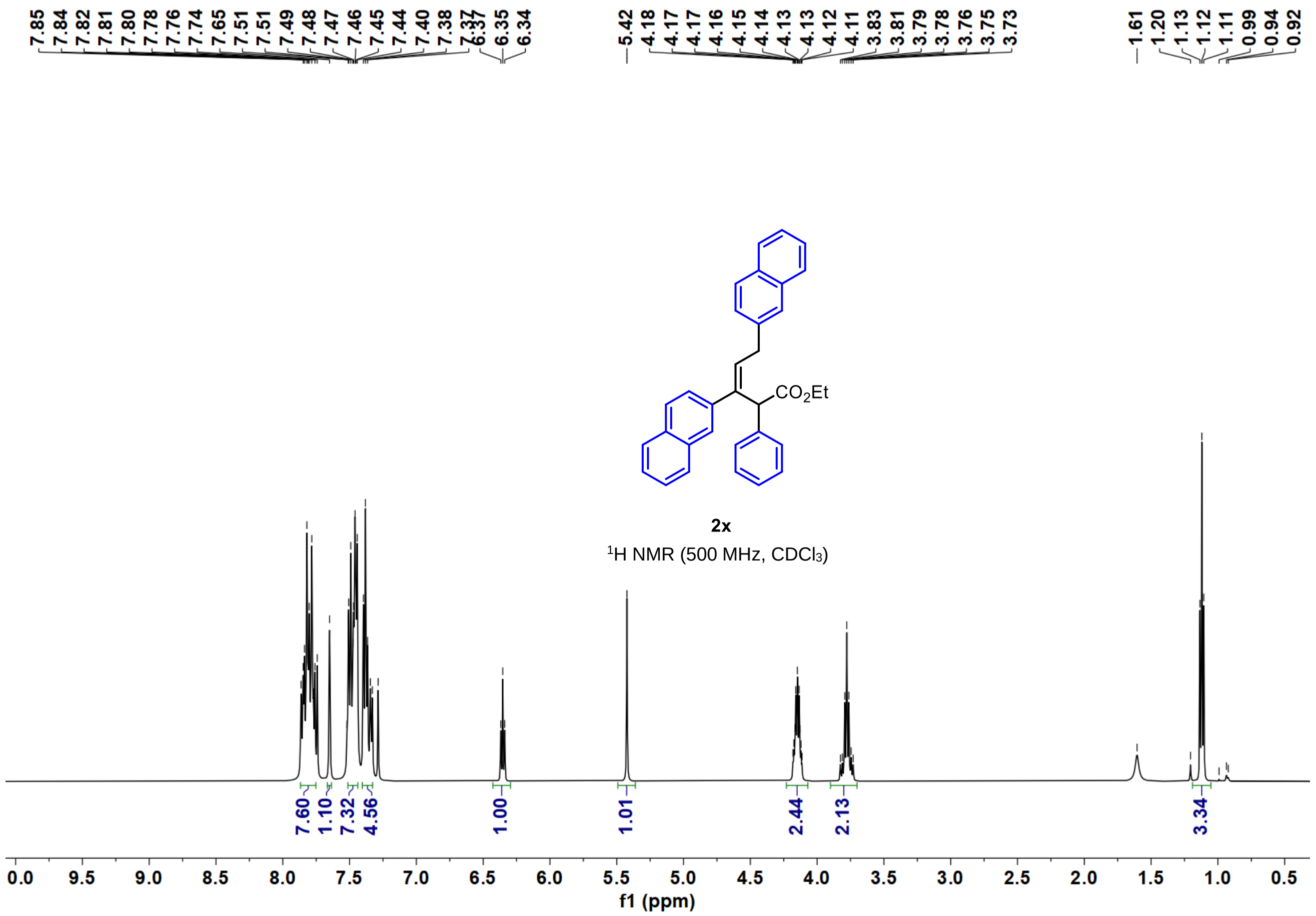
7.85 7.84 7.82 7.81 7.80 7.78 7.76 7.74 7.65 7.51 7.51 7.49 7.48 7.47 7.46 7.45 7.44 7.40 7.38 7.37 6.37 6.35 6.34
5.42 4.18 4.17 4.17 4.16 4.15 4.14 4.13 4.13 4.12 4.11 3.83 3.81 3.79 3.78 3.76 3.75 3.73
1.61 1.20 1.13 1.12 1.11 0.99 0.94 0.92
CO2Et
2x
1H NMR (500 MHz, CDCl3)
7.60 1.10 7.32 4.56 1.00 1.01 2.44 2.13 3.34
0.0 9.5 9.0 8.5 8.0 7.5 7.0 6.5 6.0 5.5 5.0 4.5 4.0 3.5 3.0 2.5 2.0 1.5 1.0 0.5
f1 (ppm)

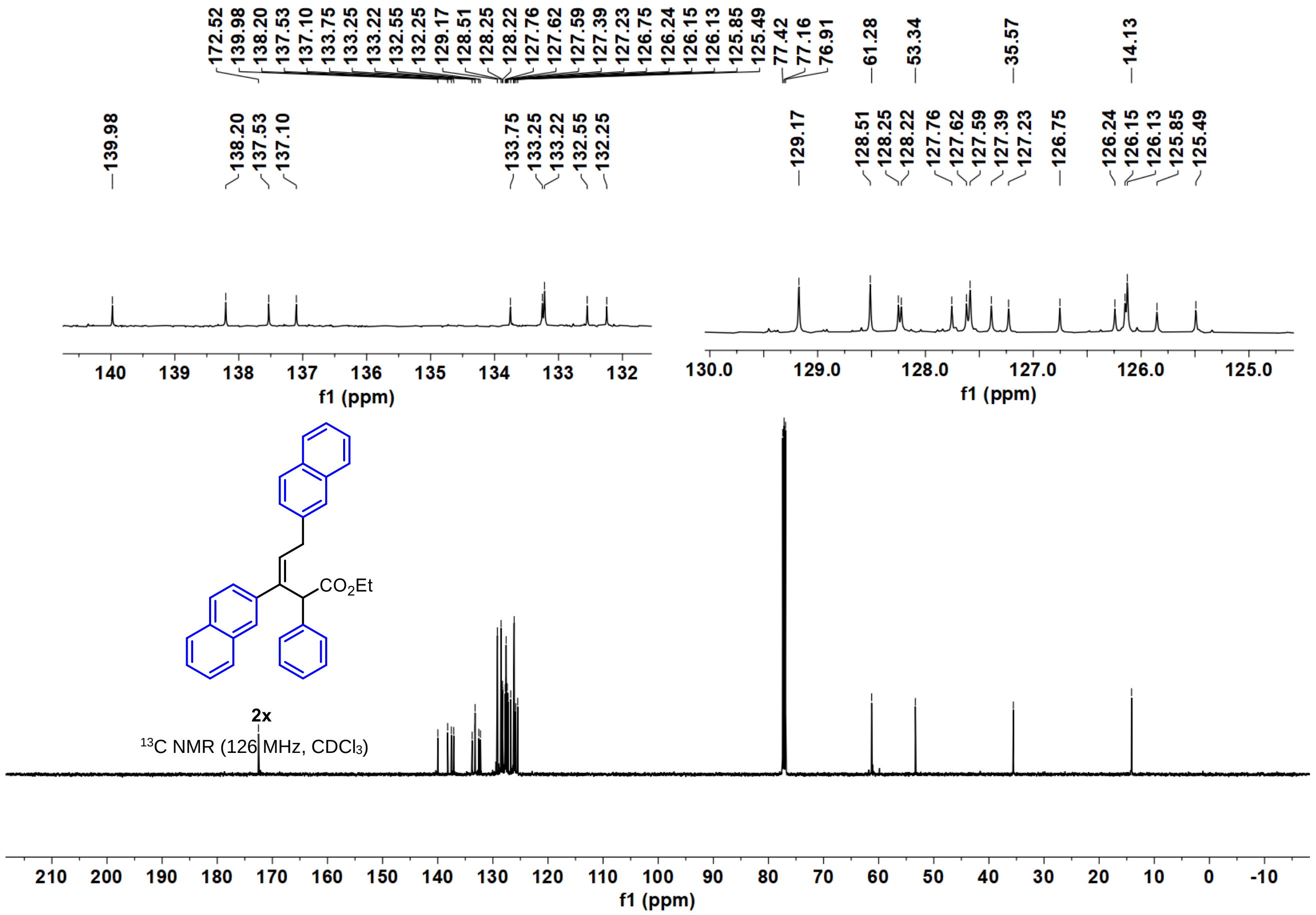
172.52 139.98 138.20 137.53 137.10 133.75 133.25 133.22 132.55 132.25 129.17 128.51 128.25 128.22 127.76 127.62 127.59 127.39 127.23 126.75 126.24 126.15 126.13 125.85 125.49 77.42 77.16 76.91 61.28 53.34 35.57 14.13
139.98 138.20 137.53 137.10 133.75 133.25 133.22 132.55 132.25
129.17 128.51 128.25 128.22 127.76 127.62 127.59 127.39 127.23 126.75 126.24 126.15 126.13 125.85 125.49
140 139 138 137 136 135 134 133 132
f1 (ppm)
130.0 129.0 128.0 127.0 126.0 125.0
f1 (ppm)
CO2Et
2x
13C NMR (126 MHz, CDCl3)
210 200 190 180 170 160 150 140 130 120 110 100 90 80 70 60 50 40 30 20 10 0 -10
f1 (ppm)

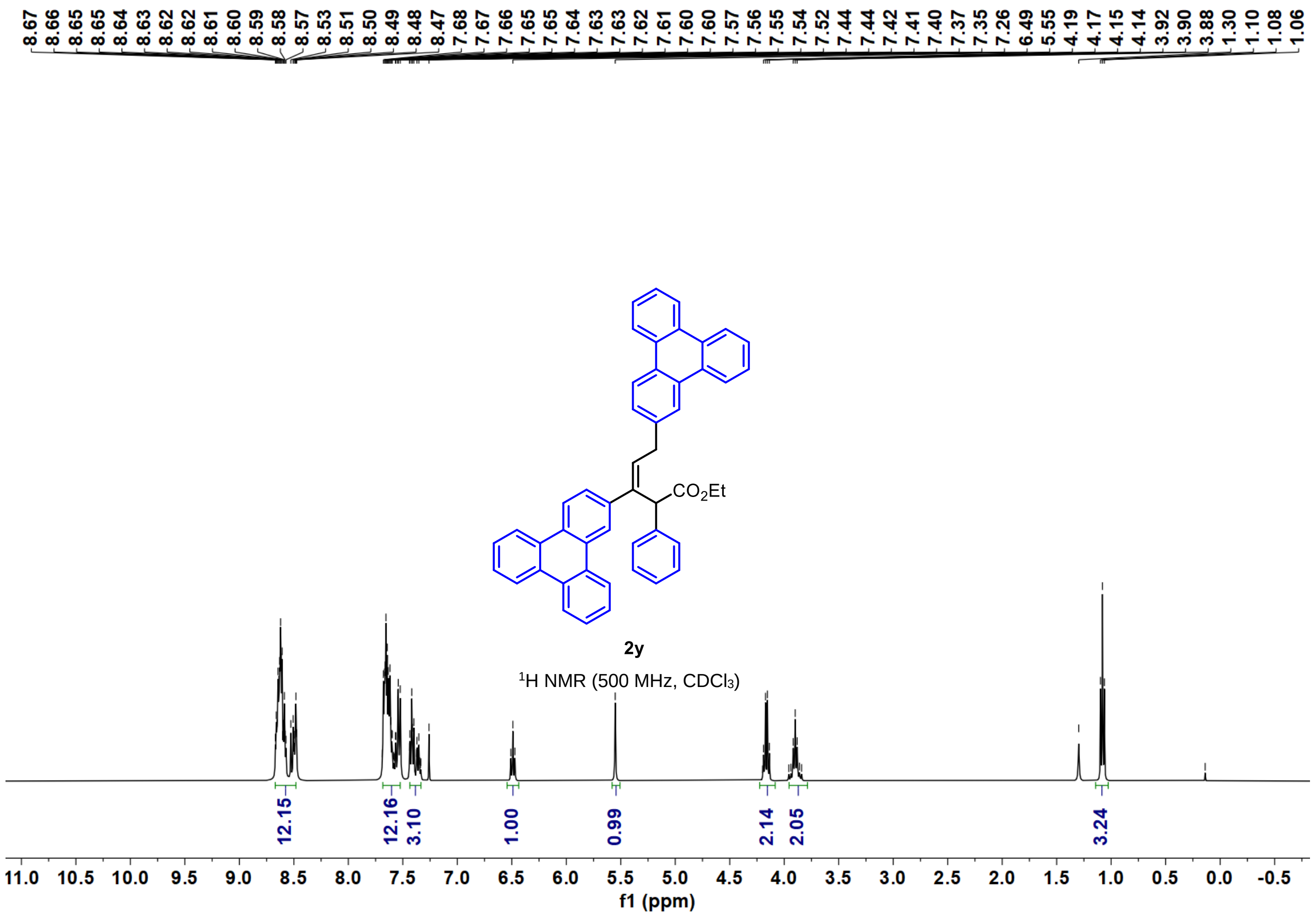
8.67 8.66 8.65 8.64 8.63 8.62 8.62 8.61 8.60 8.59 8.58 8.57 8.53 8.51 8.50 8.49 8.48 8.47 7.68 7.67 7.66 7.65 7.65 7.64 7.63 7.63 7.62 7.61 7.60 7.60 7.57 7.56 7.55 7.54 7.52 7.44 7.44 7.42 7.41 7.40 7.37 7.35 7.26 6.49 5.55 4.19 4.17 4.15 4.14 3.92 3.90 3.88 1.30 1.10 1.08 1.06
CO2Et
2y
1H NMR (500 MHz, CDCl3)
12.15 12.16 3.10 1.00 0.99 2.14 2.05 3.24
f1 (ppm)

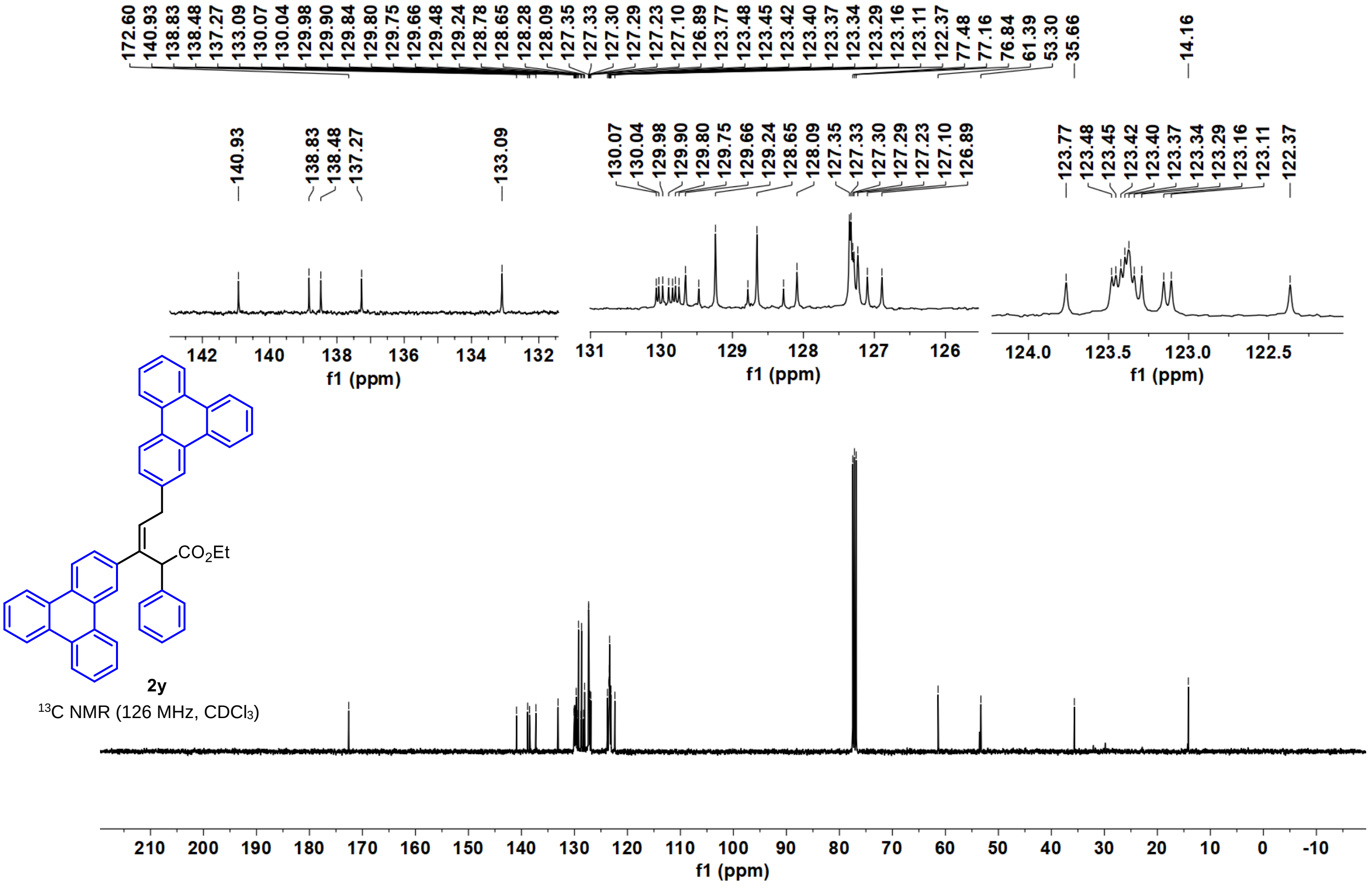
172.60 140.93 138.83 138.48 137.27 133.09 130.07 130.04 129.98 129.90 129.84 129.80 129.75 129.66 129.48 129.24 128.78 128.65 128.28 128.09 127.35 127.33 127.30 127.29 127.23 127.10 126.89 123.77 123.48 123.45 123.42 123.40 123.37 123.34 123.29 123.16 123.11 122.37 77.48 77.16 76.84 61.39 53.30 35.66 14.16
f1 (ppm)
CO2Et
2y
13C NMR (126 MHz, CDCl3)

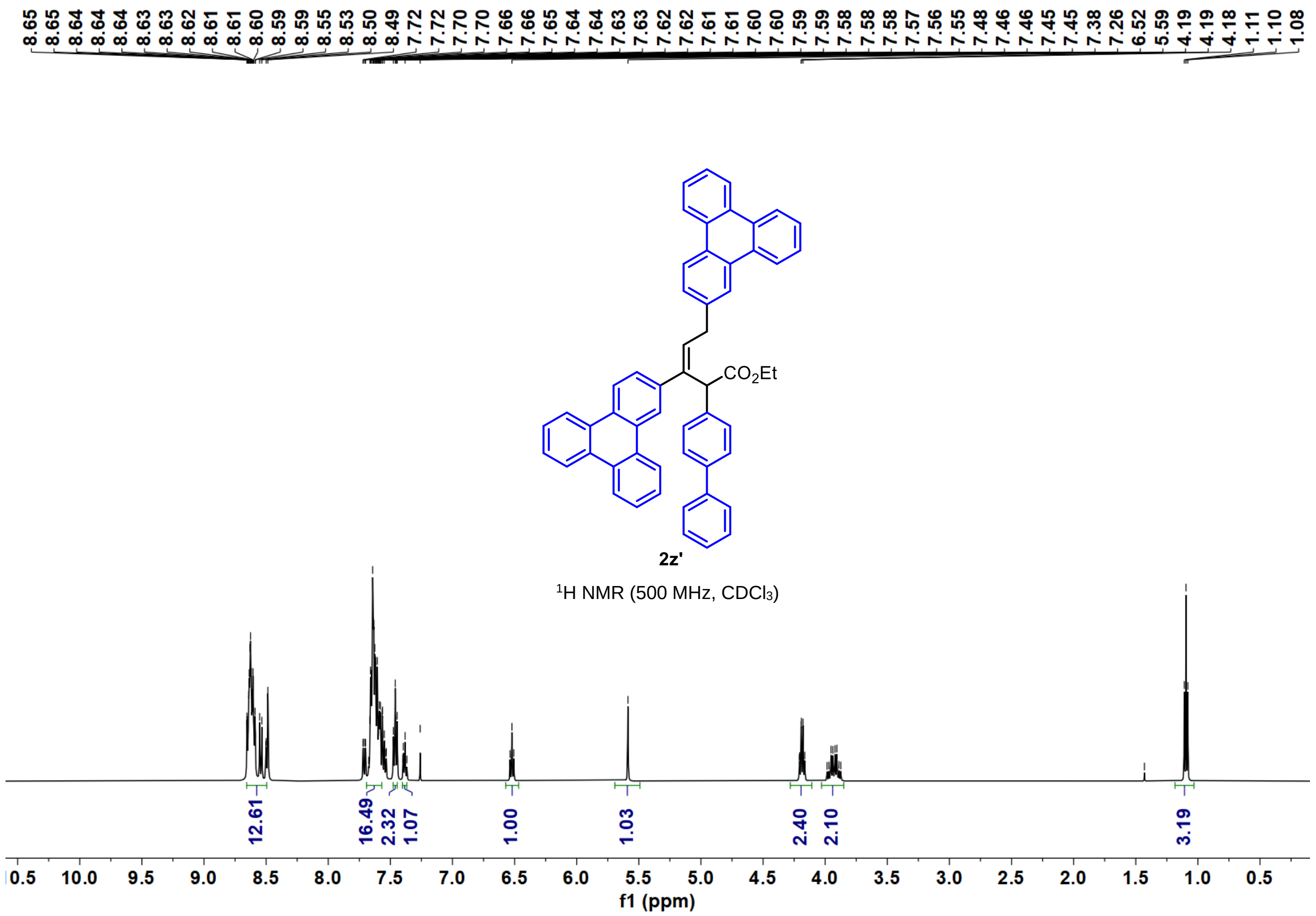

8.65 8.65 8.64 8.64 8.64 8.63 8.63 8.62 8.61 8.61 8.60 8.59 8.59 8.55 8.53 8.50 8.49 7.72 7.72 7.70 7.70 7.66 7.66 7.65 7.64 7.64 7.63 7.63 7.62 7.62 7.61 7.61 7.60 7.60 7.59 7.59 7.58 7.58 7.58 7.57 7.56 7.55 7.48 7.46 7.46 7.45 7.45 7.38 7.26 6.52 5.59 4.19 4.19 4.18 1.11 1.10 1.08
CO2Et
2z'
1H NMR (500 MHz, CDCl3)
12.61 16.49 2.32 1.07 1.00 1.03 2.40 2.10 3.19
f1 (ppm)

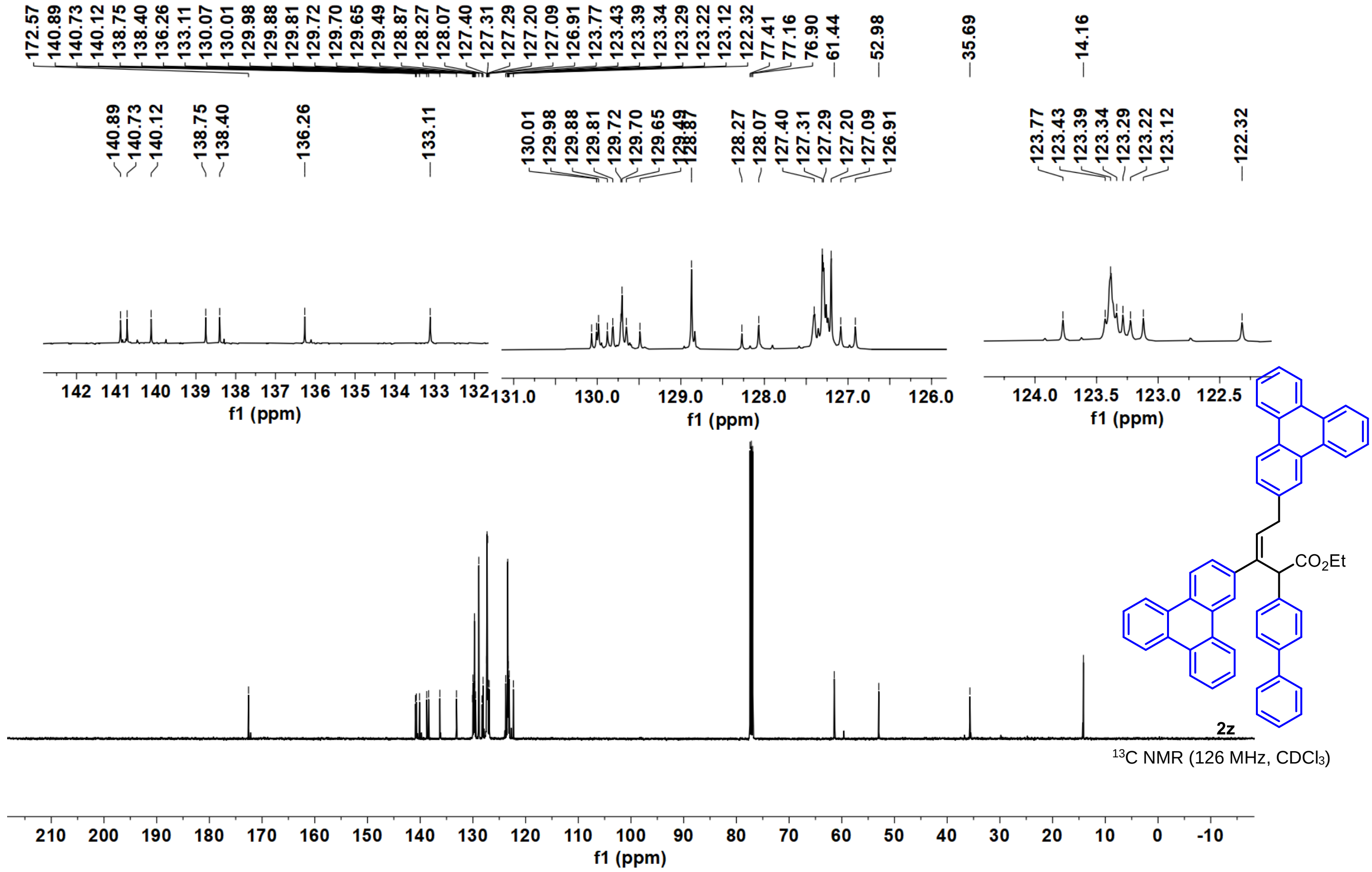

172.57 140.89 140.73 140.12 138.75 138.40 136.26 133.11 130.07 130.01 129.98 129.88 129.81 129.72 129.70 129.65 129.49 128.87 128.27 128.07 127.40 127.31 127.29 127.20 127.09 126.91 123.77 123.43 123.39 123.34 123.29 123.22 123.12 122.32 77.41 77.16 76.90 61.44 52.98 35.69 14.16
f1 (ppm)
CO2Et
2z
13C NMR (126 MHz, CDCl3)

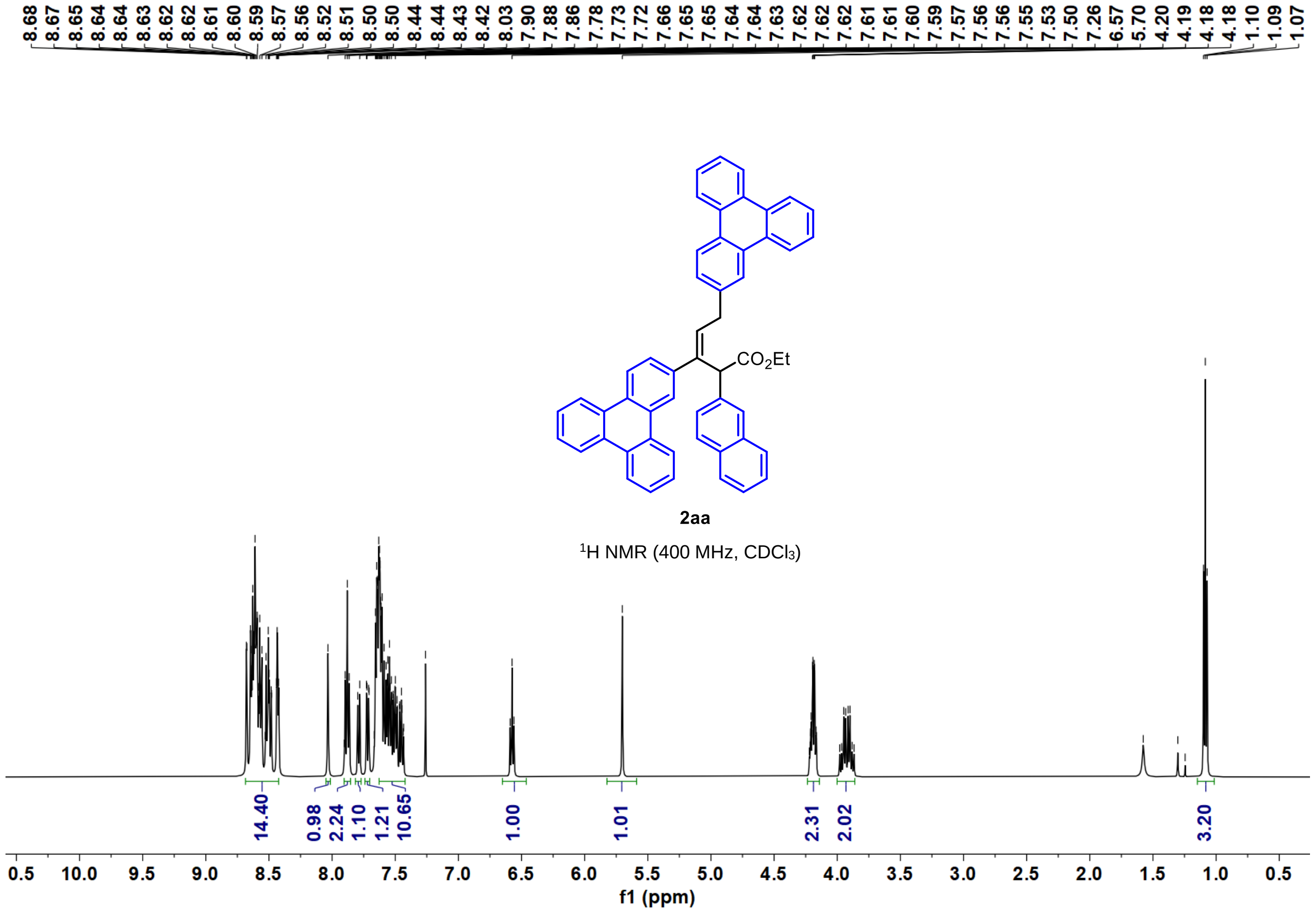
8.68 8.67 8.65 8.64 8.64 8.63 8.62 8.62 8.61 8.60 8.59 8.57 8.56 8.52 8.51 8.50 8.50 8.44 8.44 8.43 8.42 8.03 7.90 7.88 7.86 7.78 7.73 7.72 7.66 7.65 7.65 7.64 7.64 7.63 7.62 7.62 7.62 7.61 7.61 7.60 7.59 7.57 7.56 7.56 7.55 7.53 7.50 7.26 6.57 5.70 4.20 4.19 4.18 4.18 1.10 1.09 1.07
CO2Et
2aa
1H NMR (400 MHz, CDCl3)
14.40 0.98 2.24 1.10 1.21 10.65 1.00 1.01 2.31 2.02 3.20
0.5 10.0 9.5 9.0 8.5 8.0 7.5 7.0 6.5 6.0 5.5 5.0 4.5 4.0 3.5 3.0 2.5 2.0 1.5 1.0 0.5
f1 (ppm)

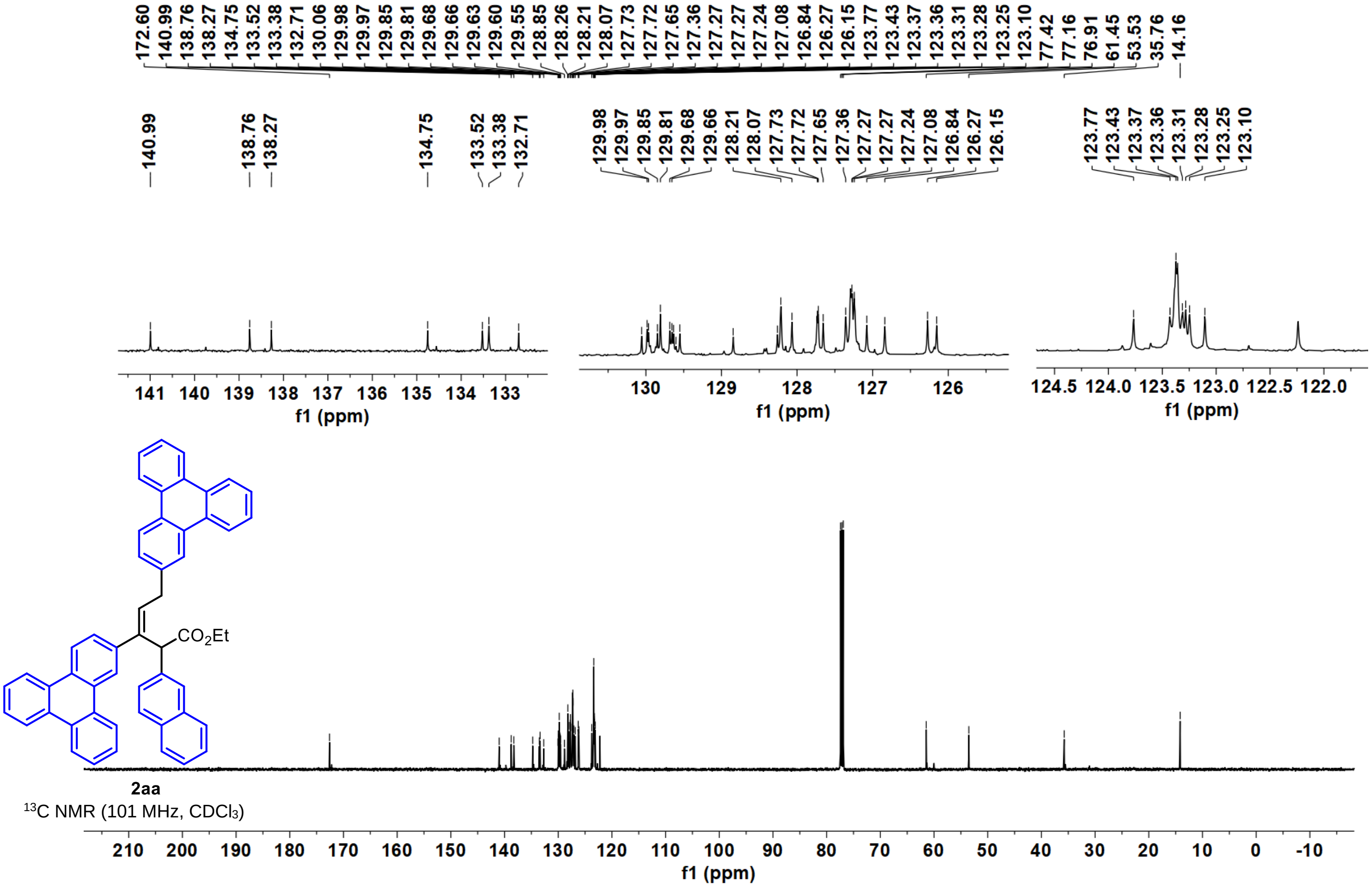
172.60 140.99 138.76 138.27 134.75 133.52 133.38 132.71 130.06 129.98 129.97 129.85 129.81 129.68 129.66 129.63 129.60 129.55 128.85 128.26 128.21 128.07 127.73 127.72 127.65 127.36 127.27 127.27 127.24 127.08 126.84 126.27 126.15 123.77 123.43 123.37 123.36 123.31 123.28 123.25 123.10 77.42 77.16 76.91 61.45 53.53 35.76 14.16
140.99 138.76 138.27 134.75 133.52 133.38 132.71
141 140 139 138 137 136 135 134 133
f1 (ppm)
129.98 129.97 129.85 129.81 129.68 129.66 128.21 128.07 127.73 127.72 127.65 127.36 127.27 127.27 127.24 127.08 126.84 126.27 126.15
130 129 128 127 126
f1 (ppm)
123.77 123.43 123.37 123.36 123.31 123.28 123.25 123.10
124.5 124.0 123.5 123.0 122.5 122.0
f1 (ppm)
CO2Et
2aa
13C NMR (101 MHz, CDCl3)
210 200 190 180 170 160 150 140 130 120 110 100 90 80 70 60 50 40 30 20 10 0 -10
f1 (ppm)

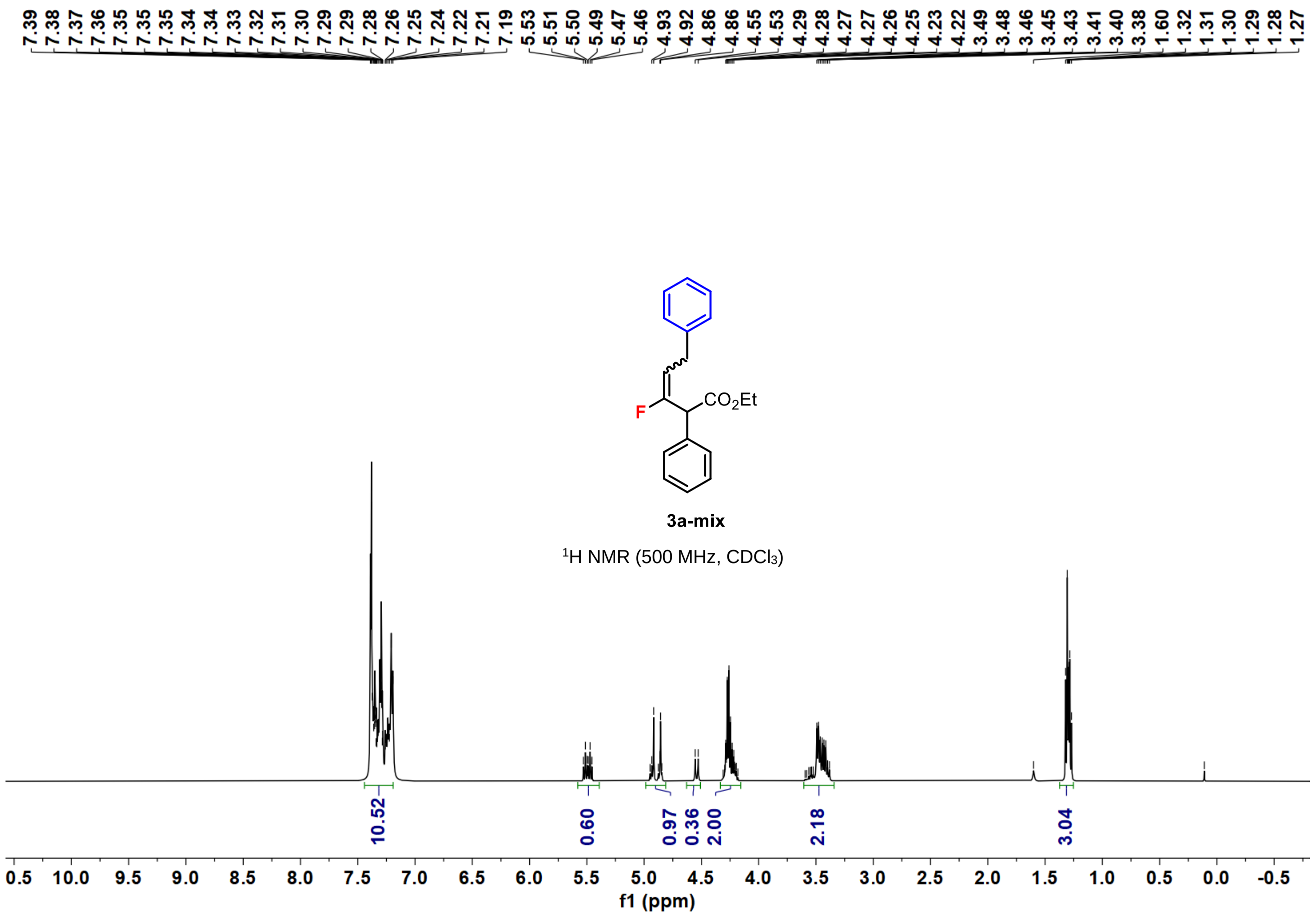
7.39 7.38 7.37 7.36 7.35 7.35 7.35 7.34 7.34 7.33 7.32 7.31 7.30 7.29 7.29 7.28 7.26 7.25 7.24 7.22 7.21 7.19 5.53 5.51 5.50 5.49 5.47 5.46 4.93 4.92 4.86 4.55 4.53 4.29 4.28 4.27 4.27 4.26 4.25 4.23 4.22 3.49 3.48 3.46 3.45 3.43 3.41 3.40 3.38 1.60 1.32 1.31 1.30 1.29 1.28 1.27
F
CO_2Et
3a-mix
^{1}H NMR (500 MHz, CDCl3)
10.52
0.60
0.97
0.36
2.00
2.18
3.04
0.5 10.0 9.5 9.0 8.5 8.0 7.5 7.0 6.5 6.0 5.5 5.0 4.5 4.0 3.5 3.0 2.5 2.0 1.5 1.0 0.5 0.0 -0.5
f1 (ppm)

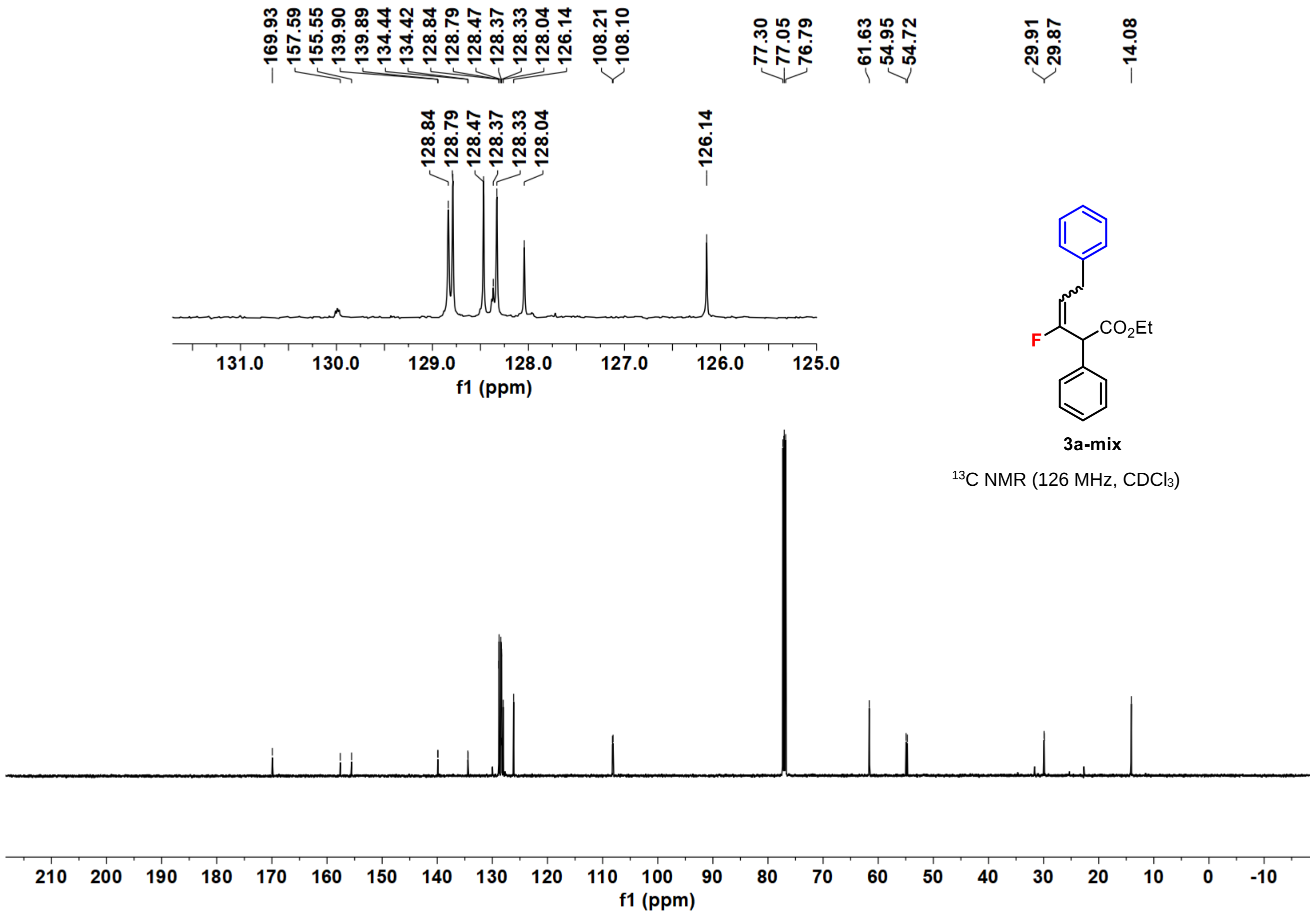
169.93 157.59 155.55 139.90 139.89 134.44 134.42 128.84 128.79 128.47 128.37 128.33 128.04 126.14 108.21 108.10 77.30 77.05 76.79 61.63 54.95 54.72 29.91 29.87 14.08
128.84 128.79 128.47 128.37 128.33 128.04
126.14
131.0 130.0 129.0 128.0 127.0 126.0 125.0
f1 (ppm)
F
CO_2Et
3a-mix
^{13}C NMR (126 MHz, CDCl3)
210 200 190 180 170 160 150 140 130 120 110 100 90 80 70 60 50 40 30 20 10 0 -10
f1 (ppm)

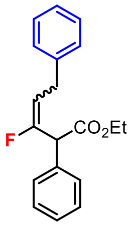

3a-mix
dr= 1.2:1
^{19}F NMR (471 MHz, $CDCl_3$)

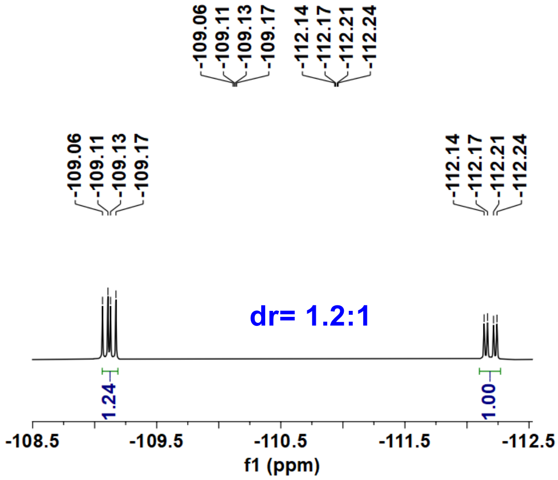

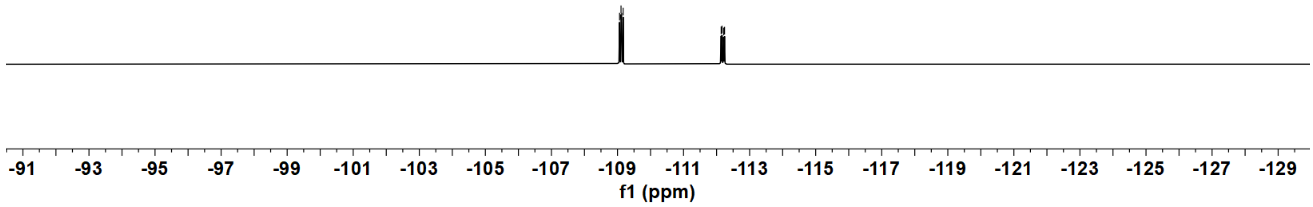

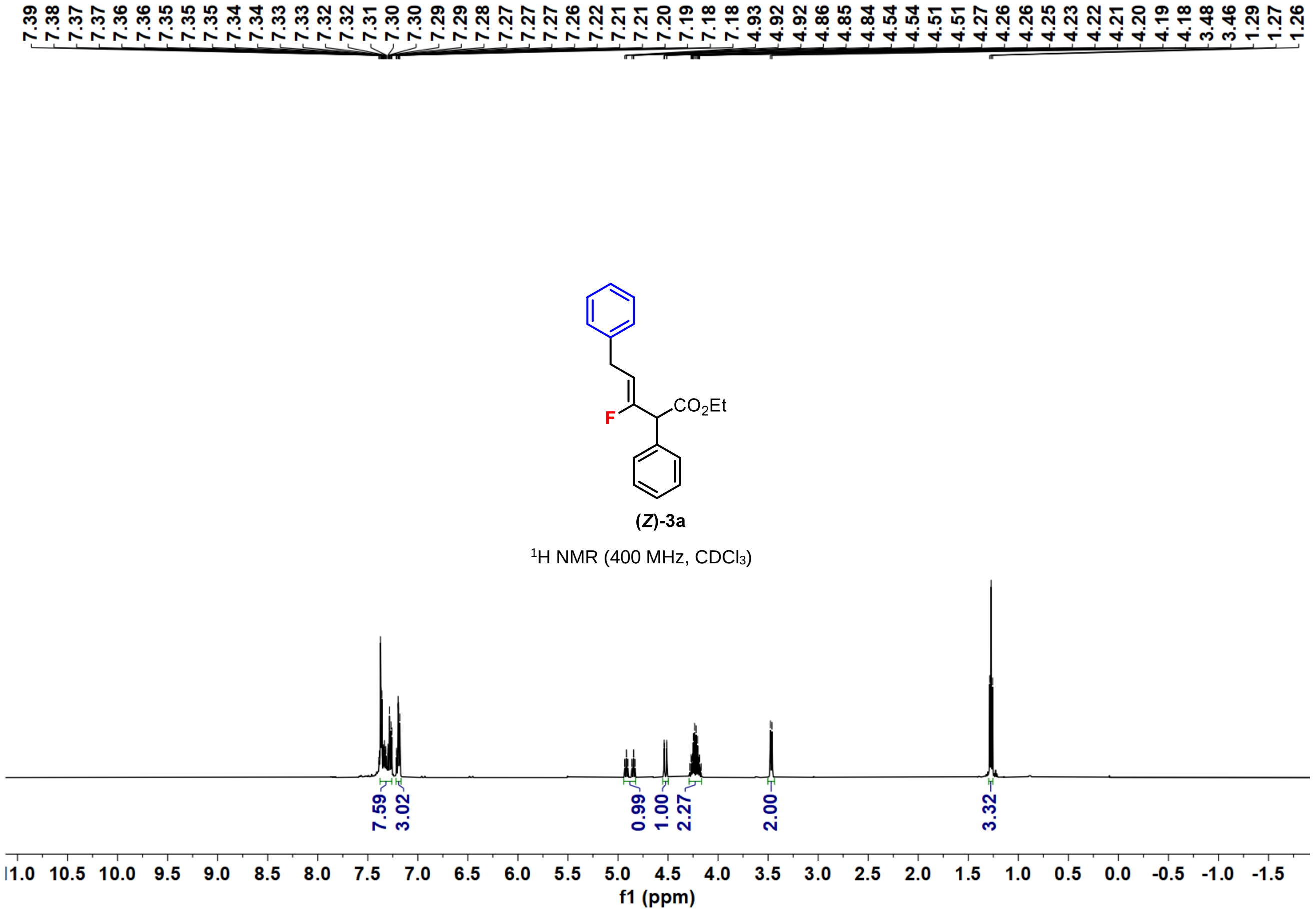
7.39 7.38 7.37 7.37 7.36 7.36 7.35 7.35 7.35 7.34 7.34 7.33 7.33 7.32 7.32 7.31 7.30 7.30 7.29 7.29 7.28 7.27 7.27 7.27 7.26 7.22 7.21 7.21 7.20 7.19 7.18 7.18 4.93 4.92 4.92 4.86 4.85 4.84 4.54 4.54 4.51 4.51 4.27 4.26 4.26 4.25 4.23 4.22 4.21 4.20 4.19 4.18 3.48 3.46 1.29 1.27 1.26
CO2Et
F
(Z)-3a
1H NMR (400 MHz, CDCl3)
7.59 3.02 0.99 1.00 2.27 2.00 3.32
11.0 10.5 10.0 9.5 9.0 8.5 8.0 7.5 7.0 6.5 6.0 5.5 5.0 4.5 4.0 3.5 3.0 2.5 2.0 1.5 1.0 0.5 0.0 -0.5 -1.0 -1.5
f1 (ppm)

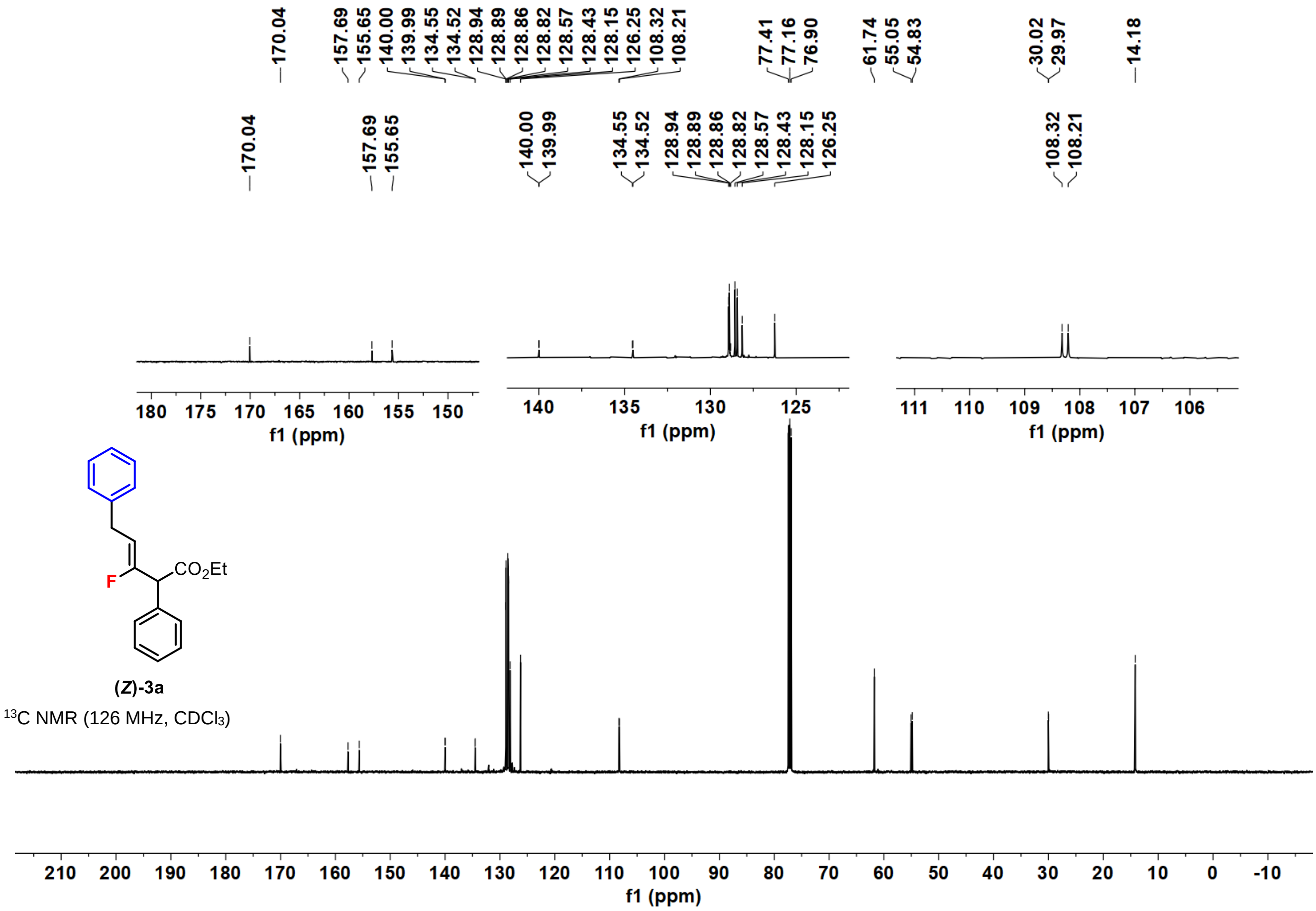
170.04 157.69 155.65 140.00 139.99 134.55 134.52 128.94 128.89 128.86 128.82 128.57 128.43 128.15 126.25 108.32 108.21 77.41 77.16 76.90 61.74 55.05 54.83 30.02 29.97 14.18
170.04 157.69 155.65
140.00 139.99 134.55 134.52 128.94 128.89 128.86 128.82 128.57 128.43 128.15 126.25
108.32 108.21
180 175 170 165 160 155 150
f1 (ppm)
140 135 130 125
f1 (ppm)
111 110 109 108 107 106
f1 (ppm)
CO2Et
F
(Z)-3a
13C NMR (126 MHz, CDCl3)
210 200 190 180 170 160 150 140 130 120 110 100 90 80 70 60 50 40 30 20 10 0 -10
f1 (ppm)

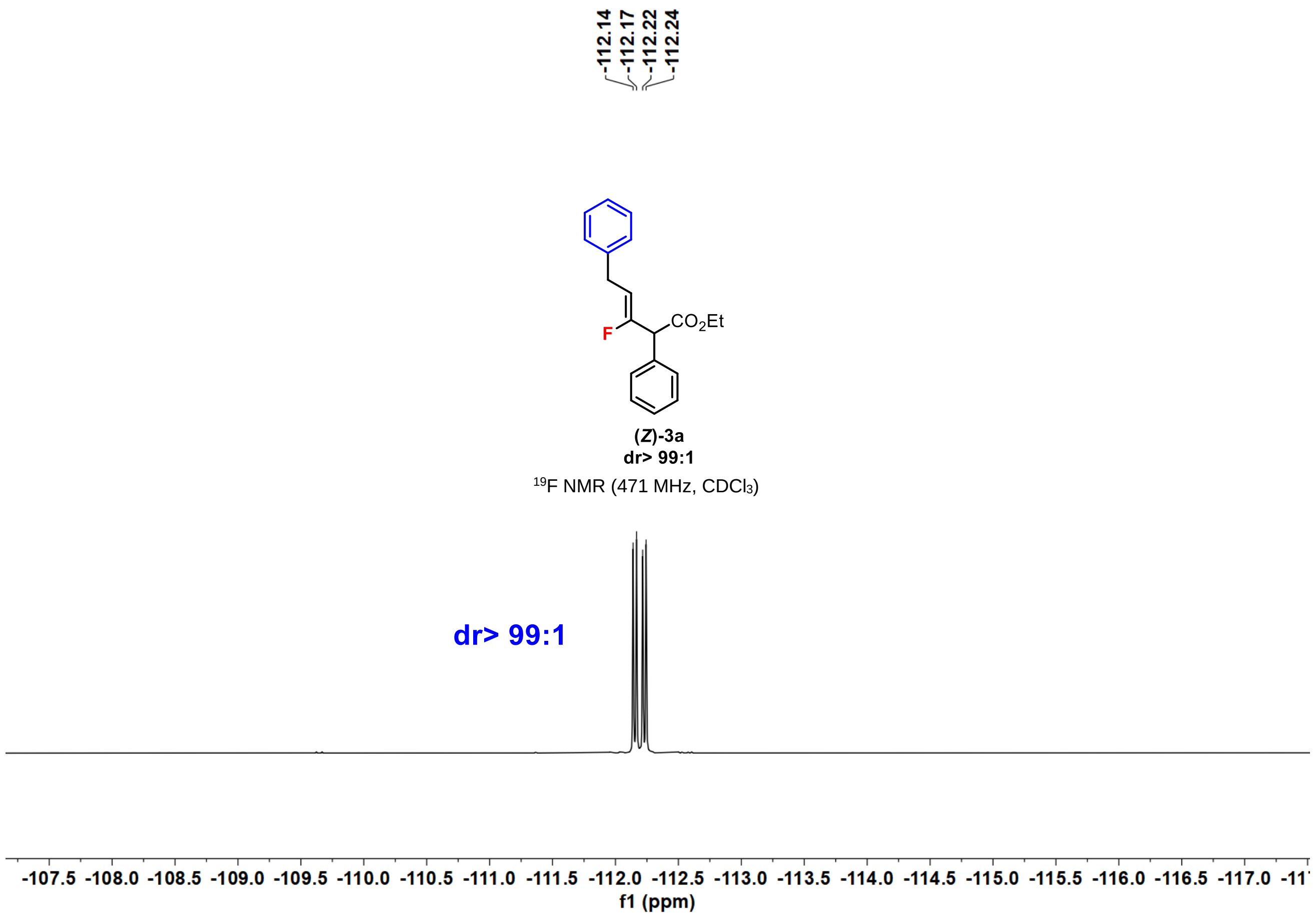

-112.14
-112.17
-112.22
-112.24
F
CO2Et
(Z)-3a
dr> 99:1
19F NMR (471 MHz, CDCl3)
dr> 99:1
-107.5 -108.0 -108.5 -109.0 -109.5 -110.0 -110.5 -111.0 -111.5 -112.0 -112.5 -113.0 -113.5 -114.0 -114.5 -115.0 -115.5 -116.0 -116.5 -117.0
f1 (ppm)

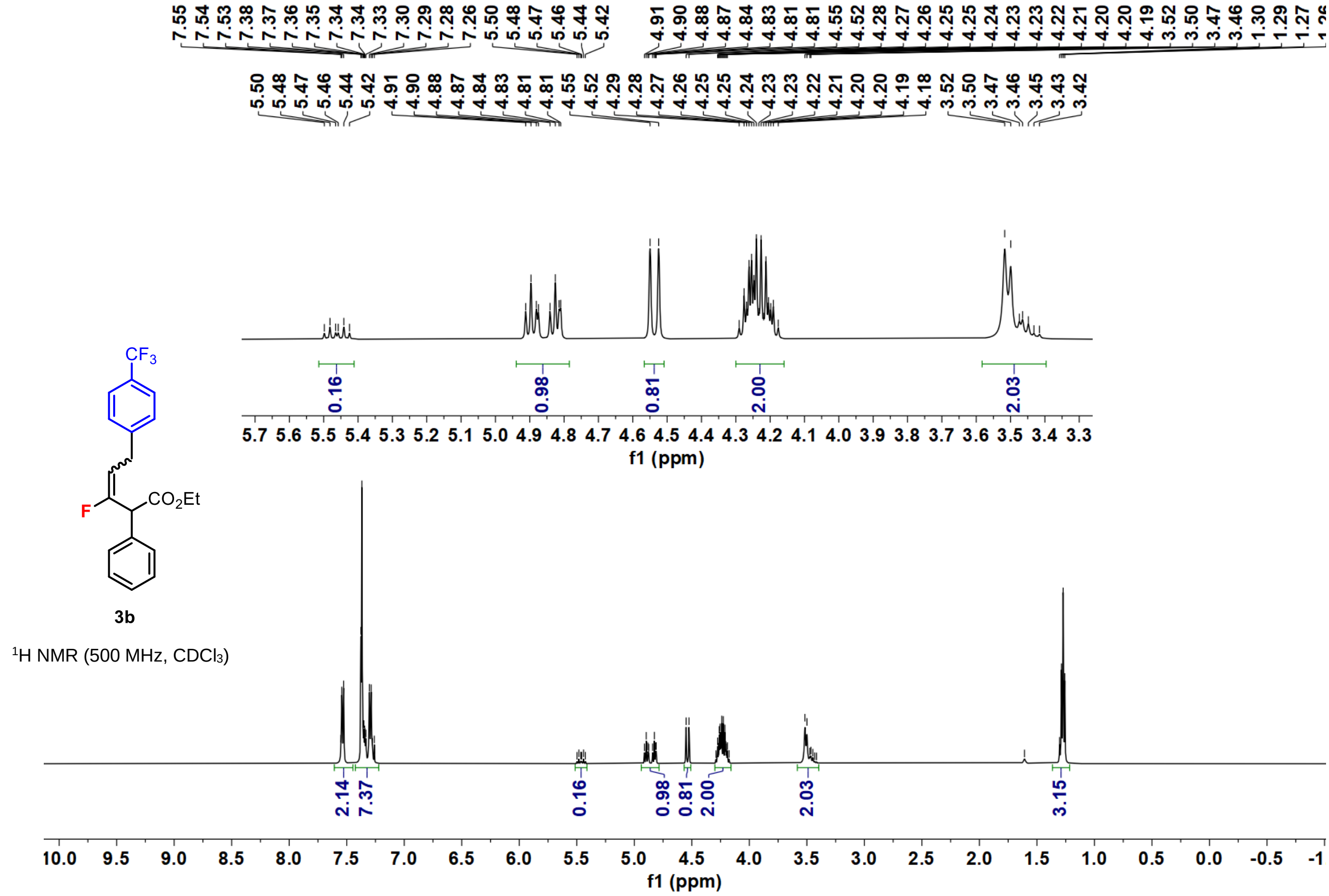

3b

^{1}H NMR (500 MHz, $CDCl_3$)

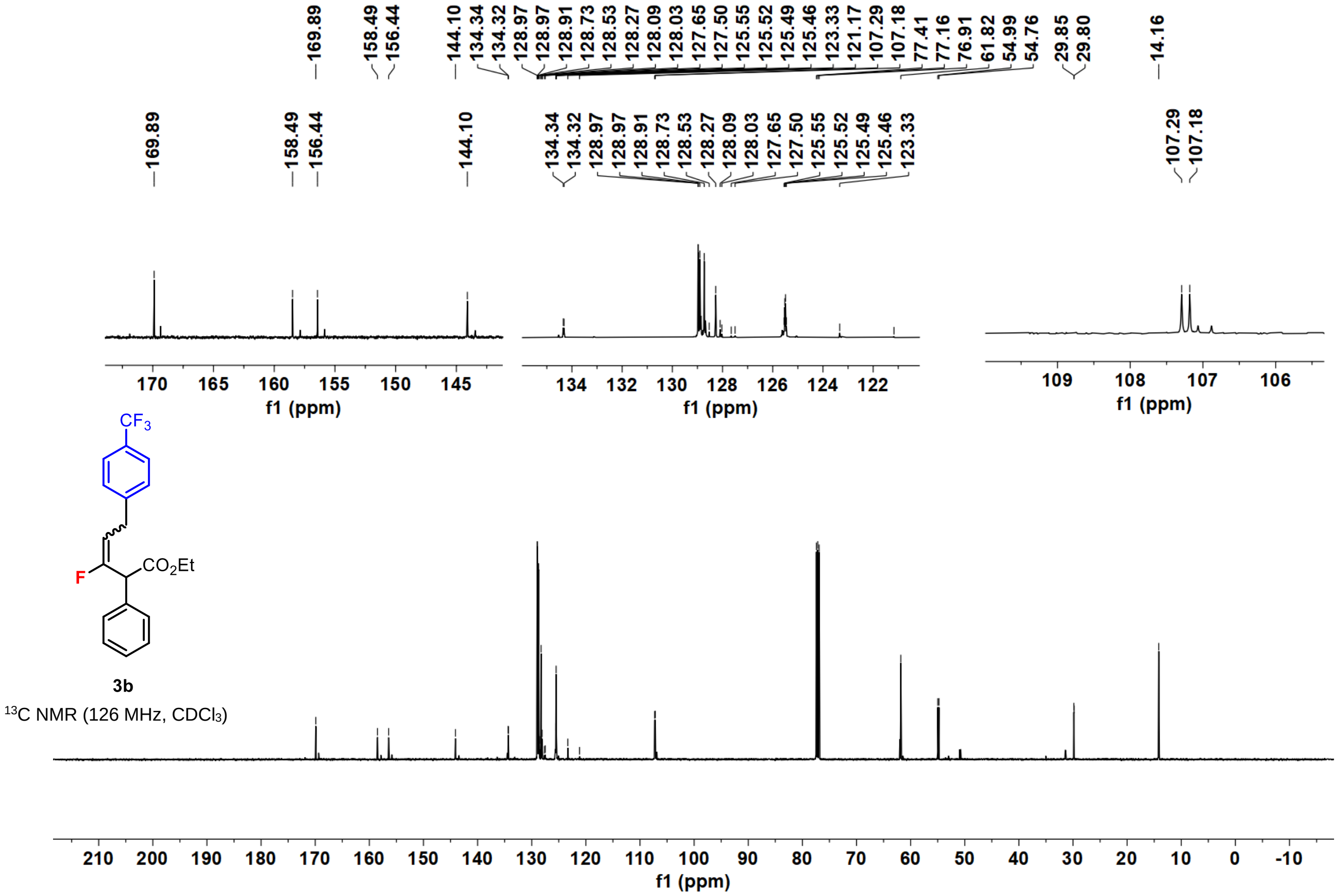

3b

^{13}C NMR (126 MHz, $CDCl_3$)

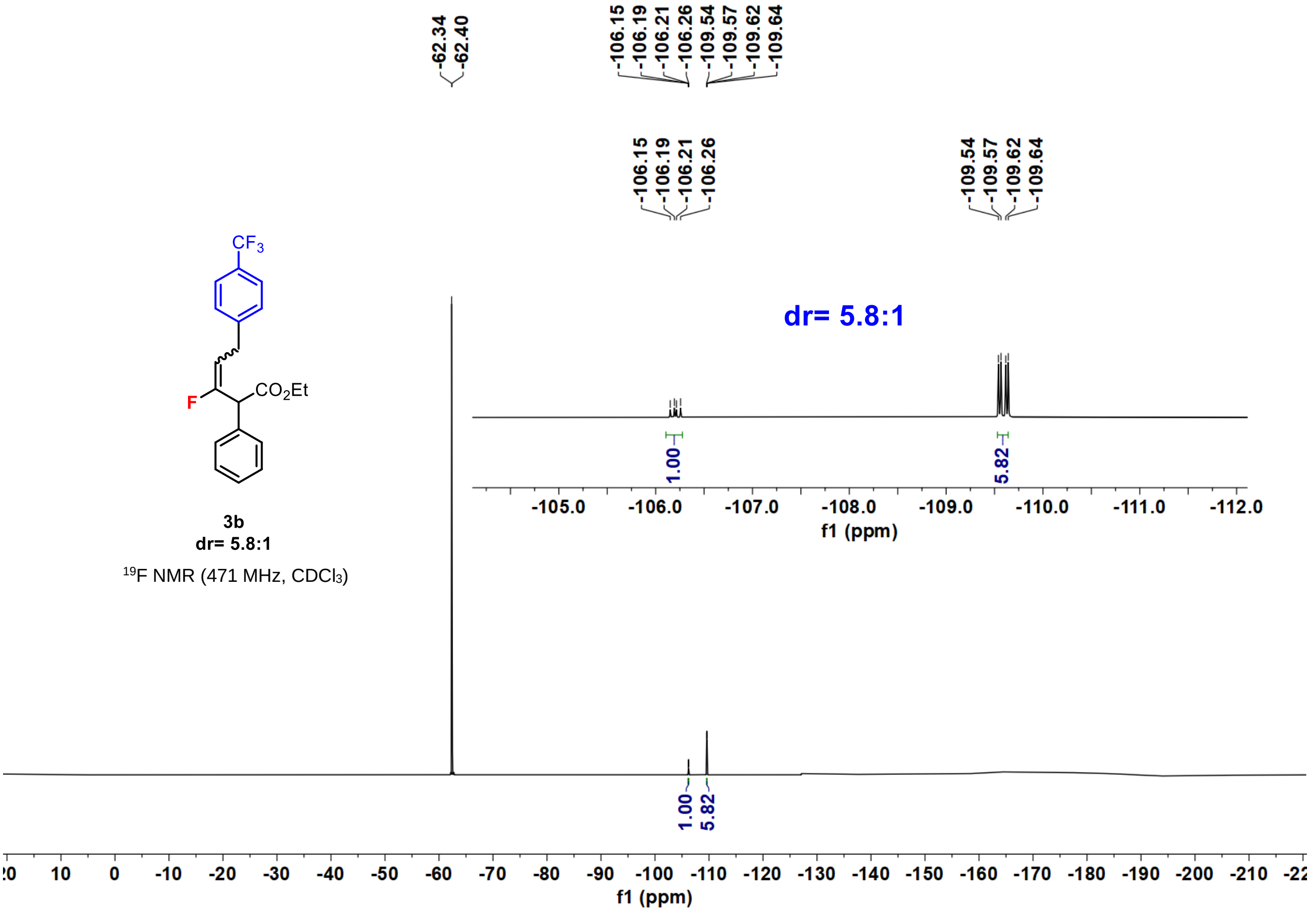

3b
dr= 5.8:1
^{19}F NMR (471 MHz, $CDCl_3$)

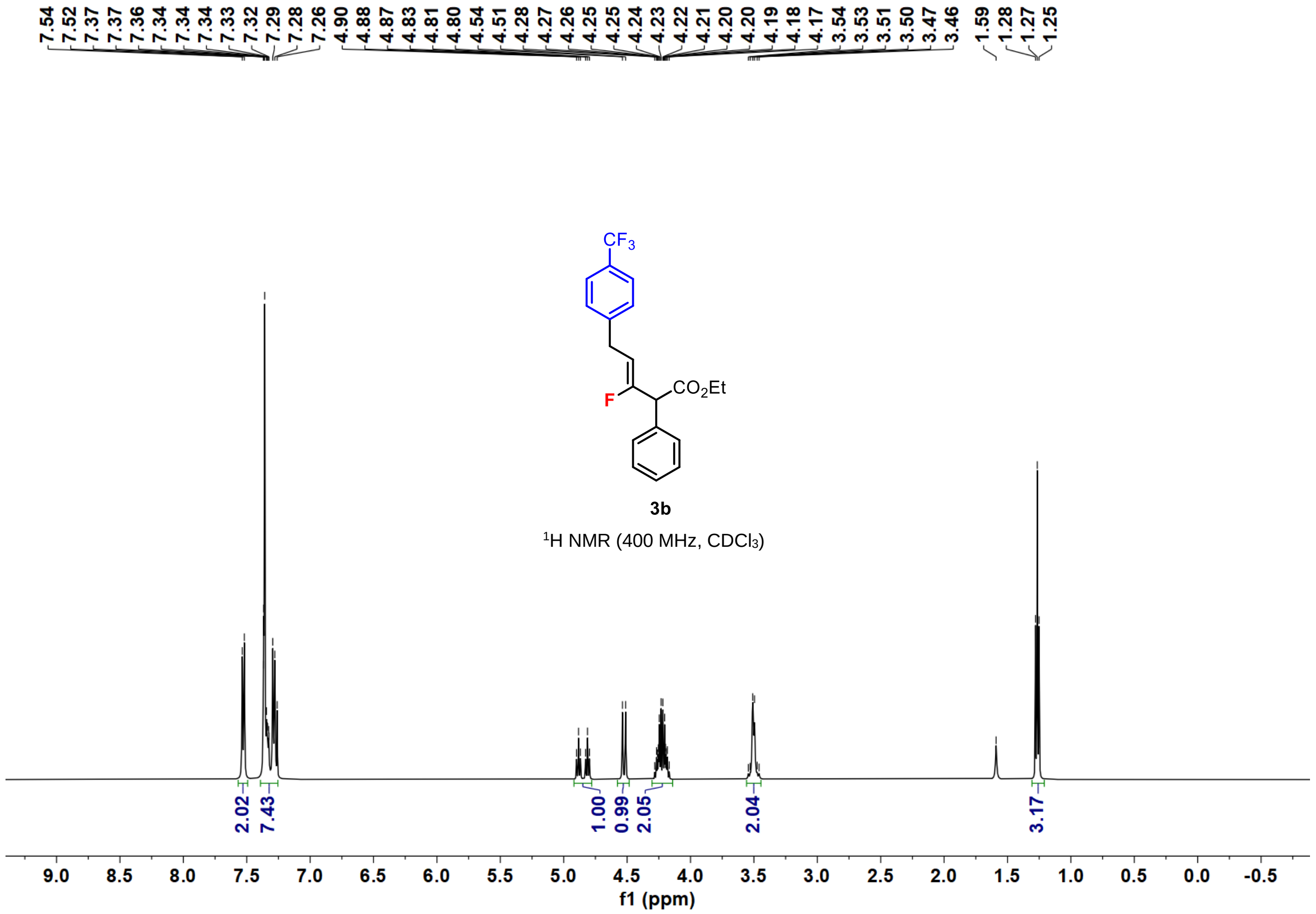
7.54 7.52 7.37 7.37 7.36 7.34 7.34 7.34 7.33 7.32 7.29 7.28 7.26 4.90 4.88 4.87 4.83 4.81 4.80 4.54 4.51 4.28 4.27 4.26 4.25 4.25 4.24 4.23 4.22 4.21 4.20 4.20 4.19 4.18 4.17 3.54 3.53 3.51 3.50 3.47 3.46 1.59 1.28 1.27 1.25
CF3
CO2Et
F
3b
1H NMR (400 MHz, CDCl3)
2.02 7.43 1.00 0.99 2.05 2.04 3.17
f1 (ppm)

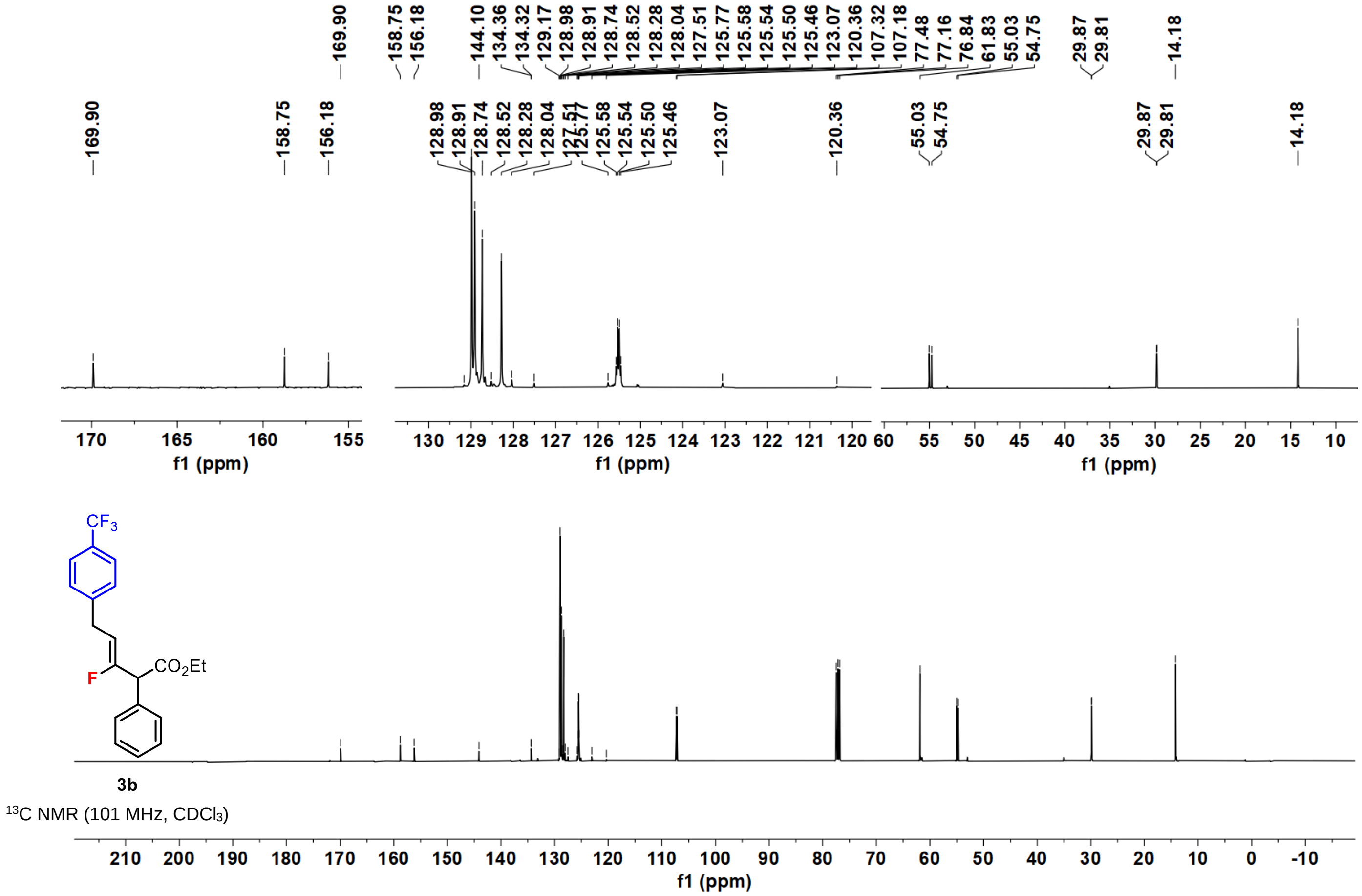
169.90 158.75 156.18 144.10 134.36 134.32 129.17 128.98 128.91 128.74 128.52 128.28 128.04 127.51 125.77 125.58 125.54 125.50 125.46 123.07 120.36 107.32 107.18 77.48 77.16 76.84 61.83 55.03 54.75 29.87 29.81 14.18
f1 (ppm)
CF3
CO2Et
F
3b
13C NMR (101 MHz, CDCl3)
f1 (ppm)

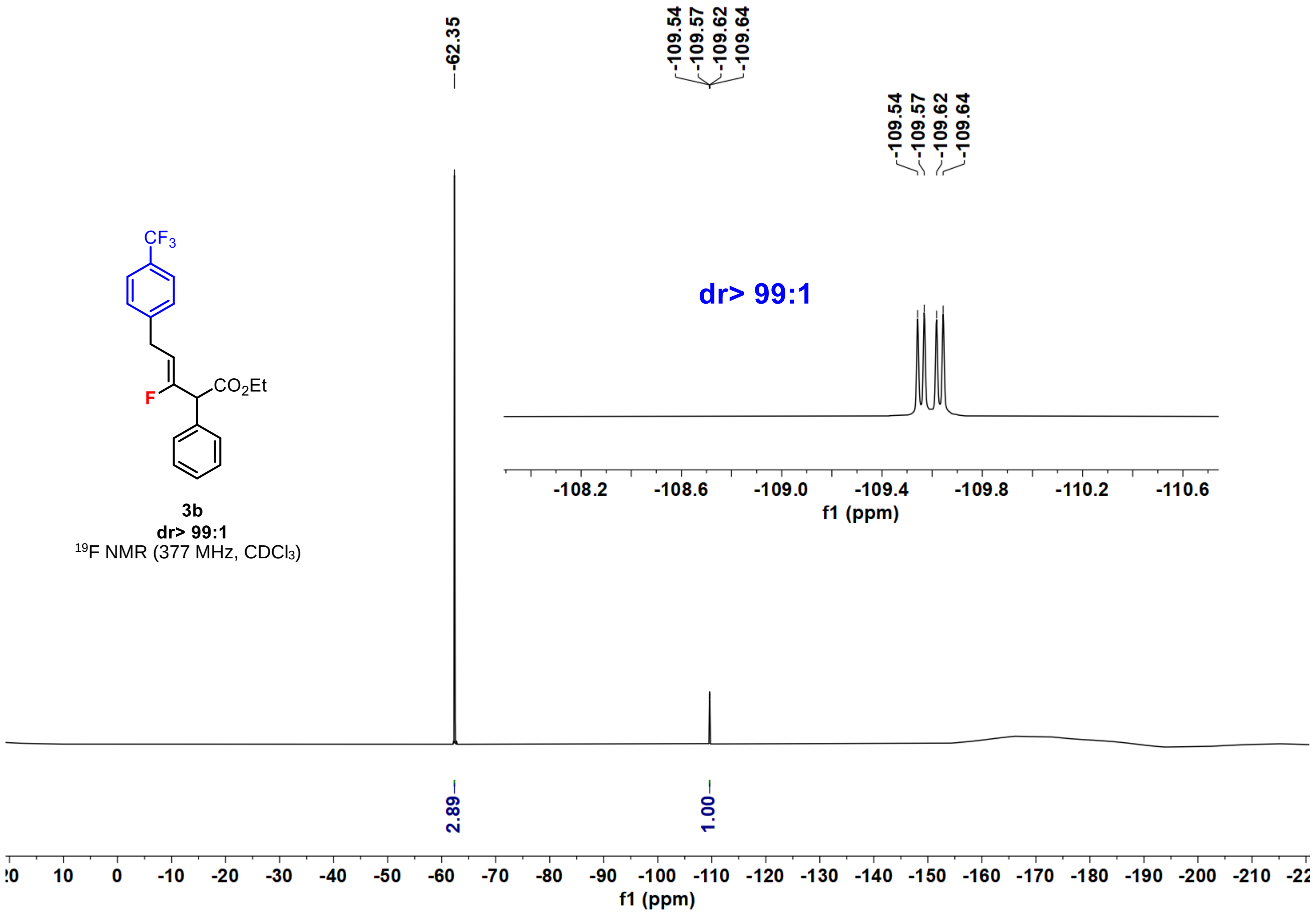

CF3
F
CO2Et
3b
dr> 99:1
19F NMR (377 MHz, CDCl3)
-62.35
-109.54
-109.57
-109.62
-109.64
dr> 99:1
2.89
1.00
f1 (ppm)

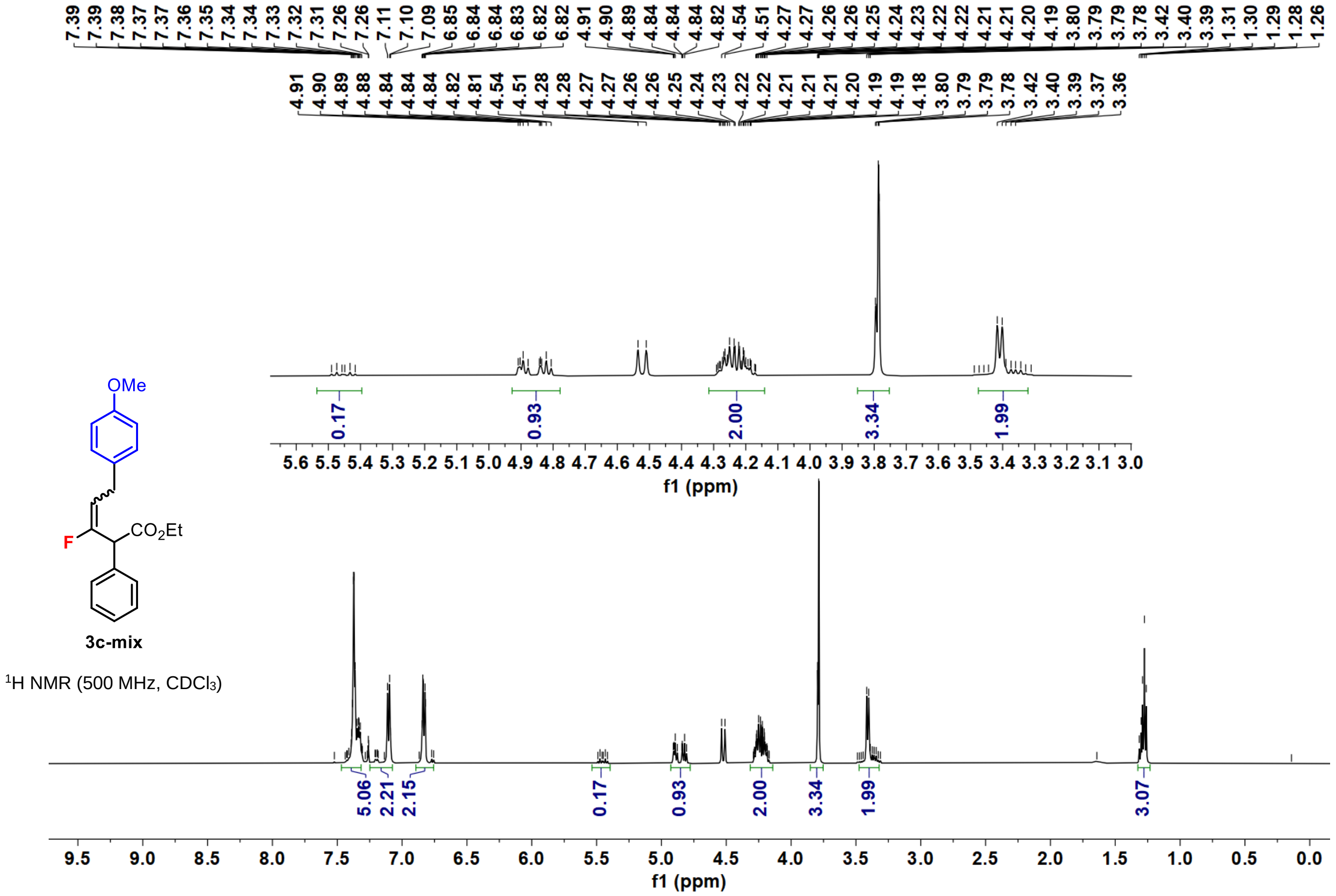

3c-mix

^{1}H NMR (500 MHz, CDCl$_3$)

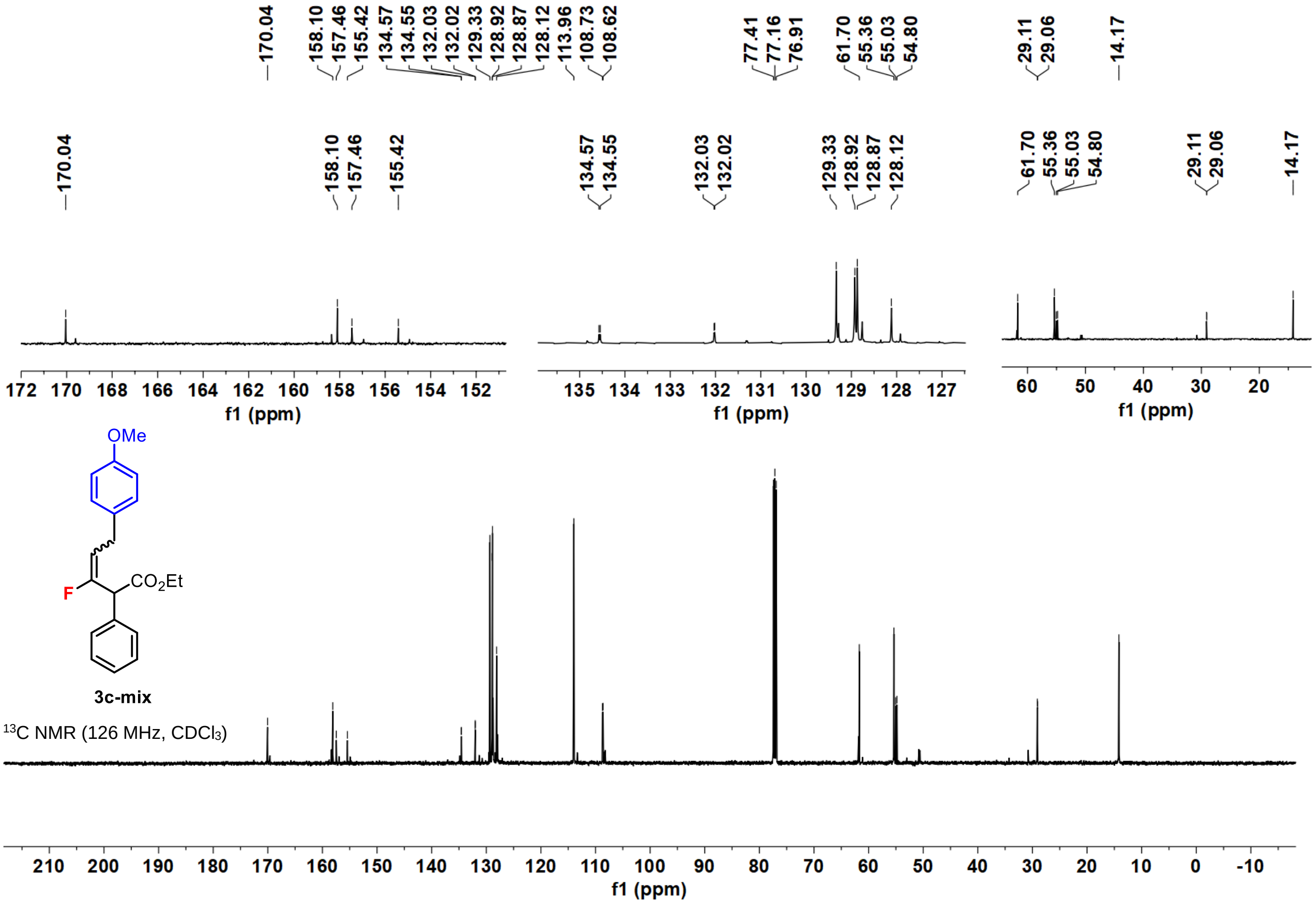

3c-mix

^{13}C NMR (126 MHz, CDCl$_3$)

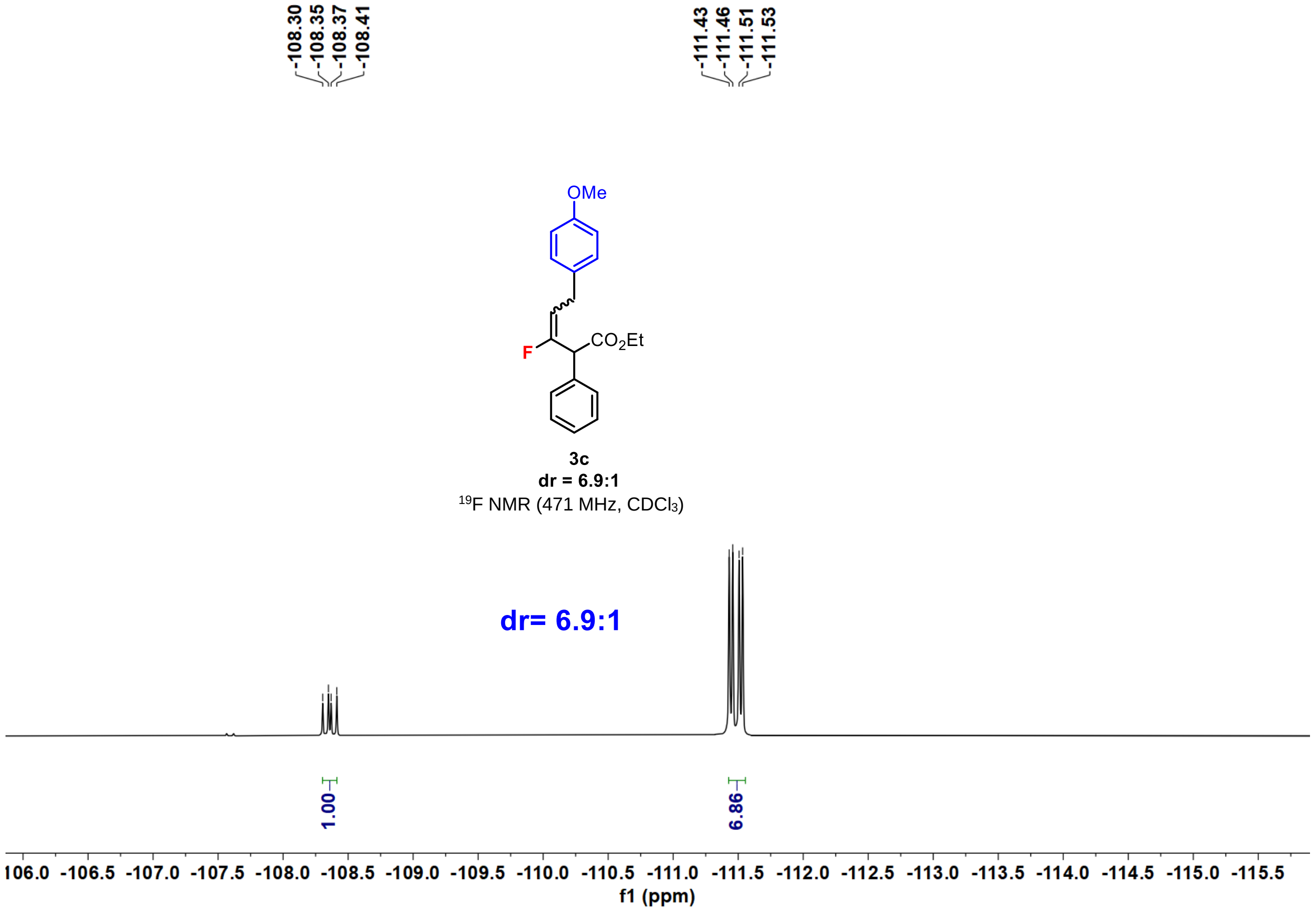
-108.30
-108.35
-108.37
-108.41
-111.43
-111.46
-111.51
-111.53
OMe
F
CO_2Et
3c
dr = 6.9:1
^{19}F NMR (471 MHz, $CDCl_3$)
dr= 6.9:1
1.00
6.86
106.0 -106.5 -107.0 -107.5 -108.0 -108.5 -109.0 -109.5 -110.0 -110.5 -111.0 -111.5 -112.0 -112.5 -113.0 -113.5 -114.0 -114.5 -115.0 -115.5
f1 (ppm)

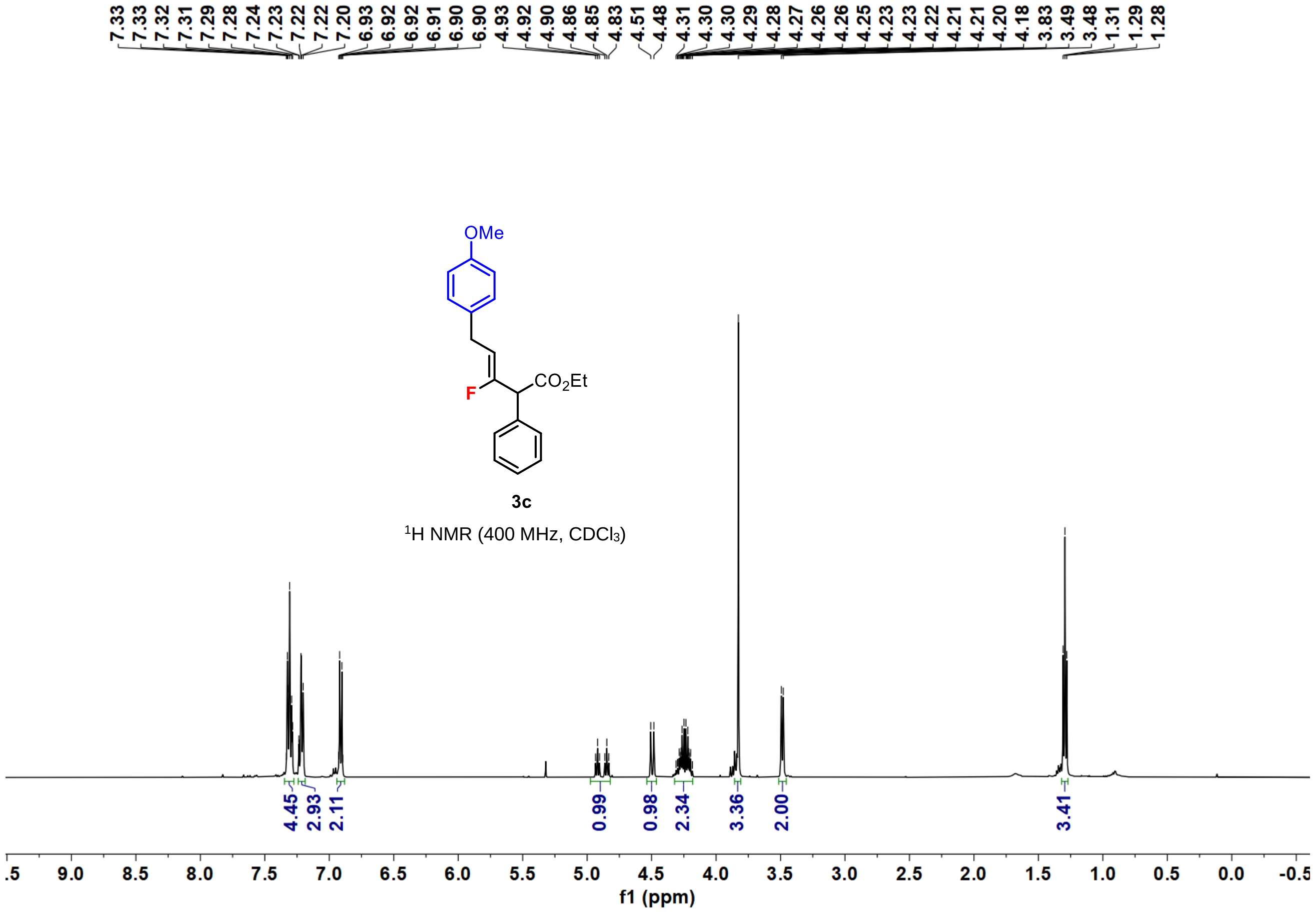

7.33
7.33
7.32
7.31
7.29
7.28
7.24
7.23
7.22
7.22
7.20
6.93
6.92
6.92
6.91
6.90
6.90
4.93
4.92
4.90
4.86
4.85
4.83
4.51
4.48
4.31
4.30
4.29
4.28
4.27
4.26
4.26
4.25
4.23
4.23
4.22
4.21
4.21
4.20
4.18
3.83
3.49
3.48
1.31
1.29
1.28
OMe
CO2Et
F
3c
1H NMR (400 MHz, CDCl3)
4.45
2.93
2.11
0.99
0.98
2.34
3.36
2.00
3.41
f1 (ppm)

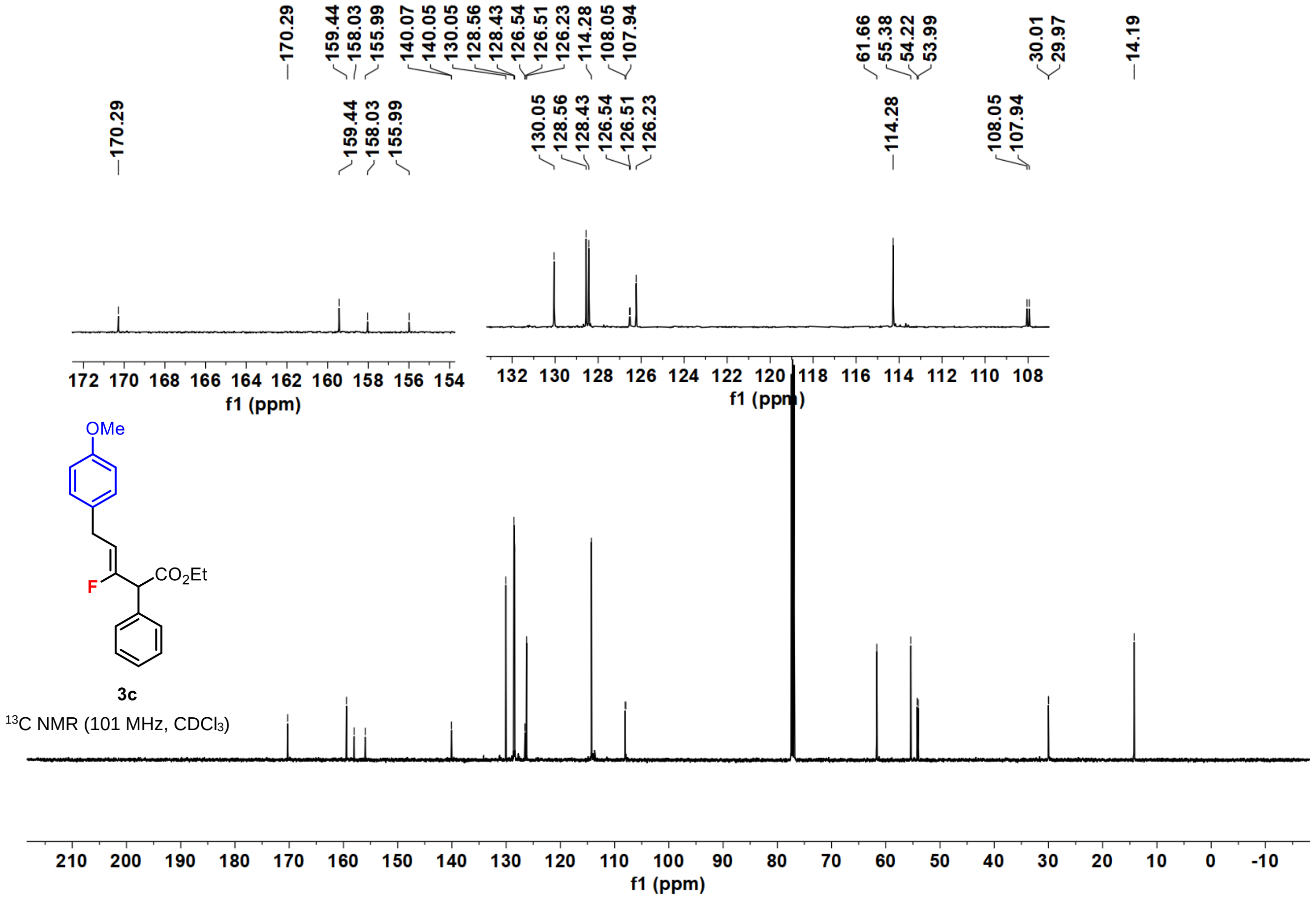

170.29
159.44
158.03
155.99
140.07
140.05
130.05
128.56
128.43
126.54
126.51
126.23
114.28
108.05
107.94
61.66
55.38
54.22
53.99
30.01
29.97
14.19
OMe
CO2Et
F
3c
13C NMR (101 MHz, CDCl3)
f1 (ppm)

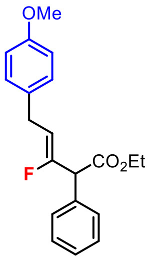

3c
dr= 29:1
^{19}F NMR (377 MHz, $CDCl_3$)

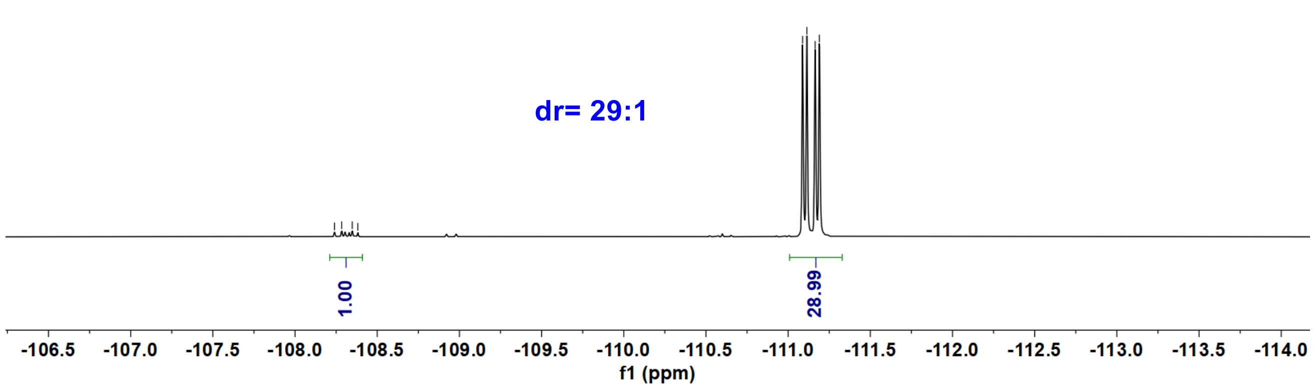

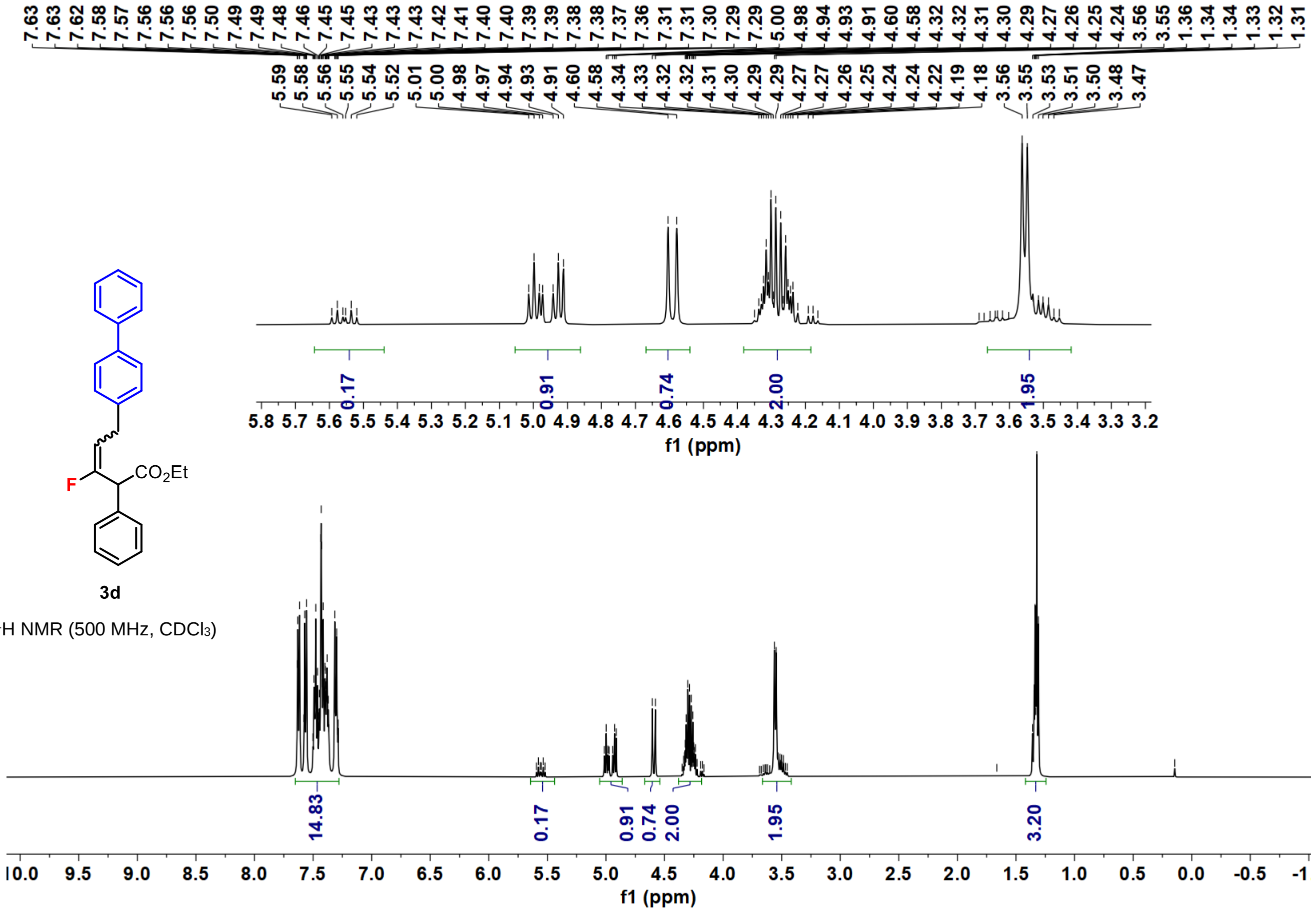

3d

^{1}H NMR (500 MHz, $CDCl_3$)

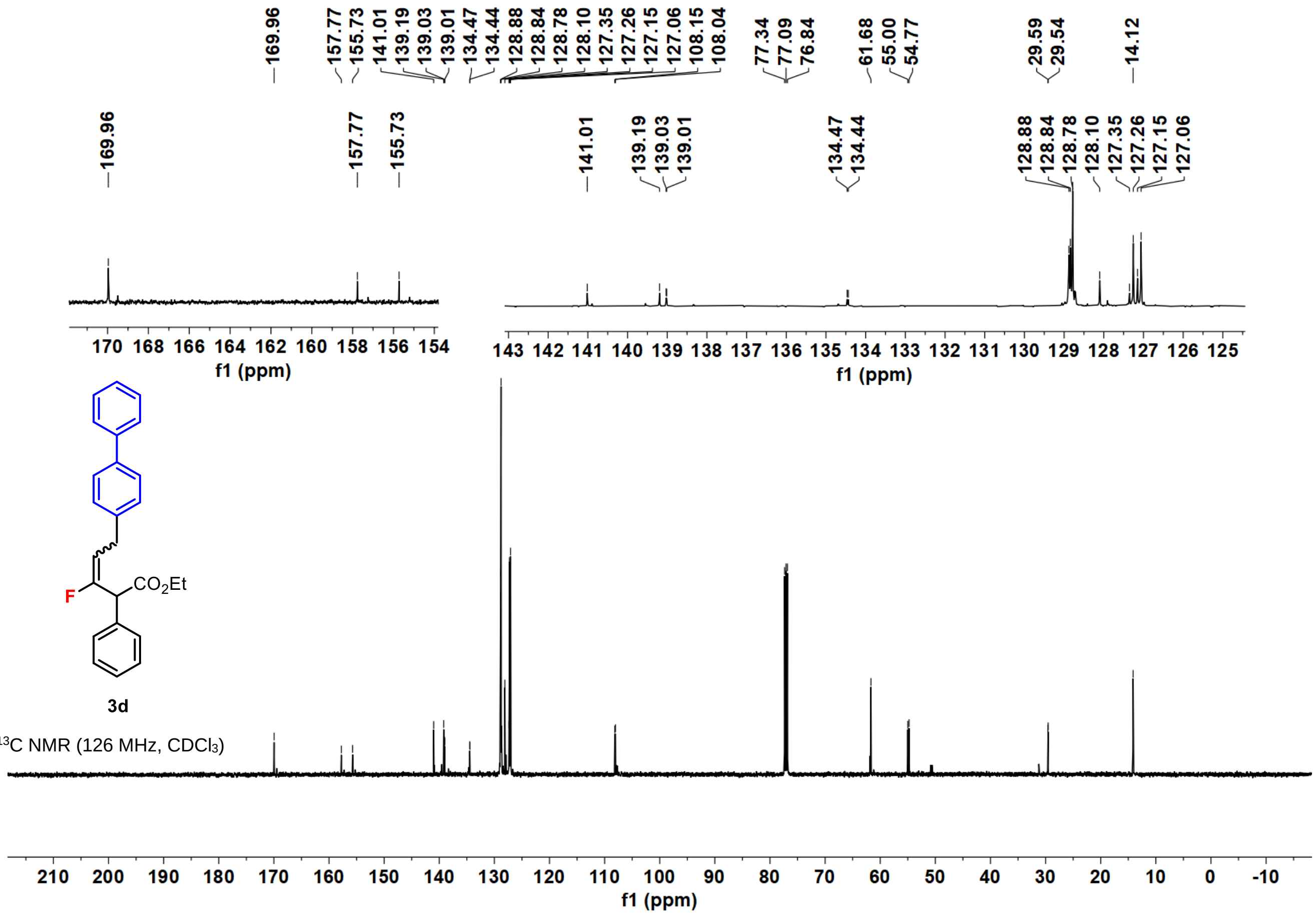

3d

^{13}C NMR (126 MHz, $CDCl_3$)

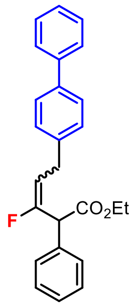

3d
dr= 6.5:1
^{19}F NMR (471MHz, CDCl3)

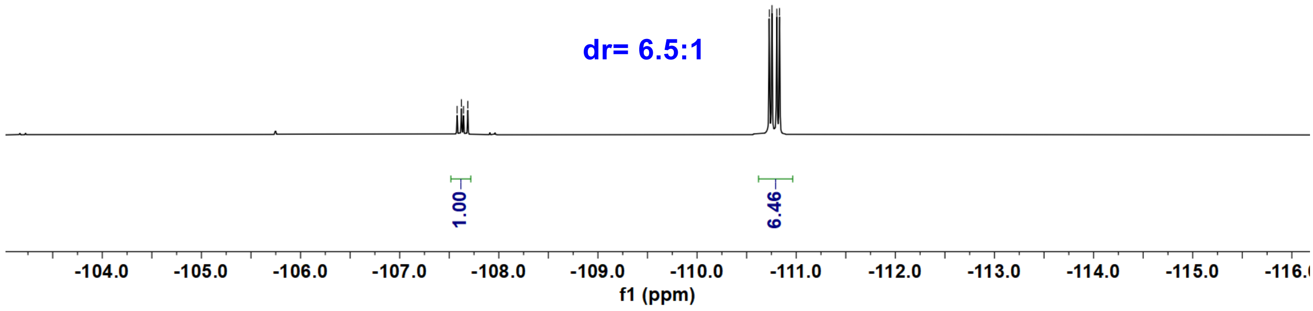

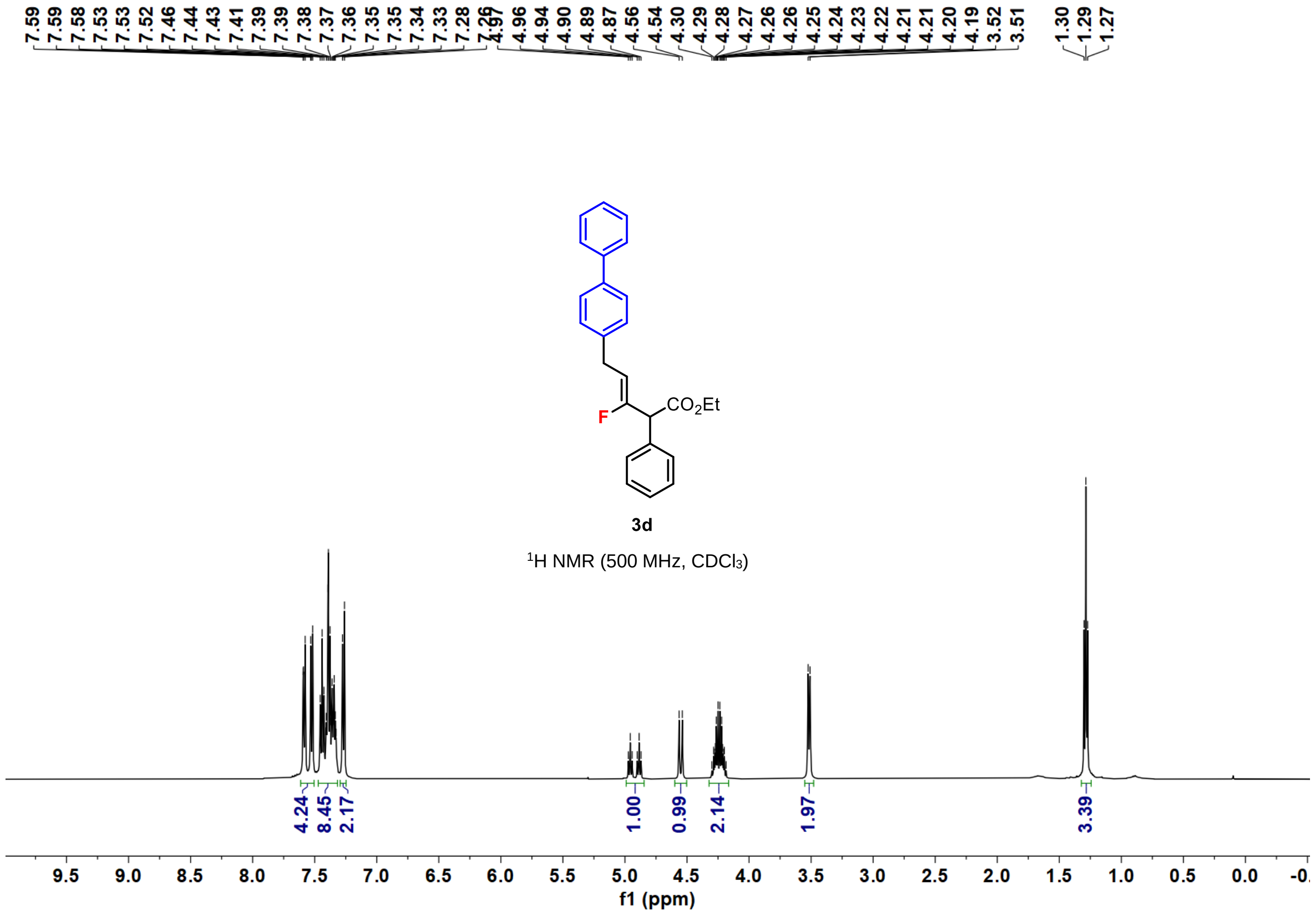

3d

^{1}H NMR (500 MHz, $CDCl_3$)

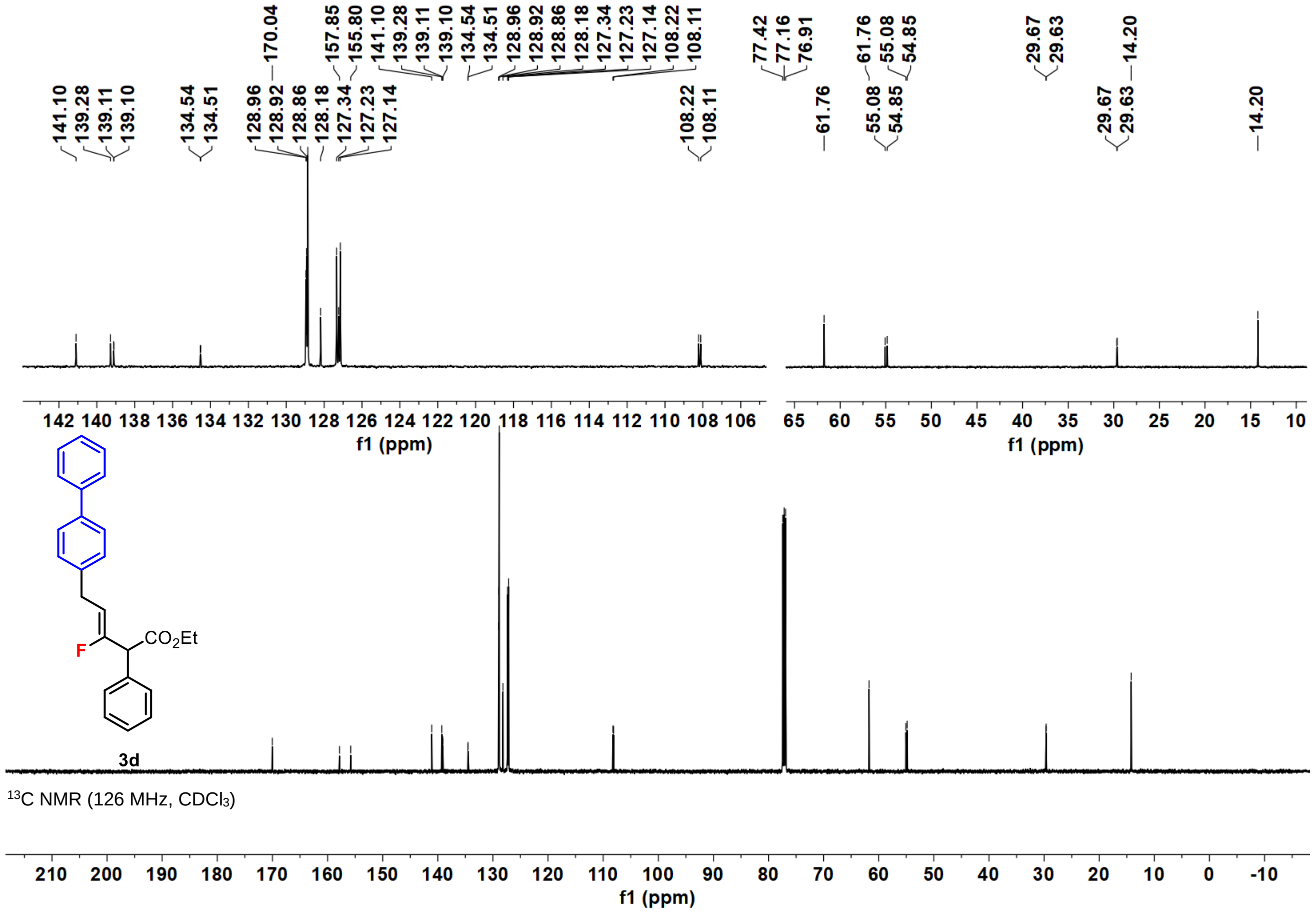

3d

^{13}C NMR (126 MHz, $CDCl_3$)

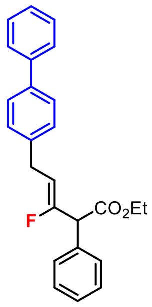

3d
dr> 99:1

^{19}F NMR (471MHz, $CDCl_3$)

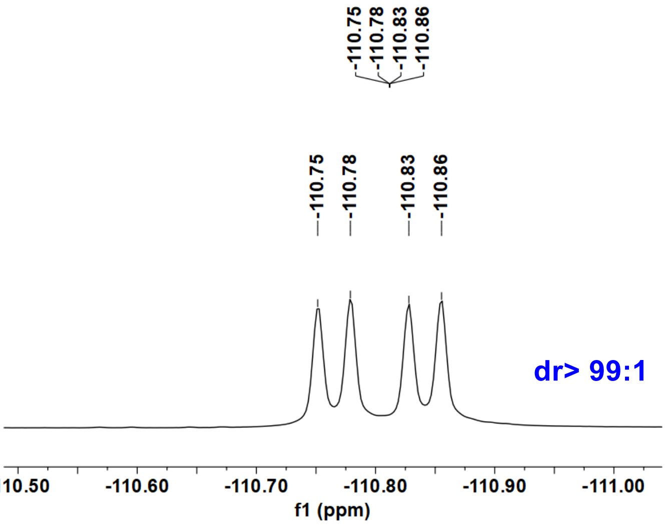

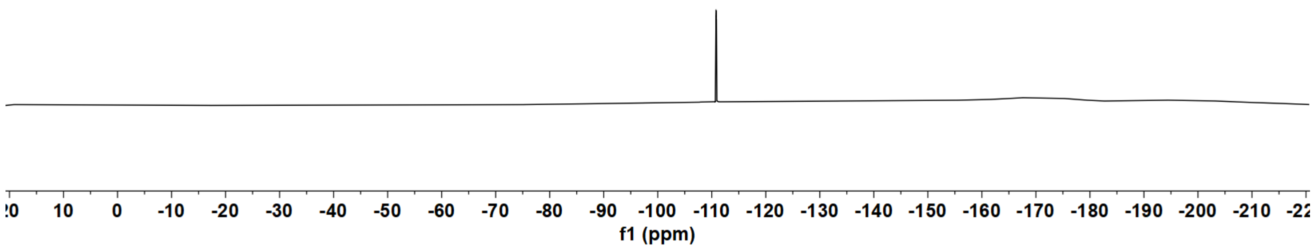

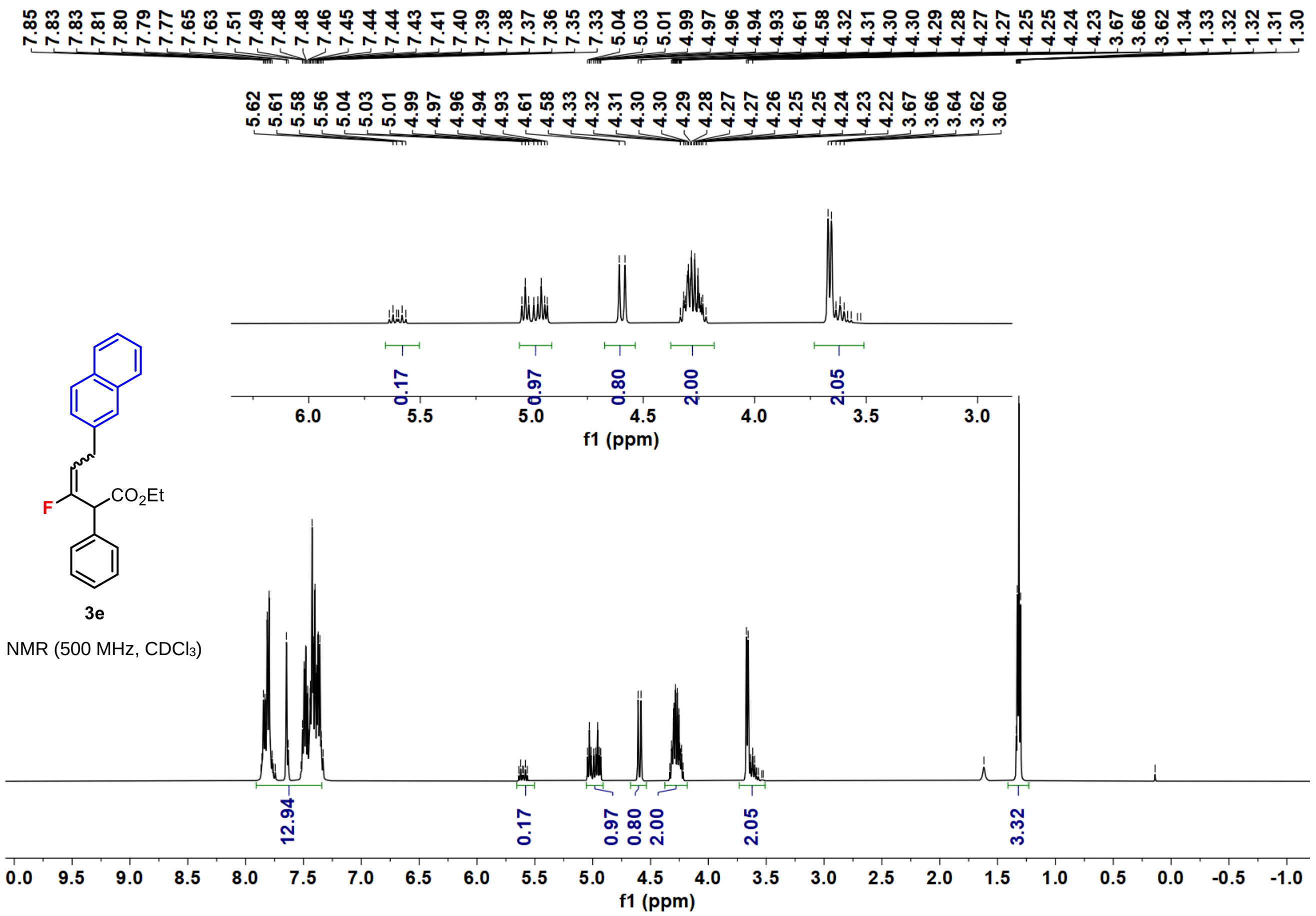

3e

^{1}H NMR (500 MHz, $CDCl_3$)

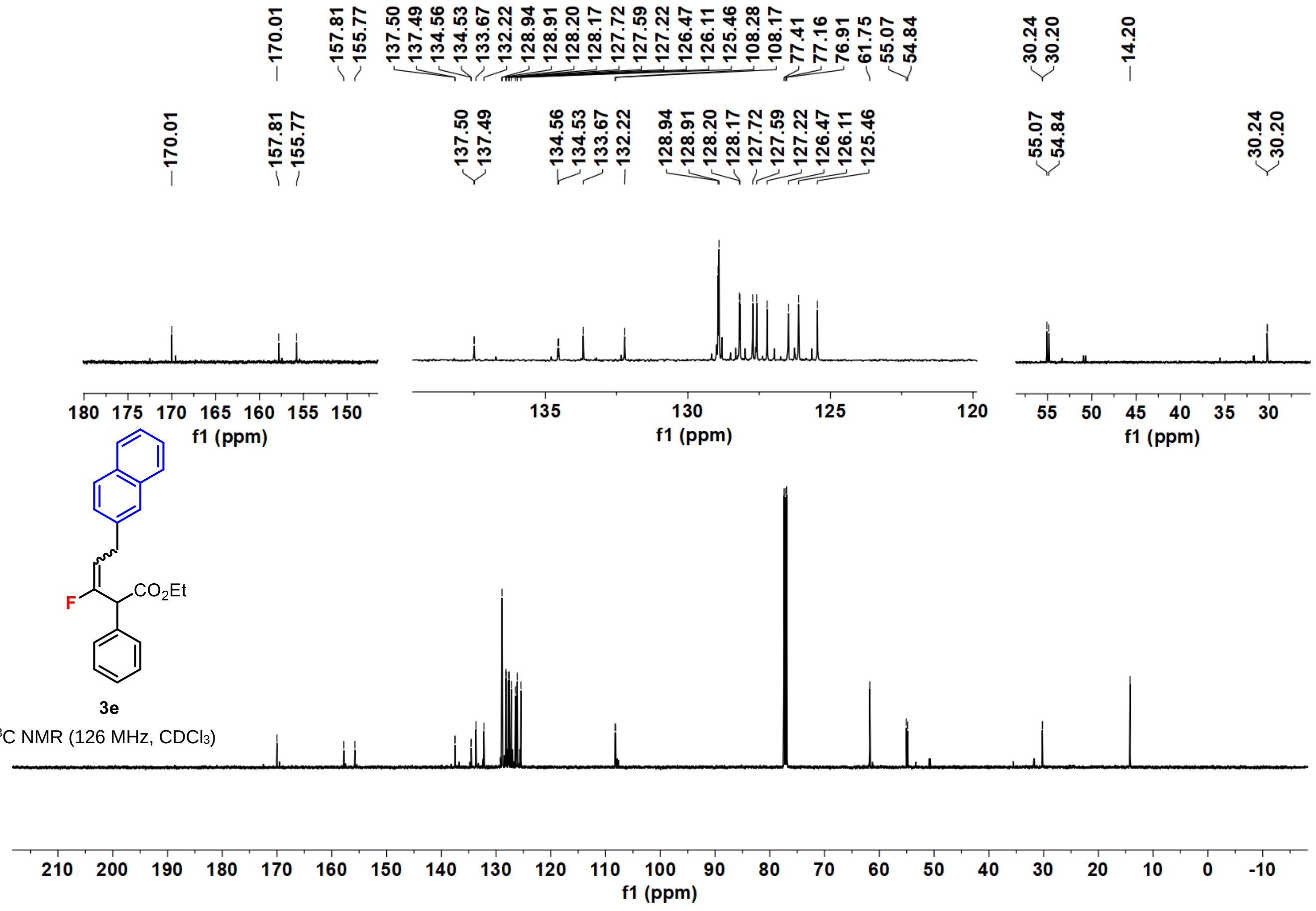

3e

^{13}C NMR (126 MHz, $CDCl_3$)

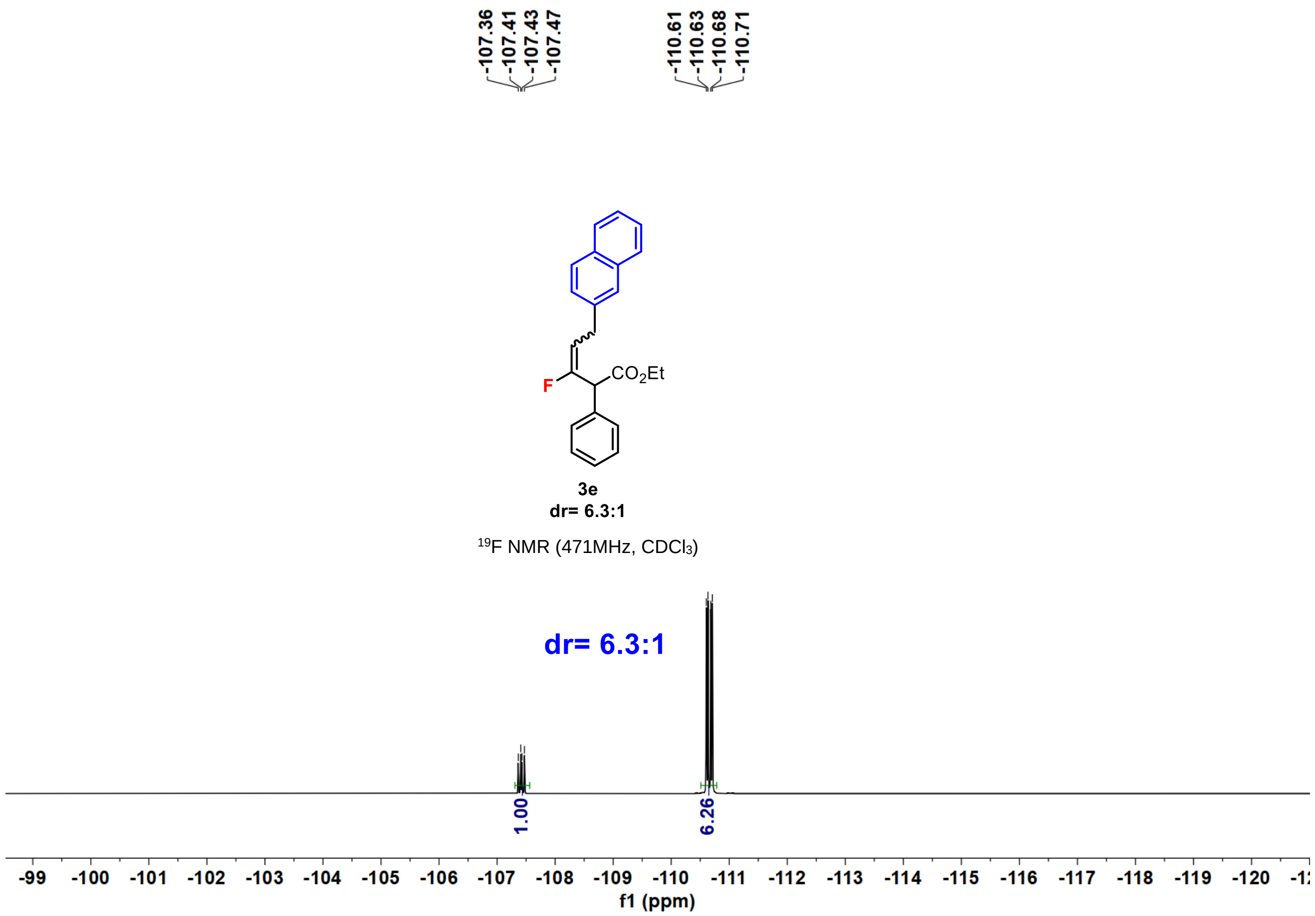

-107.36
-107.41
-107.43
-107.47
-110.61
-110.63
-110.68
-110.71
F
CO2Et
3e
dr= 6.3:1
19F NMR (471MHz, CDCl3)
dr= 6.3:1
1.00
6.26
f1 (ppm)

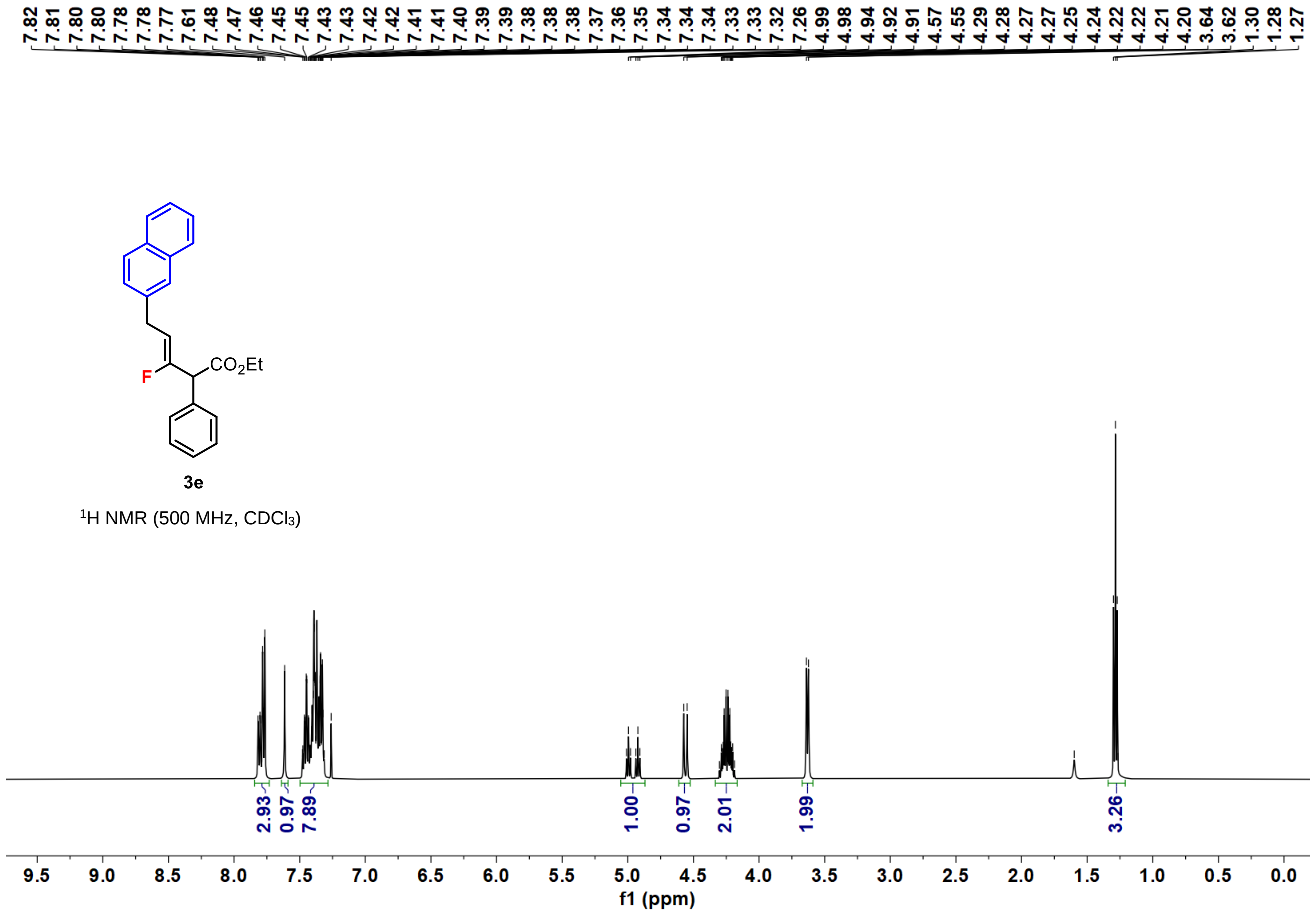
7.82 7.81 7.80 7.78 7.78 7.77 7.61 7.48 7.47 7.46 7.45 7.45 7.43 7.43 7.42 7.42 7.41 7.41 7.40 7.39 7.39 7.38 7.38 7.38 7.37 7.36 7.35 7.34 7.34 7.34 7.33 7.33 7.32 7.26 4.99 4.98 4.94 4.92 4.91 4.57 4.55 4.29 4.28 4.27 4.27 4.25 4.24 4.22 4.22 4.21 4.20 3.64 3.62 1.30 1.28 1.27
CO2Et
F
3e
1H NMR (500 MHz, CDCl3)
2.93 0.97 7.89 1.00 0.97 2.01 1.99 3.26
9.5 9.0 8.5 8.0 7.5 7.0 6.5 6.0 5.5 5.0 4.5 4.0 3.5 3.0 2.5 2.0 1.5 1.0 0.5 0.0
f1 (ppm)

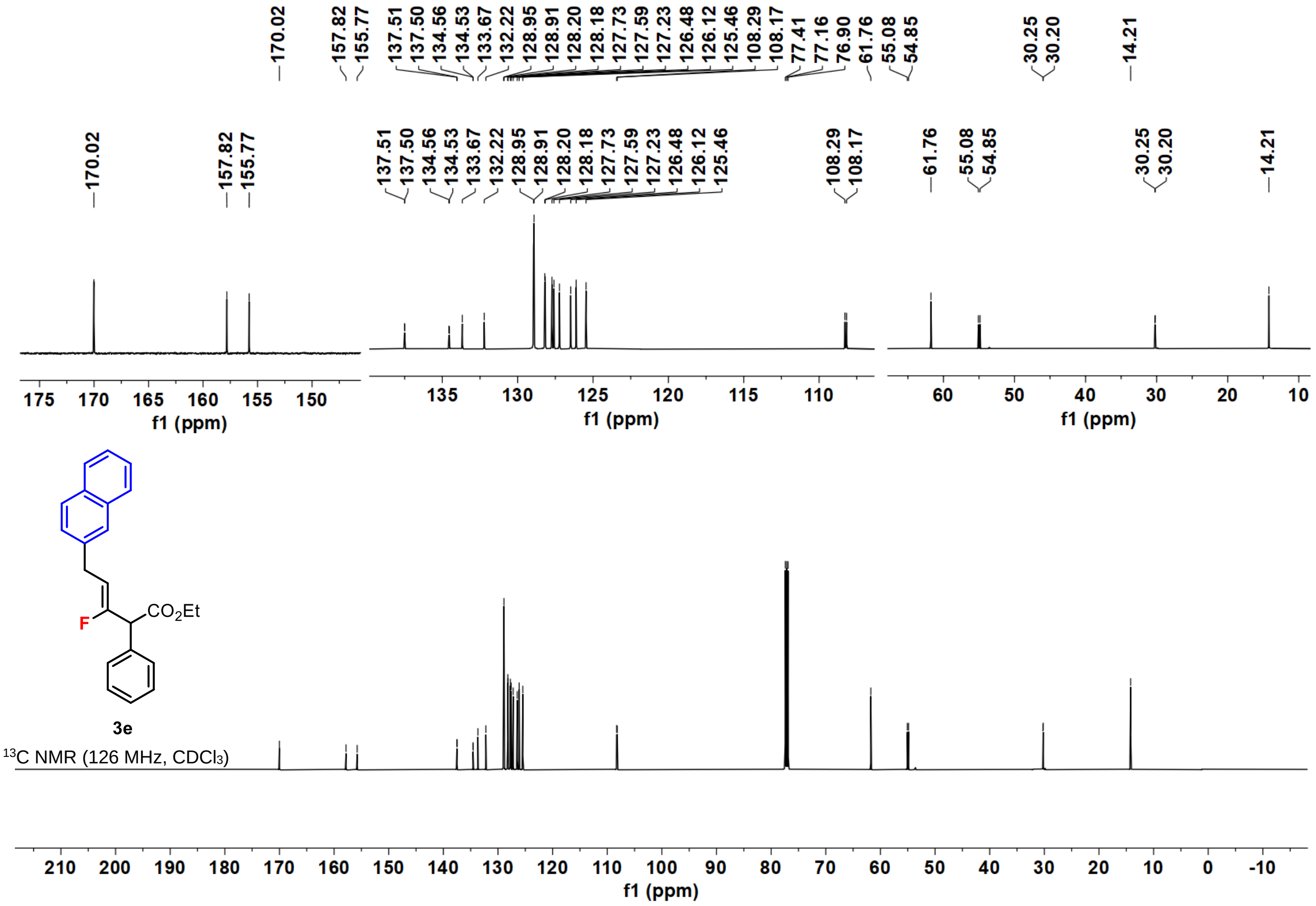
170.02 157.82 155.77 137.51 137.50 134.56 134.53 133.67 132.22 128.95 128.91 128.20 128.18 127.73 127.59 127.23 126.48 126.12 125.46 108.29 108.17 77.41 77.16 76.90 61.76 55.08 54.85 30.25 30.20 14.21
175 170 165 160 155 150
f1 (ppm)
135 130 125 120 115 110
f1 (ppm)
60 50 40 30 20 10
f1 (ppm)
CO2Et
F
3e
13C NMR (126 MHz, CDCl3)
210 200 190 180 170 160 150 140 130 120 110 100 90 80 70 60 50 40 30 20 10 0 -10
f1 (ppm)

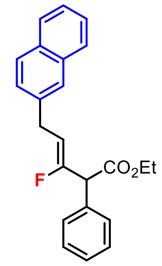

3e
dr> 99:1

^{19}F NMR (471MHz, $CDCl_3$)

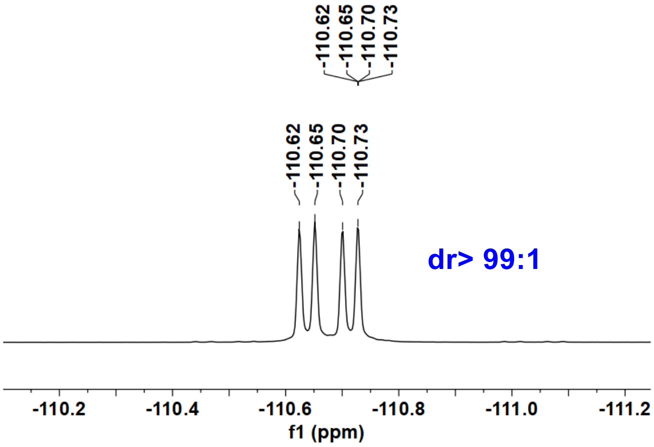

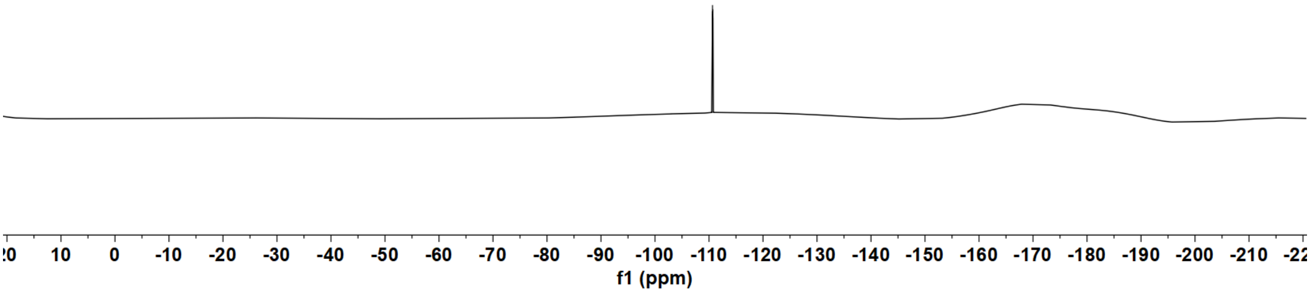

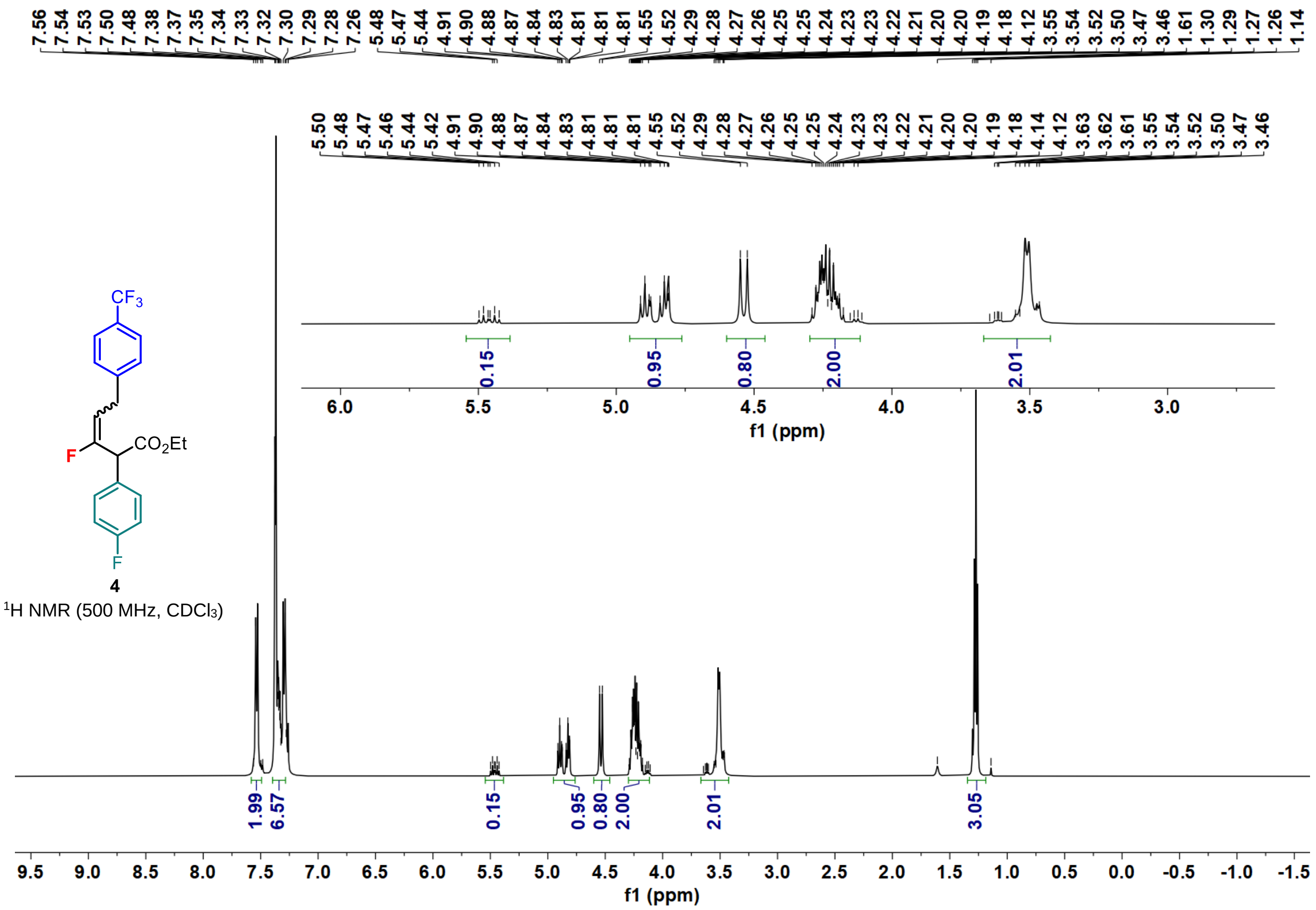

4

^{1}H NMR (500 MHz, $CDCl_3$)

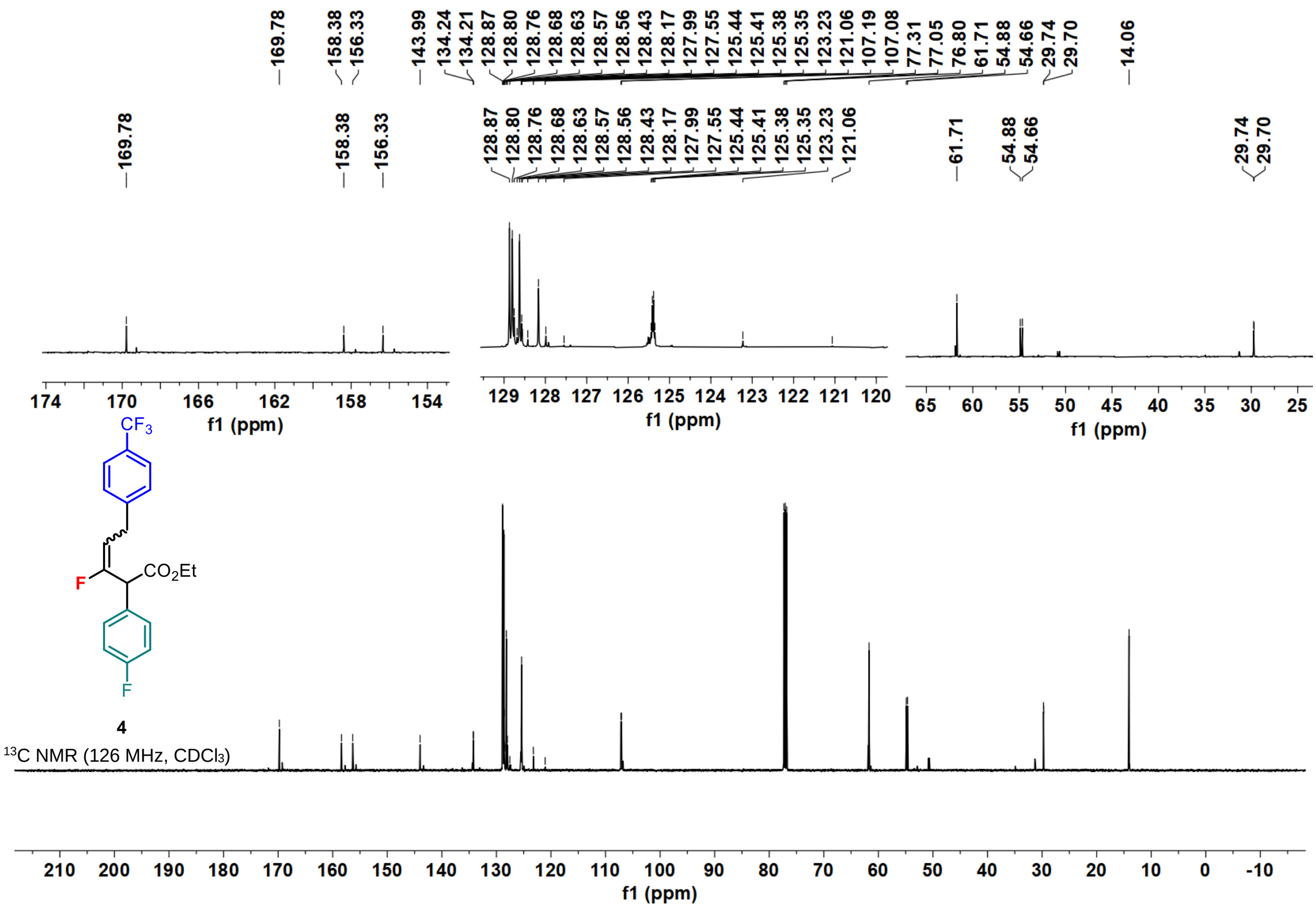

4

^{13}C NMR (126 MHz, $CDCl_3$)

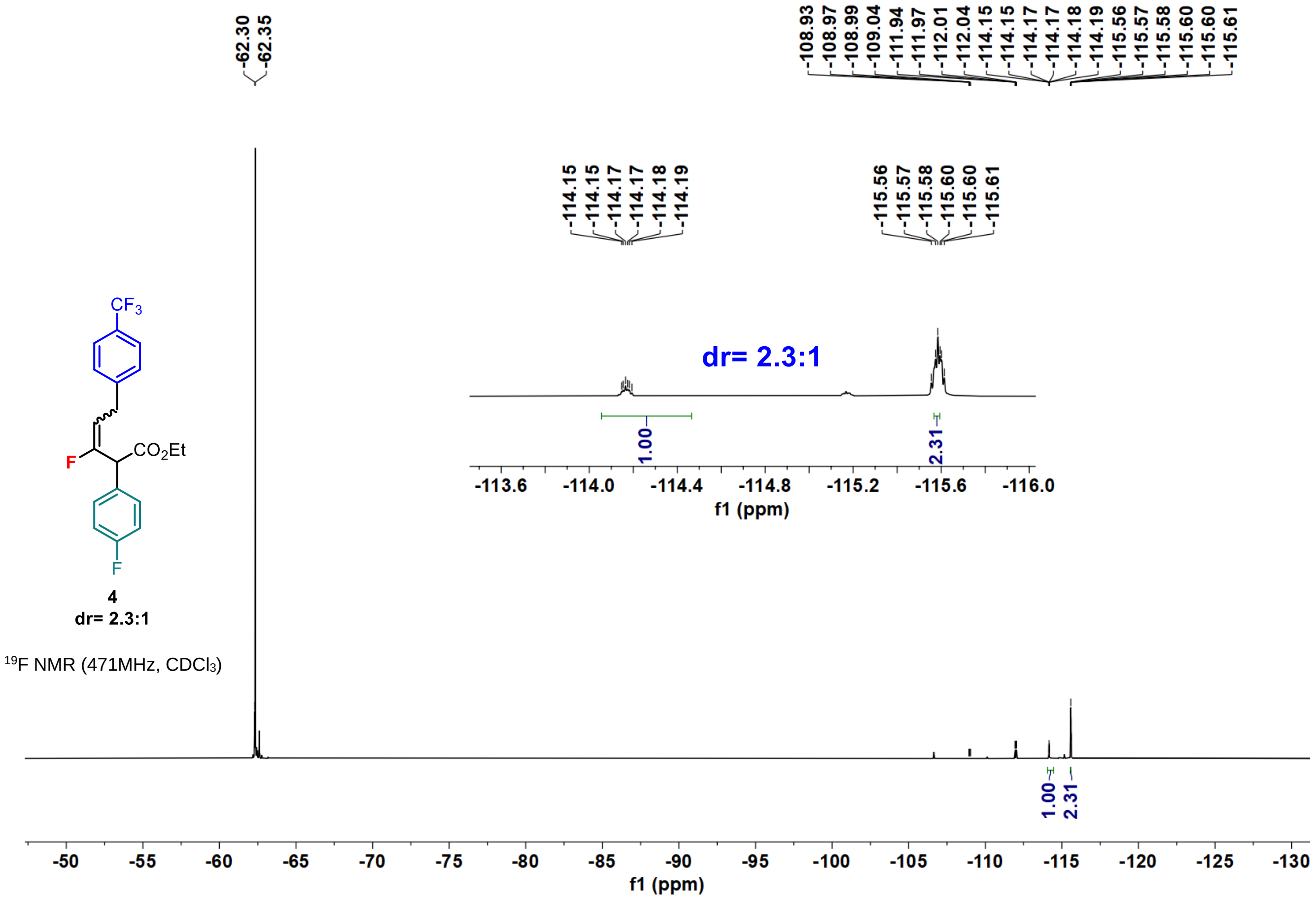
-62.30
-62.35
-108.93
-108.97
-108.99
-109.04
-111.94
-111.97
-112.01
-112.04
-114.15
-114.15
-114.17
-114.17
-114.18
-114.19
-115.56
-115.57
-115.58
-115.60
-115.60
-115.61
dr= 2.3:1
1.00
2.31
f1 (ppm)
CF3
F
CO2Et
F
4
dr= 2.3:1
19F NMR (471MHz, CDCl3)

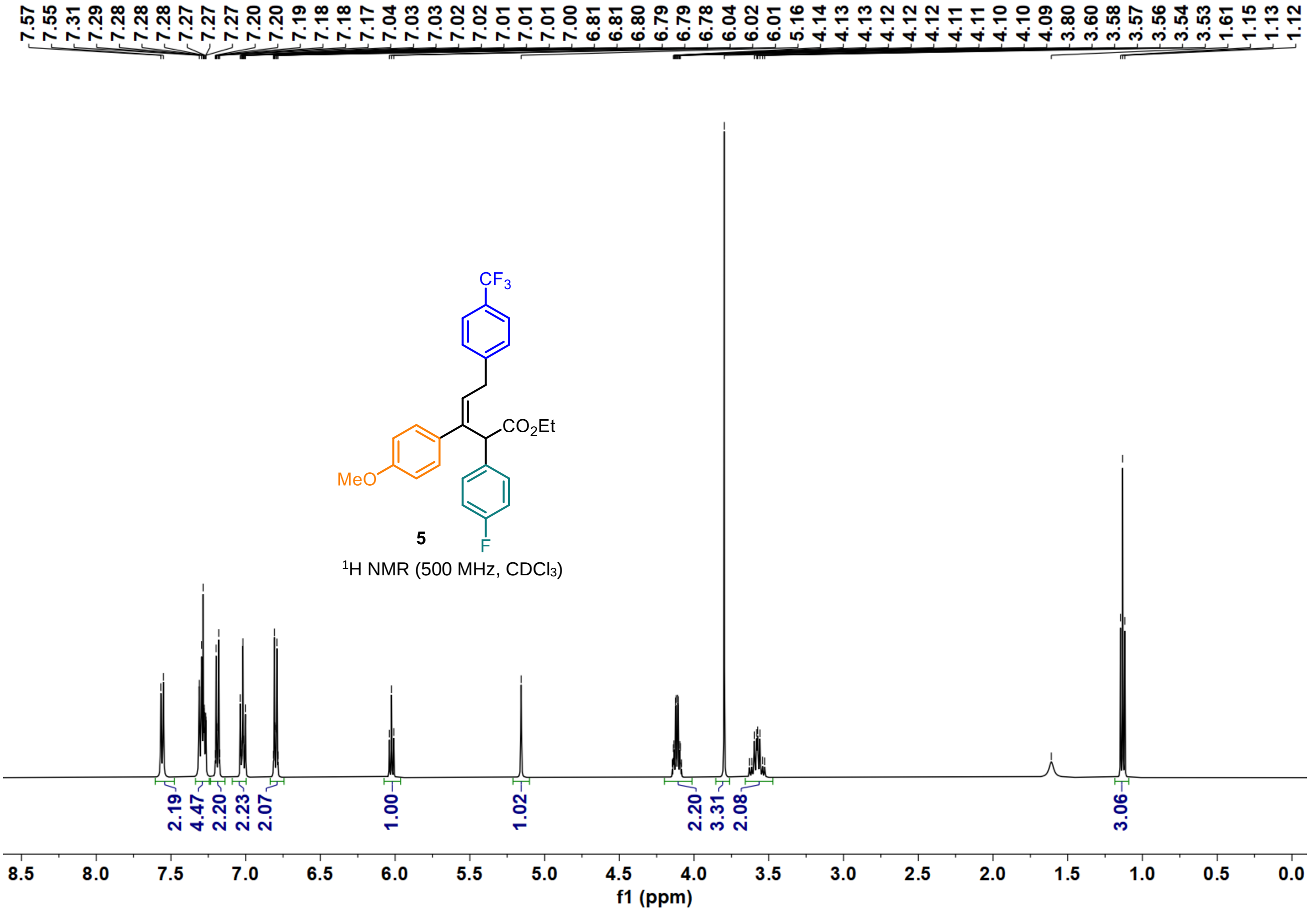

7.57 7.55 7.31 7.29 7.28 7.28 7.28 7.27 7.27 7.27 7.20 7.20 7.19 7.18 7.18 7.17 7.04 7.03 7.03 7.02 7.02 7.01 7.01 7.01 7.00 6.81 6.81 6.80 6.79 6.79 6.78 6.04 6.02 6.01 5.16 4.14 4.13 4.13 4.12 4.12 4.12 4.11 4.11 4.10 4.10 4.09 3.80 3.60 3.58 3.57 3.56 3.54 3.53 1.61 1.15 1.13 1.12
CF3
CO2Et
MeO
F
5
1H NMR (500 MHz, CDCl3)
2.19 4.47 2.20 2.23 2.07 1.00 1.02 2.20 3.31 2.08 3.06
8.5 8.0 7.5 7.0 6.5 6.0 5.5 5.0 4.5 4.0 3.5 3.0 2.5 2.0 1.5 1.0 0.5 0.0
f1 (ppm)

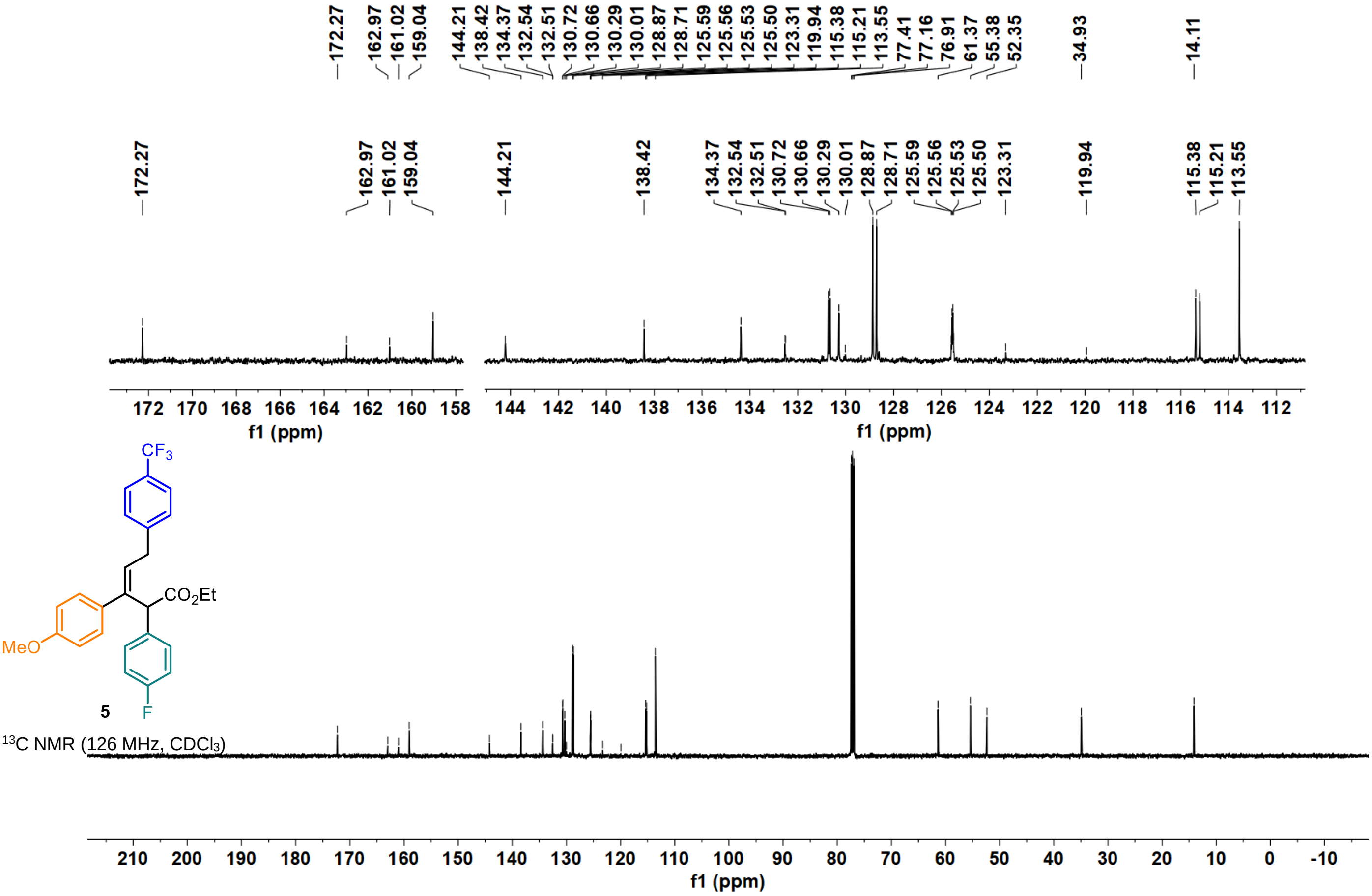

172.27 162.97 161.02 159.04 144.21 138.42 134.37 132.54 132.51 130.72 130.66 130.29 130.01 128.87 128.71 125.59 125.56 125.53 125.50 123.31 119.94 115.38 115.21 113.55 77.41 77.16 76.91 61.37 55.38 52.35 34.93 14.11
172 170 168 166 164 162 160 158
f1 (ppm)
144 142 140 138 136 134 132 130 128 126 124 122 120 118 116 114 112
f1 (ppm)
CF3
CO2Et
MeO
F
5
13C NMR (126 MHz, CDCl3)
210 200 190 180 170 160 150 140 130 120 110 100 90 80 70 60 50 40 30 20 10 0 -10
f1 (ppm)

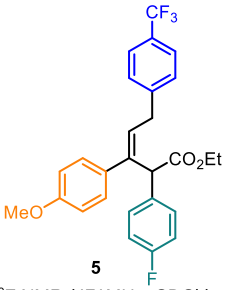

5

^{19}F NMR (471MHz, $CDCl_3$)

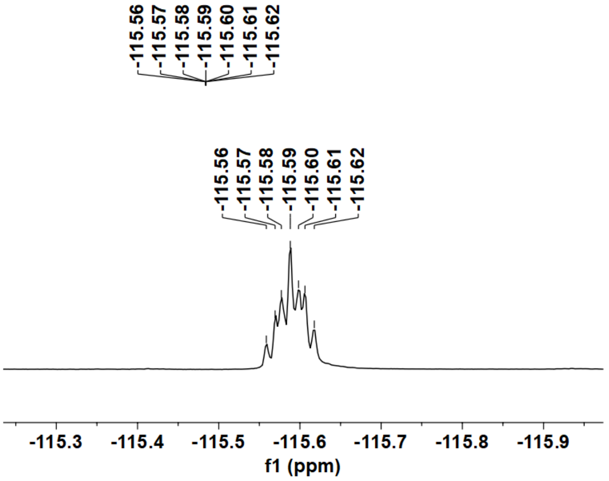

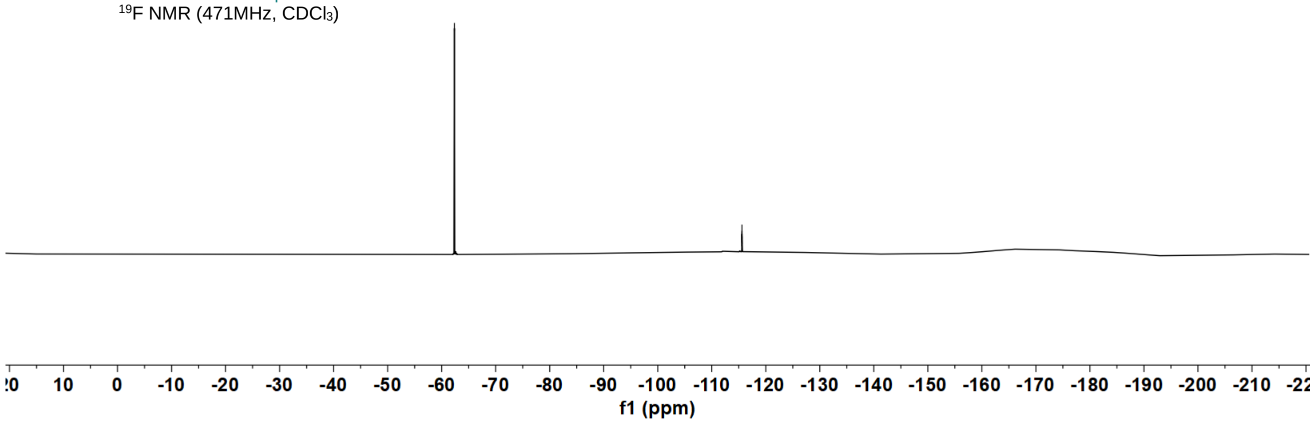

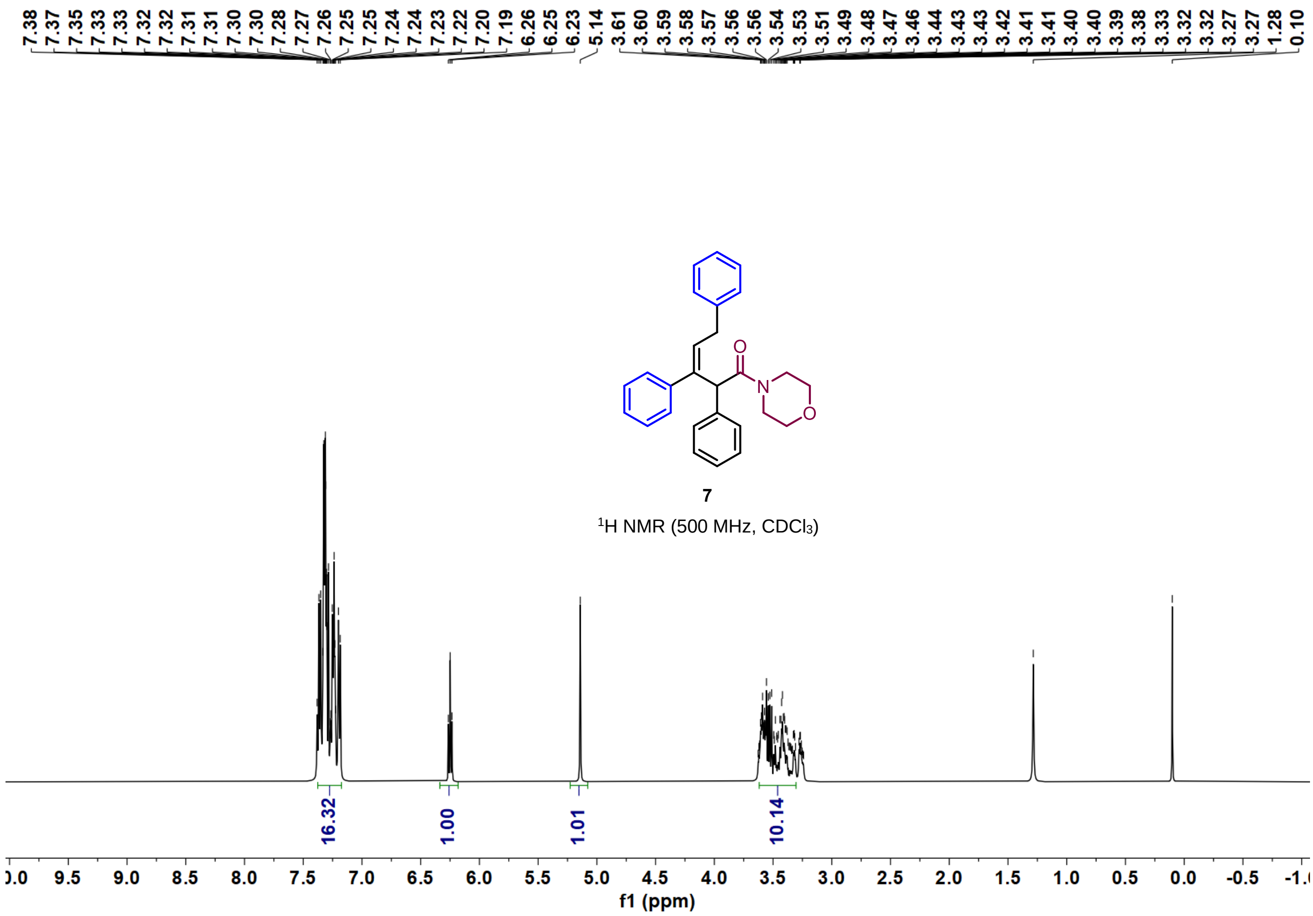
7.38 7.37 7.35 7.33 7.33 7.32 7.32 7.31 7.31 7.30 7.30 7.28 7.27 7.26 7.25 7.25 7.24 7.24 7.23 7.22 7.20 7.19 6.26 6.25 6.23 5.14 3.61 3.60 3.59 3.58 3.57 3.56 3.56 3.54 3.53 3.51 3.49 3.48 3.47 3.46 3.44 3.43 3.42 3.41 3.41 3.40 3.40 3.39 3.38 3.33 3.32 3.32 3.27 3.27 1.28 0.10
O
N
O
7
1H NMR (500 MHz, CDCl3)
16.32
1.00
1.01
10.14
f1 (ppm)

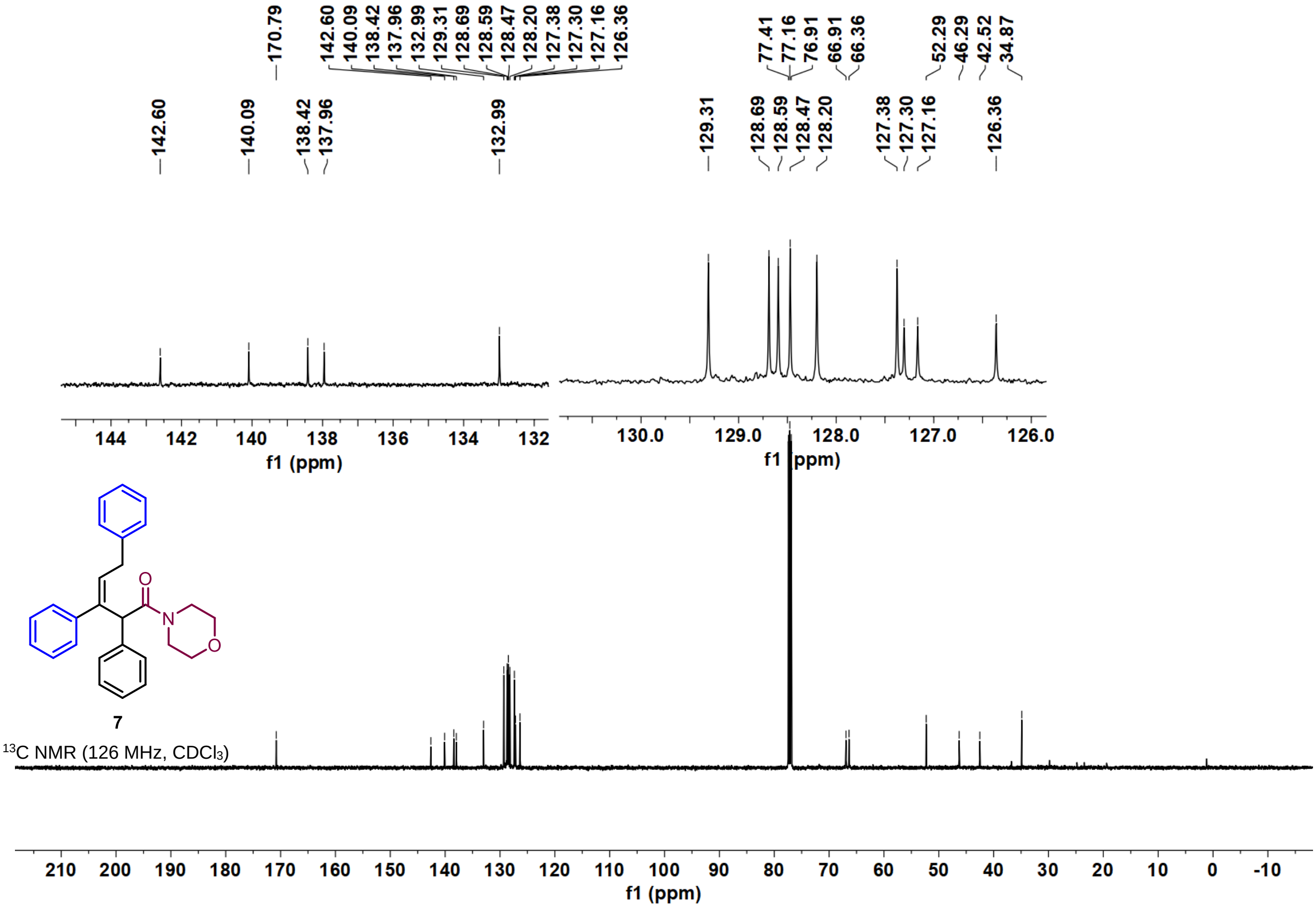
170.79 142.60 140.09 138.42 137.96 132.99 129.31 128.69 128.59 128.47 128.20 127.38 127.30 127.16 126.36 77.41 77.16 76.91 66.91 66.36 52.29 46.29 42.52 34.87
142.60 140.09 138.42 137.96 132.99
129.31 128.69 128.59 128.47 128.20 127.38 127.30 127.16 126.36
f1 (ppm)
f1 (ppm)
O
N
O
7
13C NMR (126 MHz, CDCl3)
f1 (ppm)

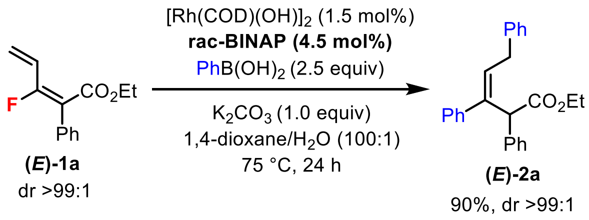

<Chromatogram>

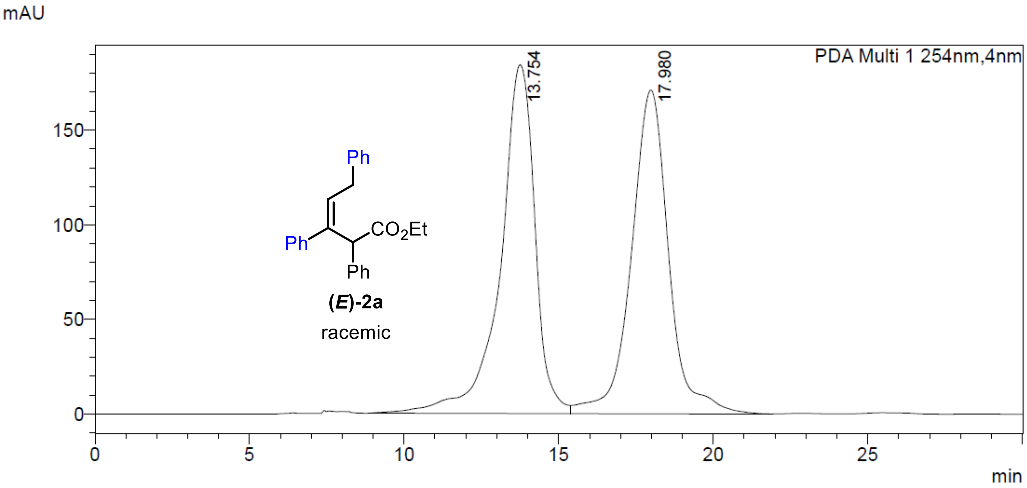

<Peak Table>

PDA Ch1 254nm

Peak#	Ret. Time	Area	Height	Conc.	Unit	Mark	Name
1	13.754	14377765	184234	0.000			
2	17.980	14347686	171070	0.000		V	
Total		28725451	355304				

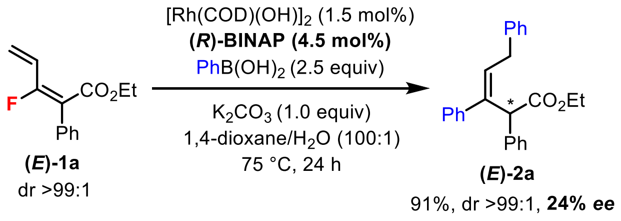

<Chromatogram>

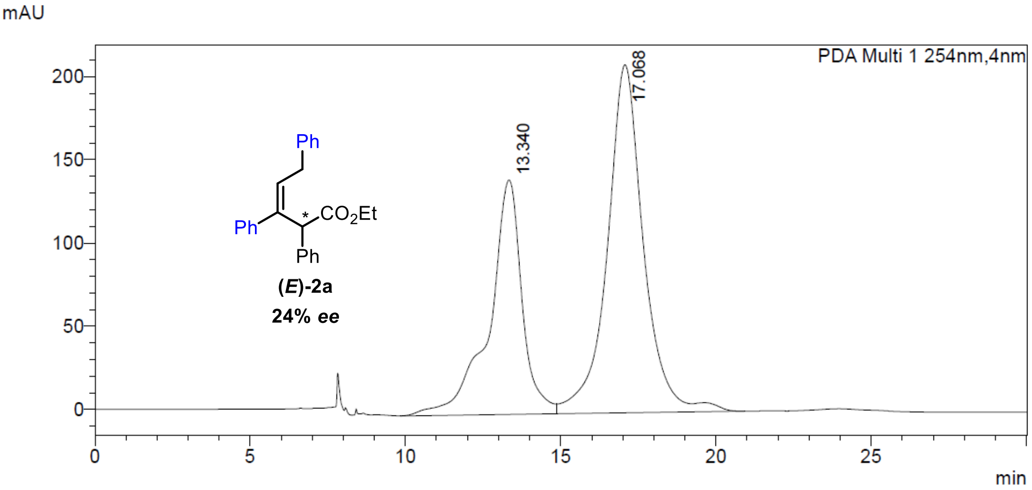

<Peak Table>

PDA Ch1 254nm

Peak#	Ret. Time	Area	Height	Conc.	Unit	Mark	Name
1	13.340	10359401	140874	0.000			
2	17.068	17032855	209151	0.000		V	
Total		27392256	350025				

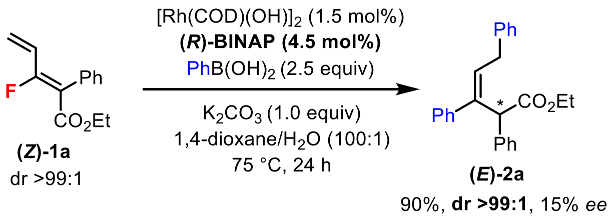

<Chromatogram>

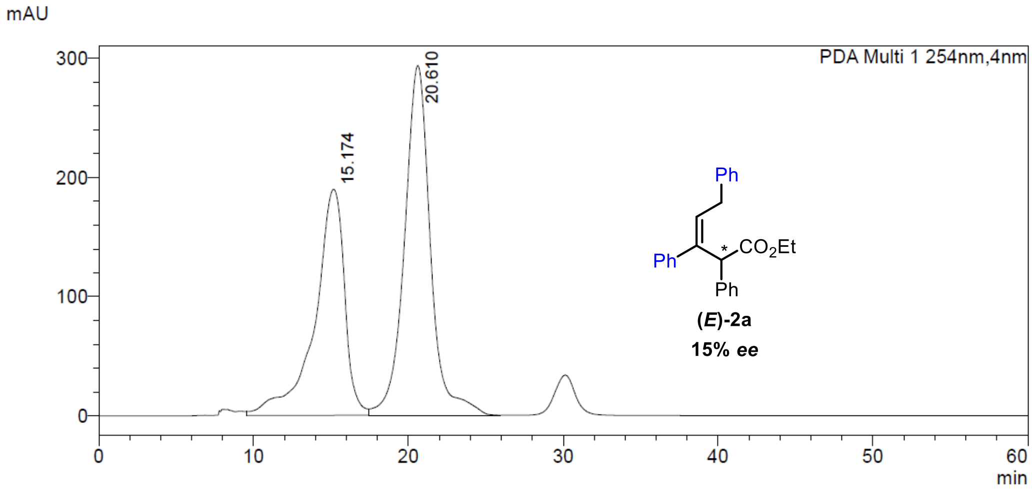

<Peak Table>

PDA Ch1 254nm

Peak#	Ret. Time	Area	Height	Conc.	Unit	Mark	Name
1	15.174	25524276	189508	0.000			
2	20.610	34290810	293235	0.000		V	
Total		59815086	482743				

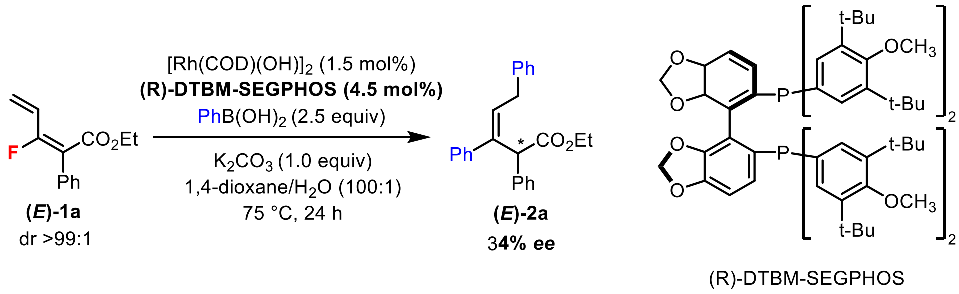

<Chromatogram>

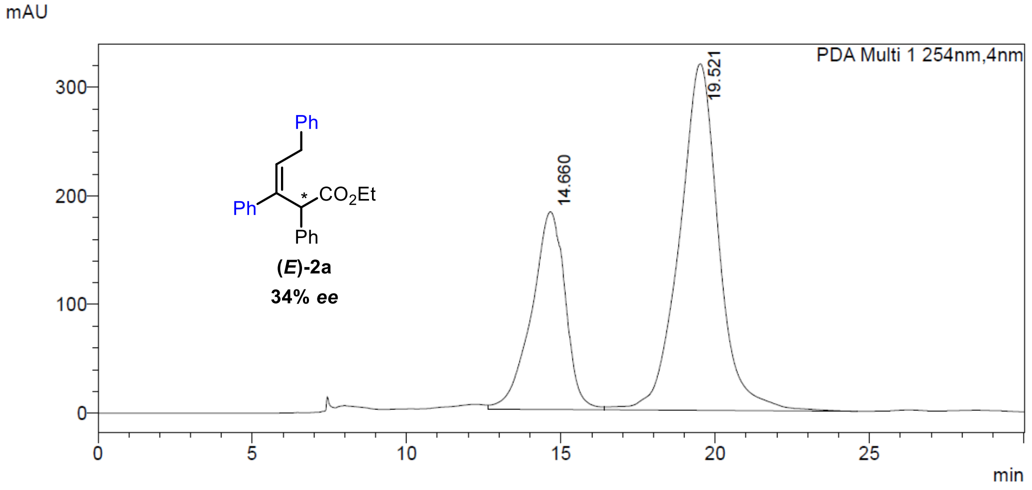

<Peak Table>

PDA Ch1 254nm

Peak#	Ret. Time	Area	Height	Conc.	Unit	Mark	Name
1	14.660	13830225	181969	0.000			
2	19.521	28190770	318953	0.000		V	
Total		42020995	500921				
